# The impact of various seed, accessibility and interaction constraints on sRNA target prediction- a systematic assessment

**DOI:** 10.1186/s12859-019-3143-4

**Published:** 2020-01-13

**Authors:** Martin Raden, Teresa Müller, Stefan Mautner, Rick Gelhausen, Rolf Backofen

**Affiliations:** 1 Bioinformatics Group, Department of Computer Science, University of Freiburg, Georges-Koehler-Allee 106, Freiburg, 79110 Germany; 2 Signalling Research Centres BIOSS and CIBSS, University of Freiburg, Schaenzlestr. 18, Freiburg, 79104 Germany

**Keywords:** RNA-RNA interaction, sRNA, Target prediction, Seed, Accessibility, Constraints

## Abstract

**Background:**

Seed and accessibility constraints are core features to enable highly accurate sRNA target screens based on RNA-RNA interaction prediction. Currently, available tools provide different (sets of) constraints and default parameter sets. Thus, it is hard to impossible for users to estimate the influence of individual restrictions on the prediction results.

**Results:**

Here, we present a systematic assessment of the impact of established and new constraints on sRNA target prediction both on a qualitative as well as computational level. This is done exemplarily based on the performance of IntaRNA, one of the most exact sRNA target prediction tools. IntaRNA provides various ways to constrain considered seed interactions, e.g. based on seed length, its accessibility, minimal unpaired probabilities, or energy thresholds, beside analogous constraints for the overall interaction. Thus, our results reveal the impact of individual constraints and their combinations.

**Conclusions:**

This provides both a guide for users what is important and recommendations for existing and upcoming sRNA target prediction approaches.We show on a large sRNA target screen benchmark data set that only by altering the parameter set, IntaRNA recovers 30% more verified interactions while becoming 5-times faster. This exemplifies the potential of seed, accessibility and interaction constraints for sRNA target prediction.

## Background

Prediction of RNA-RNA interactions is a versatile approach to detect putative targets of non-coding RNAs [1]. Accessibility-based approaches combine the prediction of a most stable interaction duplex with an energy penalty for making the interaction regions accessible, i.e. free of intra-molecular structure. They are very good compromise between the computational complex prediction of joint structures and a simple detection of stable duplexes. While accessibility-based approaches that further incorporate seed constraints are best suited for in silico target screens of prokaryotic sRNAs [2], available programs implement different (sets of) constraints and respective thresholds to increase the prediction accuracy. Although there are various studies that compare tools (based on their default parameters) [2–4], so far no study investigated the impact and potential of the different constraints in a systematic way. This is needed to both provide users with an intuition how the constraints influence the prediction results and to guide the development and improvement of current and future tools.

Accessibility-based approaches can be split into two classes based on the applied accessibility model. The site-based approaches, like RNAup [5], IntaRNA [6, 7] or RIsearch2 [8], compute and use explicit unpaired probabilities for the interacting subregions. While this is exact, the precomputation time and space consumption grows with the maximal length of considered interactions. Therefore, position-based approaches, like RNAplex [9], AccessFold [10] or RIblast [11], estimate the regions’ accessibility based on unpaired probabilities of enclosed single positions. This requires less precomputation but provides only approximate accessibility profiles and thus energy values.

Methods that incorporate seed constraints can also be grouped into approaches that use dynamic programming schemes operating on whole sequences, like IntaRNA, or two-step approaches, like RIblast, RIsearch2 or sTarPicker [12], that first identify putative seed interactions and subsequently find the optimal interaction among low energy seed extensions. Due to the low number of putative seeds, seed-extension approaches consider only a sparse subset of all potential interactions and are as such much faster than exhaustive dynamic programming schemes.

Within this study, we do a systematic evaluation of established and new constraints for RNA-RNA interaction prediction for their potential to improve sRNA target prediction. Beside a qualitative assessment, we also investigate respective runtime effects since target screens are typically done on a genomic level [13–15] and therefore time intensive. In detail, we are investigating the following constraints and combinations:

- Seed constraints:
  - seed vs. no seed
  - number of seed base pairs
  - prohibition of GU base pairs in seeds
  - maximal overall energy of seeds
  - maximal hybridization energy of seeds
  - minimal accessibility (unpaired probability) of seed regions
- Interaction constraints:
  - maximal energy of an interaction
  - minimal accessibility (unpaired probability) of interacting regions
  - maximal interaction length (region per RNA)
  - maximal interior loop size
  - impact of in silico SHAPE data from ShaKer
  - energy parameter model

## Results and discussion

Within this study, we report as a quality assessment the relative number of recovered verified sRNA-target pairs among the top-100 predictions for each tested sRNA. Furthermore, relative overall runtime of each parameter benchmark is depicted. The runtime normalization is done using the default parameter setup of IntaRNA v2.3.1, which we extended with additional constraints tested here. If not set explicitly, IntaRNA’s default values for version 2 are: 7 (canonical) base pairs in seed, allowing for GU base pairs in seed, maximal overall energy of seed or interaction of 0 kcal/mol, minimal unpaired probability of seed or interaction site of 0, maximal interaction length of 150, maximal interior/bulge loop size of 16. To reduce the parameter space, we consider only canonical seeds, i.e. seed interactions that show consecutive stackings only. For each tested parameter setting, we report the recovery for each reference target within the Additional file 1. Abbreviations in figures and text are based on respective IntaRNA parameter names.

### Seed constraint - length of the seed

The length of considered seed interactions, i.e. the number of consecutively stacked base pairs, is one of the first and most central feature of most sRNA target prediction tools as it has a strong impact on the size of the search space and prediction quality.

While tools like IntaRNA [6, 7] or TargetRNA(2) [16, 17] require 7 base pairs, other approaches as RIsearch2 [8], RIblast [11] or sTarPicker [12] are less restrictive and require only 6, 5 or at least 5 (with additional constraints), respectively. Similar constraints are also applied in the context of eukaryotic microRNAs [8, 18].

**Fig. 1:**
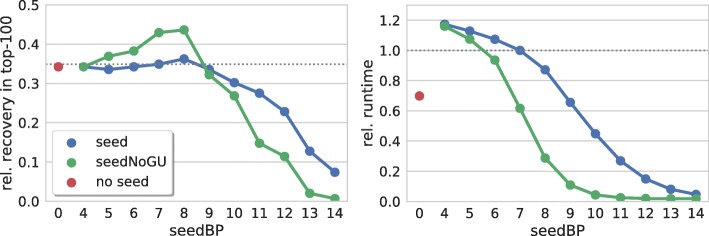
Seed length and GU base pairs. (left) Relative number of recovered verified targets among the top-100 predictions of each sRNA for different seed lengths (seedBP) with and without GU base pairs, i.e. blue = [seed] and green = [seedNoGU], resp., where red = [no seed] refers to results without seed constraints. (right) Relative overall runtime to process each parameter set. The dotted gray lines mark respective values of IntaRNA with default parameters

Figure 1 summarizes the results for various seed lengths using IntaRNA. A seed length of 8 shows the best prediction results while further increasing the required base pairs results in a rapid performance loss. These results are in line with [16]. Lower values provide similar results but require, due to the increased search space, more runtime. Overall, we observe no tremendous impact of the seed length (below the critical length of 9) on the prediction accuracy when compared to predictions that do not require a seed interaction. Note, the increased runtime when enforcing seed constraints is a result of the two-step recursions implemented by IntaRNA version 1 and version 2 and can be drastically reduced when applying a seed-extension strategy e.g. implemented in RIblast, RIsearch2 or the recent IntaRNA version 3. Still, the same trends caused by seed length constraints apply due to the inverse relation of seed length and the number of respective seeds to be processed.

### Seed constraint - avoiding weak GU base pairs in seeds

GU base pairings are the weakest among the considered base pairings in secondary structure energy models. Since a seed interaction is considered to provide a strong initial binding platform for interaction formation, reducing [12] or even completely forbidding GU base pairs in seeds is used by some approaches [19].

In Fig. 1 we show that forbidding GU base pairs in seeds indeed has a strong impact on both prediction accuracy and runtime. While the same trends apply (increase to maximum at 8 base pairs with a subsequent rapid drop for ≥9), significantly more verified interactions are recovered when compared to respective parameter sets that allow for GU base pairs. Furthermore, we observe a strong runtime reduction since many putative seeds are filtered by this constraint.

### Seed constraint - enforcing stable (low energy) seeds

Reducing the number of GU base pairs in seeds, as investigated above, is an indirect constraint on the stability of seeds to be considered for interaction prediction. Thus, some approaches introduced constraints on the seeds energy [7, 12], which is a measure of its thermodynamic stability. The rational here is that a stable seed interaction should provide a good platform for a subsequent interaction formation that is also kinetically favoured. Both restrictions on the overall seed energy [7] or the seeds’ hybridization energy [8, 11] are known from the literature.

When restricting the overall energy of seeds, we constrain a mixture of hybridization energy terms and the accessibility penalties of the seeds’ interaction site. Both are connected with the seed length (longer seeds provide lower hybridization energies and higher accessibility penalties) and thus energy constraints are seed-length specific. Here, we exemplarily investigate the effect of (hybridization) energy bounds on seeds of length 7. Investigations of seed accessibility constraints are discussed in a dedicated subsequent section.

**Fig. 2:**
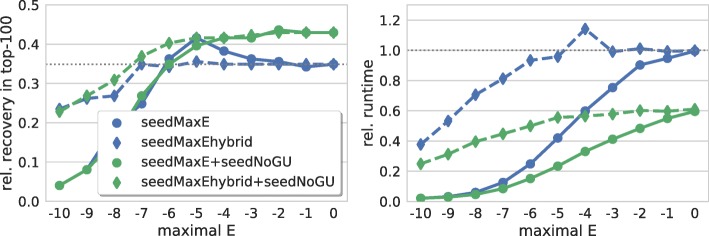
Seed stability. (left) Relative number of recovered verified targets among the top-100 predictions of each sRNA for different thresholds on a seed’s overall energy and hybridization energy, i.e. ball = [seedMaxE] and diamond = [seedMaxEhybrid], respectively. Results including GU base pairs are in blue while values for seeds without GU base pairs are in green. (right) Relative overall runtime to process each parameter set. The dotted gray lines mark respective values of IntaRNA with default parameters

Our results, depicted in Fig. 2, show that constraining the overall energy enables higher prediction accuracy (maximum at about −5 kcal/mol for 7 seed base pairs) while offering significant runtime reductions. In contrast, constraining only the seeds’ hybridization energy provides no significant prediction gain and the recovery drops for thresholds below −7 kcal/mol.

Given the superior results for seeds without GU base pairs from the last section, we also investigated the impact of energy thresholds for predictions confined to such seeds. Disallowing GU base pairs should directly relate to more stable seeds within the underlying energy model. In contrast to the discussed results for seeds including GU base pairs, a (non-significant) maximal recovery is observed for −2 kcal/mol both for overall and hybridization-only energy thresholds for no-GU-base-pair seeds. For both constraints, the recovery rate drops with decreasing maximal energy values. Also in contrast to the GU-including results, thresholds on overall seed energies are not superior to constraints on hybridization-only energies of seeds without GU base pairs. Overall, we conclude that forbidding GU base pairs has similar maximal effects than restricting the overall energy of seeds including GU base pairs.

### Seed constraint - accessibility of seed regions

Given our results concerning seed stability, we next investigated the impact of the accessibility of the seed regions, i.e. constraining considered seeds to sequence regions that are likely unpaired using increasing thresholds. Such a constraint follows the hypothesis that the initial interactions are formed between highly accessible (unstructured) regions of the two RNAs, which subsequently expand into the full interaction. This should again result in interactions that are kinetically favoured.

**Fig. 3:**
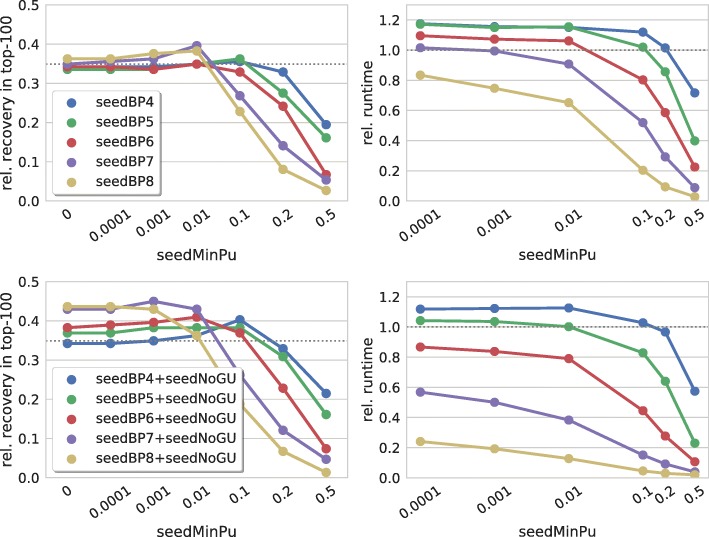
Seed accessibility. (left) Relative number of recovered verified targets among the top-100 predictions of each sRNA for different thresholds on a seed’s accessibility in terms of minimal unpaired probability of both seed regions [seedMinPu] for different seed lengths [seedBP]. (right) Relative overall runtime to process each parameter set. The (top) and (bottom) graphs show results when GU base pairs are allowed or forbidden in seeds, respectively. The dotted gray lines mark respective values of IntaRNA with default parameters

Figure 3 (top) visualizes the effect of seed accessibility constraints for different seed lengths (when allowing GU base pairs in seeds). For all seed lengths, a maximum is observed when enforcing a minimal unpaired probability between 0.001 and 0.1. Too restrictive values result in the expected drop in the recovery rate since few to no putative seeds are left for prediction, while too soft thresholds (≤0.01) show no significant effect.

For longer seeds (≥7 bp), we observe a runtime reduction for minimal unpaired probabilities of at least 0.001, which results from the reduced seed set considered for prediction.

When comparing the results for seed length 7 (seedBP7 in Fig. 3) with the seedMaxE results from Fig. 2, a similar (x-reversed) curve shape is observed. This supports our hypothesis that the effects caused by constraining the seed’s overall energy are mainly resulting from the seed’s accessibility, since unpaired probabilities *P*^*u*^ are incorporated as accessibility penalties via −*RT* log(*P*^*u*^).

Figure 3 (bottom) shows respective results for seeds without GU base pairs. While the overall recovery rates are higher, similar trends are observed. This plot also shows that disallowing GU base pairs has stronger effects for longer seeds.

Since the maximal effect of seed accessibility constraints is less independent of the seed length compared to energy constraints, we conclude that constraining the seeds’ accessibility is to be preferred over using energy thresholds on the seed.

### Interaction constraint - maximal interaction length

Next we investigated how constraints on the overall interaction influence sRNA target prediction results. The most stringent restriction limits the interaction’s length, here in terms of the maximal lengths of the subsequences covered by the interaction. This constraints stems from the observation that most known interactions are relatively short, probably due to steric hindrances [20]. Also concerning maximal interaction length, defaults from the available tools differ in their constraints. While IntaRNA uses very soft bounds enabling interactions of up to 150 nt, RNAup predicts only interactions up to 25 nt (due to the vast computational cost of its algorithm) and RIsearch2 restricts the maximal length to 30 nt. Since RNAup and IntaRNA provide similar prediction results [2], it seems sufficient to consider only short interactions for sRNA target prediction.

**Fig. 4:**
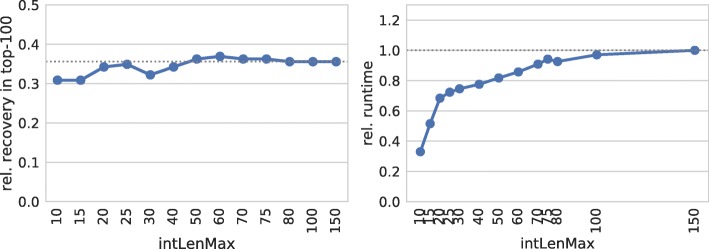
Interaction length. (left) Relative number of recovered verified targets among the top-100 predictions of each sRNA for different thresholds on the overall interaction length. (right) Relative overall runtime to process each parameter set. The dotted gray lines mark respective values of IntaRNA with default parameters

Figure 4 supports this hypothesis. If the maximal interaction size exceeds 50 nt, no significant changes of the recovery rate are observed (60 provides the best results). Shorter interactions result in a reduced number of recovered interactions, which is in accordance with the lower precision (PPV) results of RNAup reported in [2].

As expected, restricting the overall interaction length has a strong impact on the prediction runtime. Thus, we conclude that using a maximal interaction length threshold of about 60 provides a good trade-off between target prediction quality and runtime.

### Interaction constraint - stability (energy) of interactions

Next, as done for seeds, we restricted the minimal stability of the overall interaction, i.e. we set an upper bound on the overall energy of the interaction (covering both hybridization and accessibility terms). This puts a constraint on the minimal (thermodynamic) stability assumed to be needed for regulatory effects of sRNA-target interactions.

**Fig. 5:**
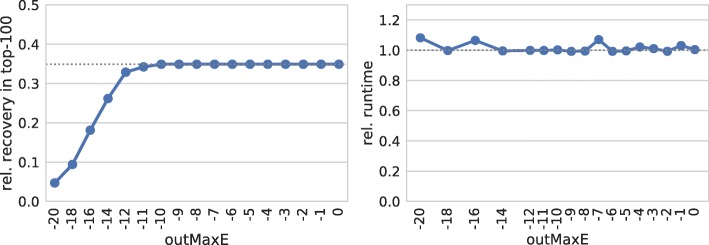
Interaction stability. (left) Relative number of recovered verified targets among the top-100 predictions of each sRNA for different thresholds on the overall interaction energy. (right) Relative overall runtime to process each parameter set. The dotted gray lines mark respective values of IntaRNA with default parameters

We observe (exemplarily for seed length 7) no effect for energy thresholds above −10 kcal/mol, as shown in Fig. 5. Below, the recovery rate drops significantly. Furthermore, no impact on the prediction runtime is found. Thus, we conclude that restricting the interaction stability (via energy thresholds) does not improve sRNA target screens.

This result is surprising on the first sight. There is, however, a possible explanation why constraining interaction energy might have low or no effect. The components of the overall interaction energy, namely hybridization and accessibility terms, are negatively correlated with interaction length. Thus, while expanding interactions typically results in lower hybridization terms, it directly results in increased accessibility penalties. The latter results from the fact that the unpaired probability of a sequence is always lower than or equal to the probability of any of its subsequences. Thus, interactions of very different lengths can show the same overall energy. Therefore, constraining the overall energy shows no effect.

### Interaction constraint - accessibility of interacting regions

Given the results and insights from our interaction stability investigation, we subsequently evaluated the impact of accessibility constraints. This reflects the assumption that fast regulatory effects are due to interactions of accessible regions. Interactions formed by the latter do not require extensive intra-molecular restructuring of the involved RNAs, which might enable even more stable interactions in thermodynamic equilibrium but take much more time to form. Thus, we restrict the minimal unpaired probability of interacting sites.

**Fig. 6:**
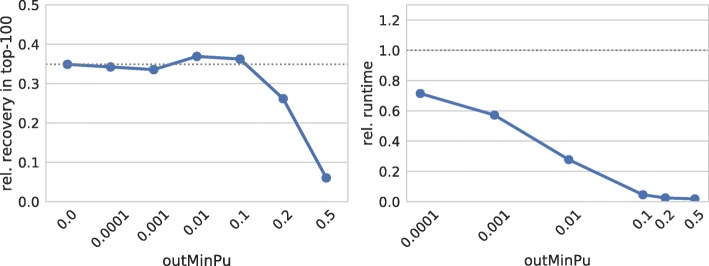
Interaction accessibility. (left) Relative number of recovered verified targets among the top-100 predictions of each sRNA for different thresholds on the minimal unpaired probability (accessibility) of the interacting regions. (right) Relative overall runtime to process each parameter set. The dotted gray lines mark respective values of IntaRNA with default parameters

The comparison of Fig. 6 with Fig. 3 (seedBP7) reveals that the effects of restricting the interaction site’s accessibility are similar to constraining the seed interaction site only. That is, a maximal recovery is observed for a minimal unpaired probability of about 0.01 and higher thresholds result in decreasing prediction performance. In contrast to the seed site results, a much higher runtime reduction is observed, which results from the exclusion of many interaction site combinations.

### Interaction constraint - maximal loop/bulge size

RNA-RNA interaction prediction tools typically restrict the size of interior and bulge loops within the interaction, i.e. the number of unpaired bases between inter-molecular base pairs, to reduce the computational complexity of the prediction. This is done under the hypothesis that a loop’s energy relates to the loop size, i.e. the larger loops show higher energies due to increased structural flexibility. Thus, it is unlikely that very large loops are part of the optimal interaction and thus excluded from the search space.

**Fig. 7:**
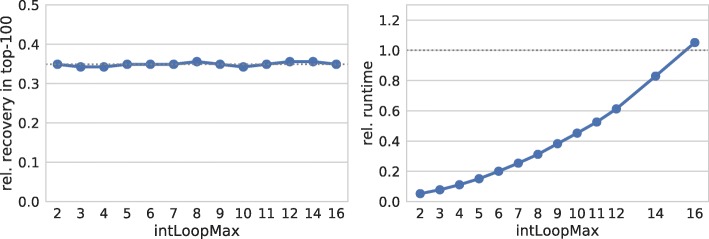
Maximal loop/bulge size. (left) Relative number of recovered verified targets among the top-100 predictions of each sRNA for different thresholds on the maximal number of unpaired bases within inter-molecular interior loops or bulges. (right) Relative overall runtime to process each parameter set. The dotted gray lines mark respective values of IntaRNA with default parameters

In Fig. 7 the quadratic runtime effect of the maximal loop length becomes visible. Surprisingly, we do not observe a significant effect of the loop length on the prediction quality. Even for extremely small loop sizes of 2, the recovery rate does not drop.

These findings imply, that most of the top ranked interactions of the target screen are mainly composed of nearly perfect stackings. Thus, disallowing large loops has no effect. Nevertheless, these findings are not considering other constraints beside that the seed has to show 7 base pairs. When combined with other restrictive constraints, we observe a drop in the recovery rate when the interior loop length falls below 8 (data not shown).

### General settings - ShaKer-enhanced accessibility prediction

IntaRNA can integrate data from structure probing protocols such as dms [21] or SHAPE [22], which can improve the assumed accessibility profiles and eventually the predicted interactions [23]. Since this data is unavailable in the general case, especially in the context of target prediction on a genome wide level, we investigated the impact of SHAPE data predicted by the recent machine learning approach ShaKer [24]. It was shown that SHAPE data predicted by ShaKer improves the accessibility profile prediction of individual RNAs. Since the latter is a key feature of RNA-RNA interaction prediction, using ShaKer should eventually improve sRNA target prediction. We investigated three scenarios how SHAPE data predicted by ShaKer is used: (a) for sRNA sequences only, (b) for target sequences only, and (c) using predicted SHAPE data for both sequence sets.

When using ShaKer with the original prediction model that was trained on a small data set of 16 RNAs with known SHAPE data and confirmed structures [24], we observe (a) a reduced recovery when applied to sRNAs only (4 verified sRNA-target pairs less recovered), (b) an improvement of +4 pairs when used on targets only, and (c) no change when applied to both.

Recently, a larger SHAPE data set has been published by A. Mustoe [25] covering 194 RNAs for which no confirmed structure is available. We predicted putative structures via RNAfold [26] using the SHAPE data and trained a new ShaKer model for SHAPE prediction. Using this model, we observe (a) one less recovered pair when applied to sRNAs only, (b) the same improvement (+4) as for the old model when used on targets only, and (c) one additional verified sRNA-target pair was recovered when applied to both.

These results suggest that especially the accessibility profiles of target sequences, which are genomic subsequences around the start codons, can be improved with in silico SHAPE data. Already, the ShaKer models show promising results even though both were trained on tentative data sets; one containing only 16 sequences, the other without reliable structure information. With better training data we expect even better results.

### General settings - energy parameter set

So far, all predictions were based on the energy parameters introduced by the Turner lab in 2004 [27], since most RNA structure or RNA-RNA interaction prediction tools are using these parameters.

Here, we tested the performance of other energy parameter sets (that are supported and shipped with the Vienna RNA package [28]). This covers beside (i) the Turner-2004 parameters [27] (ii) the old Turner-1999 model [29], (iii) the Andronescu-2007 model [30], and (iv) Langdon-2018 [31]. While the latter two are in silico models based on parameter optimization for a large RNA data set, both Turner lab models are also incorporating experimental data.

Eventually, all models provided a better recovery than the Turner-2004 model. In detail, we we observe an increase in the number of recovered sRNA-target pairs (ii) of +3 for Turner-1999, (iii) of +5 for Andronescu-2007, and (iv) of +4 when using the Langdon-2018 energy parameters.

These results show that– in accordance with expectation –target prediction results are sensitive to the used energy parameter set. Surprisingly, both in silico models (iii + iv) provide similar performance, i.e. the improved RNA structure prediction accuracy of Langdon-2018 over Andronescu-2007 does not translate to sRNA target prediction.

### Overall recommendations

Finally, we tested a final parameter combination that was compiled based on the individual benchmarks, which provides (limited) insights concerning the interplay of the different constraints tested. These results provide recommendations for users on how to constrain the RNA-RNA interaction prediction tool at hand for most efficient sRNA target prediction. Furthermore, this is useful for algorithm and software developers to further improve the available programs.

Given our results, we recommend the following constraints:

- canonical seeds of 7 base pairs
- no GU base pairs in seed
- minimal unpaired probability of 0.001 of both seed sites
- maximal interaction length of 60
- maximal interior/bulge loop size of 8
- minimal unpaired probability of 0.001 of both interaction sites

**Fig. 8:**
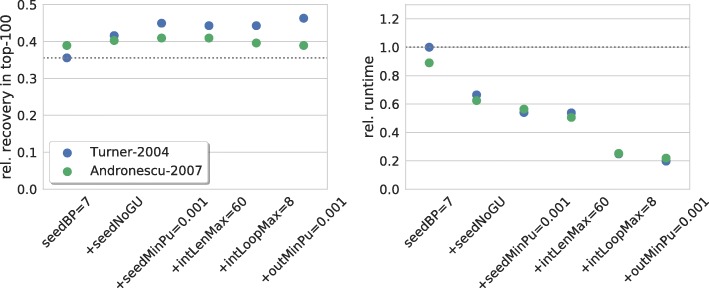
Overall recommendations. (left) Relative number of recovered verified targets among the top-100 predictions of each sRNA for an increasing set of recommended constraints, i.e. the constraints accumulate from left to right. (right) Relative overall runtime to process each parameter set. The dotted gray lines mark respective values of IntaRNA with default parameters

Figure 8 summarizes the results for increasing sets of the listed constraints for two energy parameter sets, namely Turner-2004 and Andronescu-2007. For the Turner model, the overall constraint set provides 30% more verified targets within 20% of the runtime. Constraints on the seed only provide already a recovery improvement of 26% in half the computation time. Further constraints on the overall interaction mainly reduce runtime with the exception of the minimal accessibility of the interaction site, which finally improves the recovery rate to its maximum.

We observe the same runtime behaviour for Andronescu-2007 as for the Turner-2004 model but the impact on the recovery is much less. While seed constraints still provide improvements, interaction constraints do not increase the recovery rate. This shows that (parts of) our recommendation are restricted to the Turner model. It remains open whether the Andronescu-2007 model provides less potential for improving sRNA target prediction results or if our recommended values are not suited for this model and need a dedicated investigation and optimization.

### Comparison to alternative tools

To test whether the observations for IntaRNA translate to other sRNA target prediction tools, we applied TargetRNA2 [17] and RIsearch2 version 2.1 [8], since both tools support seed constraints. Other approaches like RNAup or RNAplex with high prediction accuracy [2] allow only for the restriction of interaction length, for which we did not observe significant impact on prediction quality (see above), such that they were omitted from comparison.

For TargetRNA2, only a webserver is available, which supports the restriction of seed and overall interaction length. Since the webserver does not support direct target sequence upload, we selected the respective organisms and set target sequence extraction to the values used for our data set. Due to time-outs and thus no results for many parameter setups tested within this study, only limited results can be reported. RIsearch2 allows to constrain the number of seed base pairs and whether or not GU base pairs are allowed within the seed. The overall interaction length cannot be confined, only the maximal seed extension (per side). Since RIsearch2 implements a very simplified energy model, constraints on the overall interaction energy cannot be well related to IntaRNA results. Given these observations, comparison was restricted to seed length and seed stability in terms of prohibition of GU base pairs within seeds.

**Fig. 9:**
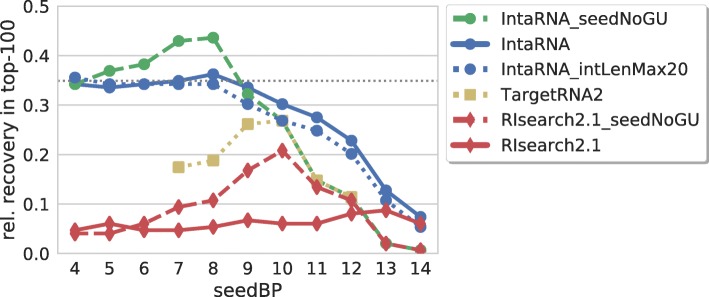
Seed-length impact for different sRNA target prediction tools. Relative number of recovered verified targets among the top-100 predictions of each sRNA for various seed lengths and tools. Since TargetRNA2 defaults restrict the maximal interaction length to 20, comparable results using a similar constraint for IntaRNA are shown by IntaRNA_intLenMax20. The dotted gray line marks the recovery of IntaRNA with default parameters

The results are provided in Fig. 9. The plot shows the overall superiority of IntaRNA and validates that prohibiting GU base pairs within seeds can significantly improve prediction accuracy. The latter is in accordance with the sensitivity analysis for TargetRNA(1) [19]. Since we see a high correlation of the seedNoGU recovery results of IntaRNA and RIsearch2 with the values of TargetRNA2, we assume that the latter also applies per default a ’seed-no-GU’ constraint, which is neither documented within the respective literature or webserver nor available as webserver option. In contrast to IntaRNA, both competitors yield highest recoveries for seed lengths of 9-10. Since both tools apply simplified energy models to speedup predictions, these results suggest that such models benefit from stronger seed constraints to reduce false positive predictions.

## Conclusions

The identification of putative sRNA targets based on RNA-RNA interaction prediction tools is often complicated due to the false positives (non-targets). Thus, different constraints have been proposed to improve the prediction results. Most successful was the incorporation of the interaction sites’ accessibility and the requirement for stable seed subinteraction [2]. While available tools implement different (combinations of) constraints and default thresholds, it remains unclear which constraints and values are most effective and which are less important. Thus, we focus on accessibility-based RNA-RNA interaction approaches with seed constraints like IntaRNA, RIsearch2, or RIblast.

Here, we investigated the impact of various constraints on seed, interaction and accessibility features to tackle this problem. The benchmark is exemplarily done using IntaRNA, which provides a flexible framework to test and combine different constraints. Using a single tool enables a comparability of the results and thus an abstraction from the absolute IntaRNA-specific performance to general trends induced by the respective constraints.

Thus, the benchmark is based on an sRNA target screen pipeline for two organisms. While this limits the generality of the study, it allows for a thorough investigation of the effects caused by the different parameter sets. Since most interaction details from the literature are based on single, arbitrary RNA-RNA interaction prediction tools, it is currently hard to impossible to evaluate the correctness of reported interaction details on a large scale. The prediction quality is assessed in terms of recovery of verified sRNA-target pairs from the literature rather than evaluation on an inter-molecular base pair level following [6, 19, 20]. That way, a lower bound on the true positives within the top-ranked predictions is measured.

In our study, we observed that seeds of length 7-8 provide the best results, which can be significantly improved when disallowing GU base pairs. These results are in line with but much simpler than the complicated seed-length-dependent GU/GC-content handling of sTarPicker [12]. Furthermore, our results suggest that the efforts done in RIsearch2 [8], to allow for GU base pairs within seeds, might be not needed and thus even better runtime and prediction performance might be possible. We conclude that disallowing GU base pairs in seeds provide a powerful constraint on the seed stability that is much less dependent on the seed length when compared to seed energy constraints. Thus, ’no GU seed base pairs’ is more general and its application is less likely to cause an overfitting of the used threshold value. Furthermore, we could show that the accessibility of the seed site is also important for the precision of the target prediction. This supports the hypothesis that seed interactions indeed relate to an initial stable subinteraction that subsequently grows into the final overall interaction. Finally, we have shown that low bounds on the maximal interaction length as well as the size of inter-molecular loops still allow high quality predictions while providing strong runtime reductions. The latter outcome is restricted to approaches without early stop criteria as implemented e.g. in RIblast. Eventually, we could show that sRNA target prediction can be significantly improved just by changing the parameter set. That is for IntaRNA we can recover 30% more verified sRNA-target pairs within only 20% of the runtime with appropriate parameters.

## Methods

### Formal preliminaries

We are focusing on accessibility-based RNA-RNA interaction prediction. To this end, an accessibility profile for each RNA *S* has to be computed, which is typically based on unpaired probabilities *P*^*u*^(*i*..*j*) [32, 33] for respective subsequences *S*_*i*..*j*_. These translate into accessibility penalty terms *ED*=−*RT* log(*P*^*u*^) (with gas constant *R* and temperature *T*) that encode how much energy is needed to free the respective subsequence from intra-molecular base pairing to enable interaction formation. The stability of an RNA-RNA interaction $I^{i..j}_{k..l}$ is then evaluated based on the sum of its hybridization energy $E_{h}(I^{i..j}_{k..l})$ defined by its inter-molecular base pairs and two accessibility penalties $ED^{1}_{i..j}$ and $ED^{2}_{k..l}$ for each RNA, respectively. Both energy and unpaired probability computation are based on the same nearest-neighbor energy model for non-crossing secondary structures using a given set of energy parameters (e.g. Turner-2004 [27]). Within this study, we consider only interactions *I* that contain a seed subinteraction *I*_*s*_, which is here defined as a canonical helix formed by a defined number of base pairs (named the seed length). For further formal details on the energy model, probability computation, and technical details of the approaches we refer to [6, 34, 35].

### Data set

Within this study, we use the benchmark data set and pipeline that we introduced in [20], which enables sRNA target screens for both *Echericha coli* (GenBank accession number NC_000913) and *Salmonella typhimurium* (NC_003197). The data set consists of homologous sequences of 15 sRNAs expressed in both organisms. As all these sRNAs have been shown to regulate translation of their targets via RNA-RNA interaction near the start codon [1, 15], we are mostly interested in interactions for these regions. Thus, target sequences are compiled by extracting the genomic region from 200 nt upstream up to 100 nt downstream of the start codon of each protein-coding gene. The data set contains 4,319 and 4,552 targets for *E.coli* and *S. typhimurium*, respectively. Furthermore, we extracted 149 experimentally verified sRNA-target pairs from the literature (Additional file 1), which we want to recover within the benchmark.

### Benchmarking pipeline

To measure the prediction performance to compare different constraints and parameter sets, we follow the pipeline used in [6, 19, 20]. That is, we run IntaRNA for each sRNA-target combination and store the respective minimal free energy of the most stable interaction. For each sRNA, we identify the 100 targets with the most stable interaction (lowest energy) and accumulate how many of the verified interactions are among these top-100 predictions (detailed recovery information within the Additional file 1). This number of recoveries provides a lower bound on the number of true targets within the top-100 predictions of all sRNAs. If a constraint or parameter set reduces the recovery rate, this can either be based on (i) an increase of false positive predictions, (ii) a decrease of true positives (verified interactions) among the top ranks or (iii) a combination of both, which can not be distinguished.

Computational performance is measured via the overall runtime needed to run the benchmark once for all sRNAs and organisms for a given parameter set. This directly relates to the computational cost of in silico target screens. Runtimes exclude accessibility computation (using precomputed unpaired probabilities) if not stated differently.

### ShaKer-based precomputations

ShaKer trains a model on triplets of sequence, structure and SHAPE data. The sequence and structure form a graph whose nodes are vectorized via a graph kernel scheme [36]. Together with SHAPE reactivity values as targets, a regression model is trained. For the prediction multiple structures are sampled [28] and annotated by the model. These annotations are weighted by the probabilities of the structures to obtain the final reactivity values for a sequence.

## Supplementary information

**Additional file 1** Supplementary material — table of verified sRNA-target pairs and their recovery for tested parameter settings.

## Data Availability

The datasets generated and/or analysed during the current study are available in the github repository, https://github.com/BackofenLab/IntaRNA-benchmark, initially published in [20], and the Additional file 1.
