# Supplementary material for: The impact of various seed, accessibility and interaction constraints on sRNA target prediction- a systematic assessment
Source: BMC Bioinformatics. 2020 Jan 13;21:15. doi: 10.1186/s12859-019-3143-4 (PMC6956497; doi:10.1186/s12859-019-3143-4)
Supplement: Supplementary file 1 — Additional file 1 Supplementary material — table of verified sRNA-target pairs and their recovery for tested parameter settings. [file 12859_2019_3143_MOESM1_ESM.pdf]

Verified sRNA-target pairs from literature

sRNA	geneID	geneName	genome	referenceDOI
ArcZ	b1892	flhD	NC_000913	https://doi.org/10.1111/j.1365-2958.2012.08209.x
ArcZ	b2741	rpoS	NC_000913	https://doi.org/10.1038/emboj.2010.179
ArcZ	b3546	eptB	NC_000913	https://doi.org/10.1111/mmi.12257
ChiX	b0619	dpiB/citA	NC_000913	https://doi.org/10.1111/j.1365-2958.2009.06665.x
ChiX	b0681	chiP	NC_000913	https://doi.org/10.1099/mic.0.023598-0
ChiX	b1737	chbC	NC_000913	https://doi.org/10.1111/j.1365-2958.2009.06807.x
CyaR	b0723	sdhA	NC_000913	https://doi.org/10.1073/pnas.1303248110
CyaR	b0814	ompX	NC_000913	https://doi.org/10.1128/JB.01157-08
CyaR	b1740	nadE	NC_000913	https://doi.org/10.1128/JB.01157-08
CyaR	b1824	yobF	NC_000913	https://doi.org/10.1073/pnas.1303248110
CyaR	b2416	ptsI	NC_000913	https://doi.org/10.1073/pnas.1303248110
CyaR	b2666	yqaE	NC_000913	https://doi.org/10.1128/JB.01157-08
CyaR	b2687	luxS	NC_000913	https://doi.org/10.1128/JB.01157-08
DsrA	b1237	hns	NC_000913	https://doi.org/10.1073/pnas.95.21.12456
DsrA	b2741	rpoS	NC_000913	https://doi.org/10.1073/pnas.95.21.12456
DsrA	b3251	mreB	NC_000913	https://doi.org/10.1039/c4ib00102h
FnrS	b0723	sdhA	NC_000913	https://doi.org/10.1073/pnas.1303248110
FnrS	b0755	gpmA	NC_000913	https://doi.org/10.1111/j.1365-2958.2010.07044.x
FnrS	b0887	cydD	NC_000913	https://doi.org/10.1074/jbc.M109.089755
FnrS	b1107	nagZ	NC_000913	https://doi.org/10.1073/pnas.1303248110
FnrS	b1479	maeA	NC_000913	https://doi.org/10.1111/j.1365-2958.2010.07044.x
FnrS	b1531	marA	NC_000913	https://doi.org/10.1073/pnas.1303248110
FnrS	b1656	sodB	NC_000913	https://doi.org/10.1074/jbc.M109.089755
FnrS	b1841	yobA	NC_000913	https://doi.org/10.1074/jbc.M109.089755
FnrS	b2153	folE	NC_000913	https://doi.org/10.1111/j.1365-2958.2010.07044.x
FnrS	b2303	folX	NC_000913	https://doi.org/10.1111/j.1365-2958.2010.07044.x
FnrS	b2531	iscR	NC_000913	https://doi.org/10.1073/pnas.1303248110
FnrS	b3829	metE	NC_000913	https://doi.org/10.1074/jbc.M109.089755
FnrS	b3908	sodA	NC_000913	https://doi.org/10.1074/jbc.M109.089755
GcvB	b1040	csgD	NC_000913	https://doi.org/10.1111/j.1365-2958.2012.07976.x
GcvB	b1130	phoP	NC_000913	https://doi.org/10.1371/journal.pgen.1003156
GcvB	b3089	sstT	NC_000913	https://doi.org/10.1099/mic.0.023598-0
GcvB	b4208	cycA	NC_000913	https://doi.org/10.1099/mic.0.023598-0
MicA	b0411	tsx	NC_000913	https://doi.org/10.1073/pnas.1109379108
MicA	b0814	ompX	NC_000913	https://doi.org/10.1073/pnas.1109379108
MicA	b0957	ompA	NC_000913	https://doi.org/10.1101/gad.354405
MicA	b1130	phoP	NC_000913	https://doi.org/10.1111/j.1365-2958.2010.07115.x
MicC	b2215	ompC	NC_000913	https://doi.org/10.1128/JB.186.20.6689-6697.2004
MicF	b0241	phoE	NC_000913	https://doi.org/10.1111/j.1365-2958.2012.07994.x
MicF	b0889	lrp	NC_000913	https://doi.org/10.1111/j.1365-2958.2012.07994.x
MicF	b0929	ompF	NC_000913	https://doi.org/10.1128/jb.178.12.3650-3653.1996
MicF	b3912	cpxR	NC_000913	https://doi.org/10.1111/j.1365-2958.2012.07994.x
OxyS	b1892	flhD	NC_000913	https://doi.org/10.1111/j.1365-2958.2012.08209.x
OxyS	b2731	fhlA	NC_000913	https://doi.org/10.1093/emboj/17.20.6069
RprA	b1040	csgD	NC_000913	https://doi.org/10.1111/j.1365-2958.2012.08002.x
RprA	b1341	ydaM	NC_000913	https://doi.org/10.1111/j.1365-2958.2012.08002.x
RprA	b2741	rpoS	NC_000913	https://doi.org/10.1046/j.1365-2958.2002.03203.x
RybB	b0081	mraZ	NC_000913	https://doi.org/10.1073/pnas.1303248110
RybB	b0721	sdhC	NC_000913	https://doi.org/10.1101/gad.182493.111
RybB	b0805	fiu	NC_000913	https://doi.org/10.1073/pnas.1109379108
RybB	b1256	ompW	NC_000913	https://doi.org/10.1016/j.jmb.2006.09.004
RybB	b2215	ompC	NC_000913	https://doi.org/10.1016/j.jmb.2006.09.004
RybB	b2594	rluD	NC_000913	https://doi.org/10.1073/pnas.1109379108
RyhB	b0118	acnB	NC_000913	https://doi.org/10.1093/nar/gku649
RyhB	b0156	erpA	NC_000913	https://doi.org/10.1073/pnas.1303248110
RyhB	b0288	ykgJ	NC_000913	https://doi.org/10.1093/nar/gkv1158
RyhB	b0592	fepB	NC_000913	https://doi.org/10.1093/nar/gkv1158
RyhB	b0683	fur	NC_000913	https://doi.org/10.1038/sj.emboj.7601553
RyhB	b0721	sdhC	NC_000913	https://doi.org/10.1101/gad.182493.111
RyhB	b0723	sdhA	NC_000913	https://doi.org/10.1073/pnas.1303248110
RyhB	b0894	dmsA	NC_000913	https://doi.org/10.1093/nar/gkv1158
RyhB	b1107	nagZ	NC_000913	https://doi.org/10.1073/pnas.1303248110
RyhB	b1200	dhaK	NC_000913	https://doi.org/10.1093/nar/gkv1158
RyhB	b1452	yncE	NC_000913	https://doi.org/10.1093/nar/gkv1158
RyhB	b1531	marA	NC_000913	https://doi.org/10.1073/pnas.1303248110
RyhB	b1588	ynfF	NC_000913	https://doi.org/10.1093/nar/gkv1158
RyhB	b1612	fumA	NC_000913	https://doi.org/10.1101/gad.2001711
RyhB	b1656	sodB	NC_000913	https://doi.org/10.1046/j.1365-2958.2003.03727.x
RyhB	b1778	msrB	NC_000913	https://doi.org/10.1371/journal.pone.0063647
RyhB	b1981	shiA	NC_000913	https://doi.org/10.1111/j.1365-2958.2007.05733.x
RyhB	b2069	yegD	NC_000913	https://doi.org/10.1093/nar/gkv1158
RyhB	b2155	cirA	NC_000913	https://doi.org/10.1093/nar/gkv1158
RyhB	b2206	napA	NC_000913	https://doi.org/10.1093/nar/gkv1158
RyhB	b2530	iscS	NC_000913	https://doi.org/10.1038/emboj.2009.116
RyhB	b3365	nirB	NC_000913	https://doi.org/10.1073/pnas.1303248110
RyhB	b3607	cysE	NC_000913	https://doi.org/10.1073/pnas.1007805107
RyhB	b3942	katG	NC_000913	https://doi.org/10.1093/nar/gkv1158
RyhB	b4070	nrfA	NC_000913	https://doi.org/10.1093/nar/gkv1158
RyhB	b4122	fumB	NC_000913	https://doi.org/10.1093/nar/gkv1158
SgrS	b1101	ptsG	NC_000913	https://doi.org/10.1111/j.1365-2958.2006.05288.x
SgrS	b1817	manX	NC_000913	https://doi.org/10.1093/nar/gkq1219
SgrS	b2416	ptsI	NC_000913	https://doi.org/10.1073/pnas.1303248110
Spot42	b0039	caiA	NC_000913	https://doi.org/10.1038/emboj.2012.52
Spot42	b0720	gltA	NC_000913	https://doi.org/10.1016/j.molcel.2010.12.027
Spot42	b0721	sdhC	NC_000913	https://doi.org/10.1101/gad.182493.111
Spot42	b0728	sucC	NC_000913	https://doi.org/10.1073/pnas.1303248110
Spot42	b0757	galK	NC_000913	https://doi.org/10.1101/gad.231702
Spot42	b1136	icd	NC_000913	https://doi.org/10.1073/pnas.1303248110
Spot42	b1302	puuE	NC_000913	https://doi.org/10.1038/emboj.2012.52
Spot42	b1398	paaK	NC_000913	https://doi.org/10.1038/emboj.2012.52
Spot42	b1761	gdhA	NC_000913	https://doi.org/10.1073/pnas.1303248110
Spot42	b1901	araF	NC_000913	https://doi.org/10.1128/JB.00691-16
Spot42	b2221	atoD	NC_000913	https://doi.org/10.1038/emboj.2012.52
Spot42	b2702	srlA	NC_000913	https://doi.org/10.1016/j.molcel.2010.12.027
Spot42	b2715	ascF	NC_000913	https://doi.org/10.1038/emboj.2012.52
Spot42	b2801	fucP	NC_000913	https://doi.org/10.1038/emboj.2012.52
Spot42	b2802	fucI	NC_000913	https://doi.org/10.1016/j.molcel.2010.12.027
Spot42	b3224	nanT	NC_000913	https://doi.org/10.1038/emboj.2012.52
Spot42	b3566	xylF	NC_000913	https://doi.org/10.1016/j.molcel.2010.12.027
Spot42	b3927	glpF	NC_000913	https://doi.org/10.1038/emboj.2012.52
Spot42	b3962	sthA	NC_000913	https://doi.org/10.1016/j.molcel.2010.12.027
Spot42	b4311	nanC	NC_000913	https://doi.org/10.1016/j.molcel.2010.12.027

sRNA	geneID	geneName	genome	referenceDOI
ArcZ	STM1682	tpx	NC_003197	https://doi.org/10.1111/j.1365-2958.2009.06857.x
ArcZ	STM2970	sdaC	NC_003197	https://doi.org/10.1111/j.1365-2958.2009.06857.x
ArcZ	STM3216	-	NC_003197	https://doi.org/10.1111/j.1365-2958.2009.06857.x
ChiX	STM0687	ybfM/chiP	NC_003197	https://doi.org/10.1101/gad.541609
ChiX	STM1313	celB	NC_003197	https://doi.org/10.1101/gad.541609
CyaR	STM0833	ompX	NC_003197	https://doi.org/10.1111/j.1365-2958.2008.06189.x
GcvB	STM0002	thrA	NC_003197	https://doi.org/10.1111/j.1365-2958.2011.07751.x
GcvB	STM0245	metQ	NC_003197	https://doi.org/10.1111/j.1365-2958.2011.07751.x
GcvB	STM0399	brnQ	NC_003197	https://doi.org/10.1111/j.1365-2958.2011.07751.x
GcvB	STM0602	ybdH	NC_003197	https://doi.org/10.1111/j.1365-2958.2011.07751.x
GcvB	STM0665	gltI	NC_003197	https://doi.org/10.1101/gad.447207
GcvB	STM0959	lrp	NC_003197	https://doi.org/10.1111/j.1365-2958.2011.07751.x
GcvB	STM1299	gdhA	NC_003197	https://doi.org/10.1111/j.1365-2958.2011.07751.x
GcvB	STM1452	tppB	NC_003197	https://doi.org/10.1111/j.1365-2958.2011.07751.x
GcvB	STM1746	oppA	NC_003197	https://doi.org/10.1101/gad.447207
GcvB	STM2355	argT	NC_003197	https://doi.org/10.1101/gad.447207
GcvB	STM2526	ndk	NC_003197	https://doi.org/10.1111/j.1365-2958.2011.07751.x
GcvB	STM3062	serA	NC_003197	https://doi.org/10.1111/j.1365-2958.2011.07751.x
GcvB	STM3064	iciA	NC_003197	https://doi.org/10.1111/j.1365-2958.2011.07751.x
GcvB	STM3225	ygjU/sstT	NC_003197	https://doi.org/10.1111/j.1365-2958.2011.07751.x
GcvB	STM3564	livK	NC_003197	https://doi.org/10.1101/gad.447207
GcvB	STM3567	livJ	NC_003197	https://doi.org/10.1101/gad.447207
GcvB	STM3630	dppA	NC_003197	https://doi.org/10.1101/gad.447207
GcvB	STM3903	ilvE	NC_003197	https://doi.org/10.1111/j.1365-2958.2011.07751.x
GcvB	STM3909	ilvC	NC_003197	https://doi.org/10.1111/j.1365-2958.2011.07751.x
GcvB	STM3930	yifK	NC_003197	https://doi.org/10.1371/journal.pgen.1004026
GcvB	STM4351	-	NC_003197	https://doi.org/10.1101/gad.447207
GcvB	STM4398	cycA	NC_003197	https://doi.org/10.1111/j.1365-2958.2011.07751.x
MicA	STM4231	lamB	NC_003197	https://doi.org/10.1111/j.1365-2958.2007.05829.x
MicC	STM1572	ompD	NC_003197	https://doi.org/10.1038/nsmb.1631
MicF	STM0366	yahO	NC_003197	https://doi.org/10.1111/j.1365-2958.2012.08031.x
MicF	STM0959	lrp	NC_003197	https://doi.org/10.1111/j.1365-2958.2012.08031.x
MicF	STM1328	lpxR	NC_003197	https://doi.org/10.1111/j.1365-2958.2012.08031.x
RybB	STM0413	tsx	NC_003197	https://doi.org/10.1073/pnas.1009784107
RybB	STM0687	ybfM/chiP	NC_003197	https://doi.org/10.1111/j.1365-2958.2010.07342.x
RybB	STM0999	ompF	NC_003197	https://doi.org/10.1073/pnas.1009784107
RybB	STM1070	ompA	NC_003197	https://doi.org/10.1073/pnas.1009784107
RybB	STM1473	ompN	NC_003197	https://doi.org/10.1016/j.molcel.2008.10.027
RybB	STM1530	-	NC_003197	http://www.bioinf.uni-freiburg.de/Publications/Wright:Thesis:2012.pdf
RybB	STM1572	ompD	NC_003197	https://doi.org/10.1073/pnas.1009784107
RybB	STM1732	ompW	NC_003197	https://doi.org/10.1073/pnas.1009784107
RybB	STM1995	ompS	NC_003197	https://doi.org/10.1073/pnas.1009784107
RybB	STM2267	ompC	NC_003197	https://doi.org/10.1073/pnas.1009784107
RybB	STM2391	fadL	NC_003197	https://doi.org/10.1073/pnas.1009784107
SgrS	STM2945	sopD	NC_003197	https://doi.org/10.1073/pnas.1119414109
SgrS	STM3962	yigL	NC_003197	https://doi.org/10.1016/j.cell.2013.03.003
Spot42	STM2190	mglB	NC_003197	https://doi.org/10.15252/embj.201593360

Echericha coli NC_000913	sum	ArcZ-b1892	ArcZ-b2741	ArcZ-b3546	ChiX-b0619	ChiX-b0681	ChiX-b1737	CyaR-b0723	CyaR-b0814	CyaR-b1740	CyaR-b1824	CyaR-b2416	CyaR-b2666	CyaR-b2687	DsrA-b1237	DsrA-b2741	DsrA-b3251	FnrS-b0723	FnrS-b0755	FnrS-b0887	FnrS-b1107	FnrS-b1479	FnrS-b1531	FnrS-b1656	FnrS-b1841	FnrS-b2153	FnrS-b2303	FnrS-b2531	FnrS-b3829	FnrS-b3908	GcvB-b1040	GcvB-b1130	GcvB-b3089	GcvB-b4208	MicA-b0411	MicA-b0814	MicA-b0957	MicA-b1130	MicC-b2215	MicF-b0241	MicF-b0889	MicF-b0929	MicF-b3912	OxyS-b1892	OxyS-b2731	RprA-b1040	RprA-b1341	RprA-b2741	RybB-b0081	RybB-b0721	RybB-b0805	RybB-b1256	RybB-b2215	RybB-b2594	RyhB-b0118	RyhB-b0156	RyhB-b0288	RyhB-b0592	RyhB-b0683	RyhB-b0721	RyhB-b0723	RyhB-b0894	RyhB-b1107	RyhB-b1200	RyhB-b1452	RyhB-b1531	RyhB-b1588	RyhB-b1612	RyhB-b1656	RyhB-b1778	RyhB-b1981	RyhB-b2069	RyhB-b2155	RyhB-b2206	RyhB-b2530	RyhB-b3365	RyhB-b3607	RyhB-b3942	RyhB-b4070	RyhB-b4122	SgrS-b1101	SgrS-b1817	SgrS-b2416	Spot42-b0039	Spot42-b0720	Spot42-b0721	Spot42-b0728	Spot42-b0757	Spot42-b1136	Spot42-b1302	Spot42-b1398	Spot42-b1761	Spot42-b1901	Spot42-b2221	Spot42-b2702	Spot42-b2715	Spot42-b2801	Spot42-b2802	Spot42-b3224	Spot42-b3566	Spot42-b3927	Spot42-b3962	Spot42-b4311
Target found among top-100 predictions for the sRNA for any tested parameterization	86	0	1	0	1	1	1	1	1	0	1	1	1	1	1	1	0	1	0	1	1	1	1	1	1	1	1	1	1	0	1	1	1	1	1	1	1	1	1	1	1	1	1	1	0	1	1	1	1	1	1	1	1	1	0	1	1	1	0	1	0	1	1	1	0	0	1	1	1	1	1	0	1	1	1	1	1	0	1	1	1	1	1	0	1	1	1	1	1	1	1	1	1	1	1	1	1	0	1	1	1	1	1
energyVRNAandronescu2007	41	0	0	0	1	1	1	0	1	0	0	1	0	0	1	1	0	0	0	1	0	0	0	0	1	0	0	1	0	0	0	0	0	1	0	0	1	1	1	1	1	1	1	0	0	1	0	1	0	0	0	0	1	0	0	1	0	0	0	1	0	1	1	0	0	0	0	0	1	0	0	0	0	0	0	1	1	0	1	1	1	0	0	0	0	0	0	1	1	1	1	1	0	0	0	1	1	0	1	0	1	0	1
energyVRNAlangdon2018	43	0	0	0	1	1	1	0	1	0	1	0	0	0	1	1	0	0	0	0	0	1	0	0	1	0	0	1	0	0	0	0	0	1	0	0	1	0	1	1	1	1	0	0	0	1	0	0	1	0	0	0	1	0	0	0	0	0	0	1	0	1	1	0	0	0	0	0	0	1	0	0	1	0	1	1	1	0	0	0	1	0	0	0	0	1	1	1	1	1	1	1	1	0	1	1	1	0	1	1	1	0	1
energyVRNAturner1999	37	0	0	0	1	1	1	0	1	0	1	1	0	0	1	1	0	1	0	0	0	0	1	0	1	0	0	1	0	0	0	0	0	0	0	0	1	1	1	0	1	1	0	0	0	1	0	1	1	0	0	0	0	0	0	0	0	0	0	0	0	1	1	0	0	0	0	0	0	0	0	0	0	0	0	1	1	0	0	0	1	0	0	0	0	1	0	1	1	1	1	1	1	0	0	0	1	0	1	1	1	0	1
energyVRNAturner2004	37	0	0	0	1	1	1	0	1	0	1	1	0	0	1	1	0	1	0	0	0	0	1	1	1	0	0	1	1	0	0	0	0	0	0	0	1	1	1	0	1	1	0	0	0	1	0	1	1	0	0	0	0	0	0	0	0	0	0	0	0	1	1	0	0	0	0	0	0	0	0	0	0	0	0	1	1	0	0	0	1	0	0	0	0	1	0	1	1	1	1	1	0	0	0	0	1	0	1	0	1	0	1
final.and.seedBP7	41	0	0	0	1	1	1	0	1	0	0	1	0	0	1	1	0	0	0	1	0	0	0	0	1	0	0	1	0	0	0	0	0	1	0	0	1	1	1	1	1	1	1	0	0	1	0	1	0	0	0	0	1	0	0	1	0	0	0	1	0	1	1	0	0	0	0	0	1	0	0	0	0	0	0	1	1	0	1	1	1	0	0	0	0	0	0	1	1	1	1	1	0	0	0	1	1	0	1	0	1	0	1
final.and.seedBP7.seedNoGU	40	0	0	0	1	1	1	0	1	0	0	1	0	0	1	1	0	0	0	1	0	0	0	0	1	0	0	1	0	0	0	0	0	1	0	0	1	1	1	1	0	1	1	0	0	1	0	1	0	0	0	0	1	0	0	1	0	0	0	1	0	1	0	0	0	0	0	0	1	0	0	0	1	0	0	1	1	0	1	1	1	0	0	0	0	1	0	1	1	1	1	1	0	0	0	1	1	0	1	0	1	0	0
final.and.seedBP7.seedNoGU.seedMinPu0.001	41	0	0	0	1	1	1	0	1	0	0	1	0	0	1	1	0	0	0	1	0	0	0	0	1	0	0	1	0	0	0	0	0	1	0	0	1	1	1	1	0	1	1	0	0	1	0	1	0	0	0	0	1	0	0	1	0	0	0	1	0	1	0	0	0	0	0	0	1	0	0	0	1	0	0	1	1	0	1	1	1	0	0	0	0	1	1	1	1	1	1	1	0	0	0	1	1	0	1	0	1	0	0
final.and.seedBP7.seedNoGU.seedMinPu0.001.intLenMax60	41	0	0	0	1	1	1	0	1	0	0	1	0	0	1	1	0	0	0	1	0	0	0	0	1	0	0	1	0	0	0	0	0	1	0	0	1	1	1	1	0	1	1	0	0	1	0	1	0	0	0	0	1	0	0	0	0	0	0	1	0	1	0	0	0	0	0	0	1	0	0	0	1	0	1	1	1	0	1	1	1	0	0	0	0	1	1	1	1	1	1	1	0	0	0	1	1	0	1	0	1	0	0
final.and.seedBP7.seedNoGU.seedMinPu0.001.intLenMax60.intLoopMax2	43	0	0	0	1	1	1	0	0	0	0	1	0	0	1	1	0	0	0	1	0	0	0	0	1	0	0	1	0	0	0	1	0	1	0	0	1	1	1	1	0	1	1	0	0	1	0	0	0	0	0	1	1	0	0	0	0	0	0	1	0	1	0	0	0	0	1	0	1	0	0	0	1	1	1	1	1	0	1	0	1	0	0	0	0	1	1	1	1	1	1	1	0	1	0	1	1	0	1	0	1	0	0
final.and.seedBP7.seedNoGU.seedMinPu0.001.intLenMax60.intLoopMax2.outMinPu0.001	43	0	0	0	1	1	1	0	0	0	0	1	0	0	1	1	0	0	0	1	0	0	0	0	1	0	0	1	0	0	0	1	0	1	0	0	1	1	1	1	0	1	1	0	0	1	0	0	0	0	0	1	1	0	0	0	0	0	0	1	0	1	0	0	0	0	1	0	1	0	0	0	1	1	1	1	1	0	1	0	1	0	0	0	0	1	1	1	1	1	1	1	0	1	0	1	1	0	1	0	1	0	0
final.and.seedBP7.seedNoGU.seedMinPu0.001.intLenMax60.intLoopMax5	40	0	0	0	1	1	1	0	0	0	0	1	0	0	1	1	0	0	0	1	0	0	0	0	1	0	0	1	0	0	0	0	0	1	0	0	1	1	1	1	0	1	1	0	0	1	0	1	0	0	0	0	1	0	0	0	0	0	0	1	0	1	0	0	0	0	0	0	1	0	0	0	1	0	1	1	1	0	1	1	1	0	0	0	0	1	1	1	1	1	1	1	0	0	0	1	1	0	1	0	1	0	0
final.and.seedBP7.seedNoGU.seedMinPu0.001.intLenMax60.intLoopMax5.outMinPu0.001	40	0	0	0	1	1	1	0	0	0	0	1	0	0	1	1	0	0	0	1	0	0	0	0	1	0	0	1	0	0	0	0	0	1	0	0	1	1	1	1	0	1	1	0	0	1	0	1	0	0	0	0	1	0	0	0	0	0	0	1	0	1	0	0	0	0	0	0	1	0	0	0	1	0	1	1	1	0	1	1	1	0	0	0	0	1	1	1	1	1	1	1	0	0	0	1	1	0	1	0	1	0	0
final.and.seedBP7.seedNoGU.seedMinPu0.001.intLenMax60.intLoopMax7	40	0	0	0	1	1	1	0	0	0	0	1	0	0	1	1	0	0	0	1	0	0	0	0	1	0	0	1	0	0	0	0	0	1	0	0	1	1	1	1	0	1	1	0	0	1	0	1	0	0	0	0	1	0	0	0	0	0	0	1	0	1	0	0	0	0	0	0	1	0	0	0	1	0	1	1	1	0	1	1	1	0	0	0	0	1	1	1	1	1	1	1	0	0	0	1	1	0	1	0	1	0	0
final.and.seedBP7.seedNoGU.seedMinPu0.001.intLenMax60.intLoopMax7.outMinPu0.001	40	0	0	0	1	1	1	0	0	0	0	1	0	0	1	1	0	0	0	1	0	0	0	0	1	0	0	1	0	0	0	0	0	1	0	0	1	1	1	1	0	1	1	0	0	1	0	1	0	0	0	0	1	0	0	0	0	0	0	1	0	1	0	0	0	0	0	0	1	0	0	0	1	0	1	1	1	0	1	1	1	0	0	0	0	1	1	1	1	1	1	1	0	0	0	1	1	0	1	0	1	0	0
final.and.seedBP7.seedNoGU.seedMinPu0.001.intLenMax60.intLoopMax8	40	0	0	0	1	1	1	0	0	0	0	1	0	0	1	1	0	0	0	1	0	0	0	0	1	0	0	1	0	0	0	0	0	1	0	0	1	1	1	1	0	1	1	0	0	1	0	1	0	0	0	0	1	0	0	0	0	0	0	1	0	1	0	0	0	0	0	0	1	0	0	0	1	0	1	1	1	0	1	1	1	0	0	0	0	1	1	1	1	1	1	1	0	0	0	1	1	0	1	0	1	0	0
final.and.seedBP7.seedNoGU.seedMinPu0.001.intLenMax60.intLoopMax8.outMinPu0.001	40	0	0	0	1	1	1	0	0	0	0	1	0	0	1	1	0	0	0	1	0	0	0	0	1	0	0	1	0	0	0	0	0	1	0	0	1	1	1	1	0	1	1	0	0	1	0	1	0	0	0	0	1	0	0	0	0	0	0	1	0	1	0	0	0	0	0	0	1	0	0	0	1	0	1	1	1	0	1	1	1	0	0	0	0	1	1	1	1	1	1	1	0	0	0	1	1	0	1	0	1	0	0
final.and.seedBP7.seedNoGU.seedMinPu0.001.intLenMax60.intLoopMax10	40	0	0	0	1	1	1	0	0	0	0	1	0	0	1	1	0	0	0	1	0	0	0	0	1	0	0	1	0	0	0	0	0	1	0	0	1	1	1	1	0	1	1	0	0	1	0	1	0	0	0	0	1	0	0	0	0	0	0	1	0	1	0	0	0	0	0	0	1	0	0	0	1	0	1	1	1	0	1	1	1	0	0	0	0	1	1	1	1	1	1	1	0	0	0	1	1	0	1	0	1	0	0
final.and.seedBP7.seedNoGU.seedMinPu0.001.intLenMax60.intLoopMax10.outMinPu0.001	40	0	0	0	1	1	1	0	0	0	0	1	0	0	1	1	0	0	0	1	0	0	0	0	1	0	0	1	0	0	0	0	0	1	0	0	1	1	1	1	0	1	1	0	0	1	0	1	0	0	0	0	1	0	0	0	0	0	0	1	0	1	0	0	0	0	0	0	1	0	0	0	1	0	1	1	1	0	1	1	1	0	0	0	0	1	1	1	1	1	1	1	0	0	0	1	1	0	1	0	1	0	0
final.seedBP7	37	0	0	0	1	1	1	0	1	0	1	1	0	0	1	1	0	1	0	0	0	0	1	1	1	0	0	1	1	0	0	0	0	0	0	0	1	1	1	0	1	1	0	0	0	1	0	1	1	0	0	0	0	0	0	0	0	0	0	0	0	1	1	0	0	0	0	0	0	0	0	0	0	0	0	1	1	0	0	0	1	0	0	0	0	1	0	1	1	1	1	1	0	0	0	0	1	0	1	0	1	0	1
final.seedBP7.seedNoGU	45	0	0	0	1	1	1	0	1	0	1	1	0	0	1	1	0	0	0	0	0	0	1	0	1	0	0	1	1	0	0	0	0	1	0	1	1	1	1	1	0	1	0	0	0	1	0	1	0	0	1	0	1	0	0	1	0	1	0	1	0	1	0	0	0	0	1	0	0	0	0	0	1	0	0	1	1	0	0	0	1	0	1	0	0	1	1	1	1	1	1	1	0	1	0	0	1	0	1	1	1	0	0
final.seedBP7.seedNoGU.seedMinPu0.001	49	0	0	0	1	1	1	0	1	0	1	1	0	0	1	1	0	0	0	0	0	0	1	0	1	0	0	1	1	0	0	0	0	1	0	1	1	1	1	1	0	1	0	0	0	1	0	1	0	0	1	0	1	0	0	1	0	1	0	1	0	1	0	0	0	0	1	0	0	0	0	0	1	1	1	1	1	0	0	1	1	0	1	0	0	0	1	1	1	1	1	1	1	1	0	1	1	0	1	1	1	0	0
final.seedBP7.seedNoGU.seedMinPu0.001.intLenMax60	48	0	0	0	1	1	1	0	1	0	1	1	0	0	1	1	0	0	0	0	0	0	1	0	1	0	0	1	1	0	0	0	0	1	0	1	1	1	1	1	0	1	0	0	0	1	0	1	0	0	1	0	1	0	0	0	0	1	0	1	0	1	0	0	0	0	1	0	0	0	0	0	1	1	1	1	1	0	0	1	1	0	1	0	0	0	1	1	1	1	1	1	1	1	0	1	1	0	1	1	1	0	0
final.seedBP7.seedNoGU.seedMinPu0.001.intLenMax60.intLoopMax2	46	0	1	0	1	1	1	0	0	0	0	1	0	0	1	1	0	0	0	1	0	1	1	0	1	0	0	1	1	0	0	0	0	0	0	1	1	1	1	1	0	1	1	0	0	1	0	0	0	0	1	1	0	0	0	0	0	0	0	1	0	1	0	0	0	0	1	0	0	1	0	0	1	1	0	1	1	0	0	1	1	0	0	0	1	0	1	1	1	1	1	1	1	1	0	1	1	0	1	0	1	0	0
final.seedBP7.seedNoGU.seedMinPu0.001.intLenMax60.intLoopMax2.outMinPu0.001	45	0	1	0	1	1	1	0	0	0	0	1	0	0	1	1	0	0	0	1	0	1	1	0	1	0	0	1	0	0	0	0	0	0	0	1	1	1	1	1	0	1	1	0	0	1	0	0	0	0	1	1	0	0	0	0	0	0	0	1	0	1	0	0	0	0	1	0	0	1	0	0	1	1	0	1	1	0	0	1	1	0	0	0	1	0	1	1	1	1	1	1	1	1	0	1	1	0	1	0	1	0	0
final.seedBP7.seedNoGU.seedMinPu0.001.intLenMax60.intLoopMax5	48	0	0	0	1	1	1	0	0	0	1	1	0	0	1	1	0	0	0	1	0	0	1	0	1	0	0	1	1	0	0	0	0	0	0	1	1	1	1	0	0	1	1	0	0	1	0	1	0	0	1	1	1	0	0	0	0	0	0	1	0	1	0	0	0	0	1	0	0	1	0	0	1	1	1	1	1	0	0	1	1	0	1	0	0	0	1	1	1	1	1	1	1	1	0	1	1	0	1	1	1	0	0
final.seedBP7.seedNoGU.seedMinPu0.001.intLenMax60.intLoopMax5.outMinPu0.001	49	0	0	0	1	1	1	0	0	0	1	1	0	0	1	1	0	0	0	1	0	1	1	0	1	0	0	1	0	0	0	0	0	0	0	1	1	1	1	1	0	1	1	0	0	1	0	1	0	0	1	1	1	0	0	0	0	0	0	1	0	1	0	0	0	0	1	0	0	1	0	0	1	1	1	1	1	0	0	1	1	0	1	0	0	0	1	1	1	1	1	1	1	1	0	1	1	0	1	1	1	0	0
final.seedBP7.seedNoGU.seedMinPu0.001.intLenMax60.intLoopMax7	48	0	0	0	1	1	1	0	0	0	1	1	0	0	1	1	0	0	0	0	0	0	1	0	1	0	0	1	1	0	0	0	0	1	0	1	1	1	1	1	0	1	0	0	0	1	0	1	0	0	1	0	1	0	0	0	0	1	0	1	0	1	0	0	0	0	1	0	0	1	0	0	1	1	1	1	1	0	0	1	1	0	1	0	0	0	1	1	1	1	1	1	1	1	0	1	1	0	1	1	1	0	0
final.seedBP7.seedNoGU.seedMinPu0.001.intLenMax60.intLoopMax7.outMinPu0.001	50	0	0	0	1	1	1	0	0	0	1	1	0	0	1	1	0	0	0	1	0	1	1	0	1	0	0	1	0	0	0	0	0	1	0	1	1	1	1	1	0	1	1	0	0	1	0	1	0	0	1	0	1	0	0	0	0	1	0	1	0	1	0	0	0	0	1	0	0	1	0	0	1	1	1	1	1	0	0	1	1	0	1	0	0	0	1	1	1	1	1	1	1	1	0	1	1	0	1	1	1	0	0
final.seedBP7.seedNoGU.seedMinPu0.001.intLenMax60.intLoopMax8	48	0	0	0	1	1	1	0	0	0	1	1	0	0	1	1	0	0	0	0	0	0	1	0	1	0	0	1	1	0	0	0	0	1	0	1	1	1	1	1	0	1	0	0	0	1	0	1	0	0	1	0	1	0	0	0	0	1	0	1	0	1	0	0	0	0	1	0	0	1	0	0	1	1	1	1	1	0	0	1	1	0	1	0	0	0	1	1	1	1	1	1	1	1	0	1	1	0	1	1	1	0	0
final.seedBP7.seedNoGU.seedMinPu0.001.intLenMax60.intLoopMax8.outMinPu0.001	50	0	0	0	1	1	1	0	0	0	1	1	0	0	1	1	0	0	0	1	0	1	1	0	1	0	0	1	0	0	0	0	0	1	0	1	1	1	1	1	0	1	1	0	0	1	0	1	0	0	1	0	1	0	0	0	0	1	0	1	0	1	0	0	0	0	1	0	0	1	0	0	1	1	1	1	1	0	0	1	1	0	1	0	0	0	1	1	1	1	1	1	1	1	0	1	1	0	1	1	1	0	0
final.seedBP7.seedNoGU.seedMinPu0.001.intLenMax60.intLoopMax10	47	0	0	0	1	1	1	0	0	0	1	1	0	0	1	1	0	0	0	0	0	0	1	0	1	0	0	1	1	0	0	0	0	1	0	1	1	1	1	1	0	1	0	0	0	1	0	1	0	0	1	0	1	0	0	0	0	1	0	1	0	1	0	0	0	0	1	0	0	0	0	0	1	1	1	1	1	0	0	1	1	0	1	0	0	0	1	1	1	1	1	1	1	1	0	1	1	0	1	1	1	0	0
final.seedBP7.seedNoGU.seedMinPu0.001.intLenMax60.intLoopMax10.outMinPu0.001	50	0	0	0	1	1	1	0	0	0	1	1	0	0	1	1	0	0	0	1	0	1	1	0	1	0	0	1	0	0	0	0	0	1	0	1	1	1	1	1	0	1	1	0	0	1	0	1	0	0	1	0	1	0	0	0	0	1	0	1	0	1	0	0	0	0	1	0	0	1	0	0	1	1	1	1	1	0	0	1	1	0	1	0	0	0	1	1	1	1	1	1	1	1	0	1	1	0	1	1	1	0	0
intLenMax10	32	0	1	0	1	1	1	0	0	0	0	0	1	1	1	0	0	0	0	1	0	0	0	1	0	1	0	1	0	0	0	1	0	1	0	0	0	1	1	0	1	0	0	0	0	0	0	0	0	0	1	1	1	0	0	0	0	0	0	0	0	0	0	0	0	0	0	0	0	1	1	0	0	1	0	1	0	0	0	0	0	0	0	0	0	1	0	0	0	1	0	1	0	1	0	0	1	1	0	1	0	1	0	1
intLenMax15	34	0	0	0	1	1	1	0	0	0	0	0	0	0	1	0	0	0	0	1	0	0	0	0	0	0	0	1	0	0	0	1	0	0	0	1	1	1	1	1	1	1	1	0	0	1	0	0	0	0	0	0	1	0	0	0	0	0	0	0	0	0	1	0	0	0	0	0	0	1	1	0	0	1	0	0	1	0	0	1	0	0	0	0	0	1	1	0	1	0	1	0	1	1	0	1	1	0	1	0	1	0	1
intLenMax20	37	0	0	0	1	1	1	0	0	0	0	1	0	0	1	1	0	0	0	1	0	0	0	0	1	0	0	1	0	0	0	0	0	0	0	0	1	1	1	0	1	1	0	0	0	1	0	0	0	0	0	1	0	0	0	0	0	0	0	1	0	1	1	0	0	0	1	0	0	1	0	0	1	0	0	1	1	0	0	1	1	0	0	0	0	1	0	0	1	1	1	1	1	1	0	0	1	0	1	0	1	0	1
intLenMax25	37	0	0	0	1	1	1	0	0	0	0	1	0	0	1	1	0	1	0	0	0	0	1	0	1	0	0	1	0	0	0	0	0	0	0	0	1	1	1	0	1	1	0	0	0	1	0	0	0	0	0	0	0	0	0	1	0	0	0	1	0	1	1	0	0	0	1	0	0	0	0	0	1	0	0	1	1	0	0	1	1	0	0	0	0	1	0	0	1	1	1	1	1	1	0	0	1	0	1	0	1	0	1
intLenMax30	34	0	0	0	1	1	1	0	0	0	0	1	0	0	1	1	0	1	0	0	0	0	1	0	0	0	0	1	0	0	0	0	0	0	0	0	1	1	1	0	1	1	0	0	0	1	0	0	0	0	0	0	1	0	0	1	0	0	0	1	0	1	1	0	0	0	1	0	0	0	0	0	1	0	0	1	1	0	0	0	1	0	0	0	0	1	0	0	1	0	1	1	0	1	0	0	1	0	1	0	1	0	1
intLenMax40	36	0	0	0	1	1	1	0	0	0	1	1	0	0	1	1	0	1	0	0	0	0	1	0	1	0	0	1	0	0	0	0	0	1	0	0	1	1	1	0	1	1	0	0	0	1	0	1	1	0	0	0	0	0	0	1	0	0	0	0	0	1	1	0	0	0	0	0	0	0	0	0	1	0	0	1	1	0	0	0	1	0	0	0	0	1	0	0	1	0	1	1	0	1	0	0	1	0	1	0	1	0	1
intLenMax50	36	0	0	0	1	1	1	0	1	0	1	1	0	0	1	1	0	1	0	0	0	0	1	0	1	0	0	1	1	0	0	0	0	0	0	0	1	1	1	0	1	1	0	0	0	1	0	1	1	0	0	0	0	0	0	0	0	0	0	0	0	1	1	0	0	0	0	0	0	0	0	0	0	0	0	1	1	0	0	0	1	0	0	0	0	1	0	1	1	1	1	1	0	0	0	0	1	0	1	0	1	0	1
intLenMax60	37	0	0	0	1	1	1	0	1	0	1	1	0	0	1	1	0	1	0	0	0	0	1	0	1	0	0	1	1	0	0	0	0	0	0	0	1	1	1	0	1	1	0	0	0	1	0	1	1	0	0	0	0	0	0	0	0	0	0	0	0	1	1	0	0	0	0	0	0	0	0	0	0	0	0	1	1	0	0	0	1	0	1	0	0	1	0	1	1	1	1	1	0	0	0	0	1	0	1	0	1	0	1
intLenMax70	37	0	0	0	1	1	1	0	1	0	1	1	0	0	1	1	0	1	0	0	0	0	1	0	1	0	0	1	1	0	0	0	0	0	0	0	1	1	1	0	1	1	0	0	0	1	0	1	1	0	0	0	0	0	0	0	0	0	0	0	0	1	1	0	0	0	0	0	0	0	0	0	0	0	0	1	1	0	0	0	1	0	1	0	0	1	0	1	1	1	1	1	0	0	0	0	1	0	1	0	1	0	1
intLenMax75	37	0	0	0	1	1	1	0	1	0	1	1	0	0	1	1	0	1	0	0	0	0	1	0	1	0	0	1	1	0	0	0	0	0	0	0	1	1	1	0	1	1	0	0	0	1	0	1	1	0	0	0	0	0	0	0	0	0	0	0	0	1	1	0	0	0	0	0	0	0	0	0	0	0	0	1	1	0	0	0	1	0	1	0	0	1	0	1	1	1	1	1	0	0	0	0	1	0	1	0	1	0	1
intLenMax80	37	0	0	0	1	1	1	0	1	0	1	1	0	0	1	1	0	1	0	0	0	0	1	0	1	0	0	1	1	0	0	0	0	0	0	0	1	1	1	0	1	1	0	0	0	1	0	1	1	0	0	0	0	0	0	0	0	0	0	0	0	1	1	0	0	0	0	0	0	0	0	0	0	0	0	1	1	0	0	0	1	0	1	0	0	1	0	1	1	1	1	1	0	0	0	0	1	0	1	0	1	0	1
intLenMax100	37	0	0	0	1	1	1	0	1	0	1	1	0	0	1	1	0	1	0	0	0	0	1	1	1	0	0	1	1	0	0	0	0	0	0	0	1	1	1	0	1	1	0	0	0	1	0	1	1	0	0	0	0	0	0	0	0	0	0	0	0	1	1	0	0	0	0	0	0	0	0	0	0	0	0	1	1	0	0	0	1	0	0	0	0	1	0	1	1	1	1	1	0	0	0	0	1	0	1	0	1	0	1
intLenMax150	37	0	0	0	1	1	1	0	1	0	1	1	0	0	1	1	0	1	0	0	0	0	1	1	1	0	0	1	1	0	0	0	0	0	0	0	1	1	1	0	1	1	0	0	0	1	0	1	1	0	0	0	0	0	0	0	0	0	0	0	0	1	1	0	0	0	0	0	0	0	0	0	0	0	0	1	1	0	0	0	1	0	0	0	0	1	0	1	1	1	1	1	0	0	0	0	1	0	1	0	1	0	1
intLoopMax2	38	0	0	0	1	1	1	0	0	0	0	1	0	0	1	1	0	1	0	0	0	0	1	0	1	0	0	1	1	0	0	0	0	0	0	0	1	1	1	0	1	1	0	0	0	1	0	0	1	0	0	0	0	0	0	0	0	0	0	1	0	1	1	0	0	0	1	0	0	0	0	0	1	0	0	1	1	0	0	0	1	0	0	0	0	1	0	1	1	1	1	1	1	1	0	0	1	0	1	0	1	0	1
intLoopMax3	37	0	0	0	1	1	1	0	0	0	1	1	0	0	1	1	0	1	0	0	0	0	1	0	1	0	0	1	1	0	0	0	0	0	0	0	1	1	1	0	1	1	0	0	0	1	0	0	1	0	0	0	0	0	0	0	0	0	0	1	0	1	1	0	0	0	0	0	0	0	0	0	1	0	0	1	1	0	0	0	1	0	0	0	0	1	0	1	1	1	1	1	0	1	0	0	1	0	1	0	1	0	1
intLoopMax4	34	0	0	0	1	1	1	0	0	0	1	1	0	0	1	1	0	1	0	0	0	0	1	0	1	0	0	1	1	0	0	0	0	0	0	0	1	1	1	0	1	1	0	0	0	1	0	0	1	0	0	0	0	0	0	0	0	0	0	0	0	1	1	0	0	0	0	0	0	0	0	0	0	0	0	1	1	0	0	0	1	0	0	0	0	1	0	1	1	1	1	1	0	0	0	0	1	0	1	0	1	0	1
intLoopMax5	35	0	0	0	1	1	1	0	0	0	1	1	0	0	1	1	0	1	0	0	0	0	1	0	1	0	0	1	1	0	0	0	0	0	0	0	1	1	1	0	1	1	0	0	0	1	0	1	1	0	0	0	0	0	0	0	0	0	0	0	0	1	1	0	0	0	0	0	0	0	0	0	0	0	0	1	1	0	0	0	1	0	0	0	0	1	0	1	1	1	1	1	0	0	0	0	1	0	1	0	1	0	1
intLoopMax6	35	0	0	0	1	1	1	0	0	0	1	1	0	0	1	1	0	1	0	0	0	0	1	0	1	0	0	1	1	0	0	0	0	0	0	0	1	1	1	0	1	1	0	0	0	1	0	1	1	0	0	0	0	0	0	0	0	0	0	0	0	1	1	0	0	0	0	0	0	0	0	0	0	0	0	1	1	0	0	0	1	0	0	0	0	1	0	1	1	1	1	1	0	0	0	0	1	0	1	0	1	0	1
intLoopMax7	35	0	0	0	1	1	1	0	0	0	1	1	0	0	1	1	0	1	0	0	0	0	1	0	1	0	0	1	1	0	0	0	0	0	0	0	1	1	1	0	1	1	0	0	0	1	0	1	1	0	0	0	0	0	0	0	0	0	0	0	0	1	1	0	0	0	0	0	0	0	0	0	0	0	0	1	1	0	0	0	1	0	0	0	0	1	0	1	1	1	1	1	0	0	0	0	1	0	1	0	1	0	1
intLoopMax8	36	0	0	0	1	1	1	0	0	0	1	1	0	0	1	1	0	1	0	0	0	0	1	0	1	0	0	1	1	0	0	0	0	0	0	0	1	1	1	0	1	1	0	0	0	1	0	1	1	0	0	0	0	0	0	0	0	0	0	0	0	1	1	0	0	0	0	0	0	0	0	0	0	0	0	1	1	0	0	0	1	0	1	0	0	1	0	1	1	1	1	1	0	0	0	0	1	0	1	0	1	0	1
intLoopMax9	36	0	0	0	1	1	1	0	0	0	1	1	0	0	1	1	0	1	0	0	0	0	1	0	1	0	0	1	1	0	0	0	0	0	0	0	1	1	1	0	1	1	0	0	0	1	0	1	1	0	0	0	0	0	0	0	0	0	0	0	0	1	1	0	0	0	0	0	0	0	0	0	0	0	0	1	1	0	0	0	1	0	1	0	0	1	0	1	1	1	1	1	0	0	0	0	1	0	1	0	1	0	1
intLoopMax10	36	0	0	0	1	1	1	0	0	0	1	1	0	0	1	1	0	1	0	0	0	0	1	0	1	0	0	1	1	0	0	0	0	0	0	0	1	1	1	0	1	1	0	0	0	1	0	1	1	0	0	0	0	0	0	0	0	0	0	0	0	1	1	0	0	0	0	0	0	0	0	0	0	0	0	1	1	0	0	0	1	0	1	0	0	1	0	1	1	1	1	1	0	0	0	0	1	0	1	0	1	0	1
intLoopMax11	36	0	0	0	1	1	1	0	0	0	1	1	0	0	1	1	0	1	0	0	0	0	1	0	1	0	0	1	1	0	0	0	0	0	0	0	1	1	1	0	1	1	0	0	0	1	0	1	1	0	0	0	0	0	0	0	0	0	0	0	0	1	1	0	0	0	0	0	0	0	0	0	0	0	0	1	1	0	0	0	1	0	1	0	0	1	0	1	1	1	1	1	0	0	0	0	1	0	1	0	1	0	1
intLoopMax12	37	0	0	0	1	1	1	0	1	0	1	1	0	0	1	1	0	1	0	0	0	0	1	0	1	0	0	1	1	0	0	0	0	0	0	0	1	1	1	0	1	1	0	0	0	1	0	1	1	0	0	0	0	0	0	0	0	0	0	0	0	1	1	0	0	0	0	0	0	0	0	0	0	0	0	1	1	0	0	0	1	0	1	0	0	1	0	1	1	1	1	1	0	0	0	0	1	0	1	0	1	0	1
intLoopMax14	37	0	0	0	1	1	1	0	1	0	1	1	0	0	1	1	0	1	0	0	0	0	1	1	1	0	0	1	1	0	0	0	0	0	0	0	1	1	1	0	1	1	0	0	0	1	0	1	1	0	0	0	0	0	0	0	0	0	0	0	0	1	1	0	0	0	0	0	0	0	0	0	0	0	0	1	1	0	0	0	1	0	0	0	0	1	0	1	1	1	1	1	0	0	0	0	1	0	1	0	1	0	1

Echericha coli NC_000913

	sum	ArcZ-b1892	ArcZ-b2741	ArcZ-b3546	ChiX-b0619	ChiX-b0681	ChiX-b1737	CyaR-b0723	CyaR-b0814	CyaR-b1740	CyaR-b1824	CyaR-b2416	CyaR-b2666	CyaR-b2687	DsrA-b1237	DsrA-b2741	DsrA-b3251	FnrS-b0723	FnrS-b0755	FnrS-b0887	FnrS-b1107	FnrS-b1479	FnrS-b1531	FnrS-b1656	FnrS-b1841	FnrS-b2153	FnrS-b2303	FnrS-b2531	FnrS-b3829	FnrS-b3908	GcvB-b1040	GcvB-b1130	GcvB-b3089	GcvB-b4208	MicA-b0411	MicA-b0814	MicA-b0957	MicA-b1130	MicC-b2215	MicF-b0241	MicF-b0889	MicF-b0929	MicF-b3912	OxyS-b1892	OxyS-b2731	RprA-b1040	RprA-b1341	RprA-b2741	RybB-b0081	RybB-b0721	RybB-b0805	RybB-b1256	RybB-b2215	RybB-b2594	RyhB-b0118	RyhB-b0156	RyhB-b0288	RyhB-b0592	RyhB-b0683	RyhB-b0721	RyhB-b0723	RyhB-b0894	RyhB-b1107	RyhB-b1200	RyhB-b1452	RyhB-b1531	RyhB-b1588	RyhB-b1612	RyhB-b1656	RyhB-b1778	RyhB-b1981	RyhB-b2069	RyhB-b2155	RyhB-b2206	RyhB-b2530	RyhB-b3365	RyhB-b3607	RyhB-b3942	RyhB-b4070	RyhB-b4122	SgrS-b1101	SgrS-b1817	SgrS-b2416	Spot42-b0039	Spot42-b0720	Spot42-b0721	Spot42-b0728	Spot42-b0757	Spot42-b1136	Spot42-b1302	Spot42-b1398	Spot42-b1761	Spot42-b1901	Spot42-b2221	Spot42-b2702	Spot42-b2715	Spot42-b2801	Spot42-b2802	Spot42-b3224	Spot42-b3566	Spot42-b3927	Spot42-b3962	Spot42-b4311
Target found among top-100 predictions for the sRNA for any tested parameterization	86	0	1	0	1	1	1	1	1	0	1	1	1	1	1	1	0	1	0	1	1	1	1	1	1	1	1	1	1	0	1	1	1	1	1	1	1	1	1	1	1	1	1	1	0	1	1	1	1	1	1	1	1	1	0	1	1	1	0	1	0	1	1	1	0	0	1	1	1	1	1	0	1	1	1	1	1	0	1	1	1	1	1	0	1	1	1	1	1	1	1	1	1	1	1	1	1	0	1	1	1	1	1
intLoopMax16	36	0	0	0	1	1	1	0	1	0	1	1	0	0	1	1	0	1	0	0	0	0	1	0	1	0	0	1	1	0	0	0	0	0	0	0	1	1	1	0	1	1	0	0	0	1	0	1	1	0	0	0	0	0	0	0	0	0	0	0	0	1	1	0	0	0	0	0	0	0	0	0	0	0	0	1	1	0	0	0	1	0	0	0	0	1	0	1	1	1	1	1	0	0	0	0	1	0	1	0	1	0	1
noSeed	35	0	0	0	1	1	1	0	1	0	1	1	0	0	1	1	0	1	0	0	0	0	1	0	1	0	0	1	1	0	0	0	0	0	0	0	1	1	1	0	1	1	0	0	0	1	0	1	1	0	0	0	0	0	0	0	0	0	0	0	0	1	1	0	0	0	0	0	0	0	0	0	0	0	0	1	1	0	0	0	1	0	0	0	0	1	0	0	1	1	1	1	0	0	0	0	1	0	1	0	1	0	1
outMaxE0	36	0	0	0	1	1	1	0	1	0	1	1	0	0	1	1	0	1	0	0	0	0	1	0	1	0	0	1	1	0	0	0	0	0	0	0	1	1	1	0	1	1	0	0	0	1	0	1	1	0	0	0	0	0	0	0	0	0	0	0	0	1	1	0	0	0	0	0	0	0	0	0	0	0	0	1	1	0	0	0	1	0	0	0	0	1	0	1	1	1	1	1	0	0	0	0	1	0	1	0	1	0	1
outMaxE-1	36	0	0	0	1	1	1	0	1	0	1	1	0	0	1	1	0	1	0	0	0	0	1	0	1	0	0	1	1	0	0	0	0	0	0	0	1	1	1	0	1	1	0	0	0	1	0	1	1	0	0	0	0	0	0	0	0	0	0	0	0	1	1	0	0	0	0	0	0	0	0	0	0	0	0	1	1	0	0	0	1	0	0	0	0	1	0	1	1	1	1	1	0	0	0	0	1	0	1	0	1	0	1
outMaxE-2	36	0	0	0	1	1	1	0	1	0	1	1	0	0	1	1	0	1	0	0	0	0	1	0	1	0	0	1	1	0	0	0	0	0	0	0	1	1	1	0	1	1	0	0	0	1	0	1	1	0	0	0	0	0	0	0	0	0	0	0	0	1	1	0	0	0	0	0	0	0	0	0	0	0	0	1	1	0	0	0	1	0	0	0	0	1	0	1	1	1	1	1	0	0	0	0	1	0	1	0	1	0	1
outMaxE-3	36	0	0	0	1	1	1	0	1	0	1	1	0	0	1	1	0	1	0	0	0	0	1	0	1	0	0	1	1	0	0	0	0	0	0	0	1	1	1	0	1	1	0	0	0	1	0	1	1	0	0	0	0	0	0	0	0	0	0	0	0	1	1	0	0	0	0	0	0	0	0	0	0	0	0	1	1	0	0	0	1	0	0	0	0	1	0	1	1	1	1	1	0	0	0	0	1	0	1	0	1	0	1
outMaxE-4	36	0	0	0	1	1	1	0	1	0	1	1	0	0	1	1	0	1	0	0	0	0	1	0	1	0	0	1	1	0	0	0	0	0	0	0	1	1	1	0	1	1	0	0	0	1	0	1	1	0	0	0	0	0	0	0	0	0	0	0	0	1	1	0	0	0	0	0	0	0	0	0	0	0	0	1	1	0	0	0	1	0	0	0	0	1	0	1	1	1	1	1	0	0	0	0	1	0	1	0	1	0	1
outMaxE-5	36	0	0	0	1	1	1	0	1	0	1	1	0	0	1	1	0	1	0	0	0	0	1	0	1	0	0	1	1	0	0	0	0	0	0	0	1	1	1	0	1	1	0	0	0	1	0	1	1	0	0	0	0	0	0	0	0	0	0	0	0	1	1	0	0	0	0	0	0	0	0	0	0	0	0	1	1	0	0	0	1	0	0	0	0	1	0	1	1	1	1	1	0	0	0	0	1	0	1	0	1	0	1
outMaxE-6	36	0	0	0	1	1	1	0	1	0	1	1	0	0	1	1	0	1	0	0	0	0	1	0	1	0	0	1	1	0	0	0	0	0	0	0	1	1	1	0	1	1	0	0	0	1	0	1	1	0	0	0	0	0	0	0	0	0	0	0	0	1	1	0	0	0	0	0	0	0	0	0	0	0	0	1	1	0	0	0	1	0	0	0	0	1	0	1	1	1	1	1	0	0	0	0	1	0	1	0	1	0	1
outMaxE-7	36	0	0	0	1	1	1	0	1	0	1	1	0	0	1	1	0	1	0	0	0	0	1	0	1	0	0	1	1	0	0	0	0	0	0	0	1	1	1	0	1	1	0	0	0	1	0	1	1	0	0	0	0	0	0	0	0	0	0	0	0	1	1	0	0	0	0	0	0	0	0	0	0	0	0	1	1	0	0	0	1	0	0	0	0	1	0	1	1	1	1	1	0	0	0	0	1	0	1	0	1	0	1
outMaxE-8	36	0	0	0	1	1	1	0	1	0	1	1	0	0	1	1	0	1	0	0	0	0	1	0	1	0	0	1	1	0	0	0	0	0	0	0	1	1	1	0	1	1	0	0	0	1	0	1	1	0	0	0	0	0	0	0	0	0	0	0	0	1	1	0	0	0	0	0	0	0	0	0	0	0	0	1	1	0	0	0	1	0	0	0	0	1	0	1	1	1	1	1	0	0	0	0	1	0	1	0	1	0	1
outMaxE-9	36	0	0	0	1	1	1	0	1	0	1	1	0	0	1	1	0	1	0	0	0	0	1	0	1	0	0	1	1	0	0	0	0	0	0	0	1	1	1	0	1	1	0	0	0	1	0	1	1	0	0	0	0	0	0	0	0	0	0	0	0	1	1	0	0	0	0	0	0	0	0	0	0	0	0	1	1	0	0	0	1	0	0	0	0	1	0	1	1	1	1	1	0	0	0	0	1	0	1	0	1	0	1
outMaxE-10	36	0	0	0	1	1	1	0	1	0	1	1	0	0	1	1	0	1	0	0	0	0	1	0	1	0	0	1	1	0	0	0	0	0	0	0	1	1	1	0	1	1	0	0	0	1	0	1	1	0	0	0	0	0	0	0	0	0	0	0	0	1	1	0	0	0	0	0	0	0	0	0	0	0	0	1	1	0	0	0	1	0	0	0	0	1	0	1	1	1	1	1	0	0	0	0	1	0	1	0	1	0	1
outMaxE-11	36	0	0	0	1	1	1	0	1	0	1	1	0	0	1	1	0	1	0	0	0	0	1	0	1	0	0	1	1	0	0	0	0	0	0	0	1	1	1	0	1	1	0	0	0	1	0	1	1	0	0	0	0	0	0	0	0	0	0	0	0	1	1	0	0	0	0	0	0	0	0	0	0	0	0	1	1	0	0	0	1	0	0	0	0	1	0	1	1	1	1	1	0	0	0	0	1	0	1	0	1	0	1
outMaxE-12	34	0	0	0	1	1	1	0	1	0	1	1	0	0	1	1	0	1	0	0	0	0	1	0	1	0	0	1	1	0	0	0	0	0	0	0	1	1	1	0	1	1	0	0	0	1	0	1	1	0	0	0	0	0	0	0	0	0	0	0	0	1	1	0	0	0	0	0	0	0	0	0	0	0	0	1	1	0	0	0	1	0	0	0	0	1	0	1	0	1	1	1	0	0	0	0	1	0	1	0	0	0	1
outMaxE-14	25	0	0	0	1	1	1	0	1	0	1	1	0	0	0	1	0	1	0	0	0	0	0	0	1	0	0	1	0	0	0	0	0	0	0	0	0	0	1	0	1	1	0	0	0	1	0	1	1	0	0	0	0	0	0	0	0	0	0	0	0	1	1	0	0	0	0	0	0	0	0	0	0	0	0	1	1	0	0	0	1	0	0	0	0	0	0	1	0	1	1	0	0	0	0	0	0	0	1	0	0	0	0
outMaxE-16	16	0	0	0	0	1	1	0	0	0	0	0	0	0	0	1	0	0	0	0	0	0	0	0	1	0	0	1	0	0	0	0	0	0	0	0	0	0	1	0	1	1	0	0	0	1	0	1	1	0	0	0	0	0	0	0	0	0	0	0	0	1	1	0	0	0	0	0	0	0	0	0	0	0	0	0	0	0	0	0	1	0	0	0	0	0	0	0	0	1	0	0	0	0	0	0	0	0	1	0	0	0	0
outMaxE-18	7	0	0	0	0	0	1	0	0	0	0	0	0	0	0	0	0	0	0	0	0	0	0	0	1	0	0	1	0	0	0	0	0	0	0	0	0	0	1	0	1	1	0	0	0	1	0	0	0	0	0	0	0	0	0	0	0	0	0	0	0	0	0	0	0	0	0	0	0	0	0	0	0	0	0	0	0	0	0	0	0	0	0	0	0	0	0	0	0	0	0	0	0	0	0	0	0	0	0	0	0	0	0
outMaxE-20	3	0	0	0	0	0	0	0	0	0	0	0	0	0	0	0	0	0	0	0	0	0	0	0	0	0	0	1	0	0	0	0	0	0	0	0	0	0	1	0	1	0	0	0	0	0	0	0	0	0	0	0	0	0	0	0	0	0	0	0	0	0	0	0	0	0	0	0	0	0	0	0	0	0	0	0	0	0	0	0	0	0	0	0	0	0	0	0	0	0	0	0	0	0	0	0	0	0	0	0	0	0	0
outMinPu0	36	0	0	0	1	1	1	0	1	0	1	1	0	0	1	1	0	1	0	0	0	0	1	0	1	0	0	1	1	0	0	0	0	0	0	0	1	1	1	0	1	1	0	0	0	1	0	1	1	0	0	0	0	0	0	0	0	0	0	0	0	1	1	0	0	0	0	0	0	0	0	0	0	0	0	1	1	0	0	0	1	0	0	0	0	1	0	1	1	1	1	1	0	0	0	0	1	0	1	0	1	0	1
outMinPu0.1	37	0	1	0	1	1	1	0	0	0	0	1	0	0	1	1	0	0	0	1	1	0	1	0	1	0	0	1	0	0	0	1	0	1	0	1	1	1	0	0	1	1	0	0	0	1	0	1	0	0	0	0	0	0	0	1	0	0	0	0	0	1	1	0	0	0	0	0	0	1	0	0	1	0	0	1	1	0	0	0	1	0	0	0	0	0	1	0	1	1	0	0	1	0	0	0	1	0	0	0	1	1	1
outMinPu0.001	33	0	0	0	1	1	1	0	0	0	0	0	0	0	1	1	0	1	0	0	0	0	1	0	1	0	0	1	0	0	0	0	0	0	0	0	1	1	1	0	1	1	0	0	0	1	0	1	1	0	0	0	0	0	0	0	0	0	0	0	0	1	1	0	0	0	0	0	0	0	0	0	0	0	0	1	1	0	0	0	1	0	1	0	0	1	0	1	1	1	1	1	0	0	0	0	1	0	1	0	1	0	1
outMinPu0.0001	35	0	0	0	1	1	1	0	1	0	1	1	0	0	1	1	0	1	0	0	0	0	1	1	1	0	0	1	1	0	0	0	0	0	0	0	1	1	1	0	1	1	0	0	0	0	0	0	1	0	0	0	0	0	0	0	0	0	0	0	0	1	1	0	0	0	0	0	0	0	0	0	0	0	0	1	1	0	0	0	1	0	0	0	0	1	0	1	1	1	1	1	0	0	0	0	1	0	1	0	1	0	1
outMinPu0.01	42	0	0	0	1	1	1	0	0	0	1	1	0	0	1	1	0	1	0	0	0	0	1	0	1	0	0	1	0	0	0	0	0	1	0	0	1	1	1	0	1	1	0	0	0	1	0	1	1	0	0	0	1	0	0	1	0	0	0	1	0	1	1	0	0	0	1	0	0	0	0	0	1	0	0	1	1	0	0	0	0	0	0	0	0	0	1	1	1	1	1	1	1	1	0	1	1	0	1	0	1	0	1
outMinPu0.2	28	0	0	0	1	1	1	0	0	0	0	1	0	0	0	1	0	0	0	0	0	0	0	0	0	1	0	0	0	0	0	1	0	1	0	1	0	0	0	0	1	1	0	0	0	1	0	1	0	0	0	1	0	0	0	1	0	0	0	0	0	1	1	0	0	0	0	0	0	1	0	0	0	0	1	1	1	0	1	0	0	0	0	0	0	0	0	0	1	1	0	0	1	0	0	0	1	0	0	0	1	1	0
outMinPu0.5	8	0	1	0	0	0	1	0	0	0	0	0	0	0	0	0	0	0	0	0	0	0	0	0	1	0	0	0	0	0	1	0	0	0	0	0	0	0	1	0	0	0	0	0	0	0	0	0	0	0	0	0	0	0	0	0	0	0	0	0	0	0	0	0	0	0	0	0	0	0	0	0	0	0	0	0	0	0	0	0	0	0	0	0	0	0	0	1	0	0	0	0	1	0	0	0	0	0	0	0	1	0	0
seedBP4	36	0	0	0	1	1	1	0	1	0	1	1	0	0	1	1	0	1	0	0	0	0	1	0	1	0	0	1	1	0	0	0	0	0	0	0	1	1	1	0	1	1	0	0	0	1	0	1	1	0	0	0	0	0	0	0	0	0	0	0	0	1	1	0	0	0	0	0	0	0	0	0	0	0	0	1	1	0	0	0	1	0	1	0	0	1	0	0	1	1	1	1	0	0	0	0	1	0	1	0	1	0	1
seedBP4.seedMinPu0	36	0	0	0	1	1	1	0	1	0	1	1	0	0	1	1	0	1	0	0	0	0	1	0	1	0	0	1	1	0	0	0	0	0	0	0	1	1	1	0	1	1	0	0	0	1	0	1	1	0	0	0	0	0	0	0	0	0	0	0	0	1	1	0	0	0	0	0	0	0	0	0	0	0	0	1	1	0	0	0	1	0	1	0	0	1	0	0	1	1	1	1	0	0	0	0	1	0	1	0	1	0	1
seedBP4.seedMinPu0.001	36	0	0	0	1	1	1	0	1	0	1	1	0	0	1	1	0	1	0	0	0	0	1	0	1	0	0	1	1	0	0	0	0	0	0	0	1	1	1	0	1	1	0	0	0	1	0	1	1	0	0	0	0	0	0	0	0	0	0	0	0	1	1	0	0	0	0	0	0	0	0	0	0	0	0	1	1	0	0	0	1	0	1	0	0	1	0	0	1	1	1	1	0	0	0	0	1	0	1	0	1	0	1
seedBP4.seedMinPu0.0001	36	0	0	0	1	1	1	0	1	0	1	1	0	0	1	1	0	1	0	0	0	0	1	0	1	0	0	1	1	0	0	0	0	0	0	0	1	1	1	0	1	1	0	0	0	1	0	1	1	0	0	0	0	0	0	0	0	0	0	0	0	1	1	0	0	0	0	0	0	0	0	0	0	0	0	1	1	0	0	0	1	0	1	0	0	1	0	0	1	1	1	1	0	0	0	0	1	0	1	0	1	0	1
seedBP4.seedMinPu0.1	36	0	0	0	1	1	1	0	1	0	1	1	0	0	1	1	0	1	0	0	0	0	1	1	1	0	0	1	1	0	0	0	0	0	0	0	1	1	1	0	1	1	0	0	0	1	0	1	0	0	0	0	1	0	0	0	0	0	0	0	0	1	1	0	0	0	0	0	0	0	0	0	0	0	0	1	1	0	0	0	0	0	1	0	0	0	0	1	1	1	1	0	1	0	0	0	1	0	1	0	1	0	1
seedBP4.seedMinPu0.01	36	0	0	0	1	1	1	0	1	0	1	1	0	0	1	1	0	1	0	0	0	0	1	0	1	0	0	1	1	0	0	0	0	0	0	0	1	1	1	0	1	1	0	0	0	1	0	1	1	0	0	0	0	0	0	0	0	0	0	0	0	1	1	0	0	0	0	0	0	0	0	0	0	0	0	1	1	0	0	0	1	0	1	0	0	1	0	0	1	1	1	1	0	0	0	0	1	0	1	0	1	0	1
seedBP4.seedMinPu0.1.seedNoGU	40	0	0	0	1	1	1	0	1	0	1	1	0	0	1	1	0	1	0	0	0	0	1	1	1	0	0	1	1	0	0	0	0	0	0	0	1	1	1	0	1	1	0	0	0	1	0	1	0	0	0	0	1	0	0	1	0	0	0	0	0	1	1	0	0	0	0	0	0	0	0	0	1	0	0	1	1	0	0	0	0	0	1	0	0	0	1	1	1	1	1	0	1	0	0	0	1	0	1	1	1	0	1
seedBP4.seedMinPu0.01.seedNoGU	36	0	0	0	1	1	1	0	1	0	1	1	0	0	1	1	0	1	0	0	0	0	1	1	1	0	0	1	1	0	0	0	0	0	0	0	1	1	1	0	1	1	0	0	0	1	0	1	1	0	0	0	0	0	0	0	0	0	0	0	0	1	1	0	0	0	0	0	0	0	0	0	0	0	0	1	1	0	0	0	1	0	1	0	0	0	0	0	1	1	1	1	0	0	0	0	1	0	1	0	1	0	1
seedBP4.seedMinPu0.0001.seedNoGU	35	0	0	0	1	1	1	0	1	0	1	1	0	0	1	1	0	1	0	0	0	0	1	0	1	0	0	1	1	0	0	0	0	0	0	0	1	1	1	0	1	1	0	0	0	1	0	1	1	0	0	0	0	0	0	0	0	0	0	0	0	1	1	0	0	0	0	0	0	0	0	0	0	0	0	1	1	0	0	0	1	0	1	0	0	0	0	0	1	1	1	1	0	0	0	0	1	0	1	0	1	0	1
seedBP4.seedMinPu0.001.seedNoGU	36	0	0	0	1	1	1	0	1	0	1	1	0	0	1	1	0	1	0	0	0	0	1	1	1	0	0	1	1	0	0	0	0	0	0	0	1	1	1	0	1	1	0	0	0	1	0	1	1	0	0	0	0	0	0	0	0	0	0	0	0	1	1	0	0	0	0	0	0	0	0	0	0	0	0	1	1	0	0	0	1	0	1	0	0	0	0	0	1	1	1	1	0	0	0	0	1	0	1	0	1	0	1
seedBP4.seedMinPu0.2	34	0	0	0	1	1	1	0	1	0	0	1	0	0	1	1	0	0	0	0	0	0	1	0	1	0	0	1	0	0	0	0	0	0	0	1	1	0	1	0	1	1	0	0	0	1	0	1	0	0	0	0	1	0	0	1	0	1	0	0	0	1	1	0	0	0	1	0	0	0	0	0	0	0	0	1	1	0	0	0	0	0	1	0	0	0	1	1	1	1	1	0	1	0	0	0	1	0	0	0	1	0	0
seedBP4.seedMinPu0.2.seedNoGU	34	0	0	0	1	1	1	0	1	0	0	1	0	0	1	1	0	0	0	0	0	0	1	0	1	0	0	1	0	0	0	0	0	1	0	1	1	0	1	0	1	1	0	0	0	1	0	0	0	0	0	0	1	0	0	1	0	1	0	0	0	1	1	0	0	0	1	0	0	0	0	0	0	0	0	1	1	0	0	0	0	0	1	0	0	0	1	1	1	1	1	0	1	0	0	0	1	0	0	0	1	0	0
seedBP4.seedMinPu0.5	20	0	1	0	1	0	1	0	0	0	0	0	0	0	0	0	0	0	0	0	1	1	1	0	1	0	0	0	0	0	0	0	0	1	0	0	1	0	0	0	1	0	0	0	0	0	0	0	0	1	0	0	0	0	0	0	0	1	0	0	0	0	1	0	0	0	0	1	0	0	0	0	1	0	1	0	0	0	0	0	0	0	0	0	0	1	1	0	1	0	0	0	1	0	0	0	0	0	0	0	0	0	0
seedBP4.seedMinPu0.5.seedNoGU	21	0	1	0	1	0	1	0	0	0	0	0	0	0	0	0	0	0	0	0	1	1	1	0	1	0	0	0	0	0	0	0	1	1	0	0	1	0	0	1	0	0	0	0	0	0	0	0	0	1	0	0	0	1	0	0	0	1	0	0	0	1	1	0	0	0	0	1	0	0	0	0	0	0	0	0	0	0	0	0	0	0	0	0	0	1	1	0	1	0	0	0	1	0	0	0	0	0	0	0	0	0	0
seedBP4.seedMinPu0.seedNoGU	35	0	0	0	1	1	1	0	1	0	1	1	0	0	1	1	0	1	0	0	0	0	1	0	1	0	0	1	1	0	0	0	0	0	0	0	1	1	1	0	1	1	0	0	0	1	0	1	1	0	0	0	0	0	0	0	0	0	0	0	0	1	1	0	0	0	0	0	0	0	0	0	0	0	0	1	1	0	0	0	1	0	1	0	0	0	0	0	1	1	1	1	0	0	0	0	1	0	1	0	1	0	1
seedBP4.seedNoGU	35	0	0	0	1	1	1	0	1	0	1	1	0	0	1	1	0	1	0	0	0	0	1	0	1	0	0	1	1	0	0	0	0	0	0	0	1	1	1	0	1	1	0	0	0	1	0	1	1	0	0	0	0	0	0	0	0	0	0	0	0	1	1	0	0	0	0	0	0	0	0	0	0	0	0	1	1	0	0	0	1	0	1	0	0	0	0	0	1	1	1	1	0	0	0	0	1	0	1	0	1	0	1
seedBP5	35	0	0	0	1	1	1	0	1	0	1	1	0	0	1	1	0	1	0	0	0	0	1	0	1	0	0	1	1	0	0	0	0	0	0	0	1	1	1	0	1	1	0	0	0	1	0	1	1	0	0	0	0	0	0	0	0	0	0	0	0	1	1	0	0	0	0	0	0	0	0	0	0	0	0	1	1	0	0	0	1	0	0	0	0	1	0	0	1	1	1	1	0	0	0	0	1	0	1	0	1	0	1
seedBP5.seedMinPu0	35	0	0	0	1	1	1	0	1	0	1	1	0	0	1	1	0	1	0	0	0	0	1	0	1	0	0	1	1	0	0	0	0	0	0	0	1	1	1	0	1	1	0	0	0	1	0	1	1	0	0	0	0	0	0	0	0	0	0	0	0	1	1	0	0	0	0	0	0	0	0	0	0	0	0	1	1	0	0	0	1	0	0	0	0	1	0	0	1	1	1	1	0	0	0	0	1	0	1	0	1	0	1
seedBP5.seedMinPu0.01	36	0	0	0	1	1	1	0	1	0	1	1	0	0	1	1	0	1	0	0	0	0	1	0	1	0	0	1	1	0	0	0	0	0	0	0	1	1	1	0	1	1	0	0	0	1	0	1	1	0	0	0	0	0	0	0	0	0	0	0	0	1	1	0	0	0	0	0	0	0	0	0	0	0	0	1	1	0	0	0	1	0	1	0	0	1	0	0	1	1	1	1	0	0	0	0	1	0	1	0	1	0	1
seedBP5.seedMinPu0.001	35	0	0	0	1	1	1	0	1	0	1	1	0	0	1	1	0	1	0	0	0	0	1	0	1	0	0	1	1	0	0	0	0	0	0	0	1	1	1	0	1	1	0	0	0	1	0	1	1	0	0	0	0	0	0	0	0	0	0	0	0	1	1	0	0	0	0	0	0	0	0	0	0	0	0	1	1	0	0	0	1	0	0	0	0	1	0	0	1	1	1	1	0	0	0	0	1	0	1	0	1	0	1
seedBP5.seedMinPu0.0001	35	0	0	0	1	1	1	0	1	0	1	1	0	0	1	1	0	1	0	0	0	0	1	0	1	0	0	1	1	0	0	0	0	0	0	0	1	1	1	0	1	1	0	0	0	1	0	1	1	0	0	0	0	0	0	0	0	0	0	0	0	1	1	0	0	0	0	0	0	0	0	0	0	0	0	1	1	0	0	0	1	0	0	0	0	1	0	0	1	1	1	1	0	0	0	0	1	0	1	0	1	0	1
seedBP5.seedMinPu0.1	37	0	0	0	1	1	1	0	1	0	1	1	0	0	1	1	0	0	0	0	0	0	1	1	1	0	0	1	1	0	0	0	0	1	0	0	1	1	1	0	1	1	0	0	0	1	0	1	0	0	0	0	0	0	0	1	0	0	0	0	0	0	1	0	0	0	1	0	0	0	0	0	1	0	0	1	1	0	0	0	1	0	1	0	0	0	1	1	1	1	1	0	0	0	0	0	1	0	0	1	1	0	0
seedBP5.seedMinPu0.001.seedNoGU	38	0	0	0	1	1	1	0	1	0	1	1	0	0	1	1	0	1	0	0	0	0	1	1	1	0	0	1	1	0	0	0	0	0	0	0	1	1	1	0	1	1	0	0	0	1	0	1	1	0	0	0	1	0	0	0	0	0	0	0	0	1	1	0	0	0	0	0	0	0	0	0	0	0	0	1	1	0	0	0	1	0	1	0	0	0	0	0	1	1	1	1	0	1	0	0	1	0	1	0	1	0	1
seedBP5.seedMinPu0.0001.seedNoGU	37	0	0	0	1	1	1	0	1	0	1	1	0	0	1	1	0	1	0	0	0	0	1	1	1	0	0	1	1	0	0	0	0	0	0	0	1	1	1	0	1	1	0	0	0	1	0	1	1	0	0	0	1	0	0	0	0	0	0	0	0	1	1	0	0	0	0	0	0	0	0	0	0	0	0	1	1	0	0	0	1	0	1	0	0	0	0	0	1	1	1	1	0	0	0	0	1	0	1	0	1	0	1
seedBP5.seedMinPu0.1.seedNoGU	38	0	1	0	1	1	1	0	1	0	1	1	0	0	1	1	0	0	0	0	0	0	1	1	1	0	0	1	0	0	0	0	0	1	0	1	1	1	1	0	1	1	0	0	0	1	0	1	0	0	0	0	0	0	0	1	0	1	0	0	0	0	1	0	0	0	1	0	0	0	0	0	0	0	1	0	1	0	0	0	1	0	1	0	0	0	1	1	1	1	0	0	1	0	0	0	1	0	0	1	1	0	0
seedBP5.seedMinPu0.01.seedNoGU	38	0	0	0	1	1	1	0	1	0	1	1	0	0	1	1	0	1	0	0	0	0	1	1	1	0	0	1	1	0	0	0	0	0	0	0	1	1	1	0	1	1	0	0	0	1	0	1	1	0	0	0	1	0	0	0	0	0	0	0	0	1	1	0	0	0	0	0	0	0	0	0	0	0	0	1	1	0	0	0	1	0	1	0	0	0	0	0	1	1	1	1	0	1	0	0	1	0	1	0	1	0	1
seedBP5.seedMinPu0.2	27	0	1	0	1	1	1	0	0	0	0	1	0	0	1	0	0	0	0	0	0	0	1	0	1	0	0	1	0	0	0	0	0	0	1	1	1	0	1	0	1	1	0	0	0	1	0	0	0	0	0	0	0	0	0	0	0	1	0	0	0	0	1	0	0	0	0	0	0	0	0	0	0	0	0	0	1	0	0	0	0	0	1	0	0	0	1	1	1	0	1	0	1	0	0	0	1	0	0	0	1	0	0
seedBP5.seedMinPu0.2.seedNoGU	30	0	1	0	1	1	1	0	0	0	0	1	0	0	1	0	0	0	0	0	1	1	1	0	1	0	0	1	0	0	0	0	0	1	0	1	1	0	1	0	1	1	0	0	0	1	0	0	0	1	0	0	0	0	0	0	0	1	0	0	0	0	1	0	0	0	0	1	0	0	0	0	0	0	0	0	0	0	1	0	0	0	1	0	0	0	1	1	1	0	0	0	1	0	0	0	1	0	0	0	1	0	0
seedBP5.seedMinPu0.5	17	0	0	0	1	0	1	0	0	0	0	0	0	0	0	0	0	0	0	0	0	1	1	0	1	0	0	0	0	0	0	0	0	0	0	0	0	0	1	1	1	1	0	0	0	0	0	0	0	1	0	0	0	1	0	0	0	1	0	1	0	0	1	0	0	0	0	1	0	0	0	0	0	0	0	0	0	0	0	0	0	0	0	0	0	1	0	0	0	0	0	0	1	0	0	0	0	0	0	0	0	0	0
seedBP5.seedMinPu0.5.seedNoGU	18	0	0	0	1	0	1	0	0	0	0	0	0	0	0	0	0	0	0	0	0	1	1	0	1	0	0	0	0	0	0	0	0	0	0	0	0	0	1	0	1	1	0	0	0	0	0	0	0	1	0	0	0	1	0	0	0	1	0	1	0	0	1	0	0	0	0	1	0	0	0	0	0	0	0	1	0	0	0	1	0	0	0	0	0	1	0	0	0	0	0	0	1	0	0	0	0	0	0	0	0	0	0
seedBP5.seedMinPu0.seedNoGU	37	0	0	0	1	1	1	0	1	0	1	1	0	0	1	1	0	1	0	0	0	0	1	1	1	0	0	1	1	0	0	0	0	0	0	0	1	1	1	0	1	1	0	0	0	1	0	1	1	0	0	0	1	0	0	0	0	0	0	0	0	1	1	0	0	0	0	0	0	0	0	0	0	0	0	1	1	0	0	0	1	0	1	0	0	0	0	0	1	1	1	1	0	0	0	0	1	0	1	0	1	0	1

Echericha coli NC_000913

	sum	ArcZ-b1892	ArcZ-b2741	ArcZ-b3546	ChiX-b0619	ChiX-b0681	ChiX-b1737	CyaR-b0723	CyaR-b0814	CyaR-b1740	CyaR-b1824	CyaR-b2416	CyaR-b2666	CyaR-b2687	DsrA-b1237	DsrA-b2741	DsrA-b3251	FnrS-b0723	FnrS-b0755	FnrS-b0887	FnrS-b1107	FnrS-b1479	FnrS-b1531	FnrS-b1656	FnrS-b1841	FnrS-b2153	FnrS-b2303	FnrS-b2531	FnrS-b3829	FnrS-b3908	GcvB-b1040	GcvB-b1130	GcvB-b3089	GcvB-b4208	MicA-b0411	MicA-b0814	MicA-b0957	MicA-b1130	MicC-b2215	MicF-b0241	MicF-b0889	MicF-b0929	MicF-b3912	OxyS-b1892	OxyS-b2731	RprA-b1040	RprA-b1341	RprA-b2741	RybB-b0081	RybB-b0721	RybB-b0805	RybB-b1256	RybB-b2215	RybB-b2594	RyhB-b0118
Target found among top-100 predictions for the sRNA for any tested parameterization	86	0	1	0	1	1	1	1	1	0	1	1	1	1	1	1	0	1	0	1	1	1	1	1	1	1	1	1	1	0	1	1	1	1	1	1	1	1	1	1	1	1	1	1	0	1	1	1	1	1	1	1	1	1	0
seedBP5.seedNoGU	37	0	0	0	1	1	1	0	1	0	1	1	0	0	1	1	0	1	0	0	0	0	1	1	1	0	0	1	1	0	0	0	0	0	0	0	1	1	1	0	1	1	0	0	0	1	0	1	1	0	0	0	1	0	0
seedBP6	36	0	0	0	1	1	1	0	1	0	1	1	0	0	1	1	0	1	0	0	0	0	1	0	1	0	0	1	1	0	0	0	0	0	0	0	1	1	1	0	1	1	0	0	0	1	0	1	1	0	0	0	0	0	0
seedBP6.seedMinPu0	36	0	0	0	1	1	1	0	1	0	1	1	0	0	1	1	0	1	0	0	0	0	1	0	1	0	0	1	1	0	0	0	0	0	0	0	1	1	1	0	1	1	0	0	0	1	0	1	1	0	0	0	0	0	0
seedBP6.seedMinPu0.1	33	0	1	0	1	1	1	0	0	0	1	1	0	0	1	1	0	0	0	0	0	0	1	1	1	0	0	1	0	0	0	0	0	1	0	1	1	1	1	0	1	1	0	0	0	1	0	0	0	0	0	0	0	0	0
seedBP6.seedMinPu0.0001	36	0	0	0	1	1	1	0	1	0	1	1	0	0	1	1	0	1	0	0	0	0	1	0	1	0	0	1	1	0	0	0	0	0	0	0	1	1	1	0	1	1	0	0	0	1	0	1	1	0	0	0	0	0	0
seedBP6.seedMinPu0.001	35	0	0	0	1	1	1	0	1	0	1	1	0	0	1	1	0	1	0	0	0	0	1	0	1	0	0	1	1	0	0	0	0	0	0	0	1	1	1	0	1	1	0	0	0	1	0	1	1	0	0	0	0	0	0
seedBP6.seedMinPu0.01	36	0	0	0	1	1	1	0	1	0	1	1	0	0	1	1	0	1	0	0	0	0	1	1	1	0	0	1	1	0	0	0	0	0	0	0	1	1	1	0	1	1	0	0	0	1	0	1	1	0	0	0	0	0	0
seedBP6.seedMinPu0.0001.seedNoGU	39	0	0	0	1	1	1	0	1	0	1	1	0	0	1	1	0	0	0	0	0	0	1	1	1	0	0	1	1	0	0	0	0	0	0	0	1	1	1	0	1	1	0	0	0	1	0	1	0	0	0	0	1	0	0
seedBP6.seedMinPu0.1.seedNoGU	37	0	1	0	1	1	1	0	0	0	1	1	0	0	1	1	0	0	0	0	0	1	1	1	1	0	0	1	0	0	0	1	0	1	0	1	1	1	1	1	1	1	0	0	0	0	0	0	0	0	0	1	0	0	0
seedBP6.seedMinPu0.01.seedNoGU	43	0	0	0	1	1	1	0	1	0	1	1	0	0	1	1	0	0	0	0	0	0	1	1	1	0	0	1	1	0	0	0	0	1	0	1	1	1	1	0	1	1	0	0	0	1	0	1	0	0	0	0	0	0	0
seedBP6.seedMinPu0.001.seedNoGU	40	0	0	0	1	1	1	0	1	0	1	1	0	0	1	1	0	0	0	0	0	0	1	1	1	0	0	1	1	0	0	0	0	0	0	0	1	1	1	0	1	1	0	0	0	1	0	1	0	0	0	0	1	0	0
seedBP6.seedMinPu0.2	21	0	1	0	1	1	1	0	0	0	0	1	0	0	0	1	0	0	0	0	0	1	1	0	1	0	0	1	0	0	0	0	0	0	0	0	0	0	1	1	1	1	0	0	0	0	0	0	0	0	0	0	0	0	0
seedBP6.seedMinPu0.2.seedNoGU	22	0	1	0	1	1	1	0	0	0	0	1	0	0	0	1	0	0	0	0	0	1	1	0	1	0	1	1	0	0	0	0	1	0	0	0	0	0	1	1	1	1	0	0	0	0	0	0	0	0	0	0	0	0	0
seedBP6.seedMinPu0.5	8	0	1	0	0	0	0	0	0	0	0	0	0	0	0	0	0	0	0	0	0	1	1	0	1	0	0	0	0	0	0	0	0	0	0	0	0	0	1	1	0	1	0	0	0	0	0	0	0	0	0	0	0	0	0
seedBP6.seedMinPu0.5.seedNoGU	10	0	1	0	0	0	0	0	0	0	0	0	0	0	0	0	0	0	0	0	0	1	1	0	1	0	0	0	0	0	0	0	0	0	0	0	0	0	1	0	0	1	0	0	0	1	0	0	0	0	0	0	0	0	0
seedBP6.seedMinPu0.seedNoGU	38	0	0	0	1	1	1	0	1	0	1	1	0	0	1	1	0	0	0	0	0	0	1	1	1	0	0	1	1	0	0	0	0	0	0	0	1	1	1	0	1	1	0	0	0	1	0	1	0	0	0	0	1	0	0
seedBP6.seedNoGU	38	0	0	0	1	1	1	0	1	0	1	1	0	0	1	1	0	0	0	0	0	0	1	1	1	0	0	1	1	0	0	0	0	0	0	0	1	1	1	0	1	1	0	0	0	1	0	1	0	0	0	0	1	0	0
seedBP7	36	0	0	0	1	1	1	0	1	0	1	1	0	0	1	1	0	1	0	0	0	0	1	0	1	0	0	1	1	0	0	0	0	0	0	0	1	1	1	0	1	1	0	0	0	1	0	1	1	0	0	0	0	0	0
seedBP7.seedMinPu0	36	0	0	0	1	1	1	0	1	0	1	1	0	0	1	1	0	1	0	0	0	0	1	0	1	0	0	1	1	0	0	0	0	0	0	0	1	1	1	0	1	1	0	0	0	1	0	1	1	0	0	0	0	0	0
seedBP7.seedMinPu0.001	37	0	0	0	1	1	1	0	1	0	1	1	0	0	1	1	0	1	0	0	0	0	1	1	1	0	0	1	1	0	0	0	0	0	0	0	1	1	1	0	1	1	0	0	0	1	0	1	1	0	0	0	0	0	0
seedBP7.seedMinPu0.01	40	0	0	0	1	1	1	0	1	0	1	1	0	0	1	1	0	1	0	0	0	0	1	1	1	0	0	1	1	0	0	0	0	0	0	0	1	1	1	0	1	1	0	0	0	1	0	1	1	0	0	0	0	0	0
seedBP7.seedMinPu0.1	25	0	1	0	1	1	1	1	0	0	0	0	0	0	1	1	0	0	0	0	0	0	1	0	1	0	0	1	0	0	0	0	0	1	0	1	0	0	1	1	1	1	0	0	0	0	0	0	0	0	0	0	0	0	0
seedBP7.seedMinPu0.0001	37	0	0	0	1	1	1	0	1	0	1	1	0	0	1	1	0	1	0	0	0	0	1	1	1	0	0	1	1	0	0	0	0	0	0	0	1	1	1	0	1	1	0	0	0	1	0	1	1	0	0	0	0	0	0
seedBP7.seedMinPu0.0001.seedNoGU	47	0	0	0	1	1	1	0	1	0	1	1	0	0	1	1	0	0	0	0	0	0	1	0	1	0	0	1	1	0	0	0	0	1	0	1	1	1	1	1	0	1	0	0	0	1	0	1	0	0	1	0	1	0	0
seedBP7.seedMinPu0.001.seedNoGU	49	0	0	0	1	1	1	0	1	0	1	1	0	0	1	1	0	0	0	0	0	0	1	0	1	0	0	1	1	0	0	0	0	1	0	1	1	1	1	1	0	1	0	0	0	1	0	1	0	0	1	0	1	0	0
seedBP7.seedMinPu0.1.seedNoGU	25	0	1	0	1	1	1	1	0	0	0	0	1	0	1	1	0	0	0	0	0	0	1	0	1	0	1	1	0	0	0	0	1	1	0	0	0	0	1	1	0	1	0	0	0	0	0	0	0	0	0	0	0	0	0
seedBP7.seedMinPu0.01.seedNoGU	47	0	1	0	1	1	1	0	1	0	1	1	0	0	1	1	0	0	0	0	0	0	1	0	1	0	0	1	1	0	0	0	0	1	0	1	1	1	1	1	0	1	0	0	0	1	0	1	0	0	1	1	0	0	0
seedBP7.seedMinPu0.2	17	0	0	0	1	1	1	0	0	0	0	0	0	0	0	1	0	0	0	0	0	0	1	0	1	0	1	1	0	0	0	0	1	0	0	0	0	0	1	1	1	1	0	0	0	0	1	0	0	0	0	0	0	0	0
seedBP7.seedMinPu0.2.seedNoGU	16	0	0	0	1	1	1	0	0	0	0	0	0	0	0	0	0	0	0	0	0	0	1	0	1	0	1	1	0	0	0	1	1	0	0	0	0	0	1	1	0	1	0	0	0	0	0	0	0	0	0	0	0	0	0
seedBP7.seedMinPu0.5	8	0	1	0	0	0	0	0	0	0	0	0	0	0	0	0	0	0	0	0	0	1	1	0	1	0	0	0	0	0	0	0	0	0	0	0	0	0	1	0	0	1	0	0	0	0	0	0	0	0	0	0	0	0	0
seedBP7.seedMinPu0.5.seedNoGU	7	0	1	0	0	0	0	0	0	0	0	0	0	0	0	0	0	0	0	0	0	0	1	0	1	0	0	0	0	0	0	0	0	0	0	0	0	0	1	0	0	1	0	0	0	0	0	0	0	0	0	0	0	0	0
seedBP7.seedMinPu0.seedNoGU	47	0	0	0	1	1	1	0	1	0	1	1	0	0	1	1	0	0	0	0	0	0	1	0	1	0	0	1	1	0	0	0	0	1	0	1	1	1	1	1	0	1	0	0	0	1	0	1	0	0	1	0	1	0	0
seedBP7.seedNoGU	47	0	0	0	1	1	1	0	1	0	1	1	0	0	1	1	0	0	0	0	0	0	1	0	1	0	0	1	1	0	0	0	0	1	0	1	1	1	1	1	0	1	0	0	0	1	0	1	0	0	1	0	1	0	0
seedBP8	38	0	0	0	1	1	1	0	1	0	1	1	0	0	1	1	0	1	0	0	0	0	1	1	1	0	0	1	1	0	0	0	0	0	0	0	1	1	1	0	1	1	0	0	0	1	0	1	1	0	0	0	1	0	0
seedBP8.seedMinPu0	38	0	0	0	1	1	1	0	1	0	1	1	0	0	1	1	0	1	0	0	0	0	1	1	1	0	0	1	1	0	0	0	0	0	0	0	1	1	1	0	1	1	0	0	0	1	0	1	1	0	0	0	1	0	0
seedBP8.seedMinPu0.1	22	0	1	0	1	1	0	0	0	0	0	0	1	0	1	1	0	0	0	0	0	0	0	0	1	0	0	1	0	0	0	0	1	1	0	0	0	0	1	1	1	1	1	0	0	0	0	0	0	0	0	0	0	0	0
seedBP8.seedMinPu0.0001	38	0	0	0	1	1	1	0	1	0	1	1	0	0	1	1	0	1	0	0	0	0	1	1	1	0	0	1	1	0	0	0	0	0	0	0	1	1	1	0	1	1	0	0	0	1	0	1	1	0	0	0	1	0	0
seedBP8.seedMinPu0.001	41	0	0	0	1	1	1	0	0	0	1	1	0	0	1	1	0	1	0	0	0	0	1	1	1	0	0	1	1	0	0	0	0	0	0	0	1	1	1	0	1	1	0	0	0	1	0	1	1	0	0	0	1	0	0
seedBP8.seedMinPu0.01	41	0	1	0	1	1	1	0	0	0	1	1	0	0	1	1	0	1	0	0	0	0	1	1	1	0	0	1	1	0	0	0	0	0	0	1	1	1	1	0	1	1	0	0	0	1	0	1	1	0	0	0	0	0	0
seedBP8.seedMinPu0.001.seedNoGU	47	0	1	0	1	1	1	0	0	0	0	1	0	0	1	1	0	0	0	1	0	1	1	0	1	0	0	1	1	0	0	1	1	1	0	0	1	1	1	1	0	1	0	0	0	1	0	1	0	0	0	0	1	0	0
seedBP8.seedMinPu0.0001.seedNoGU	49	0	0	0	1	1	1	0	1	0	0	1	0	0	1	1	0	0	0	1	0	1	1	0	1	0	0	1	1	0	0	1	1	1	0	0	1	1	1	1	0	1	0	0	0	1	0	1	0	0	0	0	1	0	0
seedBP8.seedMinPu0.1.seedNoGU	20	0	1	0	1	1	0	0	0	0	0	0	1	0	1	1	0	0	0	0	0	0	0	0	1	0	0	1	0	0	0	1	0	1	0	0	0	0	1	1	0	1	0	0	0	0	0	0	0	0	0	0	0	0	0
seedBP8.seedMinPu0.01.seedNoGU	43	0	1	0	1	1	1	0	0	0	0	1	1	0	1	1	0	0	0	1	0	1	1	0	1	0	0	1	1	0	0	1	1	1	0	0	1	1	1	1	0	1	0	0	0	1	0	1	0	0	0	0	0	0	0
seedBP8.seedMinPu0.2	10	0	0	0	1	0	0	0	0	0	0	0	0	0	0	0	0	0	0	0	0	0	0	0	1	0	0	1	0	0	0	0	0	0	0	0	0	0	1	1	0	1	0	0	0	0	0	0	0	0	0	0	0	0	0
seedBP8.seedMinPu0.2.seedNoGU	9	0	0	0	1	0	0	0	0	0	0	0	0	0	0	0	0	0	0	0	0	0	0	0	1	0	0	1	0	0	0	0	0	0	0	0	0	0	0	1	0	1	0	0	0	0	0	0	0	0	0	0	0	0	0
seedBP8.seedMinPu0.5	4	0	0	0	0	0	0	0	0	0	0	0	0	0	0	0	0	0	0	0	0	0	0	0	0	0	1	0	0	0	0	0	0	0	0	0	0	0	0	0	0	1	0	0	0	0	0	0	0	0	0	0	0	0	0
seedBP8.seedMinPu0.5.seedNoGU	2	0	0	0	0	0	0	0	0	0	0	0	0	0	0	0	0	0	0	0	0	0	0	0	0	0	0	0	0	0	0	0	0	0	0	0	0	0	0	0	0	1	0	0	0	0	0	0	0	0	0	0	0	0	0
seedBP8.seedMinPu0.seedNoGU	49	0	0	0	1	1	1	0	1	0	0	1	0	0	1	1	0	0	0	1	0	1	1	0	1	0	0	1	1	0	0	1	1	1	0	0	1	1	1	1	0	1	0	0	0	1	0	1	0	0	0	0	1	0	0
seedBP8.seedNoGU	49	0	0	0	1	1	1	0	1	0	0	1	0	0	1	1	0	0	0	1	0	1	1	0	1	0	0	1	1	0	0	1	1	1	0	0	1	1	1	1	0	1	0	0	0	1	0	1	0	0	0	0	1	0	0
seedBP9	33	0	0	0	1	1	1	0	1	0	1	1	0	0	1	1	0	0	0	0	0	0	0	0	1	0	0	1	0	0	0	0	0	1	0	0	1	1	1	0	1	1	0	0	0	1	0	0	1	0	0	0	0	0	0
seedBP9.seedNoGU	33	0	1	0	1	1	1	0	1	0	0	0	0	0	1	1	0	0	0	0	0	1	0	0	1	0	0	1	0	0	0	0	1	1	0	0	1	1	1	1	0	1	0	0	0	1	0	0	0	0	0	0	0	0	0
seedBP10	30	0	0	0	1	1	1	0	0	0	0	0	0	0	1	1	0	0	0	0	0	0	0	0	1	0	0	1	0	0	0	0	0	1	0	0	1	1	1	1	1	1	0	0	0	1	0	0	1	0	0	0	0	0	0
seedBP10.seedNoGU	27	0	1	0	1	1	1	0	0	0	0	0	0	0	1	1	0	0	0	0	0	1	0	0	0	0	0	1	0	0	0	0	0	1	0	0	0	1	1	1	0	1	0	0	0	1	0	0	0	0	0	0	0	0	0
seedBP11	28	0	0	0	1	1	1	0	0	0	0	0	0	0	1	1	0	0	0	0	0	1	0	0	1	0	0	1	0	0	0	0	0	0	1	0	1	0	1	1	0	1	1	0	0	1	1	0	0	0	0	0	0	0	0
seedBP11.seedNoGU	16	0	0	0	1	1	1	0	0	0	0	0	0	0	1	1	0	0	0	0	0	1	0	0	0	0	0	1	0	0	0	0	0	0	0	0	0	0	1	1	0	1	0	0	0	1	0	0	0	0	0	0	0	0	0
seedBP12	24	0	0	0	1	1	1	0	0	0	0	0	0	0	1	1	0	0	0	0	0	0	0	0	0	0	0	1	0	0	0	0	0	0	0	0	1	0	1	1	0	1	1	0	0	1	0	0	0	0	0	0	0	0	0
seedBP12.seedNoGU	12	0	0	0	1	1	1	0	0	0	0	0	0	0	1	1	0	0	0	0	0	0	0	0	0	0	0	1	0	0	0	0	0	0	0	0	0	0	1	1	0	1	0	0	0	1	0	0	0	0	0	0	0	0	0

	RyhB-b0156	RyhB-b0288	RyhB-b0592	RyhB-b0683	RyhB-b0721	RyhB-b0723	RyhB-b0894	RyhB-b1107	RyhB-b1200	RyhB-b1452	RyhB-b1531	RyhB-b1588	RyhB-b1612	RyhB-b1656	RyhB-b1778	RyhB-b1981	RyhB-b2069	RyhB-b2155	RyhB-b2206	RyhB-b2530	RyhB-b3365	RyhB-b3607	RyhB-b3942	RyhB-b4070	RyhB-b4122	SgrS-b1101	SgrS-b1817	SgrS-b2416	Spot42-b0039	Spot42-b0720	Spot42-b0721	Spot42-b0728	Spot42-b0757	Spot42-b1136	Spot42-b1302	Spot42-b1398	Spot42-b1761	Spot42-b1901	Spot42-b2221	Spot42-b2702	Spot42-b2715	Spot42-b2801	Spot42-b2802	Spot42-b3224	Spot42-b3566	Spot42-b3927	Spot42-b3962	Spot42-b4311
Target found among top-100 predictions for the sRNA for any tested parameterization	1	1	1	0	1	0	1	1	1	0	0	1	1	1	1	1	0	1	1	1	1	1	0	1	1	1	1	1	0	1	1	1	1	1	1	1	1	1	1	1	1	1	0	1	1	1	1	1
seedBP5.seedNoGU	0	0	0	0	0	0	1	1	0	0	0	0	0	0	0	0	0	0	0	0	1	1	0	0	0	1	0	1	0	0	0	0	0	1	1	1	1	0	0	0	0	1	0	1	0	1	0	1
seedBP6	0	0	0	0	0	0	1	1	0	0	0	0	0	0	0	0	0	0	0	0	1	1	0	0	0	1	0	0	0	0	1	0	1	1	1	1	1	0	0	0	0	1	0	1	0	1	0	1
seedBP6.seedMinPu0	0	0	0	0	0	0	1	1	0	0	0	0	0	0	0	0	0	0	0	0	1	1	0	0	0	1	0	0	0	0	1	0	1	1	1	1	1	0	0	0	0	1	0	1	0	1	0	1
seedBP6.seedMinPu0.1	0	0	1	0	0	0	0	1	0	0	0	0	0	0	1	0	0	1	0	1	0	1	0	0	0	0	0	0	0	0	0	1	1	1	1	0	0	1	0	0	0	1	0	0	0	1	0	0
seedBP6.seedMinPu0.0001	0	0	0	0	0	0	1	1	0	0	0	0	0	0	0	0	0	0	0	0	1	1	0	0	0	1	0	0	0	0	1	0	1	1	1	1	1	0	0	0	0	1	0	1	0	1	0	1
seedBP6.seedMinPu0.001	0	0	0	0	0	0	1	1	0	0	0	0	0	0	0	0	0	0	0	0	1	1	0	0	0	1	0	0	0	0	1	0	0	1	1	1	1	0	0	0	0	1	0	1	0	1	0	1
seedBP6.seedMinPu0.01	0	0	0	0	0	0	1	1	0	0	0	0	0	0	0	0	0	0	0	0	1	1	0	0	0	1	0	1	0	0	0	0	0	1	1	1	1	0	0	0	0	1	0	1	0	1	0	1
seedBP6.seedMinPu0.0001.seedNoGU	0	0	0	0	0	0	1	1	0	0	0	0	0	0	0	0	0	0	0	0	1	1	0	0	0	1	0	1	0	0	1	0	1	1	1	1	1	1	1	0	0	1	0	1	0	1	0	1
seedBP6.seedMinPu0.1.seedNoGU	0	0	1	0	0	0	0	0	0	0	0	0	1	0	1	0	0	0	0	1	0	1	0	1	0	0	0	0	0	0	0	1	1	1	1	0	0	1	0	0	0	1	0	0	0	1	1	0
seedBP6.seedMinPu0.01.seedNoGU	0	0	0	0	1	0	1	1	0	0	0	1	0	0	0	0	0	1	0	0	1	1	0	0	0	1	0	1	0	0	0	1	1	1	1	1	1	1	1	0	0	1	0	1	1	1	0	0
seedBP6.seedMinPu0.001.seedNoGU	0	0	0	0	1	0	1	1	0	0	0	0	0	0	0	0	0	1	0	0	1	1	0	0	0	1	0	1	0	0	0	0	1	1	1	1	1	1	1	0	0	1	0	1	0	1	0	1
seedBP6.seedMinPu0.2	0	0	1	0	0	0	0	0	0	0	0	0	1	0	0	0	0	0	0	0	0	0	0	1	0	0	0	0	0	0	0	0	0	1	0	0	0	1	0	0	0	1	0	0	0	1	0	0
seedBP6.seedMinPu0.2.seedNoGU	0	0	0	0	0	0	0	0	0	0	0	0	1	0	0	0	0	0	0	0	0	0	0	1	0	0	0	0	0	0	0	0	0	1	0	0	0	1	0	0	0	1	0	0	0	1	0	0
seedBP6.seedMinPu0.5	0	0	0	0	0	0	0	0	0	0	0	0	1	0	0	0	0	0	0	0	0	0	0	0	0	0	0	0	0	0	0	0	0	0	0	0	0	0	0	0	0	0	0	0	0	0	0	0
seedBP6.seedMinPu0.5.seedNoGU	0	0	0	0	0	0	0	0	1	0	0	0	1	0	0	0	0	0	0	0	0	0	0	0	0	0	0	0	0	0	0	0	0	0	0	0	0	0	1	0	0	0	0	0	0	0	0	0
seedBP6.seedMinPu0.seedNoGU	0	0	0	0	0	0	1	1	0	0	0	0	0	0	0	0	0	0	0	0	1	1	0	0	0	1	0	1	0	0	1	0	1	1	1	1	1	0	1	0	0	1	0	1	0	1	0	1
seedBP6.seedNoGU	0	0	0	0	0	0	1	1	0	0	0	0	0	0	0	0	0	0	0	0	1	1	0	0	0	1	0	1	0	0	1	0	1	1	1	1	1	0	1	0	0	1	0	1	0	1	0	1
seedBP7	0	0	0	0	0	0	1	1	0	0	0	0	0	0	0	0	0	0	0	0	1	1	0	0	0	1	0	0	0	0	1	0	1	1	1	1	1	0	0	0	0	1	0	1	0	1	0	1
seedBP7.seedMinPu0	0	0	0	0	0	0	1	1	0	0	0	0	0	0	0	0	0	0	0	0	1	1	0	0	0	1	0	0	0	0	1	0	1	1	1	1	1	0	0	0	0	1	0	1	0	1	0	1
seedBP7.seedMinPu0.001	0	0	0	0	0	0	1	1	0	0	0	0	0	0	0	0	0	0	0	0	1	1	0	0	0	1	0	1	0	0	1	0	0	1	1	1	1	0	0	0	0	1	0	1	0	1	0	1
seedBP7.seedMinPu0.01	1	0	0	0	0	0	1	1	0	0	0	0	0	0	0	0	0	1	0	0	1	1	0	0	0	1	0	1	0	0	0	0	0	1	1	1	1	1	1	0	0	1	0	1	0	1	0	1
seedBP7.seedMinPu0.1	0	0	0	0	0	0	0	1	0	0	0	0	0	0	1	0	0	0	0	0	0	0	0	1	0	0	0	0	0	0	0	0	1	1	0	0	0	1	0	0	0	1	0	0	0	1	1	0
seedBP7.seedMinPu0.0001	0	0	0	0	0	0	1	1	0	0	0	0	0	0	0	0	0	0	0	0	1	1	0	0	0	1	0	0	0	0	1	0	1	1	1	1	1	0	0	0	0	1	0	1	0	1	0	1
seedBP7.seedMinPu0.0001.seedNoGU	1	0	1	0	1	0	1	0	0	0	0	1	0	0	0	0	0	1	1	0	1	1	0	0	0	1	0	1	0	0	1	1	1	1	1	1	1	0	1	0	1	1	0	1	1	1	0	0
seedBP7.seedMinPu0.001.seedNoGU	1	0	1	0	1	0	1	0	0	0	0	1	0	0	0	0	0	1	1	1	1	1	0	0	1	1	0	1	0	0	0	1	1	1	1	1	1	1	1	0	1	1	0	1	1	1	0	0
seedBP7.seedMinPu0.1.seedNoGU	0	0	0	0	0	0	0	0	0	0	0	0	1	0	1	0	0	0	0	0	0	0	0	1	0	0	0	0	0	0	0	0	0	1	0	0	0	1	0	0	0	1	0	0	0	1	1	0
seedBP7.seedMinPu0.01.seedNoGU	0	0	1	0	0	0	1	0	0	0	0	1	0	0	1	0	0	1	1	1	1	1	0	0	0	1	0	1	0	0	0	1	1	1	1	1	1	1	1	0	1	1	0	1	0	1	0	0
seedBP7.seedMinPu0.2	0	0	0	0	0	0	0	0	0	0	0	0	0	0	0	0	0	0	0	0	0	0	0	0	0	0	0	0	0	0	0	0	0	0	0	0	0	1	0	0	0	1	0	0	0	1	0	0
seedBP7.seedMinPu0.2.seedNoGU	0	0	0	0	0	0	0	0	0	0	0	0	0	0	0	0	0	0	0	0	0	0	0	0	0	0	0	0	1	0	0	0	0	0	0	0	0	1	0	0	0	1	0	0	0	1	0	0
seedBP7.seedMinPu0.5	0	0	0	0	0	0	0	0	0	0	0	0	0	0	0	0	0	0	0	0	1	0	0	0	0	0	0	0	1	0	0	0	0	0	0	0	0	0	0	0	0	0	0	0	0	0	0	0
seedBP7.seedMinPu0.5.seedNoGU	0	0	0	0	0	0	0	0	0	0	0	0	0	0	0	0	0	0	0	0	0	0	0	0	0	0	0	0	1	0	0	0	1	0	0	0	0	0	0	0	0	0	0	0	0	0	0	0
seedBP7.seedMinPu0.seedNoGU	1	0	1	0	1	0	1	0	0	0	0	1	0	0	0	0	0	1	1	0	1	1	0	0	0	1	0	1	0	0	1	1	1	1	1	1	1	0	1	0	1	1	0	1	1	1	0	0
seedBP7.seedNoGU	1	0	1	0	1	0	1	0	0	0	0	1	0	0	0	0	0	1	1	0	1	1	0	0	0	1	0	1	0	0	1	1	1	1	1	1	1	0	1	0	1	1	0	1	1	1	0	0
seedBP8	0	0	0	0	0	0	1	1	0	0	0	0	0	0	0	0	0	0	0	0	0	1	0	0	0	1	0	1	0	0	1	0	1	1	1	1	1	0	0	0	0	1	0	1	0	1	0	1
seedBP8.seedMinPu0	0	0	0	0	0	0	1	1	0	0	0	0	0	0	0	0	0	0	0	0	0	1	0	0	0	1	0	1	0	0	1	0	1	1	1	1	1	0	0	0	0	1	0	1	0	1	0	1
seedBP8.seedMinPu0.1	0	0	1	0	0	0	0	1	0	0	0	0	0	0	0	0	0	0	0	0	0	0	0	1	0	0	0	0	0	0	0	0	0	1	0	0	0	1	0	0	0	1	0	0	0	1	0	0
seedBP8.seedMinPu0.0001	0	0	0	0	0	0	1	1	0	0	0	0	0	0	0	0	0	0	0	0	0	1	0	0	0	1	0	1	0	0	1	0	1	1	1	1	1	0	0	0	0	1	0	1	0	1	0	1
seedBP8.seedMinPu0.001	1	0	0	0	1	0	1	1	0	0	0	0	0	0	0	0	0	1	0	0	0	1	0	0	0	1	0	1	0	0	1	0	0	1	1	1	1	1	1	0	0	1	0	1	0	1	0	1
seedBP8.seedMinPu0.01	1	0	1	0	0	0	1	1	0	0	0	0	0	0	0	0	0	1	0	0	0	1	0	0	0	1	0	1	0	0	0	0	1	1	1	1	0	1	0	0	1	1	0	1	0	1	0	1
seedBP8.seedMinPu0.001.seedNoGU	0	0	1	0	1	0	1	0	0	0	0	0	0	0	1	0	0	1	1	1	0	1	0	0	1	1	0	1	0	1	0	0	1	1	1	1	1	1	1	0	1	1	0	1	0	1	0	0
seedBP8.seedMinPu0.0001.seedNoGU	1	0	1	0	1	0	1	0	0	0	0	0	0	0	1	0	0	1	1	1	0	1	0	0	1	1	0	1	0	1	1	0	1	1	1	1	1	1	1	0	1	1	0	1	0	1	0	0
seedBP8.seedMinPu0.1.seedNoGU	0	0	0	0	0	0	1	0	0	0	0	0	0	0	0	0	0	0	0	0	0	0	0	1	0	0	0	0	0	1	0	0	0	1	0	0	0	1	0	0	0	1	0	0	0	1	0	0
seedBP8.seedMinPu0.01.seedNoGU	0	1	1	0	0	0	1	0	0	0	0	0	0	1	1	0	0	0	0	1	0	1	0	1	0	1	0	1	0	0	0	0	0	1	1	1	0	1	0	0	1	1	0	1	0	1	1	0
seedBP8.seedMinPu0.2	0	0	0	0	0	0	0	0	0	0	0	0	0	0	0	0	0	0	0	0	0	0	0	0	0	0	0	1	0	0	0	0	0	0	0	0	0	1	0	0	0	1	0	0	0	1	0	0
seedBP8.seedMinPu0.2.seedNoGU	0	0	0	0	0	0	0	0	0	0	0	0	0	0	0	0	0	0	0	0	0	0	0	0	0	0	0	0	0	1	0	0	0	0	0	0	0	1	0	0	0	1	0	0	0	1	0	0
seedBP8.seedMinPu0.5	0	0	0	0	0	0	0	0	0	0	0	0	0	0	0	0	0	0	0	0	0	0	0	0	0	0	0	0	0	1	0	1	0	0	0	0	0	0	0	0	0	0	0	0	0	0	0	0
seedBP8.seedMinPu0.5.seedNoGU	0	0	0	0	0	0	0	0	0	0	0	0	0	0	0	0	0	0	0	0	0	0	0	0	0	0	0	0	0	1	0	0	0	0	0	0	0	0	0	0	0	0	0	0	0	0	0	0
seedBP8.seedMinPu0.seedNoGU	1	0	1	0	1	0	1	0	0	0	0	0	0	0	1	0	0	1	1	1	0	1	0	0	1	1	0	1	0	1	1	0	1	1	1	1	1	1	1	0	1	1	0	1	0	1	0	0
seedBP8.seedNoGU	1	0	1	0	1	0	1	0	0	0	0	0	0	0	1	0	0	1	1	1	0	1	0	0	1	1	0	1	0	1	1	0	1	1	1	1	1	1	1	0	1	1	0	1	0	1	0	0
seedBP9	1	0	0	0	1	0	0	1	0	0	0	0	0	0	0	0	0	1	0	0	0	1	0	0	0	0	0	1	0	0	1	0	1	1	0	1	1	0	0	0	0	1	0	1	0	1	0	1
seedBP9.seedNoGU	1	0	1	0	1	0	0	0	0	0	0	0	0	0	1	1	0	0	0	0	0	1	0	0	0	0	0	1	0	1	0	0	1	1	0	1	1	0	0	0	0	1	0	1	0	1	0	0
seedBP10	0	0	0	0	1	0	0	1	0	0	0	1	0	0	0	0	0	1	0	0	0	1	0	0	0	0	0	0	0	0	1	1	1	1	0	0	1	0	0	0	0	1	0	1	0	1	0	1
seedBP10.seedNoGU	0	0	1	0	0	0	0	0	0	0	0	0	0	0	1	1	0	0	0	0	0	1	0	1	0	0	0	0	0	1	0	0	1	1	0	0	1	0	0	0	1	1	0	1	0	0	0	1
seedBP11	0	0	0	0	1	0	0	1	0	0	0	0	0	0	1	0	0	1	0	0	0	1	0	0	0	0	0	0	0	0	1	1	0	1	0	0	0	0	0	0	0	1	0	1	0	1	0	1
seedBP11.seedNoGU	0	0	0	0	0	0	0	0	0	0	0	0	0	0	0	0	0	0	0	0	0	1	0	0	0	0	0	0	0	0	0	0	0	1	0	0	0	0	0	0	1	1	0	1	0	0	0	0
seedBP12	0	0	0	0	1	0	0	1	0	0	0	0	0	0	1	1	0	1	0	0	0	1	0	0	0	0	0	0	0	0	1	1	0	1	0	0	0	0	0	0	0	0	0	1	0	1	0	1
seedBP12.seedNoGU	0	0	0	0	0	0	0	0	0	0	0	0	0	0	0	0	0	0	0	0	0	1	0	0	0	0	0	0	0	0	0	0	0	0	0	0	0	0	0	0	0	0	0	1	0	0	0	0

Echericha coli NC_000913

	sum	ArcZ-b1892	ArcZ-b2741	ArcZ-b3546	ChiX-b0619	ChiX-b0681	ChiX-b1737	CyaR-b0723	CyaR-b0814	CyaR-b1740	CyaR-b1824	CyaR-b2416	CyaR-b2666	CyaR-b2687	DsrA-b1237	DsrA-b2741	DsrA-b3251	FnrS-b0723	FnrS-b0755	FnrS-b0887	FnrS-b1107	FnrS-b1479	FnrS-b1531	FnrS-b1656	FnrS-b1841	FnrS-b2153	FnrS-b2303	FnrS-b2531	FnrS-b3829	FnrS-b3908	GcvB-b1040	GcvB-b1130	GcvB-b3089	GcvB-b4208	MicA-b0411	MicA-b0814	MicA-b0957	MicA-b1130
Target found among top-100 predictions for the sRNA for any tested parameterization	86	0	1	0	1	1	1	1	1	0	1	1	1	1	1	1	0	1	0	1	1	1	1	1	1	1	1	1	1	0	1	1	1	1	1	1	1	1
seedBP13	14	0	0	0	1	0	0	0	0	0	0	0	0	0	1	1	0	0	0	0	0	0	0	0	0	0	0	1	0	0	0	0	1	0	0	0	0	0
seedBP13.seedNoGU	3	0	0	0	0	0	0	0	0	0	0	0	0	0	1	0	0	0	0	0	0	0	0	0	0	0	0	0	0	0	0	0	0	0	0	0	0	0
seedBP14	6	0	0	0	0	0	0	0	0	0	0	0	0	0	0	0	0	1	0	0	0	0	0	0	0	0	0	1	0	0	0	0	1	0	0	0	0	0
seedBP14.seedNoGU	1	0	0	0	0	0	0	0	0	0	0	0	0	0	0	0	0	0	0	0	0	0	0	0	0	0	0	0	0	0	0	0	0	0	0	0	0	0
seedMaxE0	36	0	0	0	1	1	1	0	1	0	1	1	0	0	1	1	0	1	0	0	0	0	1	0	1	0	0	1	1	0	0	0	0	0	0	0	1	1
seedMaxE0.seedMinPu0	36	0	0	0	1	1	1	0	1	0	1	1	0	0	1	1	0	1	0	0	0	0	1	0	1	0	0	1	1	0	0	0	0	0	0	0	1	1
seedMaxE0.seedMinPu0.01	40	0	0	0	1	1	1	0	1	0	1	1	0	0	1	1	0	1	0	0	0	0	1	1	1	0	0	1	1	0	0	0	0	0	0	0	1	1
seedMaxE0.seedMinPu0.001	37	0	0	0	1	1	1	0	1	0	1	1	0	0	1	1	0	1	0	0	0	0	1	1	1	0	0	1	1	0	0	0	0	0	0	0	1	1
seedMaxE0.seedMinPu0.0001	37	0	0	0	1	1	1	0	1	0	1	1	0	0	1	1	0	1	0	0	0	0	1	1	1	0	0	1	1	0	0	0	0	0	0	0	1	1
seedMaxE0.seedMinPu0.1	25	0	1	0	1	1	1	1	0	0	0	0	0	0	1	1	0	0	0	0	0	0	1	0	1	0	0	1	0	0	0	0	0	1	0	1	0	0
seedMaxE0.seedMinPu0.01.seedNoGU	47	0	1	0	1	1	1	0	1	0	1	1	0	0	1	1	0	0	0	0	0	0	1	0	1	0	0	1	1	0	0	0	0	1	0	1	1	1
seedMaxE0.seedMinPu0.001.seedNoGU	49	0	0	0	1	1	1	0	1	0	1	1	0	0	1	1	0	0	0	0	0	0	1	0	1	0	0	1	1	0	0	0	0	1	0	1	1	1
seedMaxE0.seedMinPu0.1.seedNoGU	25	0	1	0	1	1	1	1	0	0	0	0	1	0	1	1	0	0	0	0	0	0	1	0	1	0	1	1	0	0	0	0	1	1	0	0	0	0
seedMaxE0.seedMinPu0.0001.seedNoGU	47	0	0	0	1	1	1	0	1	0	1	1	0	0	1	1	0	0	0	0	0	0	1	0	1	0	0	1	1	0	0	0	0	1	0	1	1	1
seedMaxE0.seedMinPu0.2	17	0	0	0	1	1	1	0	0	0	0	0	0	0	0	1	0	0	0	0	0	0	1	0	1	0	1	1	0	0	0	0	1	0	0	0	0	0
seedMaxE0.seedMinPu0.2.seedNoGU	16	0	0	0	1	1	1	0	0	0	0	0	0	0	0	0	0	0	0	0	0	0	1	0	1	0	1	1	0	0	0	1	1	0	0	0	0	0
seedMaxE0.seedMinPu0.5	8	0	1	0	0	0	0	0	0	0	0	0	0	0	0	0	0	0	0	0	0	1	1	0	1	0	0	0	0	0	0	0	0	0	0	0	0	0
seedMaxE0.seedMinPu0.5.seedNoGU	7	0	1	0	0	0	0	0	0	0	0	0	0	0	0	0	0	0	0	0	0	0	1	0	1	0	0	0	0	0	0	0	0	0	0	0	0	0
seedMaxE0.seedMinPu0.seedNoGU	47	0	0	0	1	1	1	0	1	0	1	1	0	0	1	1	0	0	0	0	0	0	1	0	1	0	0	1	1	0	0	0	0	1	0	1	1	1
seedMaxE0.seedNoGU	47	0	0	0	1	1	1	0	1	0	1	1	0	0	1	1	0	0	0	0	0	0	1	0	1	0	0	1	1	0	0	0	0	1	0	1	1	1
seedMaxE-1	35	0	0	0	1	1	1	0	1	0	1	1	0	0	1	1	0	0	0	0	0	0	1	1	1	0	0	1	1	0	0	0	0	0	0	0	1	1
seedMaxE-1.seedMinPu0	35	0	0	0	1	1	1	0	1	0	1	1	0	0	1	1	0	0	0	0	0	0	1	1	1	0	0	1	1	0	0	0	0	0	0	0	1	1
seedMaxE-1.seedMinPu0.001	36	0	0	0	1	1	1	0	1	0	1	1	0	0	1	1	0	0	0	0	0	0	1	1	1	0	0	1	1	0	0	0	0	0	0	0	1	1
seedMaxE-1.seedMinPu0.0001	36	0	0	0	1	1	1	0	1	0	1	1	0	0	1	1	0	0	0	0	0	0	1	1	1	0	0	1	1	0	0	0	0	0	0	0	1	1
seedMaxE-1.seedMinPu0.01	39	0	0	0	1	1	1	0	1	0	1	1	0	0	1	1	0	0	0	0	0	0	1	1	1	0	0	1	1	0	0	0	0	0	0	0	1	1
seedMaxE-1.seedMinPu0.1	25	0	1	0	1	1	1	1	0	0	0	0	0	0	1	1	0	0	0	0	0	0	1	0	1	0	0	1	0	0	0	0	0	1	0	1	0	0
seedMaxE-1.seedMinPu0.1.seedNoGU	26	0	1	0	1	1	1	1	0	0	0	0	1	0	1	1	0	0	0	0	0	0	1	0	1	0	1	1	0	0	0	0	1	1	0	0	0	0
seedMaxE-1.seedMinPu0.0001.seedNoGU	47	0	0	0	1	1	1	0	1	0	1	1	0	0	1	1	0	0	0	0	0	0	1	0	1	0	0	1	1	0	0	0	0	1	0	1	1	1
seedMaxE-1.seedMinPu0.001.seedNoGU	49	0	0	0	1	1	1	0	1	0	1	1	0	0	1	1	0	0	0	0	0	0	1	0	1	0	0	1	1	0	0	0	0	1	0	1	1	1
seedMaxE-1.seedMinPu0.01.seedNoGU	48	0	1	0	1	1	1	0	1	0	1	1	0	0	1	1	0	0	0	0	0	0	1	0	1	0	0	1	1	0	0	1	0	1	0	1	1	1
seedMaxE-1.seedMinPu0.2	17	0	0	0	1	1	1	0	0	0	0	0	0	0	0	1	0	0	0	0	0	0	1	0	1	0	1	1	0	0	0	0	1	0	0	0	0	0
seedMaxE-1.seedMinPu0.2.seedNoGU	16	0	0	0	1	1	1	0	0	0	0	0	0	0	0	0	0	0	0	0	0	0	1	0	1	0	1	1	0	0	0	1	1	0	0	0	0	0
seedMaxE-1.seedMinPu0.5	8	0	1	0	0	0	0	0	0	0	0	0	0	0	0	0	0	0	0	0	0	1	1	0	1	0	0	0	0	0	0	0	0	0	0	0	0	0
seedMaxE-1.seedMinPu0.5.seedNoGU	7	0	1	0	0	0	0	0	0	0	0	0	0	0	0	0	0	0	0	0	0	0	1	0	1	0	0	0	0	0	0	0	0	0	0	0	0	0
seedMaxE-1.seedMinPu0.seedNoGU	47	0	0	0	1	1	1	0	1	0	1	1	0	0	1	1	0	0	0	0	0	0	1	0	1	0	0	1	1	0	0	0	0	1	0	1	1	1
seedMaxE-1.seedNoGU	47	0	0	0	1	1	1	0	1	0	1	1	0	0	1	1	0	0	0	0	0	0	1	0	1	0	0	1	1	0	0	0	0	1	0	1	1	1
seedMaxE-2	36	0	0	0	1	1	1	0	1	0	1	1	0	0	1	1	0	0	0	0	0	0	1	1	1	0	0	1	1	0	0	0	0	0	0	0	1	1
seedMaxE-2.seedMinPu0	36	0	0	0	1	1	1	0	1	0	1	1	0	0	1	1	0	0	0	0	0	0	1	1	1	0	0	1	1	0	0	0	0	0	0	0	1	1
seedMaxE-2.seedMinPu0.01	40	0	0	0	1	1	1	0	1	0	1	1	0	0	1	1	0	0	0	0	0	0	1	1	1	0	0	1	1	0	0	0	0	0	0	0	1	1
seedMaxE-2.seedMinPu0.1	26	0	1	0	1	1	1	1	0	0	0	0	0	0	1	1	0	0	0	0	0	0	1	0	1	0	1	1	0	0	0	0	0	1	0	1	0	0
seedMaxE-2.seedMinPu0.0001	36	0	0	0	1	1	1	0	1	0	1	1	0	0	1	1	0	0	0	0	0	0	1	1	1	0	0	1	1	0	0	0	0	0	0	0	1	1
seedMaxE-2.seedMinPu0.001	37	0	0	0	1	1	1	0	1	0	1	1	0	0	1	1	0	0	0	0	0	0	1	1	1	0	0	1	1	0	0	0	0	0	0	0	1	1
seedMaxE-2.seedMinPu0.0001.seedNoGU	47	0	0	0	1	1	1	0	1	0	1	1	0	0	1	1	0	0	0	0	0	0	1	0	1	0	0	1	1	0	0	0	0	1	0	1	1	1
seedMaxE-2.seedMinPu0.1.seedNoGU	26	0	1	0	1	1	1	1	0	0	0	0	1	0	1	1	0	0	0	0	0	0	1	0	1	0	1	1	0	0	0	0	1	1	0	0	0	0
seedMaxE-2.seedMinPu0.01.seedNoGU	48	0	1	0	1	1	1	0	1	0	1	1	0	0	1	1	0	0	0	0	0	0	1	0	1	0	0	1	1	0	0	1	0	1	0	1	1	1
seedMaxE-2.seedMinPu0.001.seedNoGU	49	0	0	0	1	1	1	0	1	0	1	1	0	0	1	1	0	0	0	0	0	0	1	0	1	0	0	1	1	0	0	0	0	1	0	1	1	1
seedMaxE-2.seedMinPu0.2	16	0	0	0	1	1	1	0	0	0	0	0	0	0	0	1	0	0	0	0	0	0	1	0	1	0	1	1	0	0	0	0	1	0	0	0	0	0
seedMaxE-2.seedMinPu0.2.seedNoGU	16	0	0	0	1	1	1	0	0	0	0	0	0	0	0	0	0	0	0	0	0	0	1	0	1	0	1	1	0	0	0	1	1	0	0	0	0	0
seedMaxE-2.seedMinPu0.5	6	0	0	0	0	0	0	0	0	0	0	0	0	0	0	0	0	0	0	0	0	0	1	0	1	0	0	0	0	0	0	0	0	0	0	0	0	0
seedMaxE-2.seedMinPu0.5.seedNoGU	5	0	0	0	0	0	0	0	0	0	0	0	0	0	0	0	0	0	0	0	0	0	1	0	1	0	0	0	0	0	0	0	0	0	0	0	0	0
seedMaxE-2.seedMinPu0.seedNoGU	47	0	0	0	1	1	1	0	1	0	1	1	0	0	1	1	0	0	0	0	0	0	1	0	1	0	0	1	1	0	0	0	0	1	0	1	1	1
seedMaxE-2.seedNoGU	47	0	0	0	1	1	1	0	1	0	1	1	0	0	1	1	0	0	0	0	0	0	1	0	1	0	0	1	1	0	0	0	0	1	0	1	1	1
seedMaxE-3	37	0	0	0	1	1	1	0	1	0	1	1	0	0	1	1	0	0	0	0	0	0	0	1	1	0	0	1	1	0	0	0	0	0	0	0	1	1
seedMaxE-3.seedMinPu0	37	0	0	0	1	1	1	0	1	0	1	1	0	0	1	1	0	0	0	0	0	0	0	1	1	0	0	1	1	0	0	0	0	0	0	0	1	1
seedMaxE-3.seedMinPu0.001	39	0	0	0	1	1	1	0	1	0	1	1	0	0	1	1	0	0	0	0	0	0	0	1	1	0	0	1	1	0	0	0	0	0	0	0	1	1
seedMaxE-3.seedMinPu0.0001	38	0	0	0	1	1	1	0	1	0	1	1	0	0	1	1	0	0	0	0	0	0	0	1	1	0	0	1	1	0	0	0	0	0	0	0	1	1
seedMaxE-3.seedMinPu0.1	24	0	1	0	1	1	1	1	0	0	0	0	0	0	0	1	0	0	0	0	0	0	0	0	1	0	1	1	0	0	0	0	0	1	0	1	0	0

	MicC-b2215	MicF-b0241	MicF-b0889	MicF-b0929	MicF-b3912	OxyS-b1892	OxyS-b2731	RprA-b1040	RprA-b1341	RprA-b2741	RybB-b0081	RybB-b0721	RybB-b0805	RybB-b1256	RybB-b2215	RybB-b2594	RyhB-b0118	RyhB-b0156	RyhB-b0288	RyhB-b0592	RyhB-b0683	RyhB-b0721	RyhB-b0723	RyhB-b0894	RyhB-b1107	RyhB-b1200	RyhB-b1452	RyhB-b1531	RyhB-b1588	RyhB-b1612	RyhB-b1656	RyhB-b1778	RyhB-b1981	RyhB-b2069
Target found among top-100 predictions for the sRNA for any tested parameterization	1	1	1	1	1	1	0	1	1	1	1	1	1	1	1	1	0	1	1	1	0	1	0	1	1	1	0	0	1	1	1	1	1	0
seedBP13	1	1	0	0	0	0	0	1	0	0	0	0	0	0	0	0	0	0	0	0	0	1	0	0	1	0	0	0	0	0	0	0	0	0
seedBP13.seedNoGU	1	0	0	0	0	0	0	1	0	0	0	0	0	0	0	0	0	0	0	0	0	0	0	0	0	0	0	0	0	0	0	0	0	0
seedBP14	1	0	0	0	0	0	0	1	0	0	0	0	0	0	0	0	0	0	0	0	0	0	0	0	0	0	0	0	0	0	0	0	0	0
seedBP14.seedNoGU	1	0	0	0	0	0	0	0	0	0	0	0	0	0	0	0	0	0	0	0	0	0	0	0	0	0	0	0	0	0	0	0	0	0
seedMaxE0	1	0	1	1	0	0	0	1	0	1	1	0	0	0	0	0	0	0	0	0	0	0	0	1	1	0	0	0	0	0	0	0	0	0
seedMaxE0.seedMinPu0	1	0	1	1	0	0	0	1	0	1	1	0	0	0	0	0	0	0	0	0	0	0	0	1	1	0	0	0	0	0	0	0	0	0
seedMaxE0.seedMinPu0.01	1	0	1	1	0	0	0	1	0	1	1	0	0	0	0	0	0	1	0	0	0	0	0	1	1	0	0	0	0	0	0	0	0	0
seedMaxE0.seedMinPu0.001	1	0	1	1	0	0	0	1	0	1	1	0	0	0	0	0	0	0	0	0	0	0	0	1	1	0	0	0	0	0	0	0	0	0
seedMaxE0.seedMinPu0.0001	1	0	1	1	0	0	0	1	0	1	1	0	0	0	0	0	0	0	0	0	0	0	0	1	1	0	0	0	0	0	0	0	0	0
seedMaxE0.seedMinPu0.1	1	1	1	1	0	0	0	0	0	0	0	0	0	0	0	0	0	0	0	0	0	0	0	0	1	0	0	0	0	0	0	1	0	0
seedMaxE0.seedMinPu0.01.seedNoGU	1	1	0	1	0	0	0	1	0	1	0	0	1	1	0	0	0	0	0	1	0	0	0	1	0	0	0	0	1	0	0	1	0	0
seedMaxE0.seedMinPu0.001.seedNoGU	1	1	0	1	0	0	0	1	0	1	0	0	1	0	1	0	0	1	0	1	0	1	0	1	0	0	0	0	1	0	0	0	0	0
seedMaxE0.seedMinPu0.1.seedNoGU	1	1	0	1	0	0	0	0	0	0	0	0	0	0	0	0	0	0	0	0	0	0	0	0	0	0	0	0	0	1	0	1	0	0
seedMaxE0.seedMinPu0.0001.seedNoGU	1	1	0	1	0	0	0	1	0	1	0	0	1	0	1	0	0	1	0	1	0	1	0	1	0	0	0	0	1	0	0	0	0	0
seedMaxE0.seedMinPu0.2	1	1	1	1	0	0	0	0	1	0	0	0	0	0	0	0	0	0	0	0	0	0	0	0	0	0	0	0	0	0	0	0	0	0
seedMaxE0.seedMinPu0.2.seedNoGU	1	1	0	1	0	0	0	0	0	0	0	0	0	0	0	0	0	0	0	0	0	0	0	0	0	0	0	0	0	0	0	0	0	0
seedMaxE0.seedMinPu0.5	1	0	0	1	0	0	0	0	0	0	0	0	0	0	0	0	0	0	0	0	0	0	0	0	0	0	0	0	0	0	0	0	0	0
seedMaxE0.seedMinPu0.5.seedNoGU	1	0	0	1	0	0	0	0	0	0	0	0	0	0	0	0	0	0	0	0	0	0	0	0	0	0	0	0	0	0	0	0	0	0
seedMaxE0.seedMinPu0.seedNoGU	1	1	0	1	0	0	0	1	0	1	0	0	1	0	1	0	0	1	0	1	0	1	0	1	0	0	0	0	1	0	0	0	0	0
seedMaxE0.seedNoGU	1	1	0	1	0	0	0	1	0	1	0	0	1	0	1	0	0	1	0	1	0	1	0	1	0	0	0	0	1	0	0	0	0	0
seedMaxE-1	1	0	1	1	0	0	0	1	0	1	1	0	0	0	0	0	0	0	0	0	0	0	0	1	1	0	0	0	0	0	0	0	0	0
seedMaxE-1.seedMinPu0	1	0	1	1	0	0	0	1	0	1	1	0	0	0	0	0	0	0	0	0	0	0	0	1	1	0	0	0	0	0	0	0	0	0
seedMaxE-1.seedMinPu0.001	1	0	1	1	0	0	0	1	0	1	1	0	0	0	0	0	0	0	0	0	0	0	0	1	1	0	0	0	0	0	0	0	0	0
seedMaxE-1.seedMinPu0.0001	1	0	1	1	0	0	0	1	0	1	1	0	0	0	0	0	0	0	0	0	0	0	0	1	1	0	0	0	0	0	0	0	0	0
seedMaxE-1.seedMinPu0.01	1	0	1	1	0	0	0	1	0	1	1	0	0	0	0	0	0	1	0	0	0	0	0	1	1	0	0	0	0	0	0	0	0	0
seedMaxE-1.seedMinPu0.1	1	1	1	1	0	0	0	0	0	0	0	0	0	0	0	0	0	0	0	0	0	0	0	0	1	0	0	0	0	0	0	1	0	0
seedMaxE-1.seedMinPu0.1.seedNoGU	1	1	0	1	0	0	0	0	0	0	0	0	0	0	0	0	0	0	0	0	0	0	0	0	0	0	0	0	0	1	0	1	0	0
seedMaxE-1.seedMinPu0.0001.seedNoGU	1	1	0	1	0	0	0	1	0	1	0	0	1	0	1	0	0	1	0	1	0	1	0	1	0	0	0	0	1	0	0	0	0	0
seedMaxE-1.seedMinPu0.001.seedNoGU	1	1	0	1	0	0	0	1	0	1	0	0	1	0	1	0	0	1	0	1	0	1	0	1	0	0	0	0	1	0	0	0	0	0
seedMaxE-1.seedMinPu0.01.seedNoGU	1	1	0	1	0	0	0	1	0	1	0	0	1	1	0	0	0	0	0	1	0	0	0	1	0	0	0	0	1	0	0	1	0	0
seedMaxE-1.seedMinPu0.2	1	1	1	1	0	0	0	0	1	0	0	0	0	0	0	0	0	0	0	0	0	0	0	0	0	0	0	0	0	0	0	0	0	0
seedMaxE-1.seedMinPu0.2.seedNoGU	1	1	0	1	0	0	0	0	0	0	0	0	0	0	0	0	0	0	0	0	0	0	0	0	0	0	0	0	0	0	0	0	0	0
seedMaxE-1.seedMinPu0.5	1	0	0	1	0	0	0	0	0	0	0	0	0	0	0	0	0	0	0	0	0	0	0	0	0	0	0	0	0	0	0	0	0	0
seedMaxE-1.seedMinPu0.5.seedNoGU	1	0	0	1	0	0	0	0	0	0	0	0	0	0	0	0	0	0	0	0	0	0	0	0	0	0	0	0	0	0	0	0	0	0
seedMaxE-1.seedMinPu0.seedNoGU	1	1	0	1	0	0	0	1	0	1	0	0	1	0	1	0	0	1	0	1	0	1	0	1	0	0	0	0	1	0	0	0	0	0
seedMaxE-1.seedNoGU	1	1	0	1	0	0	0	1	0	1	0	0	1	0	1	0	0	1	0	1	0	1	0	1	0	0	0	0	1	0	0	0	0	0
seedMaxE-2	1	0	1	1	0	0	0	1	0	1	1	0	0	0	0	0	0	0	0	0	0	0	0	1	1	0	0	0	0	0	0	0	0	0
seedMaxE-2.seedMinPu0	1	0	1	1	0	0	0	1	0	1	1	0	0	0	0	0	0	0	0	0	0	0	0	1	1	0	0	0	0	0	0	0	0	0
seedMaxE-2.seedMinPu0.01	1	0	1	1	0	0	0	1	0	1	1	0	0	0	0	0	0	1	0	0	0	0	0	1	1	0	0	0	0	0	0	0	0	0
seedMaxE-2.seedMinPu0.1	1	1	1	1	0	0	0	0	0	0	0	0	0	0	0	0	0	0	0	0	0	0	0	0	1	0	0	0	0	0	0	1	0	0
seedMaxE-2.seedMinPu0.0001	1	0	1	1	0	0	0	1	0	1	1	0	0	0	0	0	0	0	0	0	0	0	0	1	1	0	0	0	0	0	0	0	0	0
seedMaxE-2.seedMinPu0.001	1	0	1	1	0	0	0	1	0	1	1	0	0	0	0	0	0	0	0	0	0	0	0	1	1	0	0	0	0	0	0	0	0	0
seedMaxE-2.seedMinPu0.0001.seedNoGU	1	1	0	1	0	0	0	1	0	1	0	0	1	0	1	0	0	1	0	1	0	1	0	1	0	0	0	0	1	0	0	0	0	0
seedMaxE-2.seedMinPu0.1.seedNoGU	1	1	0	1	0	0	0	0	0	0	0	0	0	0	0	0	0	0	0	0	0	0	0	0	0	0	0	0	0	1	0	1	0	0
seedMaxE-2.seedMinPu0.01.seedNoGU	1	1	0	1	0	0	0	1	0	1	0	0	1	1	0	0	0	0	0	1	0	0	0	1	0	0	0	0	1	0	0	1	0	0
seedMaxE-2.seedMinPu0.001.seedNoGU	1	1	0	1	0	0	0	1	0	1	0	0	1	0	1	0	0	1	0	1	0	1	0	1	0	0	0	0	1	0	0	0	0	0
seedMaxE-2.seedMinPu0.2	1	1	0	1	0	0	0	0	1	0	0	0	0	0	0	0	0	0	0	0	0	0	0	0	0	0	0	0	0	0	0	0	0	0
seedMaxE-2.seedMinPu0.2.seedNoGU	1	1	0	1	0	0	0	0	0	0	0	0	0	0	0	0	0	0	0	0	0	0	0	0	0	0	0	0	0	0	0	0	0	0
seedMaxE-2.seedMinPu0.5	0	1	0	1	0	0	0	0	0	0	0	0	0	0	0	0	0	0	0	0	0	0	0	0	0	0	0	0	0	0	0	0	0	0
seedMaxE-2.seedMinPu0.5.seedNoGU	0	0	0	1	0	0	0	0	0	0	0	0	0	0	0	0	0	0	0	0	0	0	0	0	0	0	0	0	0	0	0	0	0	0
seedMaxE-2.seedMinPu0.seedNoGU	1	1	0	1	0	0	0	1	0	1	0	0	1	0	1	0	0	1	0	1	0	1	0	1	0	0	0	0	1	0	0	0	0	0
seedMaxE-2.seedNoGU	1	1	0	1	0	0	0	1	0	1	0	0	1	0	1	0	0	1	0	1	0	1	0	1	0	0	0	0	1	0	0	0	0	0
seedMaxE-3	1	0	1	1	0	0	0	1	0	1	1	0	0	0	1	0	0	0	0	0	0	0	0	1	1	0	0	0	0	0	0	0	0	0
seedMaxE-3.seedMinPu0	1	0	1	1	0	0	0	1	0	1	1	0	0	0	1	0	0	0	0	0	0	0	0	1	1	0	0	0	0	0	0	0	0	0
seedMaxE-3.seedMinPu0.001	1	0	1	1	0	0	0	1	0	1	1	0	0	0	1	0	0	0	0	0	0	0	0	1	1	0	0	0	0	0	0	0	0	0
seedMaxE-3.seedMinPu0.0001	1	0	1	1	0	0	0	1	0	1	1	0	0	0	1	0	0	0	0	0	0	0	0	1	1	0	0	0	0	0	0	0	0	0
seedMaxE-3.seedMinPu0.1	1	1	1	1	0	0	0	0	0	0	0	0	0	0	0	0	0	0	0	0	0	0	0	0	1	0	0	0	0	0	0	1	0	0

	RyhB-b2155	RyhB-b2206	RyhB-b2530	RyhB-b3365	RyhB-b3607	RyhB-b3942	RyhB-b4070	RyhB-b4122	SgrS-b1101	SgrS-b1817	SgrS-b2416	Spot42-b0039	Spot42-b0720	Spot42-b0721	Spot42-b0728	Spot42-b0757	Spot42-b1136	Spot42-b1302	Spot42-b1398	Spot42-b1761	Spot42-b1901	Spot42-b2221	Spot42-b2702	Spot42-b2715	Spot42-b2801	Spot42-b2802	Spot42-b3224	Spot42-b3566	Spot42-b3927	Spot42-b3962	Spot42-b4311
Target found among top-100 predictions for the sRNA for any tested parameterization	1	1	1	1	1	0	1	1	1	1	1	0	1	1	1	1	1	1	1	1	1	1	1	1	1	0	1	1	1	1	1
seedBP13	0	0	0	0	1	0	0	0	0	0	0	0	0	0	1	0	0	0	0	0	0	0	0	0	0	0	0	0	1	0	1
seedBP13.seedNoGU	0	0	0	0	0	0	0	0	0	0	0	0	0	0	0	0	0	0	0	0	0	0	0	0	0	0	0	0	0	0	0
seedBP14	0	0	0	0	0	0	0	0	0	0	0	0	0	0	0	0	0	0	0	0	0	0	0	0	0	0	0	0	0	0	1
seedBP14.seedNoGU	0	0	0	0	0	0	0	0	0	0	0	0	0	0	0	0	0	0	0	0	0	0	0	0	0	0	0	0	0	0	0
seedMaxE0	0	0	0	1	1	0	0	0	1	0	0	0	0	1	0	1	1	1	1	1	0	0	0	0	1	0	1	0	1	0	1
seedMaxE0.seedMinPu0	0	0	0	1	1	0	0	0	1	0	0	0	0	1	0	1	1	1	1	1	0	0	0	0	1	0	1	0	1	0	1
seedMaxE0.seedMinPu0.01	1	0	0	1	1	0	0	0	1	0	1	0	0	0	0	0	1	1	1	1	1	1	0	0	1	0	1	0	1	0	1
seedMaxE0.seedMinPu0.001	0	0	0	1	1	0	0	0	1	0	1	0	0	1	0	0	1	1	1	1	0	0	0	0	1	0	1	0	1	0	1
seedMaxE0.seedMinPu0.0001	0	0	0	1	1	0	0	0	1	0	0	0	0	1	0	1	1	1	1	1	0	0	0	0	1	0	1	0	1	0	1
seedMaxE0.seedMinPu0.1	0	0	0	0	0	0	1	0	0	0	0	0	0	0	0	1	1	0	0	0	1	0	0	0	1	0	0	0	1	1	0
seedMaxE0.seedMinPu0.01.seedNoGU	1	1	1	1	1	0	0	0	1	0	1	0	0	0	1	1	1	1	1	1	1	1	0	1	1	0	1	0	1	0	0
seedMaxE0.seedMinPu0.001.seedNoGU	1	1	1	1	1	0	0	1	1	0	1	0	0	0	1	1	1	1	1	1	1	1	0	1	1	0	1	1	1	0	0
seedMaxE0.seedMinPu0.1.seedNoGU	0	0	0	0	0	0	1	0	0	0	0	0	0	0	0	0	1	0	0	0	1	0	0	0	1	0	0	0	1	1	0
seedMaxE0.seedMinPu0.0001.seedNoGU	1	1	0	1	1	0	0	0	1	0	1	0	0	1	1	1	1	1	1	1	0	1	0	1	1	0	1	1	1	0	0
seedMaxE0.seedMinPu0.2	0	0	0	0	0	0	0	0	0	0	0	0	0	0	0	0	0	0	0	0	1	0	0	0	1	0	0	0	1	0	0
seedMaxE0.seedMinPu0.2.seedNoGU	0	0	0	0	0	0	0	0	0	0	0	0	1	0	0	0	0	0	0	0	1	0	0	0	1	0	0	0	1	0	0
seedMaxE0.seedMinPu0.5	0	0	0	1	0	0	0	0	0	0	0	0	1	0	0	0	0	0	0	0	0	0	0	0	0	0	0	0	0	0	0
seedMaxE0.seedMinPu0.5.seedNoGU	0	0	0	0	0	0	0	0	0	0	0	0	1	0	0	1	0	0	0	0	0	0	0	0	0	0	0	0	0	0	0
seedMaxE0.seedMinPu0.seedNoGU	1	1	0	1	1	0	0	0	1	0	1	0	0	1	1	1	1	1	1	1	0	1	0	1	1	0	1	1	1	0	0
seedMaxE0.seedNoGU	1	1	0	1	1	0	0	0	1	0	1	0	0	1	1	1	1	1	1	1	0	1	0	1	1	0	1	1	1	0	0
seedMaxE-1	0	0	0	1	1	0	0	0	1	0	0	0	0	1	0	0	1	1	1	1	0	0	0	0	1	0	1	0	1	0	1
seedMaxE-1.seedMinPu0	0	0	0	1	1	0	0	0	1	0	0	0	0	1	0	0	1	1	1	1	0	0	0	0	1	0	1	0	1	0	1
seedMaxE-1.seedMinPu0.001	0	0	0	1	1	0	0	0	1	0	1	0	0	1	0	0	1	1	1	1	0	0	0	0	1	0	1	0	1	0	1
seedMaxE-1.seedMinPu0.0001	0	0	0	1	1	0	0	0	1	0	1	0	0	1	0	0	1	1	1	1	0	0	0	0	1	0	1	0	1	0	1
seedMaxE-1.seedMinPu0.01	1	0	0	1	1	0	0	0	1	0	1	0	0	0	0	0	1	1	1	1	1	1	0	0	1	0	1	0	1	0	1
seedMaxE-1.seedMinPu0.1	0	0	0	0	0	0	1	0	0	0	0	0	0	0	0	1	1	0	0	0	1	0	0	0	1	0	0	0	1	1	0
seedMaxE-1.seedMinPu0.1.seedNoGU	0	0	0	0	0	0	1	0	0	1	0	0	0	0	0	0	1	0	0	0	1	0	0	0	1	0	0	0	1	1	0
seedMaxE-1.seedMinPu0.0001.seedNoGU	1	1	0	1	1	0	0	0	1	0	1	0	0	1	1	1	1	1	1	1	0	1	0	1	1	0	1	1	1	0	0
seedMaxE-1.seedMinPu0.001.seedNoGU	1	1	1	1	1	0	0	1	1	0	1	0	0	0	1	1	1	1	1	1	1	1	0	1	1	0	1	1	1	0	0
seedMaxE-1.seedMinPu0.01.seedNoGU	1	1	1	1	1	0	0	0	1	0	1	0	0	0	1	1	1	1	1	1	1	1	0	1	1	0	1	0	1	0	0
seedMaxE-1.seedMinPu0.2	0	0	0	0	0	0	0	0	0	0	0	0	0	0	0	0	0	0	0	0	1	0	0	0	1	0	0	0	1	0	0
seedMaxE-1.seedMinPu0.2.seedNoGU	0	0	0	0	0	0	0	0	0	0	0	0	1	0	0	0	0	0	0	0	1	0	0	0	1	0	0	0	1	0	0
seedMaxE-1.seedMinPu0.5	0	0	0	1	0	0	0	0	0	0	0	0	1	0	0	0	0	0	0	0	0	0	0	0	0	0	0	0	0	0	0
seedMaxE-1.seedMinPu0.5.seedNoGU	0	0	0	0	0	0	0	0	0	0	0	0	1	0	0	1	0	0	0	0	0	0	0	0	0	0	0	0	0	0	0
seedMaxE-1.seedMinPu0.seedNoGU	1	1	0	1	1	0	0	0	1	0	1	0	0	1	1	1	1	1	1	1	0	1	0	1	1	0	1	1	1	0	0
seedMaxE-1.seedNoGU	1	1	0	1	1	0	0	0	1	0	1	0	0	1	1	1	1	1	1	1	0	1	0	1	1	0	1	1	1	0	0
seedMaxE-2	0	0	0	1	1	0	0	0	1	0	1	0	0	1	0	0	1	1	1	1	0	0	0	0	1	0	1	0	1	0	1
seedMaxE-2.seedMinPu0	0	0	0	1	1	0	0	0	1	0	1	0	0	1	0	0	1	1	1	1	0	0	0	0	1	0	1	0	1	0	1
seedMaxE-2.seedMinPu0.01	1	0	0	1	1	0	0	0	1	0	1	0	0	0	0	1	1	1	1	1	1	1	0	0	1	0	1	0	1	0	1
seedMaxE-2.seedMinPu0.1	0	0	0	0	0	0	1	0	0	0	0	0	0	0	0	1	1	0	0	0	1	0	0	0	1	0	0	0	1	1	0
seedMaxE-2.seedMinPu0.0001	0	0	0	1	1	0	0	0	1	0	1	0	0	1	0	0	1	1	1	1	0	0	0	0	1	0	1	0	1	0	1
seedMaxE-2.seedMinPu0.001	0	0	0	1	1	0	0	0	1	0	1	0	0	1	0	0	1	1	1	1	0	1	0	0	1	0	1	0	1	0	1
seedMaxE-2.seedMinPu0.0001.seedNoGU	1	1	0	1	1	0	0	0	1	0	1	0	0	1	1	1	1	1	1	1	0	1	0	1	1	0	1	1	1	0	0
seedMaxE-2.seedMinPu0.1.seedNoGU	0	0	0	0	0	0	1	0	0	1	0	0	0	0	0	0	1	0	0	0	1	0	0	0	1	0	0	0	1	1	0
seedMaxE-2.seedMinPu0.01.seedNoGU	1	1	1	1	1	0	0	0	1	0	1	0	0	0	1	1	1	1	1	1	1	1	0	1	1	0	1	0	1	0	0
seedMaxE-2.seedMinPu0.001.seedNoGU	1	1	1	1	1	0	0	1	1	0	1	0	0	0	1	1	1	1	1	1	1	1	0	1	1	0	1	1	1	0	0
seedMaxE-2.seedMinPu0.2	0	0	0	0	0	0	0	0	0	0	0	0	0	0	0	0	0	0	0	0	1	0	0	0	1	0	0	0	1	0	0
seedMaxE-2.seedMinPu0.2.seedNoGU	0	0	0	0	0	0	0	0	0	0	0	0	1	0	0	0	0	0	0	0	1	0	0	0	1	0	0	0	1	0	0
seedMaxE-2.seedMinPu0.5	0	0	0	1	0	0	0	0	0	0	0	0	1	0	0	0	0	0	0	0	0	0	0	0	0	0	0	0	0	0	0
seedMaxE-2.seedMinPu0.5.seedNoGU	0	0	0	0	0	0	0	0	0	0	0	0	1	0	0	1	0	0	0	0	0	0	0	0	0	0	0	0	0	0	0
seedMaxE-2.seedMinPu0.seedNoGU	1	1	0	1	1	0	0	0	1	0	1	0	0	1	1	1	1	1	1	1	0	1	0	1	1	0	1	1	1	0	0
seedMaxE-2.seedNoGU	1	1	0	1	1	0	0	0	1	0	1	0	0	1	1	1	1	1	1	1	0	1	0	1	1	0	1	1	1	0	0
seedMaxE-3	0	0	0	1	1	0	0	0	1	0	1	0	0	1	0	0	1	1	1	1	0	1	0	0	1	0	1	0	1	0	1
seedMaxE-3.seedMinPu0	0	0	0	1	1	0	0	0	1	0	1	0	0	1	0	0	1	1	1	1	0	1	0	0	1	0	1	0	1	0	1
seedMaxE-3.seedMinPu0.001	0	0	0	1	1	0	0	0	1	0	1	0	0	1	0	1	1	1	1	1	1	1	0	0	1	0	1	0	1	0	1
seedMaxE-3.seedMinPu0.0001	0	0	0	1	1	0	0	0	1	0	1	0	0	1	0	0	1	1	1	1	1	1	0	0	1	0	1	0	1	0	1
seedMaxE-3.seedMinPu0.1	0	0	0	0	0	0	1	0	0	0	0	0	0	0	0	1	1	0	0	0	1	0	0	0	1	0	0	0	1	1	0

Echericha coli NC_000913

	sum	ArcZ-b1892	ArcZ-b2741	ArcZ-b3546	ChiX-b0619	ChiX-b0681	ChiX-b1737	CyaR-b0723	CyaR-b0814	CyaR-b1740	CyaR-b1824	CyaR-b2416	CyaR-b2666	CyaR-b2687	DsrA-b1237	DsrA-b2741	DsrA-b3251	FnrS-b0723	FnrS-b0755	FnrS-b0887	FnrS-b1107	FnrS-b1479	FnrS-b1531	FnrS-b1656	FnrS-b1841	FnrS-b2153	FnrS-b2303	FnrS-b2531	FnrS-b3829	FnrS-b3908	GcvB-b1040	GcvB-b1130	GcvB-b3089	GcvB-b4208	MicA-b0411	MicA-b0814	MicA-b0957	MicA-b1130	MicC-b2215	MicF-b0241	MicF-b0889	MicF-b0929	MicF-b3912	OxyS-b1892	OxyS-b2731	RprA-b1040	RprA-b1341	RprA-b2741	RybB-b0081	RybB-b0721	RybB-b0805	RybB-b1256
Target found among top-100 predictions for the sRNA for any tested parameterization	86	0	1	0	1	1	1	1	1	0	1	1	1	1	1	1	0	1	0	1	1	1	1	1	1	1	1	1	1	0	1	1	1	1	1	1	1	1	1	1	1	1	1	1	0	1	1	1	1	1	1	1
seedMaxE-3.seedMinPu0.01	39	0	0	0	1	1	1	0	1	0	1	1	0	0	1	1	0	0	0	0	0	0	0	1	1	0	0	1	1	0	0	0	0	0	0	0	1	1	1	0	1	1	0	0	0	1	0	1	1	0	0	0
seedMaxE-3.seedMinPu0.001.seedNoGU	47	0	0	0	1	1	1	0	1	0	1	1	0	0	1	1	0	0	0	0	0	0	0	0	1	0	0	1	1	0	0	0	0	1	0	1	1	1	1	1	0	1	0	0	0	1	0	1	0	0	1	0
seedMaxE-3.seedMinPu0.01.seedNoGU	48	0	1	0	1	1	1	0	1	0	1	1	0	0	1	1	0	0	0	1	0	0	0	0	1	0	0	1	1	0	0	1	1	1	0	1	1	1	1	1	0	1	0	0	0	1	0	1	0	0	1	1
seedMaxE-3.seedMinPu0.1.seedNoGU	24	0	1	0	1	1	1	1	0	0	0	0	1	0	0	1	0	0	0	0	0	0	0	0	1	0	1	1	0	0	0	0	1	1	0	0	0	0	1	1	0	1	0	0	0	0	0	0	0	0	0	0
seedMaxE-3.seedMinPu0.0001.seedNoGU	46	0	0	0	1	1	1	0	1	0	1	1	0	0	1	1	0	0	0	0	0	0	0	0	1	0	0	1	1	0	0	0	0	1	0	1	1	1	1	1	0	1	0	0	0	1	0	1	0	0	1	0
seedMaxE-3.seedMinPu0.2	14	0	0	0	1	1	1	0	0	0	0	0	0	0	0	1	0	0	0	0	0	0	0	0	1	0	1	1	0	0	0	0	1	0	0	0	0	0	0	1	0	1	0	0	0	0	1	0	0	0	0	0
seedMaxE-3.seedMinPu0.2.seedNoGU	13	0	0	0	1	1	1	0	0	0	0	0	0	0	0	0	0	0	0	0	0	0	0	0	1	0	1	1	0	0	0	1	1	0	0	0	0	0	0	1	0	1	0	0	0	0	0	0	0	0	0	0
seedMaxE-3.seedMinPu0.5	1	0	0	0	0	0	0	0	0	0	0	0	0	0	0	0	0	0	0	0	0	0	0	0	1	0	0	0	0	0	0	0	0	0	0	0	0	0	0	0	0	0	0	0	0	0	0	0	0	0	0	0
seedMaxE-3.seedMinPu0.5.seedNoGU	1	0	0	0	0	0	0	0	0	0	0	0	0	0	0	0	0	0	0	0	0	0	0	0	1	0	0	0	0	0	0	0	0	0	0	0	0	0	0	0	0	0	0	0	0	0	0	0	0	0	0	0
seedMaxE-3.seedMinPu0.seedNoGU	44	0	0	0	1	1	1	0	1	0	1	1	0	0	1	1	0	0	0	0	0	0	0	0	1	0	0	1	1	0	0	0	0	1	0	1	1	1	1	1	0	1	0	0	0	1	0	1	0	0	1	0
seedMaxE-3.seedNoGU	44	0	0	0	1	1	1	0	1	0	1	1	0	0	1	1	0	0	0	0	0	0	0	0	1	0	0	1	1	0	0	0	0	1	0	1	1	1	1	1	0	1	0	0	0	1	0	1	0	0	1	0
seedMaxE-4	38	0	0	0	1	1	1	0	1	0	1	1	0	0	1	1	0	0	0	0	0	0	0	1	1	0	0	1	1	0	0	0	0	0	0	0	0	1	1	0	1	1	0	0	0	1	0	0	1	0	0	0
seedMaxE-4.seedMinPu0	38	0	0	0	1	1	1	0	1	0	1	1	0	0	1	1	0	0	0	0	0	0	0	1	1	0	0	1	1	0	0	0	0	0	0	0	0	1	1	0	1	1	0	0	0	1	0	0	1	0	0	0
seedMaxE-4.seedMinPu0.01	40	0	0	0	1	1	1	0	1	0	1	1	0	0	1	1	0	0	0	0	0	0	0	1	1	0	0	1	1	0	0	0	0	1	0	1	0	1	1	0	1	1	0	0	0	1	0	0	1	0	0	0
seedMaxE-4.seedMinPu0.001	40	0	0	0	1	1	1	0	1	0	1	1	0	0	1	1	0	0	0	0	0	0	0	1	1	0	0	1	1	0	0	0	0	0	0	0	0	1	1	0	1	1	0	0	0	1	0	0	1	0	0	0
seedMaxE-4.seedMinPu0.1	27	0	1	0	1	1	1	1	0	0	0	0	1	0	0	1	0	0	0	0	0	0	0	0	1	0	1	1	0	0	0	0	0	1	0	0	0	0	1	1	1	1	0	0	0	0	1	0	0	0	0	0
seedMaxE-4.seedMinPu0.0001	40	0	0	0	1	1	1	0	1	0	1	1	0	0	1	1	0	0	0	0	0	0	0	1	1	0	0	1	1	0	0	0	0	0	0	0	0	1	1	0	1	1	0	0	0	1	0	0	1	0	0	0
seedMaxE-4.seedMinPu0.0001.seedNoGU	46	0	0	0	1	1	1	0	1	0	1	1	0	0	1	1	0	0	0	1	0	0	0	0	1	0	0	1	1	0	0	0	0	1	0	1	0	1	1	1	0	1	1	0	0	1	0	0	0	0	1	1
seedMaxE-4.seedMinPu0.1.seedNoGU	24	0	1	0	1	1	1	1	0	0	0	0	1	0	0	1	0	0	0	0	0	0	0	0	1	0	1	1	0	0	0	0	1	1	0	0	0	0	1	1	0	1	0	0	0	0	0	0	0	0	0	0
seedMaxE-4.seedMinPu0.001.seedNoGU	47	0	1	0	1	1	1	0	1	0	1	1	0	0	1	1	0	0	0	1	0	0	0	0	1	0	0	1	1	0	0	0	0	1	0	1	0	1	1	1	0	1	1	0	0	1	0	0	0	0	1	1
seedMaxE-4.seedMinPu0.01.seedNoGU	45	0	1	0	1	1	1	1	1	0	1	1	0	0	1	1	0	0	0	1	0	0	0	0	1	0	0	1	1	0	0	0	1	1	0	1	0	1	1	1	0	1	1	1	0	1	0	0	0	0	1	1
seedMaxE-4.seedMinPu0.2	15	0	0	0	1	1	1	0	0	0	0	0	0	0	0	1	0	0	0	0	0	0	0	0	1	0	1	1	0	0	0	0	1	0	0	0	0	0	0	1	0	1	0	0	0	0	1	0	0	0	0	0
seedMaxE-4.seedMinPu0.2.seedNoGU	13	0	0	0	1	1	1	0	0	0	0	0	0	0	0	0	0	0	0	0	0	0	0	0	1	0	1	1	0	0	0	1	1	0	0	0	0	0	0	1	0	1	0	0	0	0	0	0	0	0	0	0
seedMaxE-4.seedMinPu0.5	1	0	0	0	0	0	0	0	0	0	0	0	0	0	0	0	0	0	0	0	0	0	0	0	1	0	0	0	0	0	0	0	0	0	0	0	0	0	0	0	0	0	0	0	0	0	0	0	0	0	0	0
seedMaxE-4.seedMinPu0.5.seedNoGU	1	0	0	0	0	0	0	0	0	0	0	0	0	0	0	0	0	0	0	0	0	0	0	0	1	0	0	0	0	0	0	0	0	0	0	0	0	0	0	0	0	0	0	0	0	0	0	0	0	0	0	0
seedMaxE-4.seedMinPu0.seedNoGU	44	0	0	0	1	1	1	0	1	0	1	1	0	0	1	1	0	0	0	1	0	0	0	0	1	0	0	1	1	0	0	0	0	1	0	1	0	1	1	1	0	1	1	0	0	1	0	0	0	0	1	0
seedMaxE-4.seedNoGU	44	0	0	0	1	1	1	0	1	0	1	1	0	0	1	1	0	0	0	1	0	0	0	0	1	0	0	1	1	0	0	0	0	1	0	1	0	1	1	1	0	1	1	0	0	1	0	0	0	0	1	0
seedMaxE-5	43	0	0	0	1	1	1	0	1	0	1	1	0	0	1	0	0	0	0	0	0	0	0	1	1	0	0	1	0	0	0	0	0	1	0	0	0	1	1	1	1	1	0	0	0	1	0	0	1	0	0	0
seedMaxE-5.seedMinPu0	43	0	0	0	1	1	1	0	1	0	1	1	0	0	1	0	0	0	0	0	0	0	0	1	1	0	0	1	0	0	0	0	0	1	0	0	0	1	1	1	1	1	0	0	0	1	0	0	1	0	0	0
seedMaxE-5.seedMinPu0.1	25	0	1	0	1	1	1	1	0	0	0	0	1	0	0	0	0	0	0	0	0	0	0	0	1	0	1	0	0	0	0	0	1	1	0	0	0	0	0	1	1	1	0	0	0	0	1	0	0	0	0	0
seedMaxE-5.seedMinPu0.0001	43	0	0	0	1	1	1	0	1	0	1	1	0	0	1	0	0	0	0	0	0	0	0	1	1	0	0	1	0	0	0	0	0	1	0	0	0	1	1	1	1	1	0	0	0	1	0	0	1	0	0	0
seedMaxE-5.seedMinPu0.001	46	0	0	0	1	1	1	0	1	0	1	1	0	0	1	0	0	0	0	0	0	0	0	1	1	0	0	1	0	0	0	0	0	1	0	1	0	1	1	1	1	1	0	0	0	1	0	0	1	0	0	0
seedMaxE-5.seedMinPu0.01	42	0	1	0	1	1	1	0	1	0	1	1	0	0	1	0	0	0	0	0	0	0	0	1	1	0	0	1	0	0	0	0	0	1	0	1	0	1	1	1	1	1	0	0	0	1	0	0	1	0	0	0
seedMaxE-5.seedMinPu0.0001.seedNoGU	43	0	0	0	1	1	1	0	1	0	1	1	0	0	1	0	0	0	0	1	0	0	0	0	1	0	0	1	0	0	0	0	0	1	0	1	0	1	1	1	0	1	0	0	0	1	0	0	0	0	1	1
seedMaxE-5.seedMinPu0.01.seedNoGU	43	0	1	0	1	1	1	1	1	0	1	1	0	0	1	0	0	0	0	1	0	0	0	0	1	0	0	1	0	0	0	0	1	1	0	1	0	1	1	1	0	1	0	1	0	1	0	0	0	0	1	1
seedMaxE-5.seedMinPu0.1.seedNoGU	21	0	1	0	1	1	1	1	0	0	0	0	1	0	0	0	0	0	0	0	0	0	0	0	1	0	1	0	0	0	0	1	0	1	0	0	0	0	0	1	0	1	0	0	0	0	0	0	0	0	0	0
seedMaxE-5.seedMinPu0.001.seedNoGU	44	0	1	0	1	1	1	0	1	0	1	1	0	0	1	0	0	0	0	1	0	0	0	0	1	0	0	1	0	0	0	0	0	1	0	1	0	1	1	1	0	1	0	0	0	1	0	0	0	0	1	1
seedMaxE-5.seedMinPu0.2	12	0	0	0	1	1	1	0	0	0	0	0	0	0	0	0	0	0	0	0	0	0	0	0	1	0	1	0	0	0	0	0	1	0	0	0	0	0	0	1	0	1	0	0	0	0	1	0	0	0	0	0
seedMaxE-5.seedMinPu0.2.seedNoGU	11	0	0	0	1	1	1	0	0	0	0	0	0	0	0	0	0	0	0	0	0	0	0	0	1	0	1	0	0	0	0	1	0	0	0	0	0	0	0	1	0	1	0	0	0	0	0	0	0	0	0	0
seedMaxE-5.seedMinPu0.5	1	0	0	0	0	0	0	0	0	0	0	0	0	0	0	0	0	0	0	0	0	0	0	0	1	0	0	0	0	0	0	0	0	0	0	0	0	0	0	0	0	0	0	0	0	0	0	0	0	0	0	0
seedMaxE-5.seedMinPu0.5.seedNoGU	1	0	0	0	0	0	0	0	0	0	0	0	0	0	0	0	0	0	0	0	0	0	0	0	1	0	0	0	0	0	0	0	0	0	0	0	0	0	0	0	0	0	0	0	0	0	0	0	0	0	0	0
seedMaxE-5.seedMinPu0.seedNoGU	43	0	0	0	1	1	1	0	1	0	1	1	0	0	1	0	0	0	0	1	0	0	0	0	1	0	0	1	0	0	0	0	0	1	0	1	0	1	1	1	0	1	0	0	0	1	0	0	0	0	1	1
seedMaxE-5.seedNoGU	43	0	0	0	1	1	1	0	1	0	1	1	0	0	1	0	0	0	0	1	0	0	0	0	1	0	0	1	0	0	0	0	0	1	0	1	0	1	1	1	0	1	0	0	0	1	0	0	0	0	1	1
seedMaxE-6	40	0	0	0	1	1	1	0	1	0	1	1	0	0	1	0	0	0	0	1	0	0	0	1	1	0	0	1	0	0	0	0	0	1	0	1	0	1	1	0	1	1	0	0	0	1	0	0	1	0	0	0
seedMaxE-6.seedMinPu0	40	0	0	0	1	1	1	0	1	0	1	1	0	0	1	0	0	0	0	1	0	0	0	1	1	0	0	1	0	0	0	0	0	1	0	1	0	1	1	0	1	1	0	0	0	1	0	0	1	0	0	0
seedMaxE-6.seedMinPu0.01	37	0	0	0	1	1	1	0	1	0	1	1	0	0	1	0	0	0	0	1	0	0	0	1	1	0	0	1	0	0	0	0	0	1	0	1	0	1	0	0	1	1	0	0	0	0	0	0	1	0	1	0
seedMaxE-6.seedMinPu0.0001	41	0	0	0	1	1	1	0	1	0	1	1	0	0	1	0	0	0	0	1	0	0	0	1	1	0	0	1	0	0	0	0	0	1	0	1	0	1	1	0	1	1	0	0	0	1	0	0	1	0	1	0
seedMaxE-6.seedMinPu0.1	19	0	0	0	1	1	0	0	0	0	0	0	1	0	0	0	0	0	0	0	0	0	0	0	1	0	1	0	0	0	0	0	0	1	0	0	0	0	0	0	1	1	0	0	0	0	0	0	0	0	0	0
seedMaxE-6.seedMinPu0.001	41	0	0	0	1	1	1	0	1	0	1	1	0	0	1	0	0	0	0	1	0	0	0	1	1	0	0	1	0	0	0	0	0	1	0	1	0	1	1	0	1	1	0	0	0	1	0	0	1	0	1	0
seedMaxE-6.seedMinPu0.001.seedNoGU	44	0	0	0	1	1	1	1	1	0	1	1	0	0	1	0	0	0	0	1	0	0	0	0	1	1	1	1	0	0	0	0	0	1	0	1	0	1	1	0	0	1	0	1	0	1	0	0	0	0	1	1
seedMaxE-6.seedMinPu0.1.seedNoGU	17	0	0	0	1	1	0	0	0	0	0	0	1	0	0	0	0	0	0	0	0	0	0	0	1	0	1	0	0	0	0	1	0	1	0	0	0	0	0	0	0	1	0	0	0	0	0	0	0	0	0	0
seedMaxE-6.seedMinPu0.01.seedNoGU	40	0	0	0	1	1	1	1	1	0	1	1	0	0	1	0	0	0	0	1	0	0	0	0	1	1	1	1	0	0	0	0	0	1	0	1	0	1	0	0	0	1	0	1	0	0	0	0	0	0	1	1
seedMaxE-6.seedMinPu0.0001.seedNoGU	41	0	0	0	1	1	1	1	1	0	1	1	0	0	1	0	0	0	0	1	0	0	0	0	1	1	1	1	0	0	0	0	0	1	0	1	0	1	1	0	0	1	0	0	0	1	0	0	0	0	1	1
seedMaxE-6.seedMinPu0.2	9	0	0	0	1	0	0	0	0	0	0	0	0	0	0	0	0	0	0	0	0	0	0	0	1	0	1	0	0	0	0	1	1	0	0	0	0	0	0	0	0	1	0	0	0	0	0	0	0	0	0	0
seedMaxE-6.seedMinPu0.2.seedNoGU	8	0	0	0	1	0	0	0	0	0	0	0	0	0	0	0	0	0	0	0	0	0	0	0	1	0	1	0	0	0	0	1	0	0	0	0	0	0	0	0	0	1	0	0	0	0	0	0	0	0	0	0
seedMaxE-6.seedMinPu0.5	1	0	0	0	0	0	0	0	0	0	0	0	0	0	0	0	0	0	0	0	0	0	0	0	1	0	0	0	0	0	0	0	0	0	0	0	0	0	0	0	0	0	0	0	0	0	0	0	0	0	0	0
seedMaxE-6.seedMinPu0.5.seedNoGU	1	0	0	0	0	0	0	0	0	0	0	0	0	0	0	0	0	0	0	0	0	0	0	0	1	0	0	0	0	0	0	0	0	0	0	0	0	0	0	0	0	0	0	0	0	0	0	0	0	0	0	0

	RybB-b2215	RybB-b2594	RyhB-b0118	RyhB-b0156	RyhB-b0288	RyhB-b0592	RyhB-b0683	RyhB-b0721	RyhB-b0723	RyhB-b0894	RyhB-b1107	RyhB-b1200	RyhB-b1452	RyhB-b1531	RyhB-b1588	RyhB-b1612	RyhB-b1656	RyhB-b1778	RyhB-b1981	RyhB-b2069	RyhB-b2155	RyhB-b2206	RyhB-b2530	RyhB-b3365	RyhB-b3607	RyhB-b3942	RyhB-b4070	RyhB-b4122	SgrS-b1101	SgrS-b1817	SgrS-b2416	Spot42-b0039	Spot42-b0720	Spot42-b0721	Spot42-b0728	Spot42-b0757	Spot42-b1136	Spot42-b1302	Spot42-b1398	Spot42-b1761	Spot42-b1901	Spot42-b2221	Spot42-b2702	Spot42-b2715	Spot42-b2801	Spot42-b2802	Spot42-b3224	Spot42-b3566	Spot42-b3927	Spot42-b3962	Spot42-b4311
Target found among top-100 predictions for the sRNA for any tested parameterization	1	1	0	1	1	1	0	1	0	1	1	1	0	0	1	1	1	1	1	0	1	1	1	1	1	0	1	1	1	1	1	0	1	1	1	1	1	1	1	1	1	1	1	1	1	0	1	1	1	1	1
seedMaxE-3.seedMinPu0.01	0	0	0	1	0	0	0	0	0	1	1	0	0	0	0	0	0	0	0	0	1	0	0	1	1	0	0	0	1	0	1	0	0	0	0	1	1	1	1	1	1	1	0	0	1	0	1	0	1	0	1
seedMaxE-3.seedMinPu0.001.seedNoGU	1	0	0	1	0	1	0	1	0	1	0	0	0	0	0	0	0	0	0	0	1	1	1	1	1	0	0	1	1	0	1	0	1	0	1	1	1	1	1	1	1	1	0	1	1	0	1	0	1	0	0
seedMaxE-3.seedMinPu0.01.seedNoGU	0	0	0	0	0	1	0	0	0	1	0	0	0	0	0	0	0	1	0	0	1	1	1	1	1	0	0	0	1	0	1	0	0	0	1	1	1	1	1	1	1	1	0	1	1	0	1	0	1	0	0
seedMaxE-3.seedMinPu0.1.seedNoGU	0	0	0	0	0	0	0	0	0	0	0	0	0	0	0	1	0	1	0	0	0	0	0	0	0	0	1	0	0	1	0	0	0	0	0	0	1	0	0	0	1	0	0	0	1	0	0	0	1	1	0
seedMaxE-3.seedMinPu0.0001.seedNoGU	1	0	0	1	0	1	0	1	0	1	0	0	0	0	0	0	0	0	0	0	1	1	1	1	1	0	0	1	1	0	1	0	0	0	1	1	1	1	1	1	1	1	0	1	1	0	1	0	1	0	0
seedMaxE-3.seedMinPu0.2	0	0	0	0	0	0	0	0	0	0	0	0	0	0	0	0	0	0	0	0	0	0	0	0	0	0	0	0	0	0	0	0	0	0	0	0	0	0	0	0	1	0	0	0	1	0	0	0	1	0	0
seedMaxE-3.seedMinPu0.2.seedNoGU	0	0	0	0	0	0	0	0	0	0	0	0	0	0	0	0	0	0	0	0	0	0	0	0	0	0	0	0	0	0	0	0	0	0	0	0	0	0	0	0	1	0	0	0	1	0	0	0	1	0	0
seedMaxE-3.seedMinPu0.5	0	0	0	0	0	0	0	0	0	0	0	0	0	0	0	0	0	0	0	0	0	0	0	0	0	0	0	0	0	0	0	0	0	0	0	0	0	0	0	0	0	0	0	0	0	0	0	0	0	0	0
seedMaxE-3.seedMinPu0.5.seedNoGU	0	0	0	0	0	0	0	0	0	0	0	0	0	0	0	0	0	0	0	0	0	0	0	0	0	0	0	0	0	0	0	0	0	0	0	0	0	0	0	0	0	0	0	0	0	0	0	0	0	0	0
seedMaxE-3.seedMinPu0.seedNoGU	1	0	0	1	0	1	0	1	0	1	0	0	0	0	0	0	0	0	0	0	1	1	0	1	1	0	0	0	1	0	1	0	0	0	1	1	1	1	1	1	1	1	0	1	1	0	1	0	1	0	0
seedMaxE-3.seedNoGU	1	0	0	1	0	1	0	1	0	1	0	0	0	0	0	0	0	0	0	0	1	1	0	1	1	0	0	0	1	0	1	0	0	0	1	1	1	1	1	1	1	1	0	1	1	0	1	0	1	0	0
seedMaxE-4	1	0	0	0	0	0	0	0	0	1	1	0	0	0	0	0	0	0	0	0	1	0	0	1	1	0	0	0	1	0	1	0	0	1	0	1	1	1	1	1	1	1	0	0	1	0	1	0	1	0	1
seedMaxE-4.seedMinPu0	1	0	0	0	0	0	0	0	0	1	1	0	0	0	0	0	0	0	0	0	1	0	0	1	1	0	0	0	1	0	1	0	0	1	0	1	1	1	1	1	1	1	0	0	1	0	1	0	1	0	1
seedMaxE-4.seedMinPu0.01	0	0	0	0	0	0	0	0	0	1	1	0	0	0	0	0	0	0	0	0	1	1	0	1	1	0	0	0	1	0	1	0	0	0	1	1	1	1	1	0	1	1	0	1	1	0	1	0	1	0	1
seedMaxE-4.seedMinPu0.001	1	0	0	0	0	0	0	1	0	1	1	0	0	0	0	0	0	0	0	0	1	0	0	1	1	0	0	0	1	0	1	0	0	1	1	1	1	1	1	1	1	1	0	0	1	0	1	0	1	0	1
seedMaxE-4.seedMinPu0.1	0	0	0	0	0	1	0	0	0	0	1	0	0	0	0	1	0	1	0	0	0	0	0	0	0	0	1	0	0	0	0	0	0	0	0	1	1	0	0	0	1	0	0	0	1	0	0	0	1	1	0
seedMaxE-4.seedMinPu0.0001	1	0	0	0	0	0	0	1	0	1	1	0	0	0	0	0	0	0	0	0	1	0	0	1	1	0	0	0	1	0	1	0	0	1	1	1	1	1	1	1	1	1	0	0	1	0	1	0	1	0	1
seedMaxE-4.seedMinPu0.0001.seedNoGU	1	0	0	0	0	0	0	1	0	1	0	0	0	0	0	0	0	1	0	0	1	1	1	1	1	0	0	1	1	0	1	0	1	0	1	0	1	1	1	1	1	1	0	1	1	0	1	0	1	0	0
seedMaxE-4.seedMinPu0.1.seedNoGU	0	0	0	0	0	0	0	0	0	0	0	0	0	0	0	1	0	1	0	0	0	0	0	0	0	0	1	0	0	1	0	0	0	0	0	0	1	0	0	0	1	0	0	0	1	0	0	0	1	1	0
seedMaxE-4.seedMinPu0.001.seedNoGU	1	0	0	0	0	0	0	1	0	1	0	0	0	0	0	0	0	1	0	0	1	1	1	1	1	0	0	1	1	0	1	0	1	0	1	0	1	1	1	1	1	1	0	1	1	0	1	0	1	0	0
seedMaxE-4.seedMinPu0.01.seedNoGU	0	0	0	0	0	0	0	0	0	1	0	0	0	0	0	0	0	1	0	0	1	1	1	1	1	0	0	0	1	0	1	0	0	0	1	0	1	1	1	0	1	1	0	1	1	0	1	0	1	0	0
seedMaxE-4.seedMinPu0.2	0	1	0	0	0	0	0	0	0	0	0	0	0	0	0	0	0	0	0	0	0	0	0	0	0	0	0	0	0	0	0	0	0	0	0	0	0	0	0	0	1	0	0	0	1	0	0	0	1	0	0
seedMaxE-4.seedMinPu0.2.seedNoGU	0	0	0	0	0	0	0	0	0	0	0	0	0	0	0	0	0	0	0	0	0	0	0	0	0	0	0	0	0	0	0	0	0	0	0	0	0	0	0	0	1	0	0	0	1	0	0	0	1	0	0
seedMaxE-4.seedMinPu0.5	0	0	0	0	0	0	0	0	0	0	0	0	0	0	0	0	0	0	0	0	0	0	0	0	0	0	0	0	0	0	0	0	0	0	0	0	0	0	0	0	0	0	0	0	0	0	0	0	0	0	0
seedMaxE-4.seedMinPu0.5.seedNoGU	0	0	0	0	0	0	0	0	0	0	0	0	0	0	0	0	0	0	0	0	0	0	0	0	0	0	0	0	0	0	0	0	0	0	0	0	0	0	0	0	0	0	0	0	0	0	0	0	0	0	0
seedMaxE-4.seedMinPu0.seedNoGU	1	0	0	0	0	0	0	1	0	1	0	0	0	0	0	0	0	0	0	0	1	1	1	1	1	0	0	1	1	0	1	0	1	0	1	0	1	1	1	1	1	1	0	1	1	0	1	0	1	0	0
seedMaxE-4.seedNoGU	1	0	0	0	0	0	0	1	0	1	0	0	0	0	0	0	0	0	0	0	1	1	1	1	1	0	0	1	1	0	1	0	1	0	1	0	1	1	1	1	1	1	0	1	1	0	1	0	1	0	0
seedMaxE-5	1	0	0	0	0	0	0	1	0	1	1	0	0	0	1	0	0	0	0	0	1	1	0	1	1	0	0	0	1	0	1	0	0	1	1	1	1	1	1	1	1	1	0	1	1	0	1	0	1	0	1
seedMaxE-5.seedMinPu0	1	0	0	0	0	0	0	1	0	1	1	0	0	0	1	0	0	0	0	0	1	1	0	1	1	0	0	0	1	0	1	0	0	1	1	1	1	1	1	1	1	1	0	1	1	0	1	0	1	0	1
seedMaxE-5.seedMinPu0.1	0	0	0	0	0	0	0	0	0	0	1	0	0	0	0	1	0	1	0	0	0	0	0	0	0	0	1	0	0	1	0	0	0	0	0	1	1	0	0	0	1	0	0	0	1	0	0	0	1	1	0
seedMaxE-5.seedMinPu0.0001	1	0	0	0	0	0	0	1	0	1	1	0	0	0	1	0	0	0	0	0	1	1	0	1	1	0	0	0	1	0	1	0	0	1	1	1	1	1	1	1	1	1	0	1	1	0	1	0	1	0	1
seedMaxE-5.seedMinPu0.001	1	0	0	0	0	0	0	1	0	1	1	0	0	0	1	0	0	0	0	0	1	1	1	1	1	0	0	1	1	0	1	0	0	1	1	1	1	1	1	1	1	1	0	1	1	0	1	0	1	0	1
seedMaxE-5.seedMinPu0.01	0	0	0	0	0	0	0	0	0	1	1	0	0	0	0	0	0	1	0	0	1	1	1	1	1	0	0	0	1	0	1	0	0	0	1	1	1	1	1	0	1	1	0	1	1	0	1	0	1	0	1
seedMaxE-5.seedMinPu0.0001.seedNoGU	1	0	0	0	0	0	0	1	0	1	0	0	0	0	0	0	0	1	0	0	1	1	1	1	1	0	0	1	1	0	1	0	1	0	1	0	1	1	1	1	1	1	0	1	1	0	1	0	1	0	0
seedMaxE-5.seedMinPu0.01.seedNoGU	0	0	0	1	0	0	0	0	0	1	0	0	0	0	0	0	0	1	0	0	1	1	1	1	1	0	0	0	1	0	1	0	0	0	1	0	1	1	1	0	1	1	0	1	1	0	1	0	1	0	0
seedMaxE-5.seedMinPu0.1.seedNoGU	0	0	0	0	0	0	0	0	0	0	0	0	0	0	0	1	0	1	0	0	0	0	0	0	0	0	1	0	0	1	0	0	0	0	0	0	1	0	0	0	1	0	0	0	1	0	0	0	1	1	0
seedMaxE-5.seedMinPu0.001.seedNoGU	1	0	0	0	0	0	0	1	0	1	0	0	0	0	0	0	0	1	0	0	1	1	1	1	1	0	0	1	1	0	1	0	1	0	1	0	1	1	1	1	1	1	0	1	1	0	1	0	1	0	0
seedMaxE-5.seedMinPu0.2	0	0	0	0	0	0	0	0	0	0	0	0	0	0	0	0	0	0	0	0	0	0	0	0	0	0	0	0	0	0	0	0	0	0	0	0	0	0	0	0	1	0	0	0	1	0	0	0	1	0	0
seedMaxE-5.seedMinPu0.2.seedNoGU	0	0	0	0	0	0	0	0	0	0	0	0	0	0	0	0	0	0	0	0	0	0	0	0	0	0	0	0	0	0	0	0	0	0	0	0	0	0	0	0	1	0	0	0	1	0	0	0	1	0	0
seedMaxE-5.seedMinPu0.5	0	0	0	0	0	0	0	0	0	0	0	0	0	0	0	0	0	0	0	0	0	0	0	0	0	0	0	0	0	0	0	0	0	0	0	0	0	0	0	0	0	0	0	0	0	0	0	0	0	0	0
seedMaxE-5.seedMinPu0.5.seedNoGU	0	0	0	0	0	0	0	0	0	0	0	0	0	0	0	0	0	0	0	0	0	0	0	0	0	0	0	0	0	0	0	0	0	0	0	0	0	0	0	0	0	0	0	0	0	0	0	0	0	0	0
seedMaxE-5.seedMinPu0.seedNoGU	1	0	0	0	0	0	0	1	0	1	0	0	0	0	0	0	0	1	0	0	1	1	1	1	1	0	0	1	1	0	1	0	1	0	1	0	1	1	1	1	1	1	0	1	1	0	1	0	1	0	0
seedMaxE-5.seedNoGU	1	0	0	0	0	0	0	1	0	1	0	0	0	0	0	0	0	1	0	0	1	1	1	1	1	0	0	1	1	0	1	0	1	0	1	0	1	1	1	1	1	1	0	1	1	0	1	0	1	0	0
seedMaxE-6	1	0	0	0	0	0	0	0	0	1	1	0	0	0	0	0	0	1	0	0	1	1	1	1	0	0	0	0	0	0	1	0	1	0	1	1	1	1	1	0	1	1	0	1	1	0	1	0	1	0	0
seedMaxE-6.seedMinPu0	1	0	0	0	0	0	0	0	0	1	1	0	0	0	0	0	0	1	0	0	1	1	1	1	0	0	0	0	0	0	1	0	1	0	1	1	1	1	1	0	1	1	0	1	1	0	1	0	1	0	0
seedMaxE-6.seedMinPu0.01	0	0	0	0	0	0	0	0	0	1	1	0	0	0	0	0	0	1	0	0	1	1	1	1	0	0	0	0	0	0	1	0	0	0	1	1	1	1	1	0	1	1	0	1	1	0	1	0	1	0	0
seedMaxE-6.seedMinPu0.0001	1	0	0	0	0	0	0	0	0	1	1	0	0	0	0	0	0	1	0	0	1	1	1	1	0	0	0	0	0	0	1	0	1	0	1	1	1	1	1	0	1	1	0	1	1	0	1	0	1	0	0
seedMaxE-6.seedMinPu0.1	0	0	0	0	0	0	0	0	0	0	1	0	0	0	0	1	0	1	0	0	0	0	0	0	0	0	1	0	0	1	0	0	0	0	0	1	1	0	0	0	1	0	0	0	1	0	0	0	1	1	0
seedMaxE-6.seedMinPu0.001	1	0	0	0	0	0	0	0	0	1	1	0	0	0	0	0	0	1	0	0	1	1	1	1	0	0	0	0	0	0	1	0	1	0	1	1	1	1	1	0	1	1	0	1	1	0	1	0	1	0	0
seedMaxE-6.seedMinPu0.001.seedNoGU	1	0	0	0	1	0	0	0	0	1	0	0	0	0	0	0	0	1	0	0	1	1	1	1	0	0	1	0	0	0	1	0	1	0	1	0	1	1	1	0	1	1	0	1	1	0	1	0	1	1	0
seedMaxE-6.seedMinPu0.1.seedNoGU	0	0	0	0	0	0	0	0	0	0	0	0	0	0	0	1	0	1	0	0	0	0	0	0	0	0	1	0	0	1	0	0	0	0	0	0	1	0	0	0	1	0	0	0	1	0	0	0	1	1	0
seedMaxE-6.seedMinPu0.01.seedNoGU	0	0	0	0	1	0	0	0	0	1	0	0	0	0	0	0	0	1	0	0	1	1	1	1	0	0	1	0	0	0	1	0	0	0	1	0	1	1	1	0	1	1	0	1	1	0	1	0	1	1	0
seedMaxE-6.seedMinPu0.0001.seedNoGU	1	0	0	0	0	0	0	0	0	1	0	0	0	0	0	0	0	1	0	0	1	1	1	1	0	0	0	0	0	0	1	0	1	0	1	0	1	1	1	0	1	1	0	1	1	0	1	0	1	1	0
seedMaxE-6.seedMinPu0.2	0	0	0	0	0	0	0	0	0	0	0	0	0	0	0	0	0	0	0	0	0	0	0	0	0	0	0	0	0	0	0	0	0	0	0	0	0	0	0	0	1	0	0	0	1	0	0	0	1	0	0
seedMaxE-6.seedMinPu0.2.seedNoGU	0	0	0	0	0	0	0	0	0	0	0	0	0	0	0	0	0	0	0	0	0	0	0	0	0	0	0	0	0	0	0	0	0	0	0	0	0	0	0	0	1	0	0	0	1	0	0	0	1	0	0
seedMaxE-6.seedMinPu0.5	0	0	0	0	0	0	0	0	0	0	0	0	0	0	0	0	0	0	0	0	0	0	0	0	0	0	0	0	0	0	0	0	0	0	0	0	0	0	0	0	0	0	0	0	0	0	0	0	0	0	0
seedMaxE-6.seedMinPu0.5.seedNoGU	0	0	0	0	0	0	0	0	0	0	0	0	0	0	0	0	0	0	0	0	0	0	0	0	0	0	0	0	0	0	0	0	0	0	0	0	0	0	0	0	0	0	0	0	0	0	0	0	0	0	0

Echericha coli NC_000913	sum	ArcZ-b1892	ArcZ-b2741	ArcZ-b3546	ChiX-b0619	ChiX-b0681	ChiX-b1737	CyaR-b0723	CyaR-b0814	CyaR-b1740	CyaR-b1824	CyaR-b2416	CyaR-b2666	CyaR-b2687	DsrA-b1237	DsrA-b2741	DsrA-b3251	FnrS-b0723	FnrS-b0755	FnrS-b0887	FnrS-b1107	FnrS-b1479	FnrS-b1531	FnrS-b1656	FnrS-b1841	FnrS-b2153	FnrS-b2303	FnrS-b2531	FnrS-b3829	FnrS-b3908	GcvB-b1040	GcvB-b1130	GcvB-b3089	GcvB-b4208	MicA-b0411	MicA-b0814	MicA-b0957	MicA-b1130	MicC-b2215	MicF-b0241	MicF-b0889	MicF-b0929	MicF-b3912	OxyS-b1892	OxyS-b2731	RprA-b1040	RprA-b1341	RprA-b2741	RybB-b0081	RybB-b0721	RybB-b0805	RybB-b1256	RybB-b2215	RybB-b2594	RyhB-b0118	RyhB-b0156	RyhB-b0288	RyhB-b0592	RyhB-b0683	RyhB-b0721	RyhB-b0723	RyhB-b0894	RyhB-b1107	RyhB-b1200	RyhB-b1452	RyhB-b1531	RyhB-b1588	RyhB-b1612	RyhB-b1656	RyhB-b1778	RyhB-b1981	RyhB-b2069	RyhB-b2155	RyhB-b2206	RyhB-b2530	RyhB-b3365	RyhB-b3607	RyhB-b3942	RyhB-b4070	RyhB-b4122	SgrS-b1101	SgrS-b1817	SgrS-b2416	Spot42-b0039	Spot42-b0720	Spot42-b0721	Spot42-b0728	Spot42-b0757	Spot42-b1136	Spot42-b1302	Spot42-b1398	Spot42-b1761	Spot42-b1901	Spot42-b2221	Spot42-b2702	Spot42-b2715	Spot42-b2801	Spot42-b2802	Spot42-b3224	Spot42-b3566	Spot42-b3927	Spot42-b3962	Spot42-b4311
Target found among top-100 predictions for the sRNA for any tested parameterization	86	0	1	0	1	1	1	1	1	0	1	1	1	1	1	1	0	1	0	1	1	1	1	1	1	1	1	1	1	0	1	1	1	1	1	1	1	1	1	1	1	1	1	1	0	1	1	1	1	1	1	1	1	1	0	1	1	1	0	1	0	1	1	1	0	0	1	1	1	1	1	0	1	1	1	1	1	0	1	1	1	1	1	0	1	1	1	1	1	1	1	1	1	1	1	1	1	0	1	1	1	1	1
seedMaxE-6.seedMinPu0.seedNoGU	41	0	0	0	1	1	1	1	1	0	1	1	0	0	1	0	0	0	0	1	0	0	0	0	1	1	1	1	0	0	0	0	0	1	0	1	0	1	1	0	0	1	0	0	0	1	0	0	0	0	1	1	1	0	0	0	0	0	0	0	0	1	0	0	0	0	0	0	0	1	0	0	1	1	1	1	0	0	0	0	0	0	1	0	1	0	1	0	1	1	1	0	1	1	0	1	1	0	1	0	1	1	0
seedMaxE-6.seedNoGU	41	0	0	0	1	1	1	1	1	0	1	1	0	0	1	0	0	0	0	1	0	0	0	0	1	1	1	1	0	0	0	0	0	1	0	1	0	1	1	0	0	1	0	0	0	1	0	0	0	0	1	1	1	0	0	0	0	0	0	0	0	1	0	0	0	0	0	0	0	1	0	0	1	1	1	1	0	0	0	0	0	0	1	0	1	0	1	0	1	1	1	0	1	1	0	1	1	0	1	0	1	1	0
seedMaxE-7	28	0	0	0	0	0	0	0	0	0	0	1	0	0	0	0	0	0	0	1	0	0	0	1	0	1	1	0	0	0	0	0	0	1	0	0	0	1	1	0	1	0	0	1	0	1	0	0	0	0	1	1	1	0	0	0	0	0	0	0	0	1	1	0	0	0	0	0	0	1	0	0	1	0	1	0	0	0	0	0	0	0	0	0	0	0	0	0	1	1	1	0	1	1	0	1	1	0	1	0	1	0	0
seedMaxE-7.seedMinPu0	28	0	0	0	0	0	0	0	0	0	0	1	0	0	0	0	0	0	0	1	0	0	0	1	0	1	1	0	0	0	0	0	0	1	0	0	0	1	1	0	1	0	0	1	0	1	0	0	0	0	1	1	1	0	0	0	0	0	0	0	0	1	1	0	0	0	0	0	0	1	0	0	1	0	1	0	0	0	0	0	0	0	0	0	0	0	0	0	1	1	1	0	1	1	0	1	1	0	1	0	1	0	0
seedMaxE-7.seedMinPu0.01	26	0	0	0	0	0	0	0	0	0	0	1	0	0	0	0	0	0	0	1	0	0	0	1	0	1	1	0	0	0	0	0	0	1	0	0	0	1	0	0	1	0	0	1	0	0	0	0	0	0	1	1	0	0	0	0	0	0	0	0	0	1	1	0	0	0	0	0	0	1	0	0	1	0	1	0	1	0	0	0	0	0	0	0	0	0	0	0	1	1	1	0	1	1	0	1	1	0	1	0	1	0	0
seedMaxE-7.seedMinPu0.001	29	0	0	0	0	0	0	0	0	0	0	1	0	0	0	0	0	0	0	1	0	0	0	1	0	1	1	0	0	0	0	0	0	1	0	0	0	1	1	0	1	0	0	1	0	1	0	0	0	0	1	1	1	0	0	0	0	0	0	0	0	1	1	0	0	0	0	0	0	1	0	0	1	0	1	0	1	0	0	0	0	0	0	0	0	0	0	0	1	1	1	0	1	1	0	1	1	0	1	0	1	0	0
seedMaxE-7.seedMinPu0.0001	29	0	0	0	0	0	0	0	0	0	0	1	0	0	0	0	0	0	0	1	0	0	0	1	0	1	1	0	0	0	0	0	0	1	0	0	0	1	1	0	1	0	0	1	0	1	0	0	0	0	1	1	1	0	0	0	0	0	0	0	0	1	1	0	0	0	0	0	0	1	0	0	1	0	1	0	1	0	0	0	0	0	0	0	0	0	0	0	1	1	1	0	1	1	0	1	1	0	1	0	1	0	0
seedMaxE-7.seedMinPu0.1	11	0	0	0	0	0	0	0	0	0	0	0	1	0	0	0	0	0	0	0	0	0	0	0	0	0	1	0	0	0	0	1	0	1	0	0	0	0	0	0	1	0	0	0	0	0	0	0	0	0	0	0	0	0	0	0	0	0	0	0	0	0	1	0	0	0	0	0	0	1	0	0	0	0	0	0	0	0	0	0	0	0	0	0	0	0	0	0	1	0	0	0	1	0	0	0	1	0	0	0	1	0	0
seedMaxE-7.seedMinPu0.1.seedNoGU	9	0	0	0	0	0	0	0	0	0	0	0	1	0	0	0	0	0	0	0	0	0	0	0	0	0	1	0	0	0	0	1	0	1	0	0	0	0	0	0	0	0	0	0	0	0	0	0	0	0	0	0	0	0	0	0	0	0	0	0	0	0	0	0	0	0	0	0	0	1	0	0	0	0	0	0	0	0	0	0	0	0	0	0	0	0	0	0	1	0	0	0	1	0	0	0	1	0	0	0	1	0	0
seedMaxE-7.seedMinPu0.001.seedNoGU	30	0	0	0	0	0	0	0	0	0	0	1	1	1	0	0	0	0	0	1	0	0	0	0	0	1	1	1	0	0	0	1	0	1	0	0	0	1	1	0	0	0	0	1	0	1	0	0	0	0	1	1	1	0	0	0	0	0	0	0	0	1	0	0	0	0	0	0	0	1	0	0	1	0	1	0	1	0	0	0	0	0	0	0	0	0	0	0	1	1	1	0	1	1	0	1	1	0	1	0	1	0	0
seedMaxE-7.seedMinPu0.01.seedNoGU	26	0	0	0	0	0	0	0	0	0	0	1	1	0	0	0	0	0	0	1	0	0	0	0	0	1	1	1	0	0	0	1	0	1	0	0	0	1	0	0	0	0	0	1	0	0	0	0	0	0	1	1	0	0	0	0	0	0	0	0	0	1	0	0	0	0	0	0	0	1	0	0	1	0	1	0	1	0	0	0	0	0	0	0	0	0	0	0	1	1	1	0	1	1	0	1	1	0	1	0	1	0	0
seedMaxE-7.seedMinPu0.0001.seedNoGU	30	0	0	0	0	0	0	0	0	0	0	1	1	1	0	0	0	0	0	1	0	0	0	0	0	1	1	1	0	0	0	1	0	1	0	0	0	1	1	0	0	0	0	1	0	1	0	0	0	0	1	1	1	0	0	0	0	0	0	0	0	1	0	0	0	0	0	0	0	1	0	0	1	0	1	0	1	0	0	0	0	0	0	0	0	0	0	0	1	1	1	0	1	1	0	1	1	0	1	0	1	0	0
seedMaxE-7.seedMinPu0.2	5	0	0	0	0	0	0	0	0	0	0	0	0	0	0	0	0	0	0	0	0	0	0	0	0	0	1	0	0	0	0	1	0	0	0	0	0	0	0	0	0	0	0	0	0	0	0	0	0	0	0	0	0	0	0	0	0	0	0	0	0	0	0	0	0	0	0	0	0	0	0	0	0	0	0	0	0	0	0	0	0	0	0	0	0	0	0	0	0	0	0	0	1	0	0	0	1	0	0	0	1	0	0
seedMaxE-7.seedMinPu0.2.seedNoGU	5	0	0	0	0	0	0	0	0	0	0	0	0	0	0	0	0	0	0	0	0	0	0	0	0	0	1	0	0	0	0	1	0	0	0	0	0	0	0	0	0	0	0	0	0	0	0	0	0	0	0	0	0	0	0	0	0	0	0	0	0	0	0	0	0	0	0	0	0	0	0	0	0	0	0	0	0	0	0	0	0	0	0	0	0	0	0	0	0	0	0	0	1	0	0	0	1	0	0	0	1	0	0
seedMaxE-7.seedMinPu0.5	0	0	0	0	0	0	0	0	0	0	0	0	0	0	0	0	0	0	0	0	0	0	0	0	0	0	0	0	0	0	0	0	0	0	0	0	0	0	0	0	0	0	0	0	0	0	0	0	0	0	0	0	0	0	0	0	0	0	0	0	0	0	0	0	0	0	0	0	0	0	0	0	0	0	0	0	0	0	0	0	0	0	0	0	0	0	0	0	0	0	0	0	0	0	0	0	0	0	0	0	0	0	0
seedMaxE-7.seedMinPu0.5.seedNoGU	0	0	0	0	0	0	0	0	0	0	0	0	0	0	0	0	0	0	0	0	0	0	0	0	0	0	0	0	0	0	0	0	0	0	0	0	0	0	0	0	0	0	0	0	0	0	0	0	0	0	0	0	0	0	0	0	0	0	0	0	0	0	0	0	0	0	0	0	0	0	0	0	0	0	0	0	0	0	0	0	0	0	0	0	0	0	0	0	0	0	0	0	0	0	0	0	0	0	0	0	0	0	0
seedMaxE-7.seedMinPu0.seedNoGU	30	0	0	0	0	0	0	0	0	0	0	1	1	1	0	0	0	0	0	1	0	0	0	0	0	1	1	1	0	0	0	1	0	1	0	0	0	1	1	0	0	0	0	1	0	1	0	0	0	0	1	1	1	0	0	0	0	0	0	0	0	1	0	0	0	0	0	0	0	1	0	0	1	0	1	0	1	0	0	0	0	0	0	0	0	0	0	0	1	1	1	0	1	1	0	1	1	0	1	0	1	0	0
seedMaxE-7.seedNoGU	30	0	0	0	0	0	0	0	0	0	0	1	1	1	0	0	0	0	0	1	0	0	0	0	0	1	1	1	0	0	0	1	0	1	0	0	0	1	1	0	0	0	0	1	0	1	0	0	0	0	1	1	1	0	0	0	0	0	0	0	0	1	0	0	0	0	0	0	0	1	0	0	1	0	1	0	1	0	0	0	0	0	0	0	0	0	0	0	1	1	1	0	1	1	0	1	1	0	1	0	1	0	0
seedMaxE-8	18	0	0	0	0	0	0	0	0	0	0	0	1	0	0	0	0	0	0	1	0	0	0	1	0	1	0	0	0	0	0	1	0	1	0	0	0	1	0	0	1	0	1	0	0	0	0	0	0	0	1	1	1	0	0	0	0	0	0	0	0	1	0	0	0	0	0	0	0	1	0	0	0	0	0	0	0	0	0	0	0	0	0	0	0	0	0	0	0	0	1	0	1	0	0	1	0	0	1	0	0	0	0
seedMaxE-8.seedMinPu0	18	0	0	0	0	0	0	0	0	0	0	0	1	0	0	0	0	0	0	1	0	0	0	1	0	1	0	0	0	0	0	1	0	1	0	0	0	1	0	0	1	0	1	0	0	0	0	0	0	0	1	1	1	0	0	0	0	0	0	0	0	1	0	0	0	0	0	0	0	1	0	0	0	0	0	0	0	0	0	0	0	0	0	0	0	0	0	0	0	0	1	0	1	0	0	1	0	0	1	0	0	0	0
seedMaxE-8.seedMinPu0.01	17	0	0	0	0	0	0	0	0	0	0	0	1	0	0	0	0	0	0	1	0	0	0	1	0	1	0	0	0	0	0	1	0	1	0	0	0	1	0	0	1	0	1	0	0	0	0	0	0	0	1	1	0	0	0	0	0	0	0	0	0	1	0	0	0	0	0	0	0	1	0	0	0	0	0	0	0	0	0	0	0	0	0	0	0	0	0	0	0	0	1	0	1	0	0	1	0	0	1	0	0	0	0
seedMaxE-8.seedMinPu0.0001	18	0	0	0	0	0	0	0	0	0	0	0	1	0	0	0	0	0	0	1	0	0	0	1	0	1	0	0	0	0	0	1	0	1	0	0	0	1	0	0	1	0	1	0	0	0	0	0	0	0	1	1	1	0	0	0	0	0	0	0	0	1	0	0	0	0	0	0	0	1	0	0	0	0	0	0	0	0	0	0	0	0	0	0	0	0	0	0	0	0	1	0	1	0	0	1	0	0	1	0	0	0	0
seedMaxE-8.seedMinPu0.001	18	0	0	0	0	0	0	0	0	0	0	0	1	0	0	0	0	0	0	1	0	0	0	1	0	1	0	0	0	0	0	1	0	1	0	0	0	1	0	0	1	0	1	0	0	0	0	0	0	0	1	1	1	0	0	0	0	0	0	0	0	1	0	0	0	0	0	0	0	1	0	0	0	0	0	0	0	0	0	0	0	0	0	0	0	0	0	0	0	0	1	0	1	0	0	1	0	0	1	0	0	0	0
seedMaxE-8.seedMinPu0.1	6	0	0	0	0	0	0	0	0	0	0	0	1	0	0	0	0	0	0	0	0	0	0	0	0	0	0	0	0	0	0	1	0	1	0	0	0	0	0	0	1	0	0	0	0	0	0	0	0	0	0	0	0	0	0	0	0	0	0	0	0	0	0	0	0	0	0	0	0	1	0	0	0	0	0	0	0	0	0	0	0	0	0	0	0	0	0	0	0	0	0	0	1	0	0	0	0	0	0	0	0	0	0
seedMaxE-8.seedMinPu0.1.seedNoGU	5	0	0	0	0	0	0	0	0	0	0	0	1	0	0	0	0	0	0	0	0	0	0	0	0	0	0	0	0	0	0	1	0	1	0	0	0	0	0	0	0	0	0	0	0	0	0	0	0	0	0	0	0	0	0	0	0	0	0	0	0	0	0	0	0	0	0	0	0	1	0	0	0	0	0	0	0	0	0	0	0	0	0	0	0	0	0	0	0	0	0	0	1	0	0	0	0	0	0	0	0	0	0
seedMaxE-8.seedMinPu0.001.seedNoGU	15	0	0	0	0	0	0	0	0	0	0	0	1	0	0	0	0	0	0	1	0	0	0	0	0	1	0	0	0	0	0	1	0	1	0	0	0	1	0	0	0	0	0	0	0	0	0	0	0	0	1	1	1	0	0	0	0	0	0	0	0	1	0	0	0	0	0	0	0	1	0	0	0	0	0	0	0	0	0	0	0	0	0	0	0	0	0	0	0	0	1	0	1	0	0	1	0	0	1	0	0	0	0
seedMaxE-8.seedMinPu0.0001.seedNoGU	15	0	0	0	0	0	0	0	0	0	0	0	1	0	0	0	0	0	0	1	0	0	0	0	0	1	0	0	0	0	0	1	0	1	0	0	0	1	0	0	0	0	0	0	0	0	0	0	0	0	1	1	1	0	0	0	0	0	0	0	0	1	0	0	0	0	0	0	0	1	0	0	0	0	0	0	0	0	0	0	0	0	0	0	0	0	0	0	0	0	1	0	1	0	0	1	0	0	1	0	0	0	0
seedMaxE-8.seedMinPu0.01.seedNoGU	14	0	0	0	0	0	0	0	0	0	0	0	1	0	0	0	0	0	0	1	0	0	0	0	0	1	0	0	0	0	0	1	0	1	0	0	0	1	0	0	0	0	0	0	0	0	0	0	0	0	1	1	0	0	0	0	0	0	0	0	0	1	0	0	0	0	0	0	0	1	0	0	0	0	0	0	0	0	0	0	0	0	0	0	0	0	0	0	0	0	1	0	1	0	0	1	0	0	1	0	0	0	0
seedMaxE-8.seedMinPu0.2	2	0	0	0	0	0	0	0	0	0	0	0	0	0	0	0	0	0	0	0	0	0	0	0	0	0	0	0	0	0	0	1	0	0	0	0	0	0	0	0	0	0	0	0	0	0	0	0	0	0	0	0	0	0	0	0	0	0	0	0	0	0	0	0	0	0	0	0	0	0	0	0	0	0	0	0	0	0	0	0	0	0	0	0	0	0	0	0	0	0	0	0	1	0	0	0	0	0	0	0	0	0	0
seedMaxE-8.seedMinPu0.2.seedNoGU	2	0	0	0	0	0	0	0	0	0	0	0	0	0	0	0	0	0	0	0	0	0	0	0	0	0	0	0	0	0	0	1	0	0	0	0	0	0	0	0	0	0	0	0	0	0	0	0	0	0	0	0	0	0	0	0	0	0	0	0	0	0	0	0	0	0	0	0	0	0	0	0	0	0	0	0	0	0	0	0	0	0	0	0	0	0	0	0	0	0	0	0	1	0	0	0	0	0	0	0	0	0	0
seedMaxE-8.seedMinPu0.5	0	0	0	0	0	0	0	0	0	0	0	0	0	0	0	0	0	0	0	0	0	0	0	0	0	0	0	0	0	0	0	0	0	0	0	0	0	0	0	0	0	0	0	0	0	0	0	0	0	0	0	0	0	0	0	0	0	0	0	0	0	0	0	0	0	0	0	0	0	0	0	0	0	0	0	0	0	0	0	0	0	0	0	0	0	0	0	0	0	0	0	0	0	0	0	0	0	0	0	0	0	0	0
seedMaxE-8.seedMinPu0.5.seedNoGU	0	0	0	0	0	0	0	0	0	0	0	0	0	0	0	0	0	0	0	0	0	0	0	0	0	0	0	0	0	0	0	0	0	0	0	0	0	0	0	0	0	0	0	0	0	0	0	0	0	0	0	0	0	0	0	0	0	0	0	0	0	0	0	0	0	0	0	0	0	0	0	0	0	0	0	0	0	0	0	0	0	0	0	0	0	0	0	0	0	0	0	0	0	0	0	0	0	0	0	0	0	0	0
seedMaxE-8.seedMinPu0.seedNoGU	15	0	0	0	0	0	0	0	0	0	0	0	1	0	0	0	0	0	0	1	0	0	0	0	0	1	0	0	0	0	0	1	0	1	0	0	0	1	0	0	0	0	0	0	0	0	0	0	0	0	1	1	1	0	0	0	0	0	0	0	0	1	0	0	0	0	0	0	0	1	0	0	0	0	0	0	0	0	0	0	0	0	0	0	0	0	0	0	0	0	1	0	1	0	0	1	0	0	1	0	0	0	0
seedMaxE-8.seedNoGU	15	0	0	0	0	0	0	0	0	0	0	0	1	0	0	0	0	0	0	1	0	0	0	0	0	1	0	0	0	0	0	1	0	1	0	0	0	1	0	0	0	0	0	0	0	0	0	0	0	0	1	1	1	0	0	0	0	0	0	0	0	1	0	0	0	0	0	0	0	1	0	0	0	0	0	0	0	0	0	0	0	0	0	0	0	0	0	0	0	0	1	0	1	0	0	1	0	0	1	0	0	0	0
seedMaxE-9	7	0	0	0	0	0	0	0	0	0	0	0	0	0	0	0	0	0	0	1	0	0	0	0	0	1	0	0	0	0	0	1	0	0	0	0	0	1	0	0	0	0	0	0	0	0	0	0	0	0	1	1	0	0	0	0	0	0	0	0	0	0	0	0	0	0	0	0	0	0	0	0	0	0	0	0	0	0	0	0	0	0	0	0	0	0	0	0	0	0	0	0	1	0	0	0	0	0	0	0	0	0	0
seedMaxE-9.seedMinPu0	7	0	0	0	0	0	0	0	0	0	0	0	0	0	0	0	0	0	0	1	0	0	0	0	0	1	0	0	0	0	0	1	0	0	0	0	0	1	0	0	0	0	0	0	0	0	0	0	0	0	1	1	0	0	0	0	0	0	0	0	0	0	0	0	0	0	0	0	0	0	0	0	0	0	0	0	0	0	0	0	0	0	0	0	0	0	0	0	0	0	0	0	1	0	0	0	0	0	0	0	0	0	0
seedMaxE-9.seedMinPu0.0001	7	0	0	0	0	0	0	0	0	0	0	0	0	0	0	0	0	0	0	1	0	0	0	0	0	1	0	0	0	0	0	1	0	0	0	0	0	1	0	0	0	0	0	0	0	0	0	0	0	0	1	1	0	0	0	0	0	0	0	0	0	0	0	0	0	0	0	0	0	0	0	0	0	0	0	0	0	0	0	0	0	0	0	0	0	0	0	0	0	0	0	0	1	0	0	0	0	0	0	0	0	0	0
seedMaxE-9.seedMinPu0.1	1	0	0	0	0	0	0	0	0	0	0	0	0	0	0	0	0	0	0	0	0	0	0	0	0	0	0	0	0	0	0	1	0	0	0	0	0	0	0	0	0	0	0	0	0	0	0	0	0	0	0	0	0	0	0	0	0	0	0	0	0	0	0	0	0	0	0	0	0	0	0	0	0	0	0	0	0	0	0	0	0	0	0	0	0	0	0	0	0	0	0	0	0	0	0	0	0	0	0	0	0	0	0
seedMaxE-9.seedMinPu0.01	7	0	0	0	0	0	0	0	0	0	0	0	0	0	0	0	0	0	0	1	0	0	0	0	0	1	0	0	0	0	0	1	0	0	0	0	0	1	0	0	0	0	0	0	0	0	0	0	0	0	1	1	0	0	0	0	0	0	0	0	0	0	0	0	0	0	0	0	0	0	0	0	0	0	0	0	0	0	0	0	0	0	0	0	0	0	0	0	0	0	0	0	1	0	0	0	0	0	0	0	0	0	0
seedMaxE-9.seedMinPu0.001	7	0	0	0	0	0	0	0	0	0	0	0	0	0	0	0	0	0	0	1	0	0	0	0	0	1	0	0	0	0	0	1	0	0	0	0	0	1	0	0	0	0	0	0	0	0	0	0	0	0	1	1	0	0	0	0	0	0	0	0	0	0	0	0	0	0	0	0	0	0	0	0	0	0	0	0	0	0	0	0	0	0	0	0	0	0	0	0	0	0	0	0	1	0	0	0	0	0	0	0	0	0	0
seedMaxE-9.seedMinPu0.001.seedNoGU	6	0	0	0	0	0	0	0	0	0	0	0	0	0	0	0	0	0	0	1	0	0	0	0	0	1	0	0	0	0	0	1	0	0	0	0	0	1	0	0	0	0	0	0	0	0	0	0	0	0	1	1	0	0	0	0	0	0	0	0	0	0	0	0	0	0	0	0	0	0	0	0	0	0	0	0	0	0	0	0	0	0	0	0	0	0	0	0	0	0	0	0	0	0	0	0	0	0	0	0	0	0	0
seedMaxE-9.seedMinPu0.1.seedNoGU	1	0	0	0	0	0	0	0	0	0	0	0	0	0	0	0	0	0	0	0	0	0	0	0	0	0	0	0	0	0	0	1	0	0	0	0	0	0	0	0	0	0	0	0	0	0	0	0	0	0	0	0	0	0	0	0	0	0	0	0	0	0	0	0	0	0	0	0	0	0	0	0	0	0	0	0	0	0	0	0	0	0	0	0	0	0	0	0	0	0	0	0	0	0	0	0	0	0	0	0	0	0	0
seedMaxE-9.seedMinPu0.01.seedNoGU	6	0	0	0	0	0	0	0	0	0	0	0	0	0	0	0	0	0	0	1	0	0	0	0	0	1	0	0	0	0	0	1	0	0	0	0	0	1	0	0	0	0	0	0	0	0	0	0	0	0	1	1	0	0	0	0	0	0	0	0	0	0	0	0	0	0	0	0	0	0	0	0	0	0	0	0	0	0	0	0	0	0	0	0	0	0	0	0	0	0	0	0	0	0	0	0	0	0	0	0	0	0	0
seedMaxE-9.seedMinPu0.0001.seedNoGU	6	0	0	0	0	0	0	0	0	0	0	0	0	0	0	0	0	0	0	1	0	0	0	0	0	1	0	0	0	0	0	1	0	0	0	0	0	1	0	0	0	0	0	0	0	0	0	0	0	0	1	1	0	0	0	0	0	0	0	0	0	0	0	0	0	0	0	0	0	0	0	0	0	0	0	0	0	0	0	0	0	0	0	0	0	0	0	0	0	0	0	0	0	0	0	0	0	0	0	0	0	0	0
seedMaxE-9.seedMinPu0.2	1	0	0	0	0	0	0	0	0	0	0	0	0	0	0	0	0	0	0	0	0	0	0	0	0	0	0	0	0	0	0	1	0	0	0	0	0	0	0	0	0	0	0	0	0	0	0	0	0	0	0	0	0	0	0	0	0	0	0	0	0	0	0	0	0	0	0	0	0	0	0	0	0	0	0	0	0	0	0	0	0	0	0	0	0	0	0	0	0	0	0	0	0	0	0	0	0	0	0	0	0	0	0
seedMaxE-9.seedMinPu0.2.seedNoGU	1	0	0	0	0	0	0	0	0	0	0	0	0	0	0	0	0	0	0	0	0	0	0	0	0	0	0	0	0	0	0	1	0	0	0	0	0	0	0	0	0	0	0	0	0	0	0	0	0	0	0	0	0	0	0	0	0	0	0	0	0	0	0	0	0	0	0	0	0	0	0	0	0	0	0	0	0	0	0	0	0	0	0	0	0	0	0	0	0	0	0	0	0	0	0	0	0	0	0	0	0	0	0
seedMaxE-9.seedMinPu0.5	0	0	0	0	0	0	0	0	0	0	0	0	0	0	0	0	0	0	0	0	0	0	0	0	0	0	0	0	0	0	0	0	0	0	0	0	0	0	0	0	0	0	0	0	0	0	0	0	0	0	0	0	0	0	0	0	0	0	0	0	0	0	0	0	0	0	0	0	0	0	0	0	0	0	0	0	0	0	0	0	0	0	0	0	0	0	0	0	0	0	0	0	0	0	0	0	0	0	0	0	0	0	0
seedMaxE-9.seedMinPu0.5.seedNoGU	0	0	0	0	0	0	0	0	0	0	0	0	0	0	0	0	0	0	0	0	0	0	0	0	0	0	0	0	0	0	0	0	0	0	0	0	0	0	0	0	0	0	0	0	0	0	0	0	0	0	0	0	0	0	0	0	0	0	0	0	0	0	0	0	0	0	0	0	0	0	0	0	0	0	0	0	0	0	0	0	0	0	0	0	0	0	0	0	0	0	0	0	0	0	0	0	0	0	0	0	0	0	0
seedMaxE-9.seedMinPu0.seedNoGU	6	0	0	0	0	0	0	0	0	0	0	0	0	0	0	0	0	0	0	1	0	0	0	0	0	1	0	0	0	0	0	1	0	0	0	0	0	1	0	0	0	0	0	0	0	0	0	0	0	0	1	1	0	0	0	0	0	0	0	0	0	0	0	0	0	0	0	0	0	0	0	0	0	0	0	0	0	0	0	0	0	0	0	0	0	0	0	0	0	0	0	0	0	0	0	0	0	0	0	0	0	0	0
seedMaxE-9.seedNoGU	6	0	0	0	0	0	0	0	0	0	0	0	0	0	0	0	0	0	0	1	0	0	0	0	0	1	0	0	0	0	0	1	0	0	0	0	0	1	0	0	0	0	0	0	0	0	0	0	0	0	1	1	0	0	0	0	0	0	0	0	0	0	0	0	0	0	0	0	0	0	0	0	0	0	0	0	0	0	0	0	0	0	0	0	0	0	0	0	0	0	0	0	0	0	0	0	0	0	0	0	0	0	0
seedMaxE-10	3	0	0	0	0	0	0	0	0	0	0	0	0	0	0	0	0	0	0	0	0	0	0	0	0	0	0	0	0	0	0	1	0	0	0	0	0	0	0	0	0	0	0	0	0	0	0	0	0	0	1	1	0	0	0	0	0	0	0	0	0	0	0	0	0	0	0	0	0	0	0	0	0	0	0	0	0	0	0	0	0	0	0	0	0	0	0	0	0	0	0	0	0	0	0	0	0	0	0	0	0	0	0
seedMaxE-10.seedMinPu0	3	0	0	0	0	0	0	0	0	0	0	0	0	0	0	0	0	0	0	0	0	0	0	0	0	0	0	0	0	0	0	1	0	0	0	0	0	0	0	0	0	0	0	0	0	0	0	0	0	0	1	1	0	0	0	0	0	0	0	0	0	0	0	0	0	0	0	0	0	0	0	0	0	0	0	0	0	0	0	0	0	0	0	0	0	0	0	0	0	0	0	0	0	0	0	0	0	0	0	0	0	0	0
seedMaxE-10.seedMinPu0.1	1	0	0	0	0	0	0	0	0	0	0	0	0	0	0	0	0	0	0	0	0	0	0	0	0	0	0	0	0	0	0	1	0	0	0	0	0	0	0	0	0	0	0	0	0	0	0	0	0	0	0	0	0	0	0	0	0	0	0	0	0	0	0	0	0	0	0	0	0	0	0	0	0	0	0	0	0	0	0	0	0	0	0	0	0	0	0	0	0	0	0	0	0	0	0	0	0	0	0	0	0	0	0
seedMaxE-10.seedMinPu0.0001	3	0	0	0	0	0	0	0	0	0	0	0	0	0	0	0	0	0	0	0	0	0	0	0	0	0	0	0	0	0	0	1	0	0	0	0	0	0	0	0	0	0	0	0	0	0	0	0	0	0	1	1	0	0	0	0	0	0	0	0	0	0	0	0	0	0	0	0	0	0	0	0	0	0	0	0	0	0	0	0	0	0	0	0	0	0	0	0	0	0	0	0	0	0	0	0	0	0	0	0	0	0	0
seedMaxE-10.seedMinPu0.001	3	0	0	0	0	0	0	0	0	0	0	0	0	0	0	0	0	0	0	0	0	0	0	0	0	0	0	0	0	0	0	1	0	0	0	0	0	0	0	0	0	0	0	0	0	0	0	0	0	0	1	1	0	0	0	0	0	0	0	0	0	0	0	0	0	0	0	0	0	0	0	0	0	0	0	0	0	0	0	0	0	0	0	0	0	0	0	0	0	0	0	0	0	0	0	0	0	0	0	0	0	0	0
seedMaxE-10.seedMinPu0.01	3	0	0	0	0	0	0	0	0	0	0	0	0	0	0	0	0	0	0	0	0	0	0	0	0	0	0	0	0	0	0	1	0	0	0	0	0	0	0	0	0	0	0	0	0	0	0	0	0	0	1	1	0	0	0	0	0	0	0	0	0	0	0	0	0	0	0	0	0	0	0	0	0	0	0	0	0	0	0	0	0	0	0	0	0	0	0	0	0	0	0	0	0	0	0	0	0	0	0	0	0	0	0
seedMaxE-10.seedMinPu0.01.seedNoGU	3	0	0	0	0	0	0	0	0	0	0	0	0	0	0	0	0	0	0	0	0	0	0	0	0	0	0	0	0	0	0	1	0	0	0	0	0	0	0	0	0	0	0	0	0	0	0	0	0	0	1	1	0	0	0	0	0	0	0	0	0	0	0	0	0	0	0	0	0	0	0	0	0	0	0	0	0	0	0	0	0	0	0	0	0	0	0	0	0	0	0	0	0	0	0	0	0	0	0	0	0	0	0

Echericha coli NC_000913	sum	ArcZ-b1892	ArcZ-b2741	ArcZ-b3546	ChiX-b0619	ChiX-b0681	ChiX-b1737	CyaR-b0723	CyaR-b0814	CyaR-b1740	CyaR-b1824	CyaR-b2416	CyaR-b2666	CyaR-b2687	DsrA-b1237	DsrA-b2741	DsrA-b3251	FnrS-b0723	FnrS-b0755	FnrS-b0887	FnrS-b1107	FnrS-b1479	FnrS-b1531	FnrS-b1656	FnrS-b1841	FnrS-b2153	FnrS-b2303	FnrS-b2531	FnrS-b3829	FnrS-b3908	GcvB-b1040	GcvB-b1130	GcvB-b3089	GcvB-b4208	MicA-b0411	MicA-b0814	MicA-b0957	MicA-b1130	MicC-b2215	MicF-b0241	MicF-b0889	MicF-b0929	MicF-b3912	OxyS-b1892	OxyS-b2731	RprA-b1040	RprA-b1341	RprA-b2741	RybB-b0081	RybB-b0721	RybB-b0805	RybB-b1256	RybB-b2215	RybB-b2594	RyhB-b0118	RyhB-b0156	RyhB-b0288	RyhB-b0592	RyhB-b0683	RyhB-b0721	RyhB-b0723	RyhB-b0894	RyhB-b1107	RyhB-b1200	RyhB-b1452	RyhB-b1531	RyhB-b1588	RyhB-b1612	RyhB-b1656	RyhB-b1778	RyhB-b1981	RyhB-b2069	RyhB-b2155	RyhB-b2206	RyhB-b2530	RyhB-b3365	RyhB-b3607	RyhB-b3942	RyhB-b4070	RyhB-b4122	SgrS-b1101	SgrS-b1817	SgrS-b2416	Spot42-b0039	Spot42-b0720	Spot42-b0721	Spot42-b0728	Spot42-b0757	Spot42-b1136	Spot42-b1302	Spot42-b1398	Spot42-b1761	Spot42-b1901	Spot42-b2221	Spot42-b2702	Spot42-b2715	Spot42-b2801	Spot42-b2802	Spot42-b3224	Spot42-b3566	Spot42-b3927	Spot42-b3962	Spot42-b4311
Target found among top-100 predictions for the sRNA for any tested parameterization	86	0	1	0	1	1	1	1	1	0	1	1	1	1	1	1	0	1	0	1	1	1	1	1	1	1	1	1	1	0	1	1	1	1	1	1	1	1	1	1	1	1	1	1	0	1	1	1	1	1	1	1	1	1	0	1	1	1	0	1	0	1	1	1	0	0	1	1	1	1	1	0	1	1	1	1	1	0	1	1	1	1	1	0	1	1	1	1	1	1	1	1	1	1	1	1	1	1	0	1	1	1	1	1
seedMaxE-10.seedMinPu0.0001.seedNoGU	3	0	0	0	0	0	0	0	0	0	0	0	0	0	0	0	0	0	0	0	0	0	0	0	0	0	0	0	0	0	0	1	0	0	0	0	0	0	0	0	0	0	0	0	0	0	0	0	0	0	1	1	0	0	0	0	0	0	0	0	0	0	0	0	0	0	0	0	0	0	0	0	0	0	0	0	0	0	0	0	0	0	0	0	0	0	0	0	0	0	0	0	0	0	0	0	0	0	0	0	0	0	0
seedMaxE-10.seedMinPu0.1.seedNoGU	1	0	0	0	0	0	0	0	0	0	0	0	0	0	0	0	0	0	0	0	0	0	0	0	0	0	0	0	0	0	0	1	0	0	0	0	0	0	0	0	0	0	0	0	0	0	0	0	0	0	0	0	0	0	0	0	0	0	0	0	0	0	0	0	0	0	0	0	0	0	0	0	0	0	0	0	0	0	0	0	0	0	0	0	0	0	0	0	0	0	0	0	0	0	0	0	0	0	0	0	0	0	0
seedMaxE-10.seedMinPu0.001.seedNoGU	3	0	0	0	0	0	0	0	0	0	0	0	0	0	0	0	0	0	0	0	0	0	0	0	0	0	0	0	0	0	0	1	0	0	0	0	0	0	0	0	0	0	0	0	0	0	0	0	0	0	1	1	0	0	0	0	0	0	0	0	0	0	0	0	0	0	0	0	0	0	0	0	0	0	0	0	0	0	0	0	0	0	0	0	0	0	0	0	0	0	0	0	0	0	0	0	0	0	0	0	0	0	0
seedMaxE-10.seedMinPu0.2	1	0	0	0	0	0	0	0	0	0	0	0	0	0	0	0	0	0	0	0	0	0	0	0	0	0	0	0	0	0	0	1	0	0	0	0	0	0	0	0	0	0	0	0	0	0	0	0	0	0	0	0	0	0	0	0	0	0	0	0	0	0	0	0	0	0	0	0	0	0	0	0	0	0	0	0	0	0	0	0	0	0	0	0	0	0	0	0	0	0	0	0	0	0	0	0	0	0	0	0	0	0	0
seedMaxE-10.seedMinPu0.2.seedNoGU	1	0	0	0	0	0	0	0	0	0	0	0	0	0	0	0	0	0	0	0	0	0	0	0	0	0	0	0	0	0	0	1	0	0	0	0	0	0	0	0	0	0	0	0	0	0	0	0	0	0	0	0	0	0	0	0	0	0	0	0	0	0	0	0	0	0	0	0	0	0	0	0	0	0	0	0	0	0	0	0	0	0	0	0	0	0	0	0	0	0	0	0	0	0	0	0	0	0	0	0	0	0	0
seedMaxE-10.seedMinPu0.5	0	0	0	0	0	0	0	0	0	0	0	0	0	0	0	0	0	0	0	0	0	0	0	0	0	0	0	0	0	0	0	0	0	0	0	0	0	0	0	0	0	0	0	0	0	0	0	0	0	0	0	0	0	0	0	0	0	0	0	0	0	0	0	0	0	0	0	0	0	0	0	0	0	0	0	0	0	0	0	0	0	0	0	0	0	0	0	0	0	0	0	0	0	0	0	0	0	0	0	0	0	0	0
seedMaxE-10.seedMinPu0.5.seedNoGU	0	0	0	0	0	0	0	0	0	0	0	0	0	0	0	0	0	0	0	0	0	0	0	0	0	0	0	0	0	0	0	0	0	0	0	0	0	0	0	0	0	0	0	0	0	0	0	0	0	0	0	0	0	0	0	0	0	0	0	0	0	0	0	0	0	0	0	0	0	0	0	0	0	0	0	0	0	0	0	0	0	0	0	0	0	0	0	0	0	0	0	0	0	0	0	0	0	0	0	0	0	0	0
seedMaxE-10.seedMinPu0.seedNoGU	3	0	0	0	0	0	0	0	0	0	0	0	0	0	0	0	0	0	0	0	0	0	0	0	0	0	0	0	0	0	0	1	0	0	0	0	0	0	0	0	0	0	0	0	0	0	0	0	0	0	1	1	0	0	0	0	0	0	0	0	0	0	0	0	0	0	0	0	0	0	0	0	0	0	0	0	0	0	0	0	0	0	0	0	0	0	0	0	0	0	0	0	0	0	0	0	0	0	0	0	0	0	0
seedMaxE-10.seedNoGU	3	0	0	0	0	0	0	0	0	0	0	0	0	0	0	0	0	0	0	0	0	0	0	0	0	0	0	0	0	0	0	1	0	0	0	0	0	0	0	0	0	0	0	0	0	0	0	0	0	0	1	1	0	0	0	0	0	0	0	0	0	0	0	0	0	0	0	0	0	0	0	0	0	0	0	0	0	0	0	0	0	0	0	0	0	0	0	0	0	0	0	0	0	0	0	0	0	0	0	0	0	0	0
seedMaxEhybrid0	36	0	0	0	1	1	1	0	1	0	1	1	0	0	1	1	0	1	0	0	0	0	1	0	1	0	0	1	1	0	0	0	0	0	0	0	1	1	1	0	1	1	0	0	0	1	0	1	1	0	0	0	0	0	0	0	0	0	0	0	0	1	1	0	0	0	0	0	0	0	0	0	0	0	0	1	1	0	0	0	1	0	0	0	0	1	0	1	1	1	1	1	0	0	0	0	1	0	1	0	1	0	1
seedMaxEhybrid0.seedMinPu0	36	0	0	0	1	1	1	0	1	0	1	1	0	0	1	1	0	1	0	0	0	0	1	0	1	0	0	1	1	0	0	0	0	0	0	0	1	1	1	0	1	1	0	0	0	1	0	1	1	0	0	0	0	0	0	0	0	0	0	0	0	1	1	0	0	0	0	0	0	0	0	0	0	0	0	1	1	0	0	0	1	0	0	0	0	1	0	1	1	1	1	1	0	0	0	0	1	0	1	0	1	0	1
seedMaxEhybrid0.seedMinPu0.01	40	0	0	0	1	1	1	0	1	0	1	1	0	0	1	1	0	1	0	0	0	0	1	1	1	0	0	1	1	0	0	0	0	0	0	0	1	1	1	0	1	1	0	0	0	1	0	1	1	0	0	0	0	0	0	1	0	0	0	0	0	1	1	0	0	0	0	0	0	0	0	0	1	0	0	1	1	0	0	0	1	0	1	0	0	0	0	0	1	1	1	1	1	1	0	0	1	0	1	0	1	0	1
seedMaxEhybrid0.seedMinPu0.1	25	0	1	0	1	1	1	1	0	0	0	0	0	0	1	1	0	0	0	0	0	0	1	0	1	0	0	1	0	0	0	0	0	1	0	1	0	0	1	1	1	1	0	0	0	0	0	0	0	0	0	0	0	0	0	0	0	0	0	0	0	0	1	0	0	0	0	0	0	1	0	0	0	0	0	0	0	0	1	0	0	0	0	0	0	0	0	1	1	0	0	0	1	0	0	0	1	0	0	0	1	1	0
seedMaxEhybrid0.seedMinPu0.0001	37	0	0	0	1	1	1	0	1	0	1	1	0	0	1	1	0	1	0	0	0	0	1	1	1	0	0	1	1	0	0	0	0	0	0	0	1	1	1	0	1	1	0	0	0	1	0	1	1	0	0	0	0	0	0	0	0	0	0	0	0	1	1	0	0	0	0	0	0	0	0	0	0	0	0	1	1	0	0	0	1	0	0	0	0	1	0	1	1	1	1	1	0	0	0	0	1	0	1	0	1	0	1
seedMaxEhybrid0.seedMinPu0.001	37	0	0	0	1	1	1	0	1	0	1	1	0	0	1	1	0	1	0	0	0	0	1	1	1	0	0	1	1	0	0	0	0	0	0	0	1	1	1	0	1	1	0	0	0	1	0	1	1	0	0	0	0	0	0	0	0	0	0	0	0	1	1	0	0	0	0	0	0	0	0	0	0	0	0	1	1	0	0	0	1	0	1	0	0	1	0	0	1	1	1	1	0	0	0	0	1	0	1	0	1	0	1
seedMaxEhybrid0.seedMinPu0.1.seedNoGU	25	0	1	0	1	1	1	1	0	0	0	0	1	0	1	1	0	0	0	0	0	0	1	0	1	0	1	1	0	0	0	0	1	1	0	0	0	0	1	1	0	1	0	0	0	0	0	0	0	0	0	0	0	0	0	0	0	0	0	0	0	0	0	0	0	0	0	1	0	1	0	0	0	0	0	0	0	0	1	0	0	0	0	0	0	0	0	0	1	0	0	0	1	0	0	0	1	0	0	0	1	1	0
seedMaxEhybrid0.seedMinPu0.01.seedNoGU	47	0	1	0	1	1	1	0	1	0	1	1	0	0	1	1	0	0	0	0	0	0	1	0	1	0	0	1	1	0	0	0	0	1	0	1	1	1	1	1	0	1	0	0	0	1	0	1	0	0	1	1	0	0	0	0	0	1	0	0	0	1	0	0	0	0	1	0	0	1	0	0	1	1	1	1	1	0	0	0	1	0	1	0	0	0	1	1	1	1	1	1	1	1	0	1	1	0	1	0	1	0	0
seedMaxEhybrid0.seedMinPu0.001.seedNoGU	49	0	0	0	1	1	1	0	1	0	1	1	0	0	1	1	0	0	0	0	0	0	1	0	1	0	0	1	1	0	0	0	0	1	0	1	1	1	1	1	0	1	0	0	0	1	0	1	0	0	1	0	1	0	0	1	0	1	0	1	0	1	0	0	0	0	1	0	0	0	0	0	1	1	1	1	1	0	0	1	1	0	1	0	0	0	1	1	1	1	1	1	1	1	0	1	1	0	1	1	1	0	0
seedMaxEhybrid0.seedMinPu0.0001.seedNoGU	47	0	0	0	1	1	1	0	1	0	1	1	0	0	1	1	0	0	0	0	0	0	1	0	1	0	0	1	1	0	0	0	0	1	0	1	1	1	1	1	0	1	0	0	0	1	0	1	0	0	1	0	1	0	0	1	0	1	0	1	0	1	0	0	0	0	1	0	0	0	0	0	1	1	0	1	1	0	0	0	1	0	1	0	0	1	1	1	1	1	1	1	0	1	0	1	1	0	1	1	1	0	0
seedMaxEhybrid0.seedMinPu0.2	17	0	0	0	1	1	1	0	0	0	0	0	0	0	0	1	0	0	0	0	0	0	1	0	1	0	1	1	0	0	0	0	1	0	0	0	0	0	1	1	1	1	0	0	0	0	1	0	0	0	0	0	0	0	0	0	0	0	0	0	0	0	0	0	0	0	0	0	0	0	0	0	0	0	0	0	0	0	0	0	0	0	0	0	0	0	0	0	0	0	0	0	1	0	0	0	1	0	0	0	1	0	0
seedMaxEhybrid0.seedMinPu0.2.seedNoGU	16	0	0	0	1	1	1	0	0	0	0	0	0	0	0	0	0	0	0	0	0	0	1	0	1	0	1	1	0	0	0	1	1	0	0	0	0	0	1	1	0	1	0	0	0	0	0	0	0	0	0	0	0	0	0	0	0	0	0	0	0	0	0	0	0	0	0	0	0	0	0	0	0	0	0	0	0	0	0	0	0	0	0	0	1	0	0	0	0	0	0	0	1	0	0	0	1	0	0	0	1	0	0
seedMaxEhybrid0.seedMinPu0.5	8	0	1	0	0	0	0	0	0	0	0	0	0	0	0	0	0	0	0	0	0	1	1	0	1	0	0	0	0	0	0	0	0	0	0	0	0	0	1	0	0	1	0	0	0	0	0	0	0	0	0	0	0	0	0	0	0	0	0	0	0	0	0	0	0	0	0	0	0	0	0	0	0	0	0	1	0	0	0	0	0	0	0	0	1	0	0	0	0	0	0	0	0	0	0	0	0	0	0	0	0	0	0
seedMaxEhybrid0.seedMinPu0.5.seedNoGU	7	0	1	0	0	0	0	0	0	0	0	0	0	0	0	0	0	0	0	0	0	0	1	0	1	0	0	0	0	0	0	0	0	0	0	0	0	0	1	0	0	1	0	0	0	0	0	0	0	0	0	0	0	0	0	0	0	0	0	0	0	0	0	0	0	0	0	0	0	0	0	0	0	0	0	0	0	0	0	0	0	0	0	0	1	0	0	1	0	0	0	0	0	0	0	0	0	0	0	0	0	0	0
seedMaxEhybrid0.seedMinPu0.seedNoGU	47	0	0	0	1	1	1	0	1	0	1	1	0	0	1	1	0	0	0	0	0	0	1	0	1	0	0	1	1	0	0	0	0	1	0	1	1	1	1	1	0	1	0	0	0	1	0	1	0	0	1	0	1	0	0	1	0	1	0	1	0	1	0	0	0	0	1	0	0	0	0	0	1	1	0	1	1	0	0	0	1	0	1	0	0	1	1	1	1	1	1	1	0	1	0	1	1	0	1	1	1	0	0
seedMaxEhybrid0.seedNoGU	47	0	0	0	1	1	1	0	1	0	1	1	0	0	1	1	0	0	0	0	0	0	1	0	1	0	0	1	1	0	0	0	0	1	0	1	1	1	1	1	0	1	0	0	0	1	0	1	0	0	1	0	1	0	0	1	0	1	0	1	0	1	0	0	0	0	1	0	0	0	0	0	1	1	0	1	1	0	0	0	1	0	1	0	0	1	1	1	1	1	1	1	0	1	0	1	1	0	1	1	1	0	0
seedMaxEhybrid-1	36	0	0	0	1	1	1	0	1	0	1	1	0	0	1	1	0	1	0	0	0	0	1	0	1	0	0	1	1	0	0	0	0	0	0	0	1	1	1	0	1	1	0	0	0	1	0	1	1	0	0	0	0	0	0	0	0	0	0	0	0	1	1	0	0	0	0	0	0	0	0	0	0	0	0	1	1	0	0	0	1	0	0	0	0	1	0	1	1	1	1	1	0	0	0	0	1	0	1	0	1	0	1
seedMaxEhybrid-1.seedMinPu0	36	0	0	0	1	1	1	0	1	0	1	1	0	0	1	1	0	1	0	0	0	0	1	0	1	0	0	1	1	0	0	0	0	0	0	0	1	1	1	0	1	1	0	0	0	1	0	1	1	0	0	0	0	0	0	0	0	0	0	0	0	1	1	0	0	0	0	0	0	0	0	0	0	0	0	1	1	0	0	0	1	0	0	0	0	1	0	1	1	1	1	1	0	0	0	0	1	0	1	0	1	0	1
seedMaxEhybrid-1.seedMinPu0.0001	37	0	0	0	1	1	1	0	1	0	1	1	0	0	1	1	0	1	0	0	0	0	1	1	1	0	0	1	1	0	0	0	0	0	0	0	1	1	1	0	1	1	0	0	0	1	0	1	1	0	0	0	0	0	0	0	0	0	0	0	0	1	1	0	0	0	0	0	0	0	0	0	0	0	0	1	1	0	0	0	1	0	0	0	0	1	0	1	1	1	1	1	0	0	0	0	1	0	1	0	1	0	1
seedMaxEhybrid-1.seedMinPu0.01	40	0	0	0	1	1	1	0	1	0	1	1	0	0	1	1	0	1	0	0	0	0	1	1	1	0	0	1	1	0	0	0	0	0	0	0	1	1	1	0	1	1	0	0	0	1	0	1	1	0	0	0	0	0	0	1	0	0	0	0	0	1	1	0	0	0	0	0	0	0	0	0	1	0	0	1	1	0	0	0	1	0	1	0	0	0	0	0	1	1	1	1	1	1	0	0	1	0	1	0	1	0	1
seedMaxEhybrid-1.seedMinPu0.1	25	0	1	0	1	1	1	1	0	0	0	0	0	0	1	1	0	0	0	0	0	0	1	0	1	0	0	1	0	0	0	0	0	1	0	1	0	0	1	1	1	1	0	0	0	0	0	0	0	0	0	0	0	0	0	0	0	0	0	0	0	0	1	0	0	0	0	0	0	1	0	0	0	0	0	0	0	0	1	0	0	0	0	0	0	0	0	1	1	0	0	0	1	0	0	0	1	0	0	0	1	1	0
seedMaxEhybrid-1.seedMinPu0.001	37	0	0	0	1	1	1	0	1	0	1	1	0	0	1	1	0	1	0	0	0	0	1	1	1	0	0	1	1	0	0	0	0	0	0	0	1	1	1	0	1	1	0	0	0	1	0	1	1	0	0	0	0	0	0	0	0	0	0	0	0	1	1	0	0	0	0	0	0	0	0	0	0	0	0	1	1	0	0	0	1	0	1	0	0	1	0	0	1	1	1	1	0	0	0	0	1	0	1	0	1	0	1
seedMaxEhybrid-1.seedMinPu0.001.seedNoGU	49	0	0	0	1	1	1	0	1	0	1	1	0	0	1	1	0	0	0	0	0	0	1	0	1	0	0	1	1	0	0	0	0	1	0	1	1	1	1	1	0	1	0	0	0	1	0	1	0	0	1	0	1	0	0	1	0	1	0	1	0	1	0	0	0	0	1	0	0	0	0	0	1	1	1	1	1	0	0	1	1	0	1	0	0	0	1	1	1	1	1	1	1	1	0	1	1	0	1	1	1	0	0
seedMaxEhybrid-1.seedMinPu0.1.seedNoGU	25	0	1	0	1	1	1	1	0	0	0	0	1	0	1	1	0	0	0	0	0	0	1	0	1	0	1	1	0	0	0	0	1	1	0	0	0	0	1	1	0	1	0	0	0	0	0	0	0	0	0	0	0	0	0	0	0	0	0	0	0	0	0	0	0	0	0	1	0	1	0	0	0	0	0	0	0	0	1	0	0	0	0	0	0	0	0	0	1	0	0	0	1	0	0	0	1	0	0	0	1	1	0
seedMaxEhybrid-1.seedMinPu0.01.seedNoGU	47	0	1	0	1	1	1	0	1	0	1	1	0	0	1	1	0	0	0	0	0	0	1	0	1	0	0	1	1	0	0	0	0	1	0	1	1	1	1	1	0	1	0	0	0	1	0	1	0	0	1	1	0	0	0	0	0	1	0	0	0	1	0	0	0	0	1	0	0	1	0	0	1	1	1	1	1	0	0	0	1	0	1	0	0	0	1	1	1	1	1	1	1	1	0	1	1	0	1	0	1	0	0
seedMaxEhybrid-1.seedMinPu0.0001.seedNoGU	47	0	0	0	1	1	1	0	1	0	1	1	0	0	1	1	0	0	0	0	0	0	1	0	1	0	0	1	1	0	0	0	0	1	0	1	1	1	1	1	0	1	0	0	0	1	0	1	0	0	1	0	1	0	0	1	0	1	0	1	0	1	0	0	0	0	1	0	0	0	0	0	1	1	0	1	1	0	0	0	1	0	1	0	0	1	1	1	1	1	1	1	0	1	0	1	1	0	1	1	1	0	0
seedMaxEhybrid-1.seedMinPu0.2	17	0	0	0	1	1	1	0	0	0	0	0	0	0	0	1	0	0	0	0	0	0	1	0	1	0	1	1	0	0	0	0	1	0	0	0	0	0	1	1	1	1	0	0	0	0	1	0	0	0	0	0	0	0	0	0	0	0	0	0	0	0	0	0	0	0	0	0	0	0	0	0	0	0	0	0	0	0	0	0	0	0	0	0	0	0	0	0	0	0	0	0	1	0	0	0	1	0	0	0	1	0	0
seedMaxEhybrid-1.seedMinPu0.2.seedNoGU	16	0	0	0	1	1	1	0	0	0	0	0	0	0	0	0	0	0	0	0	0	0	1	0	1	0	1	1	0	0	0	1	1	0	0	0	0	0	1	1	0	1	0	0	0	0	0	0	0	0	0	0	0	0	0	0	0	0	0	0	0	0	0	0	0	0	0	0	0	0	0	0	0	0	0	0	0	0	0	0	0	0	0	0	1	0	0	0	0	0	0	0	1	0	0	0	1	0	0	0	1	0	0
seedMaxEhybrid-1.seedMinPu0.5	8	0	1	0	0	0	0	0	0	0	0	0	0	0	0	0	0	0	0	0	0	1	1	0	1	0	0	0	0	0	0	0	0	0	0	0	0	0	1	0	0	1	0	0	0	0	0	0	0	0	0	0	0	0	0	0	0	0	0	0	0	0	0	0	0	0	0	0	0	0	0	0	0	0	0	1	0	0	0	0	0	0	0	0	1	0	0	0	0	0	0	0	0	0	0	0	0	0	0	0	0	0	0
seedMaxEhybrid-1.seedMinPu0.5.seedNoGU	7	0	1	0	0	0	0	0	0	0	0	0	0	0	0	0	0	0	0	0	0	0	1	0	1	0	0	0	0	0	0	0	0	0	0	0	0	0	1	0	0	1	0	0	0	0	0	0	0	0	0	0	0	0	0	0	0	0	0	0	0	0	0	0	0	0	0	0	0	0	0	0	0	0	0	0	0	0	0	0	0	0	0	0	1	0	0	1	0	0	0	0	0	0	0	0	0	0	0	0	0	0	0
seedMaxEhybrid-1.seedMinPu0.seedNoGU	47	0	0	0	1	1	1	0	1	0	1	1	0	0	1	1	0	0	0	0	0	0	1	0	1	0	0	1	1	0	0	0	0	1	0	1	1	1	1	1	0	1	0	0	0	1	0	1	0	0	1	0	1	0	0	1	0	1	0	1	0	1	0	0	0	0	1	0	0	0	0	0	1	1	0	1	1	0	0	0	1	0	1	0	0	1	1	1	1	1	1	1	0	1	0	1	1	0	1	1	1	0	0
seedMaxEhybrid-1.seedNoGU	47	0	0	0	1	1	1	0	1	0	1	1	0	0	1	1	0	0	0	0	0	0	1	0	1	0	0	1	1	0	0	0	0	1	0	1	1	1	1	1	0	1	0	0	0	1	0	1	0	0	1	0	1	0	0	1	0	1	0	1	0	1	0	0	0	0	1	0	0	0	0	0	1	1	0	1	1	0	0	0	1	0	1	0	0	1	1	1	1	1	1	1	0	1	0	1	1	0	1	1	1	0	0
seedMaxEhybrid-2	36	0	0	0	1	1	1	0	1	0	1	1	0	0	1	1	0	1	0	0	0	0	1	0	1	0	0	1	1	0	0	0	0	0	0	0	1	1	1	0	1	1	0	0	0	1	0	1	1	0	0	0	0	0	0	0	0	0	0	0	0	1	1	0	0	0	0	0	0	0	0	0	0	0	0	1	1	0	0	0	1	0	0	0	0	1	0	1	1	1	1	1	0	0	0	0	1	0	1	0	1	0	1
seedMaxEhybrid-2.seedMinPu0	36	0	0	0	1	1	1	0	1	0	1	1	0	0	1	1	0	1	0	0	0	0	1	0	1	0	0	1	1	0	0	0	0	0	0	0	1	1	1	0	1	1	0	0	0	1	0	1	1	0	0	0	0	0	0	0	0	0	0	0	0	1	1	0	0	0	0	0	0	0	0	0	0	0	0	1	1	0	0	0	1	0	0	0	0	1	0	1	1	1	1	1	0	0	0	0	1	0	1	0	1	0	1
seedMaxEhybrid-2.seedMinPu0.01	40	0	0	0	1	1	1	0	1	0	1	1	0	0	1	1	0	1	0	0	0	0	1	1	1	0	0	1	1	0	0	0	0	0	0	0	1	1	1	0	1	1	0	0	0	1	0	1	1	0	0	0	0	0	0	1	0	0	0	0	0	1	1	0	0	0	0	0	0	0	0	0	1	0	0	1	1	0	0	0	1	0	1	0	0	0	0	0	1	1	1	1	1	1	0	0	1	0	1	0	1	0	1
seedMaxEhybrid-2.seedMinPu0.001	37	0	0	0	1	1	1	0	1	0	1	1	0	0	1	1	0	1	0	0	0	0	1	1	1	0	0	1	1	0	0	0	0	0	0	0	1	1	1	0	1	1	0	0	0	1	0	1	1	0	0	0	0	0	0	0	0	0	0	0	0	1	1	0	0	0	0	0	0	0	0	0	0	0	0	1	1	0	0	0	1	0	1	0	0	1	0	0	1	1	1	1	0	0	0	0	1	0	1	0	1	0	1
seedMaxEhybrid-2.seedMinPu0.0001	37	0	0	0	1	1	1	0	1	0	1	1	0	0	1	1	0	1	0	0	0	0	1	1	1	0	0	1	1	0	0	0	0	0	0	0	1	1	1	0	1	1	0	0	0	1	0	1	1	0	0	0	0	0	0	0	0	0	0	0	0	1	1	0	0	0	0	0	0	0	0	0	0	0	0	1	1	0	0	0	1	0	0	0	0	1	0	1	1	1	1	1	0	0	0	0	1	0	1	0	1	0	1
seedMaxEhybrid-2.seedMinPu0.1	25	0	1	0	1	1	1	1	0	0	0	0	0	0	1	1	0	0	0	0	0	0	1	0	1	0	0	1	0	0	0	0	0	1	0	1	0	0	1	1	1	1	0	0	0	0	0	0	0	0	0	0	0	0	0	0	0	0	0	0	0	0	1	0	0	0	0	0	0	1	0	0	0	0	0	0	0	0	1	0	0	0	0	0	0	0	0	1	1	0	0	0	1	0	0	0	1	0	0	0	1	1	0
seedMaxEhybrid-2.seedMinPu0.001.seedNoGU	49	0	0	0	1	1	1	0	1	0	1	1	0	0	1	1	0	0	0	0	0	0	1	0	1	0	0	1	1	0	0	0	0	1	0	1	1	1	1	1	0	1	0	0	0	1	0	1	0	0	1	0	1	0	0	1	0	1	0	1	0	1	0	0	0	0	1	0	0	0	0	0	1	1	1	1	1	0	0	1	1	0	1	0	0	0	1	1	1	1	1	1	1	1	0	1	1	0	1	1	1	0	0
seedMaxEhybrid-2.seedMinPu0.1.seedNoGU	25	0	1	0	1	1	1	1	0	0	0	0	1	0	1	1	0	0	0	0	0	0	1	0	1	0	1	1	0	0	0	0	1	1	0	0	0	0	1	1	0	1	0	0	0	0	0	0	0	0	0	0	0	0	0	0	0	0	0	0	0	0	0	0	0	0	0	1	0	1	0	0	0	0	0	0	0	0	1	0	0	0	0	0	0	0	0	0	1	0	0	0	1	0	0	0	1	0	0	0	1	1	0
seedMaxEhybrid-2.seedMinPu0.0001.seedNoGU	47	0	0	0	1	1	1	0	1	0	1	1	0	0	1	1	0	0	0	0	0	0	1	0	1	0	0	1	1	0	0	0	0	1	0	1	1	1	1	1	0	1	0	0	0	1	0	1	0	0	1	0	1	0	0	1	0	1	0	1	0	1	0	0	0	0	1	0	0	0	0	0	1	1	0	1	1	0	0	0	1	0	1	0	0	1	1	1	1	1	1	1	0	1	0	1	1	0	1	1	1	0	0
seedMaxEhybrid-2.seedMinPu0.01.seedNoGU	47	0	1	0	1	1	1	0	1	0	1	1	0	0	1	1	0	0	0	0	0	0	1	0	1	0	0	1	1	0	0	0	0	1	0	1	1	1	1	1	0	1	0	0	0	1	0	1	0	0	1	1	0	0	0	0	0	1	0	0	0	1	0	0	0	0	1	0	0	1	0	0	1	1	1	1	1	0	0	0	1	0	1	0	0	0	1	1	1	1	1	1	1	1	0	1	1	0	1	0	1	0	0
seedMaxEhybrid-2.seedMinPu0.2	17	0	0	0	1	1	1	0	0	0	0	0	0	0	0	1	0	0	0	0	0	0	1	0	1	0	1	1	0	0	0	0	1	0	0	0	0	0	1	1	1	1	0	0	0	0	1	0	0	0	0	0	0	0	0	0	0	0	0	0	0	0	0	0	0	0	0	0	0	0	0	0	0	0	0	0	0	0	0	0	0	0	0	0	0	0	0	0	0	0	0	0	1	0	0	0	1	0	0	0	1	0	0
seedMaxEhybrid-2.seedMinPu0.2.seedNoGU	16	0	0	0	1	1	1	0	0	0	0	0	0	0	0	0	0	0	0	0	0	0	1	0	1	0	1	1	0	0	0	1	1	0	0	0	0	0	1	1	0	1	0	0	0	0	0	0	0	0	0	0	0	0	0	0	0	0	0	0	0	0	0	0	0	0	0	0	0	0	0	0	0	0	0	0	0	0	0	0	0	0	0	0	1	0	0	0	0	0	0	0	1	0	0	0	1	0	0	0	1	0	0
seedMaxEhybrid-2.seedMinPu0.5	6	0	0	0	0	0	0	0	0	0	0	0	0	0	0	0	0	0	0	0	0	1	1	0	1	0	0	0	0	0	0	0	0	0	0	0	0	0	0	0	0	1	0	0	0	0	0	0	0	0	0	0	0	0	0	0	0	0	0	0	0	0	0	0	0	0	0	0	0	0	0	0	0	0	0	1	0	0	0	0	0	0	0	0	1	0	0	0	0	0	0	0	0	0	0	0	0	0	0	0	0	0	0
seedMaxEhybrid-2.seedMinPu0.5.seedNoGU	5	0	0	0	0	0	0	0	0	0	0	0	0	0	0	0	0	0	0	0	0	0	1	0	1	0	0	0	0	0	0	0	0	0	0	0	0	0	0	0	0	1	0	0	0	0	0	0	0	0	0	0	0	0	0	0	0	0	0	0	0	0	0	0	0	0	0	0	0	0	0	0	0	0	0	0	0	0	0	0	0	0	0	0	1	0	0	1	0	0	0	0	0	0	0	0	0	0	0	0	0	0	0
seedMaxEhybrid-2.seedMinPu0.seedNoGU	47	0	0	0	1	1	1	0	1	0	1	1	0	0	1	1	0	0	0	0	0	0	1	0	1	0	0	1	1	0	0	0	0	1	0	1	1	1	1	1	0	1	0	0	0	1	0	1	0	0	1	0	1	0	0	1	0	1	0	1	0	1	0	0	0	0	1	0	0	0	0	0	1	1	0	1	1	0	0	0	1	0	1	0	0	1	1	1	1	1	1	1	0	1	0	1	1	0	1	1	1	0	0
seedMaxEhybrid-2.seedNoGU	47	0	0	0	1	1	1	0	1	0	1	1	0	0	1	1	0	0	0	0	0	0	1	0	1	0	0	1	1	0	0	0	0	1	0	1	1	1	1	1	0	1	0	0	0	1	0	1	0	0	1	0	1	0	0	1	0	1	0	1	0	1	0	0	0	0	1	0	0	0	0	0	1	1	0	1	1	0	0	0	1	0	1	0	0	1	1	1	1	1	1	1	0	1	0	1	1	0	1	1	1	0	0

Echericha coli NC_000913

	sum	ArcZ-b1892	ArcZ-b2741	ArcZ-b3546	ChiX-b0619	ChiX-b0681	ChiX-b1737	CyaR-b0723	CyaR-b0814	CyaR-b1740	CyaR-b1824	CyaR-b2416	CyaR-b2666	CyaR-b2687	DsrA-b1237	DsrA-b2741	DsrA-b3251	FnrS-b0723	FnrS-b0755	FnrS-b0887	FnrS-b1107	FnrS-b1479	FnrS-b1531	FnrS-b1656	FnrS-b1841	FnrS-b2153	FnrS-b2303	FnrS-b2531	FnrS-b3829	FnrS-b3908	GcvB-b1040	GcvB-b1130	GcvB-b3089	GcvB-b4208	MicA-b0411	MicA-b0814	MicA-b0957	MicA-b1130	MicC-b2215	MicF-b0241	MicF-b0889	MicF-b0929	MicF-b3912	OxyS-b1892	OxyS-b2731	RprA-b1040	RprA-b1341	RprA-b2741	RybB-b0081	RybB-b0721	RybB-b0805	RybB-b1256	RybB-b2215	RybB-b2594	RyhB-b0118	RyhB-b0156	RyhB-b0288	RyhB-b0592	RyhB-b0683	RyhB-b0721	RyhB-b0723	RyhB-b0894	RyhB-b1107	RyhB-b1200	RyhB-b1452	RyhB-b1531	RyhB-b1588	RyhB-b1612	RyhB-b1656	RyhB-b1778	RyhB-b1981	RyhB-b2069	RyhB-b2155	RyhB-b2206	RyhB-b2530	RyhB-b3365	RyhB-b3607	RyhB-b3942	RyhB-b4070	RyhB-b4122	SgrS-b1101	SgrS-b1817	SgrS-b2416	Spot42-b0039	Spot42-b0720	Spot42-b0721	Spot42-b0728	Spot42-b0757	Spot42-b1136	Spot42-b1302	Spot42-b1398	Spot42-b1761	Spot42-b1901	Spot42-b2221	Spot42-b2702	Spot42-b2715	Spot42-b2801	Spot42-b2802	Spot42-b3224	Spot42-b3566	Spot42-b3927	Spot42-b3962	Spot42-b4311
Target found among top-100 predictions for the sRNA for any tested parameterization	86	0	1	0	1	1	1	1	1	0	1	1	1	1	1	1	0	1	0	1	1	1	1	1	1	1	1	1	1	0	1	1	1	1	1	1	1	1	1	1	1	1	1	1	0	1	1	1	1	1	1	1	1	1	0	1	1	1	0	1	0	1	1	1	0	0	1	1	1	1	1	0	1	1	1	1	1	0	1	1	1	1	1	0	1	1	1	1	1	1	1	1	1	1	1	1	1	0	1	1	1	1	1
seedMaxEhybrid-3	36	0	0	0	1	1	1	0	1	0	1	1	0	0	1	1	0	1	0	0	0	0	1	0	1	0	0	1	1	0	0	0	0	0	0	0	1	1	1	0	1	1	0	0	0	1	0	1	1	0	0	0	0	0	0	0	0	0	0	0	0	1	1	0	0	0	0	0	0	0	0	0	0	0	0	1	1	0	0	0	1	0	0	0	0	1	0	1	1	1	1	1	0	0	0	0	1	0	1	0	1	0	1
seedMaxEhybrid-3.seedMinPu0	36	0	0	0	1	1	1	0	1	0	1	1	0	0	1	1	0	1	0	0	0	0	1	0	1	0	0	1	1	0	0	0	0	0	0	0	1	1	1	0	1	1	0	0	0	1	0	1	1	0	0	0	0	0	0	0	0	0	0	0	0	1	1	0	0	0	0	0	0	0	0	0	0	0	0	1	1	0	0	0	1	0	0	0	0	1	0	1	1	1	1	1	0	0	0	0	1	0	1	0	1	0	1
seedMaxEhybrid-3.seedMinPu0.01	40	0	0	0	1	1	1	0	1	0	1	1	0	0	1	1	0	1	0	0	0	0	1	1	1	0	0	1	1	0	0	0	0	0	0	0	1	1	1	0	1	1	0	0	0	1	0	1	1	0	0	0	0	0	0	1	0	0	0	0	0	1	1	0	0	0	0	0	0	0	0	0	1	0	0	1	1	0	0	0	1	0	1	0	0	0	0	0	1	1	1	1	1	1	0	0	1	0	1	0	1	0	1
seedMaxEhybrid-3.seedMinPu0.1	25	0	1	0	1	1	1	1	0	0	0	0	0	0	1	1	0	0	0	0	0	0	0	0	1	0	1	1	0	0	0	0	0	1	0	1	0	0	1	1	1	1	0	0	0	0	0	0	0	0	0	0	0	0	0	0	0	0	0	0	0	0	1	0	0	0	0	0	0	1	0	0	0	0	0	0	0	0	1	0	0	0	0	0	0	0	0	1	1	0	0	0	1	0	0	0	1	0	0	0	1	1	0
seedMaxEhybrid-3.seedMinPu0.001	37	0	0	0	1	1	1	0	1	0	1	1	0	0	1	1	0	1	0	0	0	0	1	1	1	0	0	1	1	0	0	0	0	0	0	0	1	1	1	0	1	1	0	0	0	1	0	1	1	0	0	0	0	0	0	0	0	0	0	0	0	1	1	0	0	0	0	0	0	0	0	0	0	0	0	1	1	0	0	0	1	0	1	0	0	1	0	0	1	1	1	1	0	0	0	0	1	0	1	0	1	0	1
seedMaxEhybrid-3.seedMinPu0.0001	37	0	0	0	1	1	1	0	1	0	1	1	0	0	1	1	0	1	0	0	0	0	1	1	1	0	0	1	1	0	0	0	0	0	0	0	1	1	1	0	1	1	0	0	0	1	0	1	1	0	0	0	0	0	0	0	0	0	0	0	0	1	1	0	0	0	0	0	0	0	0	0	0	0	0	1	1	0	0	0	1	0	0	0	0	1	0	1	1	1	1	1	0	0	0	0	1	0	1	0	1	0	1
seedMaxEhybrid-3.seedMinPu0.001.seedNoGU	48	0	0	0	1	1	1	0	1	0	1	1	0	0	1	1	0	0	0	0	0	0	0	0	1	0	0	1	1	0	0	0	0	1	0	1	1	1	1	1	0	1	0	0	0	1	0	1	0	0	1	0	1	0	0	1	0	1	0	1	0	1	0	0	0	0	1	0	0	0	0	0	1	1	1	1	1	0	0	1	1	0	1	0	0	0	1	1	1	1	1	1	1	1	0	1	1	0	1	1	1	0	0
seedMaxEhybrid-3.seedMinPu0.01.seedNoGU	46	0	1	0	1	1	1	0	1	0	1	1	0	0	1	1	0	0	0	0	0	0	0	0	1	0	0	1	1	0	0	0	0	1	0	1	1	1	1	1	0	1	0	0	0	1	0	1	0	0	1	1	0	0	0	0	0	1	0	0	0	1	0	0	0	0	1	0	0	1	0	0	1	1	1	1	1	0	0	0	1	0	1	0	0	0	1	1	1	1	1	1	1	1	0	1	1	0	1	0	1	0	0
seedMaxEhybrid-3.seedMinPu0.1.seedNoGU	24	0	1	0	1	1	1	1	0	0	0	0	1	0	1	1	0	0	0	0	0	0	0	0	1	0	1	1	0	0	0	0	1	1	0	0	0	0	1	1	0	1	0	0	0	0	0	0	0	0	0	0	0	0	0	0	0	0	0	0	0	0	0	0	0	0	0	1	0	1	0	0	0	0	0	0	0	0	1	0	0	0	0	0	0	0	0	0	1	0	0	0	1	0	0	0	1	0	0	0	1	1	0
seedMaxEhybrid-3.seedMinPu0.0001.seedNoGU	46	0	0	0	1	1	1	0	1	0	1	1	0	0	1	1	0	0	0	0	0	0	0	0	1	0	0	1	1	0	0	0	0	1	0	1	1	1	1	1	0	1	0	0	0	1	0	1	0	0	1	0	1	0	0	1	0	1	0	1	0	1	0	0	0	0	1	0	0	0	0	0	1	1	0	1	1	0	0	0	1	0	1	0	0	1	1	1	1	1	1	1	0	1	0	1	1	0	1	1	1	0	0
seedMaxEhybrid-3.seedMinPu0.2	15	0	0	0	1	1	1	0	0	0	0	0	0	0	0	1	0	0	0	0	0	0	0	0	1	0	1	1	0	0	0	0	1	0	0	0	0	0	1	1	0	1	0	0	0	0	1	0	0	0	0	0	0	0	0	0	0	0	0	0	0	0	0	0	0	0	0	0	0	0	0	0	0	0	0	0	0	0	0	0	0	0	0	0	0	0	0	0	0	0	0	0	1	0	0	0	1	0	0	0	1	0	0
seedMaxEhybrid-3.seedMinPu0.2.seedNoGU	14	0	0	0	1	1	1	0	0	0	0	0	0	0	0	0	0	0	0	0	0	0	0	0	1	0	1	1	0	0	0	1	1	0	0	0	0	0	1	1	0	1	0	0	0	0	0	0	0	0	0	0	0	0	0	0	0	0	0	0	0	0	0	0	0	0	0	0	0	0	0	0	0	0	0	0	0	0	0	0	0	0	0	0	0	0	0	0	0	0	0	0	1	0	0	0	1	0	0	0	1	0	0
seedMaxEhybrid-3.seedMinPu0.5	1	0	0	0	0	0	0	0	0	0	0	0	0	0	0	0	0	0	0	0	0	0	0	0	1	0	0	0	0	0	0	0	0	0	0	0	0	0	0	0	0	0	0	0	0	0	0	0	0	0	0	0	0	0	0	0	0	0	0	0	0	0	0	0	0	0	0	0	0	0	0	0	0	0	0	0	0	0	0	0	0	0	0	0	0	0	0	0	0	0	0	0	0	0	0	0	0	0	0	0	0	0	0
seedMaxEhybrid-3.seedMinPu0.5.seedNoGU	1	0	0	0	0	0	0	0	0	0	0	0	0	0	0	0	0	0	0	0	0	0	0	0	1	0	0	0	0	0	0	0	0	0	0	0	0	0	0	0	0	0	0	0	0	0	0	0	0	0	0	0	0	0	0	0	0	0	0	0	0	0	0	0	0	0	0	0	0	0	0	0	0	0	0	0	0	0	0	0	0	0	0	0	0	0	0	0	0	0	0	0	0	0	0	0	0	0	0	0	0	0	0
seedMaxEhybrid-3.seedMinPu0.seedNoGU	46	0	0	0	1	1	1	0	1	0	1	1	0	0	1	1	0	0	0	0	0	0	0	0	1	0	0	1	1	0	0	0	0	1	0	1	1	1	1	1	0	1	0	0	0	1	0	1	0	0	1	0	1	0	0	1	0	1	0	1	0	1	0	0	0	0	1	0	0	0	0	0	1	1	0	1	1	0	0	0	1	0	1	0	0	1	1	1	1	1	1	1	0	1	0	1	1	0	1	1	1	0	0
seedMaxEhybrid-3.seedNoGU	46	0	0	0	1	1	1	0	1	0	1	1	0	0	1	1	0	0	0	0	0	0	0	0	1	0	0	1	1	0	0	0	0	1	0	1	1	1	1	1	0	1	0	0	0	1	0	1	0	0	1	0	1	0	0	1	0	1	0	1	0	1	0	0	0	0	1	0	0	0	0	0	1	1	0	1	1	0	0	0	1	0	1	0	0	1	1	1	1	1	1	1	0	1	0	1	1	0	1	1	1	0	0
seedMaxEhybrid-4	36	0	0	0	1	1	1	0	1	0	1	1	0	0	1	1	0	0	0	0	0	0	1	1	1	0	0	1	1	0	0	0	0	0	0	0	1	1	1	0	1	1	0	0	0	1	0	1	1	0	0	0	0	0	0	0	0	0	0	0	0	1	1	0	0	0	0	0	0	0	0	0	0	0	0	1	1	0	0	0	1	0	0	0	0	1	0	1	1	1	1	1	0	0	0	0	1	0	1	0	1	0	1
seedMaxEhybrid-4.seedMinPu0	36	0	0	0	1	1	1	0	1	0	1	1	0	0	1	1	0	0	0	0	0	0	1	1	1	0	0	1	1	0	0	0	0	0	0	0	1	1	1	0	1	1	0	0	0	1	0	1	1	0	0	0	0	0	0	0	0	0	0	0	0	1	1	0	0	0	0	0	0	0	0	0	0	0	0	1	1	0	0	0	1	0	0	0	0	1	0	1	1	1	1	1	0	0	0	0	1	0	1	0	1	0	1
seedMaxEhybrid-4.seedMinPu0.01	39	0	0	0	1	1	1	0	1	0	1	1	0	0	1	1	0	0	0	0	0	0	1	1	1	0	0	1	1	0	0	0	0	0	0	0	1	1	1	0	1	1	0	0	0	1	0	1	1	0	0	0	0	0	0	1	0	0	0	0	0	1	1	0	0	0	0	0	0	0	0	0	1	0	0	1	1	0	0	0	1	0	1	0	0	0	0	0	1	1	1	1	1	1	0	0	1	0	1	0	1	0	1
seedMaxEhybrid-4.seedMinPu0.0001	36	0	0	0	1	1	1	0	1	0	1	1	0	0	1	1	0	0	0	0	0	0	1	1	1	0	0	1	1	0	0	0	0	0	0	0	1	1	1	0	1	1	0	0	0	1	0	1	1	0	0	0	0	0	0	0	0	0	0	0	0	1	1	0	0	0	0	0	0	0	0	0	0	0	0	1	1	0	0	0	1	0	0	0	0	1	0	1	1	1	1	1	0	0	0	0	1	0	1	0	1	0	1
seedMaxEhybrid-4.seedMinPu0.001	37	0	0	0	1	1	1	0	1	0	1	1	0	0	1	1	0	0	0	0	0	0	1	1	1	0	0	1	1	0	0	0	0	0	0	0	1	1	1	0	1	1	0	0	0	1	0	1	1	0	0	0	0	0	0	0	0	0	0	0	0	1	1	0	0	0	0	0	0	0	0	0	0	0	0	1	1	0	0	0	1	0	1	0	0	1	0	0	1	1	1	1	0	1	0	0	1	0	1	0	1	0	1
seedMaxEhybrid-4.seedMinPu0.1	25	0	1	0	1	1	1	1	0	0	0	0	0	0	1	1	0	0	0	0	0	0	0	0	1	0	1	1	0	0	0	0	0	1	0	1	0	0	1	1	1	1	0	0	0	0	0	0	0	0	0	0	0	0	0	0	0	0	0	0	0	0	1	0	0	0	0	0	0	1	0	0	0	0	0	0	0	0	1	0	0	0	0	0	0	0	0	1	1	0	0	0	1	0	0	0	1	0	0	0	1	1	0
seedMaxEhybrid-4.seedMinPu0.01.seedNoGU	47	0	1	0	1	1	1	0	1	0	1	1	0	0	1	1	0	0	0	0	0	0	0	0	1	0	0	1	1	0	0	1	0	1	0	1	1	1	1	1	0	1	0	0	0	1	0	1	0	0	1	1	0	0	0	0	0	1	0	0	0	1	0	0	0	0	1	0	0	1	0	0	1	1	1	1	1	0	0	0	1	0	1	0	0	0	1	1	1	1	1	1	1	1	0	1	1	0	1	0	1	0	0
seedMaxEhybrid-4.seedMinPu0.001.seedNoGU	48	0	0	0	1	1	1	0	1	0	1	1	0	0	1	1	0	0	0	0	0	0	0	0	1	0	0	1	1	0	0	0	0	1	0	1	1	1	1	1	0	1	0	0	0	1	0	1	0	0	1	0	1	0	0	1	0	1	0	1	0	1	0	0	0	0	1	0	0	0	0	0	1	1	1	1	1	0	0	1	1	0	1	0	0	0	1	1	1	1	1	1	1	1	0	1	1	0	1	1	1	0	0
seedMaxEhybrid-4.seedMinPu0.1.seedNoGU	25	0	1	0	1	1	1	1	0	0	0	0	1	0	1	1	0	0	0	0	0	0	0	0	1	0	1	1	0	0	0	0	1	1	0	0	0	0	1	1	0	1	0	0	0	0	0	0	0	0	0	0	0	0	0	0	0	0	0	0	0	0	0	0	0	0	0	1	0	1	0	0	0	0	0	0	0	0	1	0	0	1	0	0	0	0	0	1	0	0	0	1	0	0	0	1	0	0	0	1	1	0
seedMaxEhybrid-4.seedMinPu0.0001.seedNoGU	46	0	0	0	1	1	1	0	1	0	1	1	0	0	1	1	0	0	0	0	0	0	0	0	1	0	0	1	1	0	0	0	0	1	0	1	1	1	1	1	0	1	0	0	0	1	0	1	0	0	1	0	1	0	0	1	0	1	0	1	0	1	0	0	0	0	1	0	0	0	0	0	1	1	0	1	1	0	0	0	1	0	1	0	0	1	1	1	1	1	1	1	0	1	0	1	1	0	1	1	1	0	0
seedMaxEhybrid-4.seedMinPu0.2	14	0	0	0	1	1	1	0	0	0	0	0	0	0	0	1	0	0	0	0	0	0	0	0	1	0	1	1	0	0	0	0	1	0	0	0	0	0	0	1	0	1	0	0	0	0	1	0	0	0	0	0	0	0	0	0	0	0	0	0	0	0	0	0	0	0	0	0	0	0	0	0	0	0	0	0	0	0	0	0	0	0	0	0	0	0	0	0	0	0	0	0	1	0	0	0	1	0	0	0	1	0	0
seedMaxEhybrid-4.seedMinPu0.2.seedNoGU	13	0	0	0	1	1	1	0	0	0	0	0	0	0	0	0	0	0	0	0	0	0	0	0	1	0	1	1	0	0	0	1	1	0	0	0	0	0	0	1	0	1	0	0	0	0	0	0	0	0	0	0	0	0	0	0	0	0	0	0	0	0	0	0	0	0	0	0	0	0	0	0	0	0	0	0	0	0	0	0	0	0	0	0	0	0	0	0	0	0	0	0	1	0	0	0	1	0	0	0	1	0	0
seedMaxEhybrid-4.seedMinPu0.5	1	0	0	0	0	0	0	0	0	0	0	0	0	0	0	0	0	0	0	0	0	0	0	0	1	0	0	0	0	0	0	0	0	0	0	0	0	0	0	0	0	0	0	0	0	0	0	0	0	0	0	0	0	0	0	0	0	0	0	0	0	0	0	0	0	0	0	0	0	0	0	0	0	0	0	0	0	0	0	0	0	0	0	0	0	0	0	0	0	0	0	0	0	0	0	0	0	0	0	0	0	0	0
seedMaxEhybrid-4.seedMinPu0.5.seedNoGU	1	0	0	0	0	0	0	0	0	0	0	0	0	0	0	0	0	0	0	0	0	0	0	0	1	0	0	0	0	0	0	0	0	0	0	0	0	0	0	0	0	0	0	0	0	0	0	0	0	0	0	0	0	0	0	0	0	0	0	0	0	0	0	0	0	0	0	0	0	0	0	0	0	0	0	0	0	0	0	0	0	0	0	0	0	0	0	0	0	0	0	0	0	0	0	0	0	0	0	0	0	0	0
seedMaxEhybrid-4.seedMinPu0.seedNoGU	46	0	0	0	1	1	1	0	1	0	1	1	0	0	1	1	0	0	0	0	0	0	0	0	1	0	0	1	1	0	0	0	0	1	0	1	1	1	1	1	0	1	0	0	0	1	0	1	0	0	1	0	1	0	0	1	0	1	0	1	0	1	0	0	0	0	1	0	0	0	0	0	1	1	0	1	1	0	0	0	1	0	1	0	0	1	1	1	1	1	1	1	0	1	0	1	1	0	1	1	1	0	0
seedMaxEhybrid-4.seedNoGU	46	0	0	0	1	1	1	0	1	0	1	1	0	0	1	1	0	0	0	0	0	0	0	0	1	0	0	1	1	0	0	0	0	1	0	1	1	1	1	1	0	1	0	0	0	1	0	1	0	0	1	0	1	0	0	1	0	1	0	1	0	1	0	0	0	0	1	0	0	0	0	0	1	1	0	1	1	0	0	0	1	0	1	0	0	1	1	1	1	1	1	1	0	1	0	1	1	0	1	1	1	0	0
seedMaxEhybrid-5	36	0	0	0	1	1	1	0	1	0	1	1	0	0	1	1	0	0	0	0	0	0	1	1	1	0	0	1	1	0	0	0	0	0	0	0	1	1	1	0	1	1	0	0	0	1	0	1	1	0	0	0	0	0	0	0	0	0	0	0	0	1	1	0	0	0	0	0	0	0	0	0	0	0	0	1	1	0	0	0	1	0	0	0	0	1	0	1	1	1	1	1	0	0	0	0	1	0	1	0	1	0	1
seedMaxEhybrid-5.seedMinPu0	36	0	0	0	1	1	1	0	1	0	1	1	0	0	1	1	0	0	0	0	0	0	1	1	1	0	0	1	1	0	0	0	0	0	0	0	1	1	1	0	1	1	0	0	0	1	0	1	1	0	0	0	0	0	0	0	0	0	0	0	0	1	1	0	0	0	0	0	0	0	0	0	0	0	0	1	1	0	0	0	1	0	0	0	0	1	0	1	1	1	1	1	0	0	0	0	1	0	1	0	1	0	1
seedMaxEhybrid-5.seedMinPu0.0001	36	0	0	0	1	1	1	0	1	0	1	1	0	0	1	1	0	0	0	0	0	0	1	1	1	0	0	1	1	0	0	0	0	0	0	0	1	1	1	0	1	1	0	0	0	1	0	1	1	0	0	0	0	0	0	0	0	0	0	0	0	1	1	0	0	0	0	0	0	0	0	0	0	0	0	1	1	0	0	0	1	0	0	0	0	1	0	1	1	1	1	1	0	0	0	0	1	0	1	0	1	0	1
seedMaxEhybrid-5.seedMinPu0.01	40	0	0	0	1	1	1	0	1	0	1	1	0	0	1	1	0	0	0	0	0	0	1	1	1	0	0	1	1	0	0	0	0	0	0	0	1	1	1	0	1	1	0	0	0	1	0	1	1	0	0	0	0	0	0	1	0	0	0	0	0	1	1	0	0	0	0	0	0	0	0	0	1	0	0	1	1	0	0	0	1	0	1	0	0	0	0	1	1	1	1	1	1	1	0	0	1	0	1	0	1	0	1
seedMaxEhybrid-5.seedMinPu0.1	27	0	1	0	1	1	1	1	0	0	0	0	1	0	0	1	0	0	0	0	0	0	0	0	1	0	1	1	0	0	0	0	0	1	0	1	0	0	1	1	1	1	0	0	0	0	1	0	0	0	0	0	0	0	0	0	0	0	0	0	0	0	1	0	0	0	0	1	0	1	0	0	0	0	0	0	0	0	1	0	0	0	0	0	0	0	0	1	1	0	0	0	1	0	0	0	1	0	0	0	1	1	0
seedMaxEhybrid-5.seedMinPu0.001	38	0	0	0	1	1	1	0	1	0	1	1	0	0	1	1	0	0	0	0	0	0	1	1	1	0	0	1	1	0	0	0	0	0	0	0	1	1	1	0	1	1	0	0	0	1	0	1	1	0	0	0	1	0	0	0	0	0	0	0	0	1	1	0	0	0	0	0	0	0	0	0	0	0	0	1	1	0	0	0	1	0	1	0	0	1	0	0	1	1	1	1	0	1	0	0	1	0	1	0	1	0	1
seedMaxEhybrid-5.seedMinPu0.01.seedNoGU	48	0	1	0	1	1	1	0	1	0	1	1	0	0	1	1	0	0	0	0	0	0	0	0	1	0	0	1	1	0	0	1	0	1	0	1	1	1	1	1	0	1	1	0	0	1	0	1	0	0	1	1	0	0	0	0	0	1	0	0	0	1	0	0	0	0	1	0	0	1	0	0	1	1	1	1	1	0	0	0	1	0	1	0	0	0	1	1	1	1	1	1	1	1	0	1	1	0	1	0	1	0	0
seedMaxEhybrid-5.seedMinPu0.0001.seedNoGU	46	0	0	0	1	1	1	0	1	0	1	1	0	0	1	1	0	0	0	0	0	0	0	0	1	0	0	1	1	0	0	0	0	1	0	1	1	1	1	1	0	1	0	0	0	1	0	1	0	0	1	0	1	0	0	1	0	1	0	1	0	1	0	0	0	0	1	0	0	0	0	0	1	1	0	1	1	0	0	0	1	0	1	0	0	1	1	1	1	1	1	1	0	1	0	1	1	0	1	1	1	0	0
seedMaxEhybrid-5.seedMinPu0.1.seedNoGU	24	0	1	0	1	1	1	1	0	0	0	0	1	0	0	1	0	0	0	0	0	0	0	0	1	0	1	1	0	0	0	0	1	1	0	0	0	0	1	1	0	1	0	0	0	0	0	0	0	0	0	0	0	0	0	0	0	0	0	0	0	0	0	0	0	0	0	1	0	1	0	0	0	0	0	0	0	0	1	0	0	1	0	0	0	0	0	1	0	0	0	1	0	0	0	1	0	0	0	1	1	0
seedMaxEhybrid-5.seedMinPu0.001.seedNoGU	48	0	0	0	1	1	1	0	1	0	1	1	0	0	1	1	0	0	0	0	0	0	0	0	1	0	0	1	1	0	0	0	0	1	0	1	1	1	1	1	0	1	0	0	0	1	0	1	0	0	1	0	1	0	0	1	0	1	0	1	0	1	0	0	0	0	1	0	0	0	0	0	1	1	1	1	1	0	0	1	1	0	1	0	0	0	1	1	1	1	1	1	1	1	0	1	1	0	1	1	1	0	0
seedMaxEhybrid-5.seedMinPu0.2	14	0	0	0	1	1	1	0	0	0	0	0	0	0	0	1	0	0	0	0	0	0	0	0	1	0	1	1	0	0	0	0	1	0	0	0	0	0	0	1	0	1	0	0	0	0	1	0	0	0	0	0	0	0	0	0	0	0	0	0	0	0	0	0	0	0	0	0	0	0	0	0	0	0	0	0	0	0	0	0	0	0	0	0	0	0	0	0	0	0	0	0	1	0	0	0	1	0	0	0	1	0	0
seedMaxEhybrid-5.seedMinPu0.2.seedNoGU	13	0	0	0	1	1	1	0	0	0	0	0	0	0	0	0	0	0	0	0	0	0	0	0	1	0	1	1	0	0	0	1	1	0	0	0	0	0	0	1	0	1	0	0	0	0	0	0	0	0	0	0	0	0	0	0	0	0	0	0	0	0	0	0	0	0	0	0	0	0	0	0	0	0	0	0	0	0	0	0	0	0	0	0	0	0	0	0	0	0	0	0	1	0	0	0	1	0	0	0	1	0	0
seedMaxEhybrid-5.seedMinPu0.5	1	0	0	0	0	0	0	0	0	0	0	0	0	0	0	0	0	0	0	0	0	0	0	0	1	0	0	0	0	0	0	0	0	0	0	0	0	0	0	0	0	0	0	0	0	0	0	0	0	0	0	0	0	0	0	0	0	0	0	0	0	0	0	0	0	0	0	0	0	0	0	0	0	0	0	0	0	0	0	0	0	0	0	0	0	0	0	0	0	0	0	0	0	0	0	0	0	0	0	0	0	0	0
seedMaxEhybrid-5.seedMinPu0.5.seedNoGU	1	0	0	0	0	0	0	0	0	0	0	0	0	0	0	0	0	0	0	0	0	0	0	0	1	0	0	0	0	0	0	0	0	0	0	0	0	0	0	0	0	0	0	0	0	0	0	0	0	0	0	0	0	0	0	0	0	0	0	0	0	0	0	0	0	0	0	0	0	0	0	0	0	0	0	0	0	0	0	0	0	0	0	0	0	0	0	0	0	0	0	0	0	0	0	0	0	0	0	0	0	0	0
seedMaxEhybrid-5.seedMinPu0.seedNoGU	46	0	0	0	1	1	1	0	1	0	1	1	0	0	1	1	0	0	0	0	0	0	0	0	1	0	0	1	1	0	0	0	0	1	0	1	1	1	1	1	0	1	0	0	0	1	0	1	0	0	1	0	1	0	0	1	0	1	0	1	0	1	0	0	0	0	1	0	0	0	0	0	1	1	0	1	1	0	0	0	1	0	1	0	0	1	1	1	1	1	1	1	0	1	0	1	1	0	1	1	1	0	0
seedMaxEhybrid-5.seedNoGU	46	0	0	0	1	1	1	0	1	0	1	1	0	0	1	1	0	0	0	0	0	0	0	0	1	0	0	1	1	0	0	0	0	1	0	1	1	1	1	1	0	1	0	0	0	1	0	1	0	0	1	0	1	0	0	1	0	1	0	1	0	1	0	0	0	0	1	0	0	0	0	0	1	1	0	1	1	0	0	0	1	0	1	0	0	1	1	1	1	1	1	1	0	1	0	1	1	0	1	1	1	0	0
seedMaxEhybrid-6	35	0	0	0	1	1	1	0	1	0	1	1	0	0	1	1	0	0	0	0	0	0	0	1	1	0	0	1	1	0	0	0	0	0	0	0	1	1	1	0	1	1	0	0	0	1	0	0	1	0	0	0	1	0	0	0	0	0	0	0	0	1	1	0	0	0	0	0	0	0	0	0	0	0	0	1	1	0	0	0	1	0	0	0	0	1	0	1	1	1	1	1	0	0	0	0	1	0	1	0	1	0	1
seedMaxEhybrid-6.seedMinPu0	35	0	0	0	1	1	1	0	1	0	1	1	0	0	1	1	0	0	0	0	0	0	0	1	1	0	0	1	1	0	0	0	0	0	0	0	1	1	1	0	1	1	0	0	0	1	0	0	1	0	0	0	1	0	0	0	0	0	0	0	0	1	1	0	0	0	0	0	0	0	0	0	0	0	0	1	1	0	0	0	1	0	0	0	0	1	0	1	1	1	1	1	0	0	0	0	1	0	1	0	1	0	1
seedMaxEhybrid-6.seedMinPu0.1	22	0	0	0	1	1	1	0	0	0	0	0	1	0	0	1	0	0	0	0	0	0	0	0	1	0	1	1	0	0	0	0	0	1	0	0	0	0	0	0	1	1	0	0	0	0	1	0	0	0	0	0	0	0	0	0	0	0	0	0	0	0	1	0	0	0	0	1	0	1	0	0	0	0	0	0	0	0	1	0	0	0	0	0	0	0	0	1	1	0	0	0	1	0	0	0	1	0	0	0	1	1	0
seedMaxEhybrid-6.seedMinPu0.0001	35	0	0	0	1	1	1	0	1	0	1	1	0	0	1	1	0	0	0	0	0	0	0	1	1	0	0	1	1	0	0	0	0	0	0	0	1	1	1	0	1	1	0	0	0	1	0	0	1	0	0	0	1	0	0	0	0	0	0	0	0	1	1	0	0	0	0	0	0	0	0	0	0	0	0	1	1	0	0	0	1	0	0	0	0	1	0	1	1	1	1	1	0	0	0	0	1	0	1	0	1	0	1
seedMaxEhybrid-6.seedMinPu0.001	36	0	0	0	1	1	1	0	1	0	1	1	0	0	1	1	0	0	0	0	0	0	0	1	1	0	0	1	1	0	0	0	0	0	0	0	0	1	1	0	1	1	0	0	0	1	0	0	1	0	0	0	1	0	0	0	0	0	0	0	0	1	1	0	0	0	0	0	0	0	0	0	0	0	0	1	1	0	0	0	1	0	1	0	0	1	0	0	1	1	1	1	1	1	0	0	1	0	1	0	1	0	1
seedMaxEhybrid-6.seedMinPu0.01	35	0	0	0	1	1	1	0	1	0	1	1	0	0	1	0	0	0	0	0	0	0	0	1	1	0	0	1	0	0	0	0	0	0	0	1	0	1	1	0	1	1	0	0	0	1	0	0	1	0	0	0	0	0	0	0	0	0	0	0	0	1	1	0	0	0	0	0	0	0	0	0	1	0	0	1	1	0	0	0	1	0	1	0	0	0	0	1	1	1	1	1	1	1	0	0	1	0	1	0	1	0	1
seedMaxEhybrid-6.seedMinPu0.0001.seedNoGU	45	0	0	0	1	1	1	0	1	0	1	1	0	0	1	1	0	0	0	0	0	0	0	0	1	0	0	1	0	0	0	0	0	1	0	1	1	1	1	1	0	1	0	0	0	1	0	0	0	0	1	1	1	0	0	1	0	1	0	1	0	1	0	0	0	0	1	0	0	0	0	0	1	1	0	1	1	0	0	0	1	0	1	0	0	1	1	1	1	1	1	1	0	1	0	1	1	0	1	1	1	0	0
seedMaxEhybrid-6.seedMinPu0.01.seedNoGU	47	0	1	0	1	1	1	1	1	0	1	1	0	0	1	0	0	0	0	1	0	0	0	0	1	0	0	1	0	0	0	1	0	1	0	1	0	1	1	1	0	1	1	1	0	1	0	0	0	0	1	1	0	0	0	0	0	1	0	0	0	1	0	0	0	0	1	0	0	1	0	0	1	1	1	1	1	0	0	0	1	0	1	0	0	0	1	1	1	1	1	1	1	1	0	1	1	0	1	0	1	0	0
seedMaxEhybrid-6.seedMinPu0.1.seedNoGU	20	0	0	0	1	1	1	0	0	0	0	0	1	0	0	0	0	0	0	0	0	0	0	0	1	0	1	1	0	0	0	0	1	1	1	0	0	0	0	0	0	1	0	0	0	0	0	0	0	0	0	0	0	0	0	0	0	0	0	0	0	0	0	0	0	0	0	1	0	1	0	0	0	0	0	0	0	0	1	0	0	1	0	0	0	0	0	1	0	0	0	1	0	0	0	1	0	0	0	1	1	0

Echericha coli NC_000913

	sum	ArcZ-b1892	ArcZ-b2741	ArcZ-b3546	ChiX-b0619	ChiX-b0681	ChiX-b1737	CyaR-b0723	CyaR-b0814	CyaR-b1740	CyaR-b1824	CyaR-b2416	CyaR-b2666	CyaR-b2687	DsrA-b1237	DsrA-b2741	DsrA-b3251	FnrS-b0723	FnrS-b0755	FnrS-b0887	FnrS-b1107	FnrS-b1479	FnrS-b1531	FnrS-b1656	FnrS-b1841	FnrS-b2153	FnrS-b2303	FnrS-b2531	FnrS-b3829	FnrS-b3908	GcvB-b1040	GcvB-b1130	GcvB-b3089	GcvB-b4208	MicA-b0411	MicA-b0814	MicA-b0957	MicA-b1130	MicC-b2215	MicF-b0241	MicF-b0889	MicF-b0929	MicF-b3912	OxyS-b1892	OxyS-b2731	RprA-b1040	RprA-b1341	RprA-b2741	RybB-b0081	RybB-b0721	RybB-b0805	RybB-b1256	RybB-b2215	RybB-b2594	RyhB-b0118	RyhB-b0156	RyhB-b0288	RyhB-b0592	RyhB-b0683	RyhB-b0721	RyhB-b0723	RyhB-b0894	RyhB-b1107	RyhB-b1200	RyhB-b1452	RyhB-b1531	RyhB-b1588	RyhB-b1612	RyhB-b1656	RyhB-b1778	RyhB-b1981	RyhB-b2069	RyhB-b2155	RyhB-b2206	RyhB-b2530	RyhB-b3365	RyhB-b3607	RyhB-b3942	RyhB-b4070	RyhB-b4122	SgrS-b1101	SgrS-b1817	SgrS-b2416	Spot42-b0039	Spot42-b0720	Spot42-b0721	Spot42-b0728	Spot42-b0757	Spot42-b1136	Spot42-b1302	Spot42-b1398	Spot42-b1761	Spot42-b1901	Spot42-b2221	Spot42-b2702	Spot42-b2715	Spot42-b2801	Spot42-b2802	Spot42-b3224	Spot42-b3566	Spot42-b3927	Spot42-b3962	Spot42-b4311
Target found among top-100 predictions for the sRNA for any tested parameterization	86	0	1	0	1	1	1	1	1	0	1	1	1	1	1	1	0	1	0	1	1	1	1	1	1	1	1	1	1	0	1	1	1	1	1	1	1	1	1	1	1	1	1	1	0	1	1	1	1	1	1	1	1	1	0	1	1	1	0	1	0	1	1	1	0	0	1	1	1	1	1	0	1	1	1	1	1	0	1	1	1	1	1	0	1	1	1	1	1	1	1	1	1	1	1	1	1	0	1	1	1	1	1
seedMaxEhybrid-6.seedMinPu0.001.seedNoGU	48	0	0	0	1	1	1	0	1	0	1	1	0	0	1	1	0	0	0	1	0	0	0	0	1	0	0	1	0	0	0	0	0	1	0	1	0	1	1	1	0	1	1	0	0	1	0	0	0	0	1	1	1	0	0	1	0	1	0	1	0	1	0	0	0	0	1	0	0	0	0	0	1	1	1	1	1	0	0	1	1	0	1	0	0	0	1	1	1	1	1	1	1	1	0	1	1	0	1	1	1	0	0
seedMaxEhybrid-6.seedMinPu0.2	12	0	0	0	1	1	1	0	0	0	0	0	0	0	0	1	0	0	0	0	0	0	0	0	1	0	1	0	0	0	0	0	1	0	0	0	0	0	0	0	0	1	0	0	0	0	1	0	0	0	0	0	0	0	0	0	0	0	0	0	0	0	0	0	0	0	0	0	0	0	0	0	0	0	0	0	0	0	0	0	0	0	0	0	0	0	0	0	0	0	0	0	1	0	0	0	1	0	0	0	1	0	0
seedMaxEhybrid-6.seedMinPu0.2.seedNoGU	11	0	0	0	1	1	1	0	0	0	0	0	0	0	0	0	0	0	0	0	0	0	0	0	1	0	1	0	0	0	0	1	1	0	0	0	0	0	0	0	0	1	0	0	0	0	0	0	0	0	0	0	0	0	0	0	0	0	0	0	0	0	0	0	0	0	0	0	0	0	0	0	0	0	0	0	0	0	0	0	0	0	0	0	0	0	0	0	0	0	0	0	1	0	0	0	1	0	0	0	1	0	0
seedMaxEhybrid-6.seedMinPu0.5	1	0	0	0	0	0	0	0	0	0	0	0	0	0	0	0	0	0	0	0	0	0	0	0	1	0	0	0	0	0	0	0	0	0	0	0	0	0	0	0	0	0	0	0	0	0	0	0	0	0	0	0	0	0	0	0	0	0	0	0	0	0	0	0	0	0	0	0	0	0	0	0	0	0	0	0	0	0	0	0	0	0	0	0	0	0	0	0	0	0	0	0	0	0	0	0	0	0	0	0	0	0	0
seedMaxEhybrid-6.seedMinPu0.5.seedNoGU	1	0	0	0	0	0	0	0	0	0	0	0	0	0	0	0	0	0	0	0	0	0	0	0	1	0	0	0	0	0	0	0	0	0	0	0	0	0	0	0	0	0	0	0	0	0	0	0	0	0	0	0	0	0	0	0	0	0	0	0	0	0	0	0	0	0	0	0	0	0	0	0	0	0	0	0	0	0	0	0	0	0	0	0	0	0	0	0	0	0	0	0	0	0	0	0	0	0	0	0	0	0	0
seedMaxEhybrid-6.seedMinPu0.seedNoGU	45	0	0	0	1	1	1	0	1	0	1	1	0	0	1	1	0	0	0	0	0	0	0	0	1	0	0	1	0	0	0	0	0	1	0	1	1	1	1	1	0	1	0	0	0	1	0	0	0	0	1	1	1	0	0	1	0	1	0	1	0	1	0	0	0	0	1	0	0	0	0	0	1	1	0	1	1	0	0	0	1	0	1	0	0	1	1	1	1	1	1	1	0	1	0	1	1	0	1	1	1	0	0
seedMaxEhybrid-6.seedNoGU	45	0	0	0	1	1	1	0	1	0	1	1	0	0	1	1	0	0	0	0	0	0	0	0	1	0	0	1	0	0	0	0	0	1	0	1	1	1	1	1	0	1	0	0	0	1	0	0	0	0	1	1	1	0	0	1	0	1	0	1	0	1	0	0	0	0	1	0	0	0	0	0	1	1	0	1	1	0	0	0	1	0	1	0	0	1	1	1	1	1	1	1	0	1	0	1	1	0	1	1	1	0	0
seedMaxEhybrid-7	34	0	0	0	1	1	1	0	1	0	1	1	0	0	1	1	0	0	0	0	0	0	1	0	0	0	0	1	1	0	0	0	0	0	0	0	0	1	1	0	1	1	0	0	0	1	0	0	1	0	0	0	1	0	0	0	0	0	0	0	0	1	1	0	0	0	0	0	0	0	0	0	0	0	0	1	1	0	0	0	1	0	0	0	0	1	0	1	1	1	1	1	0	1	0	0	1	0	1	0	1	0	1
seedMaxEhybrid-7.seedMinPu0	34	0	0	0	1	1	1	0	1	0	1	1	0	0	1	1	0	0	0	0	0	0	1	0	0	0	0	1	1	0	0	0	0	0	0	0	0	1	1	0	1	1	0	0	0	1	0	0	1	0	0	0	1	0	0	0	0	0	0	0	0	1	1	0	0	0	0	0	0	0	0	0	0	0	0	1	1	0	0	0	1	0	0	0	0	1	0	1	1	1	1	1	0	1	0	0	1	0	1	0	1	0	1
seedMaxEhybrid-7.seedMinPu0.001	36	0	0	0	1	1	1	0	1	0	1	1	0	0	1	0	0	0	0	0	0	0	0	1	0	0	0	0	1	0	0	0	0	0	0	0	0	1	1	1	1	1	0	0	0	1	0	0	1	0	0	0	1	0	0	0	0	0	0	0	0	1	1	0	0	0	0	0	0	0	0	0	1	0	0	1	1	0	0	0	1	0	1	0	0	1	0	1	1	1	1	1	1	1	0	0	1	0	1	0	1	0	1
seedMaxEhybrid-7.seedMinPu0.1	13	0	0	0	0	0	0	0	0	0	0	0	1	0	0	0	0	0	0	0	0	0	0	0	0	0	1	0	0	0	0	0	0	1	0	0	0	0	0	0	1	1	0	0	0	0	0	0	0	0	0	0	0	0	0	0	0	0	0	0	0	0	1	0	0	0	0	0	0	1	0	0	0	0	0	0	0	0	1	0	0	0	0	0	0	0	0	1	1	0	0	0	1	0	0	0	1	0	0	0	1	0	0
seedMaxEhybrid-7.seedMinPu0.01	36	0	1	0	1	1	1	0	1	0	1	1	0	0	1	0	0	0	0	0	0	0	0	1	0	0	0	0	0	0	0	0	0	1	0	0	0	1	1	1	1	1	0	0	0	1	0	0	1	0	1	0	0	0	0	0	0	0	0	0	0	1	1	0	0	0	0	0	0	0	0	0	1	0	0	1	1	0	0	0	1	0	1	0	0	0	0	1	1	1	1	0	1	1	0	1	1	0	1	0	1	0	1
seedMaxEhybrid-7.seedMinPu0.0001	36	0	0	0	1	1	1	0	1	0	1	1	0	0	1	1	0	0	0	0	0	0	0	1	0	0	0	1	1	0	0	0	0	0	0	0	0	1	1	0	1	1	0	0	0	1	0	0	1	0	0	0	1	0	0	0	0	0	0	0	0	1	1	0	0	0	0	0	0	0	0	0	0	0	0	1	1	0	0	0	1	0	1	0	0	1	0	1	1	1	1	1	1	1	0	0	1	0	1	0	1	0	1
seedMaxEhybrid-7.seedMinPu0.1.seedNoGU	11	0	0	0	0	0	0	0	0	0	0	0	1	0	0	0	0	0	0	0	0	0	0	0	0	0	1	0	0	0	0	1	0	1	0	0	0	0	0	0	0	1	0	0	0	0	0	0	0	0	0	0	0	0	0	0	0	0	0	0	0	0	0	0	0	0	0	0	0	1	0	0	0	0	0	0	0	0	1	0	0	0	0	0	0	0	0	0	1	0	0	0	1	0	0	0	1	0	0	0	1	0	0
seedMaxEhybrid-7.seedMinPu0.001.seedNoGU	43	0	1	0	1	1	1	0	1	0	1	1	0	0	1	0	0	0	0	1	0	0	0	0	0	0	0	0	0	0	0	0	0	1	0	0	0	1	1	1	0	1	1	0	0	1	0	0	0	0	1	1	1	0	0	1	0	1	0	1	0	1	0	0	0	0	0	0	0	1	0	0	1	1	1	1	1	0	0	1	1	0	1	0	1	0	0	0	1	1	1	1	1	1	0	1	1	0	1	0	1	0	0
seedMaxEhybrid-7.seedMinPu0.0001.seedNoGU	43	0	0	0	1	1	1	0	1	0	1	1	0	0	1	1	0	0	0	1	0	0	0	0	0	0	0	1	0	0	0	0	0	1	0	0	0	1	1	1	0	1	1	0	0	1	0	0	0	0	1	1	1	0	0	1	0	1	0	1	0	1	0	0	0	0	0	0	0	0	0	0	1	1	0	1	1	0	0	1	1	0	1	0	0	1	0	1	1	1	1	1	1	1	0	1	1	0	1	0	1	0	0
seedMaxEhybrid-7.seedMinPu0.01.seedNoGU	40	0	1	0	1	1	1	1	1	0	1	1	0	0	1	0	0	0	0	1	0	0	0	0	0	0	0	0	0	0	0	0	1	1	0	0	0	1	1	1	0	1	1	1	0	1	0	0	0	0	1	1	0	1	0	0	0	0	0	0	0	1	0	0	0	0	0	0	0	1	0	0	1	1	1	1	1	0	0	0	1	0	1	0	0	0	0	0	1	1	1	0	1	1	0	1	1	0	1	0	1	0	0
seedMaxEhybrid-7.seedMinPu0.2	7	0	0	0	0	0	0	0	0	0	0	0	0	0	0	0	0	0	0	0	0	0	0	0	0	0	1	0	0	0	0	1	1	0	0	0	0	0	0	0	0	1	0	0	0	0	0	0	0	0	0	0	0	0	0	0	0	0	0	0	0	0	0	0	0	0	0	0	0	0	0	0	0	0	0	0	0	0	0	0	0	0	0	0	0	0	0	0	0	0	0	0	1	0	0	0	1	0	0	0	1	0	0
seedMaxEhybrid-7.seedMinPu0.2.seedNoGU	6	0	0	0	0	0	0	0	0	0	0	0	0	0	0	0	0	0	0	0	0	0	0	0	0	0	1	0	0	0	0	1	0	0	0	0	0	0	0	0	0	1	0	0	0	0	0	0	0	0	0	0	0	0	0	0	0	0	0	0	0	0	0	0	0	0	0	0	0	0	0	0	0	0	0	0	0	0	0	0	0	0	0	0	0	0	0	0	0	0	0	0	1	0	0	0	1	0	0	0	1	0	0
seedMaxEhybrid-7.seedMinPu0.5	0	0	0	0	0	0	0	0	0	0	0	0	0	0	0	0	0	0	0	0	0	0	0	0	0	0	0	0	0	0	0	0	0	0	0	0	0	0	0	0	0	0	0	0	0	0	0	0	0	0	0	0	0	0	0	0	0	0	0	0	0	0	0	0	0	0	0	0	0	0	0	0	0	0	0	0	0	0	0	0	0	0	0	0	0	0	0	0	0	0	0	0	0	0	0	0	0	0	0	0	0	0	0
seedMaxEhybrid-7.seedMinPu0.5.seedNoGU	0	0	0	0	0	0	0	0	0	0	0	0	0	0	0	0	0	0	0	0	0	0	0	0	0	0	0	0	0	0	0	0	0	0	0	0	0	0	0	0	0	0	0	0	0	0	0	0	0	0	0	0	0	0	0	0	0	0	0	0	0	0	0	0	0	0	0	0	0	0	0	0	0	0	0	0	0	0	0	0	0	0	0	0	0	0	0	0	0	0	0	0	0	0	0	0	0	0	0	0	0	0	0
seedMaxEhybrid-7.seedMinPu0.seedNoGU	40	0	0	0	1	1	1	0	1	0	1	1	0	0	1	1	0	0	0	0	0	0	0	0	0	0	0	1	0	0	0	0	0	1	0	0	0	1	1	1	0	1	0	0	0	1	0	0	0	0	1	1	1	0	0	1	0	1	0	1	0	1	0	0	0	0	0	0	0	0	0	0	1	1	0	1	1	0	0	0	1	0	1	0	0	1	0	1	1	1	1	1	1	1	0	1	1	0	1	0	1	0	0
seedMaxEhybrid-7.seedNoGU	40	0	0	0	1	1	1	0	1	0	1	1	0	0	1	1	0	0	0	0	0	0	0	0	0	0	0	1	0	0	0	0	0	1	0	0	0	1	1	1	0	1	0	0	0	1	0	0	0	0	1	1	1	0	0	1	0	1	0	1	0	1	0	0	0	0	0	0	0	0	0	0	1	1	0	1	1	0	0	0	1	0	1	0	0	1	0	1	1	1	1	1	1	1	0	1	1	0	1	0	1	0	0
seedMaxEhybrid-8	30	0	0	0	1	1	1	0	1	0	1	0	0	0	1	0	0	0	0	0	0	0	0	1	0	0	0	1	0	0	0	0	0	1	0	0	0	1	1	1	0	0	0	0	0	1	0	0	0	0	1	0	1	0	0	0	0	0	0	0	0	1	1	0	0	0	0	0	0	0	0	0	0	0	0	1	1	0	0	0	1	0	1	0	0	1	0	1	1	1	1	0	1	1	0	0	0	0	1	0	1	0	0
seedMaxEhybrid-8.seedMinPu0	30	0	0	0	1	1	1	0	1	0	1	0	0	0	1	0	0	0	0	0	0	0	0	1	0	0	0	1	0	0	0	0	0	1	0	0	0	1	1	1	0	0	0	0	0	1	0	0	0	0	1	0	1	0	0	0	0	0	0	0	0	1	1	0	0	0	0	0	0	0	0	0	0	0	0	1	1	0	0	0	1	0	1	0	0	1	0	1	1	1	1	0	1	1	0	0	0	0	1	0	1	0	0
seedMaxEhybrid-8.seedMinPu0.0001	31	0	0	0	1	1	1	0	1	0	1	0	0	0	1	0	0	0	0	0	0	0	0	1	0	0	0	1	0	0	0	0	0	1	0	0	0	1	1	1	0	0	0	0	0	1	0	0	0	0	1	0	1	0	0	0	0	0	0	0	0	1	1	0	0	0	0	0	0	0	0	0	0	0	0	1	1	0	0	0	1	0	1	0	0	1	0	1	1	1	1	0	1	1	0	1	0	0	1	0	1	0	0
seedMaxEhybrid-8.seedMinPu0.01	35	0	1	0	1	1	1	1	0	0	1	0	0	0	1	0	0	0	0	1	0	0	0	1	0	0	0	0	0	0	0	0	0	1	0	0	0	1	1	1	0	0	0	0	0	1	0	0	0	0	1	1	0	1	0	0	0	0	0	0	0	1	1	0	0	0	0	0	0	1	0	0	1	1	0	1	1	0	0	0	1	0	1	0	0	0	0	1	1	1	1	0	1	1	0	1	0	0	1	0	1	0	0
seedMaxEhybrid-8.seedMinPu0.1	7	0	0	0	0	0	0	0	0	0	0	0	1	0	0	0	0	0	0	0	0	0	0	0	0	0	0	0	0	0	0	1	0	1	0	0	0	0	0	0	0	0	0	0	0	0	0	0	0	0	0	0	0	0	0	0	0	0	0	0	0	0	0	0	0	0	0	0	0	1	0	0	0	0	0	0	0	0	0	0	0	0	0	0	0	0	0	0	1	0	0	0	1	0	0	0	0	0	0	0	1	0	0
seedMaxEhybrid-8.seedMinPu0.001	34	0	0	0	1	1	1	0	1	0	1	0	0	0	1	0	0	0	0	0	0	0	0	1	0	0	0	0	0	0	0	0	0	1	0	0	0	1	1	1	0	0	0	0	0	1	0	0	0	0	1	0	1	0	0	1	0	0	0	1	0	1	1	0	0	0	1	0	0	0	0	0	1	0	0	1	1	0	0	0	1	0	1	0	0	1	0	1	1	1	1	0	1	1	0	1	0	0	1	0	1	0	0
seedMaxEhybrid-8.seedMinPu0.1.seedNoGU	7	0	0	0	0	0	0	0	0	0	0	0	1	0	0	0	0	0	0	0	0	0	0	0	0	0	0	0	0	0	0	1	0	1	0	0	0	0	0	0	0	0	0	0	0	0	0	0	0	0	0	0	0	0	0	0	0	0	0	0	0	0	0	0	0	0	0	0	0	1	0	0	0	0	0	0	0	0	0	0	0	0	0	0	0	0	0	0	1	0	0	0	1	0	0	0	0	0	0	0	1	0	0
seedMaxEhybrid-8.seedMinPu0.01.seedNoGU	35	0	1	0	1	1	1	1	0	0	1	0	1	0	1	0	0	0	0	1	0	0	0	0	0	1	0	0	0	0	0	1	0	1	0	0	0	1	1	1	0	0	0	0	0	0	0	0	0	0	1	1	0	1	0	1	0	0	0	0	0	1	0	0	0	0	0	0	0	1	0	0	1	1	0	1	1	0	0	0	1	0	1	0	0	0	0	0	1	1	1	0	1	1	0	1	0	0	1	0	1	0	0
seedMaxEhybrid-8.seedMinPu0.001.seedNoGU	38	0	1	0	1	1	1	0	1	0	1	0	0	0	1	0	0	0	0	1	0	0	0	0	0	0	0	0	0	0	0	0	1	1	0	0	0	1	1	1	0	0	0	0	0	1	0	0	0	0	1	1	1	1	0	1	0	0	0	1	0	1	0	0	0	0	0	0	0	1	0	0	1	1	0	1	1	0	0	1	1	0	1	0	1	0	0	0	1	1	1	0	1	1	0	1	0	0	1	0	1	0	0
seedMaxEhybrid-8.seedMinPu0.0001.seedNoGU	38	0	0	0	1	1	1	0	1	0	1	0	0	0	1	0	0	0	0	1	0	0	0	0	0	0	0	1	0	0	0	0	1	1	0	0	0	1	1	1	0	0	0	0	0	1	0	0	0	0	1	1	1	0	0	1	0	0	0	1	0	1	0	0	0	0	0	0	0	1	0	0	1	1	0	1	1	0	0	1	1	0	1	0	1	1	0	0	1	1	1	0	1	1	0	1	0	0	1	0	1	0	0
seedMaxEhybrid-8.seedMinPu0.2	3	0	0	0	0	0	0	0	0	0	0	0	0	0	0	0	0	0	0	0	0	0	0	0	0	0	0	0	0	0	0	1	0	0	0	0	0	0	0	0	0	0	0	0	0	0	0	0	0	0	0	0	0	0	0	0	0	0	0	0	0	0	0	0	0	0	0	0	0	0	0	0	0	0	0	0	0	0	0	0	0	0	0	0	0	0	0	0	0	0	0	0	1	0	0	0	0	0	0	0	1	0	0
seedMaxEhybrid-8.seedMinPu0.2.seedNoGU	3	0	0	0	0	0	0	0	0	0	0	0	0	0	0	0	0	0	0	0	0	0	0	0	0	0	0	0	0	0	0	1	0	0	0	0	0	0	0	0	0	0	0	0	0	0	0	0	0	0	0	0	0	0	0	0	0	0	0	0	0	0	0	0	0	0	0	0	0	0	0	0	0	0	0	0	0	0	0	0	0	0	0	0	0	0	0	0	0	0	0	0	1	0	0	0	0	0	0	0	1	0	0
seedMaxEhybrid-8.seedMinPu0.5	0	0	0	0	0	0	0	0	0	0	0	0	0	0	0	0	0	0	0	0	0	0	0	0	0	0	0	0	0	0	0	0	0	0	0	0	0	0	0	0	0	0	0	0	0	0	0	0	0	0	0	0	0	0	0	0	0	0	0	0	0	0	0	0	0	0	0	0	0	0	0	0	0	0	0	0	0	0	0	0	0	0	0	0	0	0	0	0	0	0	0	0	0	0	0	0	0	0	0	0	0	0	0
seedMaxEhybrid-8.seedMinPu0.5.seedNoGU	0	0	0	0	0	0	0	0	0	0	0	0	0	0	0	0	0	0	0	0	0	0	0	0	0	0	0	0	0	0	0	0	0	0	0	0	0	0	0	0	0	0	0	0	0	0	0	0	0	0	0	0	0	0	0	0	0	0	0	0	0	0	0	0	0	0	0	0	0	0	0	0	0	0	0	0	0	0	0	0	0	0	0	0	0	0	0	0	0	0	0	0	0	0	0	0	0	0	0	0	0	0	0
seedMaxEhybrid-8.seedMinPu0.seedNoGU	37	0	0	0	1	1	1	0	1	0	1	0	0	0	1	0	0	0	0	1	0	0	0	0	0	0	0	1	0	0	0	0	1	1	0	0	0	1	1	1	0	0	0	0	0	1	0	0	0	0	1	1	1	0	0	1	0	0	0	1	0	1	0	0	0	0	0	0	0	0	0	0	1	1	0	1	1	0	0	1	1	0	1	0	1	1	0	0	1	1	1	0	1	1	0	1	0	0	1	0	1	0	0
seedMaxEhybrid-8.seedNoGU	37	0	0	0	1	1	1	0	1	0	1	0	0	0	1	0	0	0	0	1	0	0	0	0	0	0	0	1	0	0	0	0	1	1	0	0	0	1	1	1	0	0	0	0	0	1	0	0	0	0	1	1	1	0	0	1	0	0	0	1	0	1	0	0	0	0	0	0	0	0	0	0	1	1	0	1	1	0	0	1	1	0	1	0	1	1	0	0	1	1	1	0	1	1	0	1	0	0	1	0	1	0	0
seedMaxEhybrid-9	29	0	0	0	1	1	1	0	1	0	1	0	0	0	0	0	0	0	0	1	0	0	0	1	0	0	0	0	0	0	0	0	0	1	0	0	0	1	1	1	0	0	0	0	0	1	0	0	0	0	1	0	1	0	0	1	0	0	0	1	0	1	1	0	0	0	0	0	0	0	0	0	1	0	0	0	1	0	0	0	0	0	1	0	1	1	0	1	0	0	1	0	1	1	0	1	0	0	1	0	0	0	0
seedMaxEhybrid-9.seedMinPu0	29	0	0	0	1	1	1	0	1	0	1	0	0	0	0	0	0	0	0	1	0	0	0	1	0	0	0	0	0	0	0	0	0	1	0	0	0	1	1	1	0	0	0	0	0	1	0	0	0	0	1	0	1	0	0	1	0	0	0	1	0	1	1	0	0	0	0	0	0	0	0	0	1	0	0	0	1	0	0	0	0	0	1	0	1	1	0	1	0	0	1	0	1	1	0	1	0	0	1	0	0	0	0
seedMaxEhybrid-9.seedMinPu0.01	26	0	1	0	1	1	1	1	0	0	1	0	0	0	0	0	0	0	0	1	0	0	0	1	0	1	0	0	0	0	0	0	0	1	0	0	0	1	1	0	0	0	1	0	0	0	0	0	0	0	1	1	0	0	0	0	0	0	0	0	0	1	0	0	0	0	0	0	0	1	0	0	1	1	0	0	0	0	0	0	0	0	1	0	0	0	0	1	0	0	1	0	1	1	0	1	0	0	1	0	0	0	0
seedMaxEhybrid-9.seedMinPu0.1	3	0	0	0	0	0	0	0	0	0	0	0	1	0	0	0	0	0	0	0	0	0	0	0	0	0	0	0	0	0	0	1	0	0	0	0	0	0	0	0	0	0	0	0	0	0	0	0	0	0	0	0	0	0	0	0	0	0	0	0	0	0	0	0	0	0	0	0	0	1	0	0	0	0	0	0	0	0	0	0	0	0	0	0	0	0	0	0	0	0	0	0	0	0	0	0	0	0	0	0	0	0	0
seedMaxEhybrid-9.seedMinPu0.0001	30	0	0	0	1	1	1	0	1	0	1	0	0	0	0	0	0	0	0	1	0	0	0	1	0	0	0	0	0	0	0	0	0	1	0	0	0	1	1	1	0	0	0	0	0	1	0	0	0	0	1	0	1	0	0	1	0	0	0	1	0	1	1	0	0	0	0	0	0	0	0	0	1	1	0	0	1	0	0	0	0	0	1	0	1	1	0	1	0	0	1	0	1	1	0	1	0	0	1	0	0	0	0
seedMaxEhybrid-9.seedMinPu0.001	32	0	0	0	1	1	1	0	1	0	1	0	0	0	0	0	0	0	0	1	0	0	0	1	0	0	0	0	0	0	0	0	0	1	0	0	0	1	1	0	0	0	1	0	0	1	0	0	0	0	1	1	1	0	0	1	0	0	0	1	0	1	1	0	0	0	0	0	0	1	0	0	1	1	0	0	0	0	0	1	0	0	1	0	1	1	0	1	0	0	1	0	1	1	0	1	0	0	1	0	0	0	0
seedMaxEhybrid-9.seedMinPu0.001.seedNoGU	31	0	1	0	1	1	1	1	1	0	1	0	0	0	0	0	0	0	0	1	0	0	0	0	0	1	0	1	0	0	0	1	0	1	0	0	0	1	1	0	0	0	0	0	0	1	0	0	0	0	1	1	1	0	0	1	0	0	0	1	0	1	0	0	0	0	0	0	0	1	0	0	1	1	0	0	0	0	0	1	0	0	1	0	1	0	0	0	0	0	1	0	0	1	0	1	0	0	1	0	0	0	0
seedMaxEhybrid-9.seedMinPu0.1.seedNoGU	3	0	0	0	0	0	0	0	0	0	0	0	1	0	0	0	0	0	0	0	0	0	0	0	0	0	0	0	0	0	0	1	0	0	0	0	0	0	0	0	0	0	0	0	0	0	0	0	0	0	0	0	0	0	0	0	0	0	0	0	0	0	0	0	0	0	0	0	0	1	0	0	0	0	0	0	0	0	0	0	0	0	0	0	0	0	0	0	0	0	0	0	0	0	0	0	0	0	0	0	0	0	0
seedMaxEhybrid-9.seedMinPu0.01.seedNoGU	25	0	1	0	1	1	1	1	0	0	1	0	1	0	0	0	0	0	0	1	0	0	0	0	0	1	0	1	0	0	0	1	0	1	0	0	0	1	1	0	0	0	0	0	0	0	0	0	0	0	1	1	0	0	0	0	0	0	0	0	0	1	0	0	0	0	0	0	0	1	0	0	1	1	0	0	0	0	0	0	0	0	1	0	0	0	0	0	0	0	1	0	0	1	0	1	0	0	1	0	0	0	0
seedMaxEhybrid-9.seedMinPu0.0001.seedNoGU	31	0	0	0	1	1	1	0	1	0	1	0	0	0	0	0	0	0	0	1	0	0	0	0	0	1	0	1	0	0	0	0	0	1	0	0	0	1	1	1	0	0	0	0	0	1	0	0	0	0	1	1	1	0	0	1	0	0	0	1	0	1	0	0	0	0	0	0	0	1	0	0	1	1	0	0	1	0	0	1	0	0	1	0	1	1	0	0	0	0	1	0	0	1	0	1	0	0	1	0	0	0	0
seedMaxEhybrid-9.seedMinPu0.2	1	0	0	0	0	0	0	0	0	0	0	0	0	0	0	0	0	0	0	0	0	0	0	0	0	0	0	0	0	0	0	1	0	0	0	0	0	0	0	0	0	0	0	0	0	0	0	0	0	0	0	0	0	0	0	0	0	0	0	0	0	0	0	0	0	0	0	0	0	0	0	0	0	0	0	0	0	0	0	0	0	0	0	0	0	0	0	0	0	0	0	0	0	0	0	0	0	0	0	0	0	0	0
seedMaxEhybrid-9.seedMinPu0.2.seedNoGU	1	0	0	0	0	0	0	0	0	0	0	0	0	0	0	0	0	0	0	0	0	0	0	0	0	0	0	0	0	0	0	1	0	0	0	0	0	0	0	0	0	0	0	0	0	0	0	0	0	0	0	0	0	0	0	0	0	0	0	0	0	0	0	0	0	0	0	0	0	0	0	0	0	0	0	0	0	0	0	0	0	0	0	0	0	0	0	0	0	0	0	0	0	0	0	0	0	0	0	0	0	0	0
seedMaxEhybrid-9.seedMinPu0.5	0	0	0	0	0	0	0	0	0	0	0	0	0	0	0	0	0	0	0	0	0	0	0	0	0	0	0	0	0	0	0	0	0	0	0	0	0	0	0	0	0	0	0	0	0	0	0	0	0	0	0	0	0	0	0	0	0	0	0	0	0	0	0	0	0	0	0	0	0	0	0	0	0	0	0	0	0	0	0	0	0	0	0	0	0	0	0	0	0	0	0	0	0	0	0	0	0	0	0	0	0	0	0
seedMaxEhybrid-9.seedMinPu0.5.seedNoGU	0	0	0	0	0	0	0	0	0	0	0	0	0	0	0	0	0	0	0	0	0	0	0	0	0	0	0	0	0	0	0	0	0	0	0	0	0	0	0	0	0	0	0	0	0	0	0	0	0	0	0	0	0	0	0	0	0	0	0	0	0	0	0	0	0	0	0	0	0	0	0	0	0	0	0	0	0	0	0	0	0	0	0	0	0	0	0	0	0	0	0	0	0	0	0	0	0	0	0	0	0	0	0
seedMaxEhybrid-9.seedMinPu0.seedNoGU	29	0	0	0	1	1	1	0	1	0	1	0	0	0	0	0	0	0	0	1	0	0	0	0	0	0	0	0	0	0	0	0	0	1	0	0	0	1	1	1	0	0	0	0	0	1	0	0	0	0	1	1	1	0	0	1	0	0	0	1	0	1	0	0	0	0	0	0	0	1	0	0	1	1	0	0	1	0	0	1	0	0	1	0	1	1	0	0	0	0	1	0	0	1	0	1	0	0	1	0	0	0	0
seedMaxEhybrid-9.seedNoGU	29	0	0	0	1	1	1	0	1	0	1	0	0	0	0	0	0	0	0	1	0	0	0	0	0	0	0	0	0	0	0	0	0	1	0	0	0	1	1	1	0	0	0	0	0	1	0	0	0	0	1	1	1	0	0	1	0	0	0	1	0	1	0	0	0	0	0	0	0	1	0	0	1	1	0	0	1	0	0	1	0	0	1	0	1	1	0	0	0	0	1	0	0	1	0	1	0	0	1	0	0	0	0
seedMaxEhybrid-10	27	0	0	0	0	0	1	0	1	0	0	0	0	1	0	0	0	0	0	1	0	0	0	0	0	1	0	0	0	0	0	0	1	1	0	0	0	1	1	0	0	0	0	0	0	0	0	0	0	0	1	1	1	0	0	0	0	0	0	1	0	1	1	0	0	0	0	0	0	1	0	0	1	1	0	0	0	0	0	1	0	0	0	0	1	1	0	1	0	0	1	0	1	1	0	1	0	0	1	0	0	0	0
seedMaxEhybrid-10.seedMinPu0	27	0	0	0	0	0	1	0	1	0	0	0	0	1	0	0	0	0	0	1	0	0	0	0	0	1	0	0	0	0	0	0	1	1	0	0	0	1	1	0	0	0	0	0	0	0	0	0	0	0	1	1	1	0	0	0	0	0	0	1	0	1	1	0	0	0	0	0	0	1	0	0	1	1	0	0	0	0	0	1	0	0	0	0	1	1	0	1	0	0	1	0	1	1	0	1	0	0	1	0	0	0	0

Echericha coli NC_000913

	sum	ArcZ-b1892	ArcZ-b2741	ArcZ-b3546	ChiX-b0619	ChiX-b0681	ChiX-b1737	CyaR-b0723	CyaR-b0814	CyaR-b1740	CyaR-b1824	CyaR-b2416	CyaR-b2666	CyaR-b2687	DsrA-b1237	DsrA-b2741	DsrA-b3251	FnrS-b0723	FnrS-b0755	FnrS-b0887	FnrS-b1107	FnrS-b1479	FnrS-b1531	FnrS-b1656	FnrS-b1841	FnrS-b2153	FnrS-b2303
Target found among top-100 predictions for the sRNA for any tested parameterization	86	0	1	0	1	1	1	1	1	0	1	1	1	1	1	1	0	1	0	1	1	1	1	1	1	1	1
seedMaxEhybrid-10.seedMinPu0.1	2	0	0	0	0	0	0	0	0	0	0	0	0	0	0	0	0	0	0	0	0	0	0	0	0	0	0
seedMaxEhybrid-10.seedMinPu0.01	19	0	0	0	0	0	0	0	0	0	0	0	0	0	0	0	0	0	0	1	0	0	0	0	0	1	0
seedMaxEhybrid-10.seedMinPu0.0001	27	0	0	0	0	0	0	0	1	0	0	0	0	1	0	0	0	0	0	1	0	0	0	0	0	1	0
seedMaxEhybrid-10.seedMinPu0.001	28	0	0	0	0	0	0	0	1	0	0	0	0	1	0	0	0	0	0	1	0	0	0	0	0	1	0
seedMaxEhybrid-10.seedMinPu0.001.seedNoGU	22	0	0	0	0	0	0	0	1	0	0	0	0	1	0	0	0	0	0	1	0	0	0	0	0	1	0
seedMaxEhybrid-10.seedMinPu0.0001.seedNoGU	23	0	0	0	0	0	0	0	1	0	0	0	0	1	0	0	0	0	0	1	0	0	0	0	0	1	0
seedMaxEhybrid-10.seedMinPu0.01.seedNoGU	16	0	0	0	0	0	0	0	0	0	0	0	0	0	0	0	0	0	0	1	0	0	0	0	0	1	0
seedMaxEhybrid-10.seedMinPu0.1.seedNoGU	2	0	0	0	0	0	0	0	0	0	0	0	0	0	0	0	0	0	0	0	0	0	0	0	0	0	0
seedMaxEhybrid-10.seedMinPu0.2	1	0	0	0	0	0	0	0	0	0	0	0	0	0	0	0	0	0	0	0	0	0	0	0	0	0	0
seedMaxEhybrid-10.seedMinPu0.2.seedNoGU	1	0	0	0	0	0	0	0	0	0	0	0	0	0	0	0	0	0	0	0	0	0	0	0	0	0	0
seedMaxEhybrid-10.seedMinPu0.5	0	0	0	0	0	0	0	0	0	0	0	0	0	0	0	0	0	0	0	0	0	0	0	0	0	0	0
seedMaxEhybrid-10.seedMinPu0.5.seedNoGU	0	0	0	0	0	0	0	0	0	0	0	0	0	0	0	0	0	0	0	0	0	0	0	0	0	0	0
seedMaxEhybrid-10.seedMinPu0.seedNoGU	24	0	0	0	0	0	1	0	1	0	0	0	0	1	0	0	0	0	0	1	0	0	0	0	0	1	0
seedMaxEhybrid-10.seedNoGU	24	0	0	0	0	0	1	0	1	0	0	0	0	1	0	0	0	0	0	1	0	0	0	0	0	1	0

	FnrS-b2531	FnrS-b3829	FnrS-b3908	GcvB-b1040	GcvB-b1130	GcvB-b3089	GcvB-b4208	MicA-b0411	MicA-b0814	MicA-b0957	MicA-b1130	MicC-b2215	MicF-b0241	MicF-b0889	MicF-b0929	MicF-b3912	OxyS-b1892	OxyS-b2731	RprA-b1040	RprA-b1341	RprA-b2741	RybB-b0081	RybB-b0721	RybB-b0805	RybB-b1256	RybB-b2215
Target found among top-100 predictions for the sRNA for any tested parameterization	1	1	0	1	1	1	1	1	1	1	1	1	1	1	1	1	1	0	1	1	1	1	1	1	1	1
seedMaxEhybrid-10.seedMinPu0.1	0	0	0	0	1	0	0	0	0	0	0	0	0	0	0	0	0	0	0	0	0	0	0	0	0	0
seedMaxEhybrid-10.seedMinPu0.01	0	0	0	0	1	0	1	0	0	0	1	0	0	0	0	1	0	0	0	0	0	0	0	1	1	0
seedMaxEhybrid-10.seedMinPu0.0001	0	0	0	0	1	1	1	0	0	0	1	1	0	0	0	0	0	0	0	0	0	0	0	1	1	1
seedMaxEhybrid-10.seedMinPu0.001	0	0	0	0	1	1	1	0	0	0	1	1	0	0	0	1	0	0	0	0	0	0	0	1	1	1
seedMaxEhybrid-10.seedMinPu0.001.seedNoGU	0	0	0	0	1	0	1	0	0	0	1	1	0	0	0	0	0	0	0	0	0	0	0	1	1	1
seedMaxEhybrid-10.seedMinPu0.0001.seedNoGU	0	0	0	0	1	0	1	0	0	0	1	1	0	0	0	0	0	0	0	0	0	0	0	1	1	1
seedMaxEhybrid-10.seedMinPu0.01.seedNoGU	0	0	0	0	1	0	1	0	0	0	1	0	0	0	0	0	0	0	0	0	0	0	0	1	1	0
seedMaxEhybrid-10.seedMinPu0.1.seedNoGU	0	0	0	0	1	0	0	0	0	0	0	0	0	0	0	0	0	0	0	0	0	0	0	0	0	0
seedMaxEhybrid-10.seedMinPu0.2	0	0	0	0	1	0	0	0	0	0	0	0	0	0	0	0	0	0	0	0	0	0	0	0	0	0
seedMaxEhybrid-10.seedMinPu0.2.seedNoGU	0	0	0	0	1	0	0	0	0	0	0	0	0	0	0	0	0	0	0	0	0	0	0	0	0	0
seedMaxEhybrid-10.seedMinPu0.5	0	0	0	0	0	0	0	0	0	0	0	0	0	0	0	0	0	0	0	0	0	0	0	0	0	0
seedMaxEhybrid-10.seedMinPu0.5.seedNoGU	0	0	0	0	0	0	0	0	0	0	0	0	0	0	0	0	0	0	0	0	0	0	0	0	0	0
seedMaxEhybrid-10.seedMinPu0.seedNoGU	0	0	0	0	1	0	1	0	0	0	1	1	0	0	0	0	0	0	0	0	0	0	0	1	1	1
seedMaxEhybrid-10.seedNoGU	0	0	0	0	1	0	1	0	0	0	1	1	0	0	0	0	0	0	0	0	0	0	0	1	1	1

	RybB-b2594	RyhB-b0118	RyhB-b0156	RyhB-b0288	RyhB-b0592	RyhB-b0683	RyhB-b0721	RyhB-b0723	RyhB-b0894	RyhB-b1107	RyhB-b1200	RyhB-b1452	RyhB-b1531	RyhB-b1588	RyhB-b1612	RyhB-b1656	RyhB-b1778	RyhB-b1981	RyhB-b2069	RyhB-b2155	RyhB-b2206	RyhB-b2530	RyhB-b3365	RyhB-b3607	RyhB-b3942	RyhB-b4070
Target found among top-100 predictions for the sRNA for any tested parameterization	1	0	1	1	1	0	1	0	1	1	1	0	0	1	1	1	1	1	0	1	1	1	1	1	0	1
seedMaxEhybrid-10.seedMinPu0.1	0	0	0	0	0	0	0	0	0	0	0	0	0	0	0	0	1	0	0	0	0	0	0	0	0	0
seedMaxEhybrid-10.seedMinPu0.01	0	0	0	0	0	0	0	0	1	0	0	0	0	0	0	0	1	0	0	1	1	0	0	1	0	0
seedMaxEhybrid-10.seedMinPu0.0001	0	0	0	0	0	0	1	0	1	1	0	0	0	0	0	0	1	0	0	1	1	0	0	0	0	0
seedMaxEhybrid-10.seedMinPu0.001	0	0	0	0	0	0	1	0	1	1	0	0	0	0	0	0	1	0	0	1	1	0	0	0	0	0
seedMaxEhybrid-10.seedMinPu0.001.seedNoGU	0	0	0	0	0	0	1	0	1	0	0	0	0	0	0	0	1	0	0	1	1	0	0	0	0	0
seedMaxEhybrid-10.seedMinPu0.0001.seedNoGU	0	0	0	0	0	0	1	0	1	0	0	0	0	0	0	0	1	0	0	1	1	0	0	0	0	0
seedMaxEhybrid-10.seedMinPu0.01.seedNoGU	0	0	0	0	0	0	0	0	1	0	0	0	0	0	0	0	1	0	0	1	1	0	0	1	0	0
seedMaxEhybrid-10.seedMinPu0.1.seedNoGU	0	0	0	0	0	0	0	0	0	0	0	0	0	0	0	0	1	0	0	0	0	0	0	0	0	0
seedMaxEhybrid-10.seedMinPu0.2	0	0	0	0	0	0	0	0	0	0	0	0	0	0	0	0	0	0	0	0	0	0	0	0	0	0
seedMaxEhybrid-10.seedMinPu0.2.seedNoGU	0	0	0	0	0	0	0	0	0	0	0	0	0	0	0	0	0	0	0	0	0	0	0	0	0	0
seedMaxEhybrid-10.seedMinPu0.5	0	0	0	0	0	0	0	0	0	0	0	0	0	0	0	0	0	0	0	0	0	0	0	0	0	0
seedMaxEhybrid-10.seedMinPu0.5.seedNoGU	0	0	0	0	0	0	0	0	0	0	0	0	0	0	0	0	0	0	0	0	0	0	0	0	0	0
seedMaxEhybrid-10.seedMinPu0.seedNoGU	0	0	0	0	0	0	1	0	1	0	0	0	0	0	0	0	1	0	0	1	1	0	0	0	0	0
seedMaxEhybrid-10.seedNoGU	0	0	0	0	0	0	1	0	1	0	0	0	0	0	0	0	1	0	0	1	1	0	0	0	0	0

	RyhB-b4122	SgrS-b1101	SgrS-b1817	SgrS-b2416	Spot42-b0039	Spot42-b0720	Spot42-b0721	Spot42-b0728	Spot42-b0757	Spot42-b1136	Spot42-b1302	Spot42-b1398	Spot42-b1761	Spot42-b1901	Spot42-b2221	Spot42-b2702	Spot42-b2715	Spot42-b2801	Spot42-b2802	Spot42-b3224	Spot42-b3566	Spot42-b3927	Spot42-b3962	Spot42-b4311
Target found among top-100 predictions for the sRNA for any tested parameterization	1	1	1	1	0	1	1	1	1	1	1	1	1	1	1	1	1	1	0	1	1	1	1	1
seedMaxEhybrid-10.seedMinPu0.1	0	0	0	0	0	0	0	0	0	0	0	0	0	0	0	0	0	0	0	0	0	0	0	0
seedMaxEhybrid-10.seedMinPu0.01	0	0	0	0	0	0	0	0	1	0	0	1	0	1	1	0	1	0	0	1	0	0	0	0
seedMaxEhybrid-10.seedMinPu0.0001	1	0	0	0	0	1	1	0	1	0	0	1	0	1	1	0	1	0	0	1	0	0	0	0
seedMaxEhybrid-10.seedMinPu0.001	1	0	0	0	0	1	1	0	1	0	0	1	0	1	1	0	1	0	0	1	0	0	0	0
seedMaxEhybrid-10.seedMinPu0.001.seedNoGU	1	0	0	0	0	1	0	0	0	0	0	1	0	0	1	0	1	0	0	1	0	0	0	0
seedMaxEhybrid-10.seedMinPu0.0001.seedNoGU	1	0	0	0	0	1	1	0	0	0	0	1	0	0	1	0	1	0	0	1	0	0	0	0
seedMaxEhybrid-10.seedMinPu0.01.seedNoGU	0	0	0	0	0	0	0	0	0	0	0	1	0	0	1	0	1	0	0	1	0	0	0	0
seedMaxEhybrid-10.seedMinPu0.1.seedNoGU	0	0	0	0	0	0	0	0	0	0	0	0	0	0	0	0	0	0	0	0	0	0	0	0
seedMaxEhybrid-10.seedMinPu0.2	0	0	0	0	0	0	0	0	0	0	0	0	0	0	0	0	0	0	0	0	0	0	0	0
seedMaxEhybrid-10.seedMinPu0.2.seedNoGU	0	0	0	0	0	0	0	0	0	0	0	0	0	0	0	0	0	0	0	0	0	0	0	0
seedMaxEhybrid-10.seedMinPu0.5	0	0	0	0	0	0	0	0	0	0	0	0	0	0	0	0	0	0	0	0	0	0	0	0
seedMaxEhybrid-10.seedMinPu0.5.seedNoGU	0	0	0	0	0	0	0	0	0	0	0	0	0	0	0	0	0	0	0	0	0	0	0	0
seedMaxEhybrid-10.seedMinPu0.seedNoGU	1	0	0	0	0	1	1	0	0	0	0	1	0	0	1	0	1	0	0	1	0	0	0	0
seedMaxEhybrid-10.seedNoGU	1	0	0	0	0	1	1	0	0	0	0	1	0	0	1	0	1	0	0	1	0	0	0	0

***Salmonella typhimurium* NC_003197**	sum	ArcZ-STM1682	ArcZ-STM2970	ArcZ-STM3216	ChiX-STM0687	ChiX-STM1313	CyaR-STM0833	GcvB-STM0002	GcvB-STM0245	GcvB-STM0399	GcvB-STM0602	GcvB-STM0665	GcvB-STM0959	GcvB-STM1299	GcvB-STM1452	GcvB-STM1746	GcvB-STM2355	GcvB-STM2526	GcvB-STM3062	GcvB-STM3064	GcvB-STM3225	GcvB-STM3564	GcvB-STM3567	GcvB-STM3630	GcvB-STM3903	GcvB-STM3909	GcvB-STM3930	GcvB-STM4351	GcvB-STM4398	MicA-STM4231	MicC-STM1572	MicF-STM0366	MicF-STM0959	MicF-STM1328	RybB-STM0413	RybB-STM0687	RybB-STM0999	RybB-STM1070	RybB-STM1473	RybB-STM1530	RybB-STM1572	RybB-STM1732	RybB-STM1995	RybB-STM2267	RybB-STM2391	SgrS-STM2945	SgrS-STM3962	Spot42-STM2190
Target found among top-100 predictions for the sRNA for any tested parameterization	40	1	0	0	1	1	1	1	1	1	1	1	1	1	0	0	1	1	1	1	1	1	1	1	1	1	1	1	1	1	1	1	1	1	1	1	1	1	1	1	1	0	0	1	1	0	1	1
energyVRNAandronescu2007	17	0	0	0	1	1	1	0	0	1	0	0	0	0	0	0	0	0	0	0	0	0	1	1	0	1	0	1	1	0	1	1	1	0	1	0	0	0	1	1	0	0	0	1	0	0	0	1
energyVRNAlangdon2018	14	0	0	0	1	1	1	1	0	1	0	0	0	0	0	0	1	0	0	0	0	0	1	1	0	1	0	0	1	0	0	0	1	0	0	0	1	0	0	0	0	0	0	1	0	0	0	1
energyVRNAturner1999	19	0	0	0	1	1	1	0	0	1	0	0	0	0	0	0	1	0	0	1	0	1	1	1	0	1	0	1	1	1	1	0	1	0	0	0	0	0	1	1	0	0	0	1	0	0	0	1
energyVRNAturner2004	16	0	0	0	1	1	1	0	0	1	0	0	0	0	0	0	0	0	0	1	0	0	1	1	0	1	0	0	1	0	1	0	1	0	1	0	0	0	1	1	0	0	0	1	0	0	0	1
final.and.seedBP7	17	0	0	0	1	1	1	0	0	1	0	0	0	0	0	0	0	0	0	0	0	0	1	1	0	1	0	1	1	0	1	1	1	0	1	0	0	0	1	1	0	0	0	1	0	0	0	1
final.and.seedBP7.seedNoGU	20	0	0	0	1	1	1	1	0	1	0	1	0	0	0	0	1	0	0	0	0	1	1	1	0	1	0	1	1	0	1	0	0	1	1	0	0	1	1	0	0	0	0	1	0	0	0	1
final.and.seedBP7.seedNoGU.seedMinPu0.001	20	0	0	0	1	1	1	1	0	1	0	1	0	0	0	0	1	0	0	0	0	1	1	1	0	1	0	1	1	0	1	0	0	1	1	0	0	1	1	0	0	0	0	1	0	0	0	1
final.and.seedBP7.seedNoGU.seedMinPu0.001.intLenMax60	20	0	0	0	1	1	1	1	0	1	0	1	0	0	0	0	1	0	0	0	0	1	1	1	0	1	0	1	1	0	1	0	0	1	1	0	0	1	1	0	0	0	0	1	0	0	0	1
final.and.seedBP7.seedNoGU.seedMinPu0.001.intLenMax60.intLoopMax2	16	0	0	0	1	1	0	0	0	1	0	1	0	0	0	0	1	0	0	0	0	1	1	1	0	1	0	0	1	0	1	0	0	1	0	0	0	1	1	0	0	0	0	1	0	0	0	1
final.and.seedBP7.seedNoGU.seedMinPu0.001.intLenMax60.intLoopMax2.outMinPu0.001	16	0	0	0	1	1	0	0	0	1	0	1	0	0	0	0	1	0	0	0	0	1	1	1	0	1	0	0	1	0	1	0	0	1	0	0	0	1	1	0	0	0	0	1	0	0	0	1
final.and.seedBP7.seedNoGU.seedMinPu0.001.intLenMax60.intLoopMax5	17	0	0	0	1	1	0	1	0	1	0	0	0	0	0	0	1	0	0	0	0	1	1	1	0	1	0	1	1	0	1	0	0	1	0	0	0	1	1	0	0	0	0	1	0	0	0	1
final.and.seedBP7.seedNoGU.seedMinPu0.001.intLenMax60.intLoopMax5.outMinPu0.001	17	0	0	0	1	1	0	1	0	1	0	0	0	0	0	0	1	0	0	0	0	1	1	1	0	1	0	1	1	0	1	0	0	1	0	0	0	1	1	0	0	0	0	1	0	0	0	1
final.and.seedBP7.seedNoGU.seedMinPu0.001.intLenMax60.intLoopMax7	19	0	0	0	1	1	0	1	0	1	0	1	0	0	0	0	1	0	0	0	0	1	1	1	0	1	0	1	1	0	1	0	0	1	1	0	0	1	1	0	0	0	0	1	0	0	0	1
final.and.seedBP7.seedNoGU.seedMinPu0.001.intLenMax60.intLoopMax7.outMinPu0.001	18	0	0	0	1	1	0	1	0	1	0	1	0	0	0	0	1	0	0	0	0	1	1	1	0	1	0	1	1	0	1	0	0	1	0	0	0	1	1	0	0	0	0	1	0	0	0	1
final.and.seedBP7.seedNoGU.seedMinPu0.001.intLenMax60.intLoopMax8	19	0	0	0	1	1	0	1	0	1	0	1	0	0	0	0	1	0	0	0	0	1	1	1	0	1	0	1	1	0	1	0	0	1	1	0	0	1	1	0	0	0	0	1	0	0	0	1
final.and.seedBP7.seedNoGU.seedMinPu0.001.intLenMax60.intLoopMax8.outMinPu0.001	18	0	0	0	1	1	0	1	0	1	0	1	0	0	0	0	1	0	0	0	0	1	1	1	0	1	0	1	1	0	1	0	0	1	0	0	0	1	1	0	0	0	0	1	0	0	0	1
final.and.seedBP7.seedNoGU.seedMinPu0.001.intLenMax60.intLoopMax10	19	0	0	0	1	1	0	1	0	1	0	1	0	0	0	0	1	0	0	0	0	1	1	1	0	1	0	1	1	0	1	0	0	1	1	0	0	1	1	0	0	0	0	1	0	0	0	1
final.and.seedBP7.seedNoGU.seedMinPu0.001.intLenMax60.intLoopMax10.outMinPu0.001	18	0	0	0	1	1	0	1	0	1	0	1	0	0	0	0	1	0	0	0	0	1	1	1	0	1	0	1	1	0	1	0	0	1	0	0	0	1	1	0	0	0	0	1	0	0	0	1
final.seedBP7	16	0	0	0	1	1	1	0	0	1	0	0	0	0	0	0	0	0	0	1	0	0	1	1	0	1	0	0	1	0	1	0	1	0	1	0	0	0	1	1	0	0	0	1	0	0	0	1
final.seedBP7.seedNoGU	17	0	0	0	1	1	1	0	0	1	0	1	0	0	0	0	1	0	0	1	0	1	1	1	0	1	0	0	1	0	1	0	0	0	1	0	0	0	1	0	0	0	0	1	0	0	0	1
final.seedBP7.seedNoGU.seedMinPu0.001	18	0	0	0	1	1	1	0	0	1	0	1	0	0	0	0	1	0	0	1	0	1	1	1	0	1	0	1	1	0	1	0	0	0	1	0	0	0	1	0	0	0	0	1	0	0	0	1
final.seedBP7.seedNoGU.seedMinPu0.001.intLenMax60	18	0	0	0	1	1	1	0	0	1	0	1	0	0	0	0	1	0	0	1	0	1	1	1	0	1	0	1	1	0	1	0	0	0	1	0	0	0	1	0	0	0	0	1	0	0	0	1
final.seedBP7.seedNoGU.seedMinPu0.001.intLenMax60.intLoopMax2	15	0	0	0	1	1	0	0	0	1	0	0	0	0	0	0	1	1	0	1	0	1	1	1	0	1	0	0	0	0	1	0	0	1	0	0	0	0	1	0	0	0	0	1	0	0	0	1
final.seedBP7.seedNoGU.seedMinPu0.001.intLenMax60.intLoopMax2.outMinPu0.001	15	0	0	0	1	1	0	0	0	1	0	0	0	0	0	0	1	1	0	1	0	1	1	1	0	1	0	0	0	0	1	0	0	1	0	0	0	0	1	0	0	0	0	1	0	0	0	1
final.seedBP7.seedNoGU.seedMinPu0.001.intLenMax60.intLoopMax5	16	0	0	0	1	1	0	0	0	1	0	1	0	0	0	0	1	0	0	1	0	1	1	1	0	1	0	1	0	0	1	0	0	0	1	0	0	0	1	0	0	0	0	1	0	0	0	1
final.seedBP7.seedNoGU.seedMinPu0.001.intLenMax60.intLoopMax5.outMinPu0.001	17	0	0	0	1	1	0	0	0	1	0	1	0	0	0	0	1	0	0	1	0	1	1	1	0	1	0	1	0	0	1	0	0	1	1	0	0	0	1	0	0	0	0	1	0	0	0	1
final.seedBP7.seedNoGU.seedMinPu0.001.intLenMax60.intLoopMax7	17	0	0	0	1	1	0	0	0	1	0	1	0	0	0	0	1	0	0	1	0	1	1	1	0	1	0	1	1	0	1	0	0	0	1	0	0	0	1	0	0	0	0	1	0	0	0	1
final.seedBP7.seedNoGU.seedMinPu0.001.intLenMax60.intLoopMax7.outMinPu0.001	18	0	0	0	1	1	0	0	0	1	0	1	0	0	0	0	1	0	0	1	0	1	1	1	0	1	0	1	1	0	1	0	0	1	1	0	0	0	1	0	0	0	0	1	0	0	0	1
final.seedBP7.seedNoGU.seedMinPu0.001.intLenMax60.intLoopMax8	18	0	0	0	1	1	0	0	0	1	0	1	0	0	0	0	1	0	0	1	0	1	1	1	0	1	1	1	1	0	1	0	0	0	1	0	0	0	1	0	0	0	0	1	0	0	0	1
final.seedBP7.seedNoGU.seedMinPu0.001.intLenMax60.intLoopMax8.outMinPu0.001	19	0	0	0	1	1	0	0	0	1	0	1	0	0	0	0	1	0	0	1	0	1	1	1	0	1	1	1	1	0	1	0	0	1	1	0	0	0	1	0	0	0	0	1	0	0	0	1
final.seedBP7.seedNoGU.seedMinPu0.001.intLenMax60.intLoopMax10	18	0	0	0	1	1	0	0	0	1	0	1	0	0	0	0	1	0	0	1	0	1	1	1	0	1	1	1	1	0	1	0	0	0	1	0	0	0	1	0	0	0	0	1	0	0	0	1
final.seedBP7.seedNoGU.seedMinPu0.001.intLenMax60.intLoopMax10.outMinPu0.001	19	0	0	0	1	1	0	0	0	1	0	1	0	0	0	0	1	0	0	1	0	1	1	1	0	1	1	1	1	0	1	0	0	1	1	0	0	0	1	0	0	0	0	1	0	0	0	1
intLenMax10	14	0	0	0	1	1	0	1	0	0	0	0	0	0	0	0	0	0	0	0	0	0	0	0	0	0	0	0	1	0	1	1	1	1	1	0	1	0	0	1	0	0	0	1	1	0	0	1
intLenMax15	12	0	0	0	1	0	0	0	0	0	0	1	0	0	0	0	0	0	0	0	0	0	0	0	0	1	0	0	0	0	1	1	1	1	0	0	0	0	1	1	0	0	0	1	0	0	1	1
intLenMax20	14	0	0	0	1	1	0	0	0	0	0	1	0	0	0	0	1	1	0	0	0	0	0	0	0	1	0	0	0	0	1	1	1	1	0	0	0	0	1	1	0	0	0	1	0	0	0	1
intLenMax25	15	0	0	0	1	1	0	1	0	0	0	1	0	0	0	0	1	1	0	0	0	1	0	0	0	1	0	0	1	0	1	1	1	0	0	0	0	0	1	0	0	0	0	1	0	0	0	1
intLenMax30	14	0	0	0	1	1	0	0	0	0	0	0	0	0	0	0	1	1	0	0	0	0	0	1	0	1	0	0	1	0	1	1	1	0	0	0	0	0	1	1	0	0	0	1	0	0	0	1
intLenMax40	15	0	0	0	1	1	0	0	0	0	0	0	0	0	0	0	1	0	0	0	0	0	1	1	0	1	0	0	1	0	1	1	1	0	1	0	0	0	1	1	0	0	0	1	0	0	0	1
intLenMax50	18	0	0	0	1	1	1	0	0	1	0	1	0	0	0	0	0	0	0	1	0	0	1	1	0	1	0	0	1	0	1	1	1	0	1	0	0	0	1	1	0	0	0	1	0	0	0	1
intLenMax60	18	0	0	0	1	1	1	0	0	1	0	1	0	0	0	0	0	0	0	1	0	1	1	1	0	1	0	0	1	0	1	0	1	0	1	0	0	0	1	1	0	0	0	1	0	0	0	1
intLenMax70	17	0	0	0	1	1	1	0	0	1	0	0	0	0	0	0	0	0	0	1	0	1	1	1	0	1	0	0	1	0	1	0	1	0	1	0	0	0	1	1	0	0	0	1	0	0	0	1
intLenMax75	17	0	0	0	1	1	1	0	0	1	0	0	0	0	0	0	0	0	0	1	0	1	1	1	0	1	0	0	1	0	1	0	1	0	1	0	0	0	1	1	0	0	0	1	0	0	0	1
intLenMax80	16	0	0	0	1	1	1	0	0	1	0	0	0	0	0	0	0	0	0	1	0	0	1	1	0	1	0	0	1	0	1	0	1	0	1	0	0	0	1	1	0	0	0	1	0	0	0	1
intLenMax100	16	0	0	0	1	1	1	0	0	1	0	0	0	0	0	0	0	0	0	1	0	0	1	1	0	1	0	0	1	0	1	0	1	0	1	0	0	0	1	1	0	0	0	1	0	0	0	1
intLenMax150	16	0	0	0	1	1	1	0	0	1	0	0	0	0	0	0	0	0	0	1	0	0	1	1	0	1	0	0	1	0	1	0	1	0	1	0	0	0	1	1	0	0	0	1	0	0	0	1
intLoopMax2	14	0	0	0	1	1	0	0	0	1	0	0	0	0	0	0	1	0	0	1	0	0	0	1	0	1	0	0	1	0	1	0	1	0	0	0	0	0	1	1	0	0	0	1	0	0	0	1
intLoopMax3	14	0	0	0	1	1	0	0	0	1	0	0	0	0	0	0	1	0	0	1	0	0	0	1	0	1	0	0	1	0	1	0	1	0	0	0	0	0	1	1	0	0	0	1	0	0	0	1
intLoopMax4	17	0	0	0	1	1	0	0	0	1	0	1	0	0	0	0	1	0	0	1	0	1	1	1	0	1	0	0	1	0	1	0	1	0	0	0	0	0	1	1	0	0	0	1	0	0	0	1
intLoopMax5	17	0	0	0	1	1	0	0	0	1	0	1	0	0	0	0	1	0	0	1	0	1	0	1	0	1	0	0	1	0	1	1	1	0	0	0	0	0	1	1	0	0	0	1	0	0	0	1
intLoopMax6	17	0	0	0	1	1	0	0	0	1	0	1	0	0	0	0	0	0	0	1	0	1	0	1	0	1	0	0	1	0	1	1	1	0	1	0	0	0	1	1	0	0	0	1	0	0	0	1
intLoopMax7	17	0	0	0	1	1	0	0	0	1	0	1	0	0	0	0	0	0	0	1	0	1	0	1	0	1	0	0	1	0	1	1	1	0	1	0	0	0	1	1	0	0	0	1	0	0	0	1
intLoopMax8	17	0	0	0	1	1	0	0	0	1	0	0	0	0	0	0	0	0	0	1	0	1	1	1	0	1	0	0	1	0	1	1	1	0	1	0	0	0	1	1	0	0	0	1	0	0	0	1
intLoopMax9	16	0	0	0	1	1	0	0	0	1	0	0	0	0	0	0	0	0	0	1	0	1	1	1	0	1	0	0	1	0	1	0	1	0	1	0	0	0	1	1	0	0	0	1	0	0	0	1
intLoopMax10	15	0	0	0	1	1	0	0	0	1	0	0	0	0	0	0	0	0	0	1	0	0	1	1	0	1	0	0	1	0	1	0	1	0	1	0	0	0	1	1	0	0	0	1	0	0	0	1
intLoopMax11	16	0	0	0	1	1	1	0	0	1	0	0	0	0	0	0	0	0	0	1	0	0	1	1	0	1	0	0	1	0	1	0	1	0	1	0	0	0	1	1	0	0	0	1	0	0	0	1
intLoopMax12	16	0	0	0	1	1	1	0	0	1	0	0	0	0	0	0	0	0	0	1	0	0	1	1	0	1	0	0	1	0	1	0	1	0	1	0	0	0	1	1	0	0	0	1	0	0	0	1

Salmonella typhimurium NC_003197

	sum	ArcZ-STM1682	ArcZ-STM2970	ArcZ-STM3216	ChiX-STM0687	ChiX-STM1313	CyaR-STM0833	GcvB-STM0002	GcvB-STM0245	GcvB-STM0399	GcvB-STM0602	GcvB-STM0665	GcvB-STM0959	GcvB-STM1299	GcvB-STM1452	GcvB-STM1746	GcvB-STM2355	GcvB-STM2526	GcvB-STM3062	GcvB-STM3064	GcvB-STM3225	GcvB-STM3564	GcvB-STM3567	GcvB-STM3630	GcvB-STM3903	GcvB-STM3909	GcvB-STM3930	GcvB-STM4351	GcvB-STM4398	MicA-STM4231	MicC-STM1572	MicF-STM0366	MicF-STM0959	MicF-STM1328	RybB-STM0413	RybB-STM0687	RybB-STM0999	RybB-STM1070	RybB-STM1473	RybB-STM1530	RybB-STM1572	RybB-STM1732	RybB-STM1995	RybB-STM2267	RybB-STM2391	SgrS-STM2945	SgrS-STM3962	Spot42-STM2190
Target found among top-100 predictions for the sRNA for any tested parameterization	40	1	0	0	1	1	1	1	1	1	1	1	1	1	0	0	1	1	1	1	1	1	1	1	1	1	1	1	1	1	1	1	1	1	1	1	1	1	1	1	1	0	0	1	1	0	1	1
intLoopMax14	16	0	0	0	1	1	1	0	0	1	0	0	0	0	0	0	0	0	0	1	0	0	1	1	0	1	0	0	1	0	1	0	1	0	1	0	0	0	1	1	0	0	0	1	0	0	0	1
intLoopMax16	16	0	0	0	1	1	1	0	0	1	0	0	0	0	0	0	0	0	0	1	0	0	1	1	0	1	0	0	1	0	1	0	1	0	1	0	0	0	1	1	0	0	0	1	0	0	0	1
noSeed	16	0	0	0	1	1	1	0	0	1	0	0	0	0	0	0	0	0	0	1	0	0	1	1	0	1	0	0	1	0	1	0	1	0	1	0	0	0	1	1	0	0	0	1	0	0	0	1
outMaxE0	16	0	0	0	1	1	1	0	0	1	0	0	0	0	0	0	0	0	0	1	0	0	1	1	0	1	0	0	1	0	1	0	1	0	1	0	0	0	1	1	0	0	0	1	0	0	0	1
outMaxE-1	16	0	0	0	1	1	1	0	0	1	0	0	0	0	0	0	0	0	0	1	0	0	1	1	0	1	0	0	1	0	1	0	1	0	1	0	0	0	1	1	0	0	0	1	0	0	0	1
outMaxE-2	16	0	0	0	1	1	1	0	0	1	0	0	0	0	0	0	0	0	0	1	0	0	1	1	0	1	0	0	1	0	1	0	1	0	1	0	0	0	1	1	0	0	0	1	0	0	0	1
outMaxE-3	16	0	0	0	1	1	1	0	0	1	0	0	0	0	0	0	0	0	0	1	0	0	1	1	0	1	0	0	1	0	1	0	1	0	1	0	0	0	1	1	0	0	0	1	0	0	0	1
outMaxE-4	16	0	0	0	1	1	1	0	0	1	0	0	0	0	0	0	0	0	0	1	0	0	1	1	0	1	0	0	1	0	1	0	1	0	1	0	0	0	1	1	0	0	0	1	0	0	0	1
outMaxE-5	16	0	0	0	1	1	1	0	0	1	0	0	0	0	0	0	0	0	0	1	0	0	1	1	0	1	0	0	1	0	1	0	1	0	1	0	0	0	1	1	0	0	0	1	0	0	0	1
outMaxE-6	16	0	0	0	1	1	1	0	0	1	0	0	0	0	0	0	0	0	0	1	0	0	1	1	0	1	0	0	1	0	1	0	1	0	1	0	0	0	1	1	0	0	0	1	0	0	0	1
outMaxE-7	16	0	0	0	1	1	1	0	0	1	0	0	0	0	0	0	0	0	0	1	0	0	1	1	0	1	0	0	1	0	1	0	1	0	1	0	0	0	1	1	0	0	0	1	0	0	0	1
outMaxE-8	16	0	0	0	1	1	1	0	0	1	0	0	0	0	0	0	0	0	0	1	0	0	1	1	0	1	0	0	1	0	1	0	1	0	1	0	0	0	1	1	0	0	0	1	0	0	0	1
outMaxE-9	16	0	0	0	1	1	1	0	0	1	0	0	0	0	0	0	0	0	0	1	0	0	1	1	0	1	0	0	1	0	1	0	1	0	1	0	0	0	1	1	0	0	0	1	0	0	0	1
outMaxE-10	16	0	0	0	1	1	1	0	0	1	0	0	0	0	0	0	0	0	0	1	0	0	1	1	0	1	0	0	1	0	1	0	1	0	1	0	0	0	1	1	0	0	0	1	0	0	0	1
outMaxE-11	15	0	0	0	1	1	1	0	0	1	0	0	0	0	0	0	0	0	0	1	0	0	1	1	0	1	0	0	1	0	1	0	1	0	1	0	0	0	1	1	0	0	0	1	0	0	0	0
outMaxE-12	15	0	0	0	1	1	1	0	0	1	0	0	0	0	0	0	0	0	0	1	0	0	1	1	0	1	0	0	1	0	1	0	1	0	1	0	0	0	1	1	0	0	0	1	0	0	0	0
outMaxE-14	14	0	0	0	1	1	1	0	0	1	0	0	0	0	0	0	0	0	0	1	0	0	1	1	0	1	0	0	1	0	0	0	1	0	1	0	0	0	1	1	0	0	0	1	0	0	0	0
outMaxE-16	11	0	0	0	0	1	0	0	0	1	0	0	0	0	0	0	0	0	0	1	0	0	1	1	0	1	0	0	1	0	0	0	1	0	1	0	0	0	1	1	0	0	0	0	0	0	0	0
outMaxE-18	7	0	0	0	0	0	0	0	0	1	0	0	0	0	0	0	0	0	0	1	0	0	1	1	0	1	0	0	1	0	0	0	1	0	0	0	0	0	0	0	0	0	0	0	0	0	0	0
outMaxE-20	4	0	0	0	0	0	0	0	0	1	0	0	0	0	0	0	0	0	0	0	0	0	0	1	0	1	0	0	1	0	0	0	0	0	0	0	0	0	0	0	0	0	0	0	0	0	0	0
outMinPu0	16	0	0	0	1	1	1	0	0	1	0	0	0	0	0	0	0	0	0	1	0	0	1	1	0	1	0	0	1	0	1	0	1	0	1	0	0	0	1	1	0	0	0	1	0	0	0	1
outMinPu0.1	17	0	0	0	1	1	0	1	0	0	1	0	1	0	0	0	1	1	0	0	0	0	0	1	0	1	0	0	1	0	1	1	1	1	0	0	0	0	1	1	0	0	0	1	0	0	0	0
outMinPu0.001	17	0	0	0	1	1	1	0	0	1	0	0	0	0	0	0	0	0	0	1	0	0	1	1	0	1	0	0	1	0	1	1	1	0	1	0	0	0	1	1	0	0	0	1	0	0	0	1
outMinPu0.0001	16	0	0	0	1	1	1	0	0	1	0	0	0	0	0	0	0	0	0	1	0	0	1	1	0	1	0	0	1	0	1	0	1	0	1	0	0	0	1	1	0	0	0	1	0	0	0	1
outMinPu0.01	13	0	0	0	1	1	0	0	0	0	0	0	0	0	0	0	1	0	0	0	0	0	0	1	0	1	0	0	1	0	1	1	1	0	0	0	0	0	1	1	0	0	0	1	0	0	0	1
outMinPu0.2	11	0	0	0	1	1	0	1	0	0	0	0	0	0	0	0	0	0	0	0	0	0	0	1	0	0	0	0	1	0	1	1	1	1	0	0	0	0	1	1	0	0	0	0	0	0	0	0
outMinPu0.5	1	0	0	0	0	0	0	0	0	0	0	0	0	0	0	0	0	0	0	0	0	0	0	0	0	0	0	0	0	0	0	0	0	0	0	0	0	0	1	0	0	0	0	0	0	0	0	0
seedBP4	15	0	0	0	1	1	1	0	0	1	0	0	0	0	0	0	0	0	0	1	0	0	0	1	0	1	0	0	1	0	1	0	1	0	1	0	0	0	1	1	0	0	0	1	0	0	0	1
seedBP4.seedMinPu0	15	0	0	0	1	1	1	0	0	1	0	0	0	0	0	0	0	0	0	1	0	0	0	1	0	1	0	0	1	0	1	0	1	0	1	0	0	0	1	1	0	0	0	1	0	0	0	1
seedBP4.seedMinPu0.001	15	0	0	0	1	1	1	0	0	1	0	0	0	0	0	0	0	0	0	1	0	0	0	1	0	1	0	0	1	0	1	0	1	0	1	0	0	0	1	1	0	0	0	1	0	0	0	1
seedBP4.seedMinPu0.0001	15	0	0	0	1	1	1	0	0	1	0	0	0	0	0	0	0	0	0	1	0	0	0	1	0	1	0	0	1	0	1	0	1	0	1	0	0	0	1	1	0	0	0	1	0	0	0	1
seedBP4.seedMinPu0.1	17	0	0	0	1	1	1	0	0	1	0	0	0	0	0	0	0	0	0	1	0	1	1	1	0	1	0	0	1	0	1	0	1	0	1	0	0	0	1	1	0	0	0	1	0	0	0	1
seedBP4.seedMinPu0.01	16	0	0	0	1	1	1	0	0	1	0	0	0	0	0	0	0	0	0	1	0	0	1	1	0	1	0	0	1	0	1	0	1	0	1	0	0	0	1	1	0	0	0	1	0	0	0	1
seedBP4.seedMinPu0.1.seedNoGU	20	0	0	0	1	1	1	0	0	1	0	1	0	0	0	0	1	0	0	1	0	1	1	1	0	1	0	0	1	0	1	1	1	0	1	0	0	0	1	1	0	0	0	1	0	0	0	1
seedBP4.seedMinPu0.01.seedNoGU	18	0	0	0	1	1	1	0	0	1	0	1	0	0	0	0	0	0	0	1	0	1	1	1	0	1	0	0	1	0	1	0	1	0	1	0	0	0	1	1	0	0	0	1	0	0	0	1
seedBP4.seedMinPu0.0001.seedNoGU	16	0	0	0	1	1	1	0	0	1	0	0	0	0	0	0	0	0	0	1	0	0	1	1	0	1	0	0	1	0	1	0	1	0	1	0	0	0	1	1	0	0	0	1	0	0	0	1
seedBP4.seedMinPu0.001.seedNoGU	16	0	0	0	1	1	1	0	0	1	0	0	0	0	0	0	0	0	0	1	0	0	1	1	0	1	0	0	1	0	1	0	1	0	1	0	0	0	1	1	0	0	0	1	0	0	0	1
seedBP4.seedMinPu0.2	15	0	0	0	0	0	1	0	0	1	0	1	0	0	0	0	1	0	0	0	0	1	1	1	0	1	0	0	1	0	1	1	1	0	1	0	0	0	1	1	0	0	0	0	0	0	0	0
seedBP4.seedMinPu0.2.seedNoGU	15	0	0	0	0	0	1	0	0	1	0	1	0	0	0	0	1	0	0	0	0	1	1	1	0	1	0	0	1	0	1	1	1	0	1	0	0	0	1	1	0	0	0	0	0	0	0	0
seedBP4.seedMinPu0.5	9	0	0	0	0	0	0	1	0	0	0	0	0	0	0	0	0	1	0	0	0	1	0	1	0	0	0	1	0	0	0	0	1	0	1	0	0	0	1	1	0	0	0	0	0	0	0	0
seedBP4.seedMinPu0.5.seedNoGU	11	1	0	0	0	0	0	1	0	0	1	0	0	0	0	0	0	1	0	0	0	1	0	1	0	0	0	1	1	0	0	0	0	0	1	0	0	0	1	1	0	0	0	0	0	0	0	0
seedBP4.seedMinPu0.seedNoGU	16	0	0	0	1	1	1	0	0	1	0	0	0	0	0	0	0	0	0	1	0	0	1	1	0	1	0	0	1	0	1	0	1	0	1	0	0	0	1	1	0	0	0	1	0	0	0	1
seedBP4.seedNoGU	16	0	0	0	1	1	1	0	0	1	0	0	0	0	0	0	0	0	0	1	0	0	1	1	0	1	0	0	1	0	1	0	1	0	1	0	0	0	1	1	0	0	0	1	0	0	0	1
seedBP5	15	0	0	0	1	1	1	0	0	1	0	0	0	0	0	0	0	0	0	1	0	0	0	1	0	1	0	0	1	0	1	0	1	0	1	0	0	0	1	1	0	0	0	1	0	0	0	1
seedBP5.seedMinPu0	15	0	0	0	1	1	1	0	0	1	0	0	0	0	0	0	0	0	0	1	0	0	0	1	0	1	0	0	1	0	1	0	1	0	1	0	0	0	1	1	0	0	0	1	0	0	0	1
seedBP5.seedMinPu0.01	16	0	0	0	1	1	1	0	0	1	0	0	0	0	0	0	0	0	0	1	0	0	1	1	0	1	0	0	1	0	1	0	1	0	1	0	0	0	1	1	0	0	0	1	0	0	0	1
seedBP5.seedMinPu0.001	15	0	0	0	1	1	1	0	0	1	0	0	0	0	0	0	0	0	0	1	0	0	0	1	0	1	0	0	1	0	1	0	1	0	1	0	0	0	1	1	0	0	0	1	0	0	0	1
seedBP5.seedMinPu0.0001	15	0	0	0	1	1	1	0	0	1	0	0	0	0	0	0	0	0	0	1	0	0	0	1	0	1	0	0	1	0	1	0	1	0	1	0	0	0	1	1	0	0	0	1	0	0	0	1
seedBP5.seedMinPu0.1	17	0	0	0	1	1	1	0	0	1	0	1	0	0	0	0	0	0	0	1	0	1	1	1	0	1	0	0	1	0	1	1	1	0	1	0	0	0	1	1	0	0	0	0	0	0	0	0
seedBP5.seedMinPu0.001.seedNoGU	19	0	0	0	1	1	1	0	0	1	0	1	0	0	0	0	0	0	0	1	0	1	1	1	0	1	0	0	1	0	1	1	1	0	1	0	0	0	1	1	0	0	0	1	0	0	0	1
seedBP5.seedMinPu0.0001.seedNoGU	18	0	0	0	1	1	1	0	0	1	0	1	0	0	0	0	0	0	0	1	0	1	1	1	0	1	0	0	1	0	1	0	1	0	1	0	0	0	1	1	0	0	0	1	0	0	0	1
seedBP5.seedMinPu0.1.seedNoGU	19	0	0	0	1	1	1	0	0	1	0	1	0	0	0	0	1	0	0	1	0	1	1	1	0	1	0	1	1	0	1	1	1	0	1	0	0	0	1	1	0	0	0	0	0	0	0	0
seedBP5.seedMinPu0.01.seedNoGU	19	0	0	0	1	1	1	0	0	1	0	1	0	0	0	0	1	0	0	1	0	0	1	1	0	1	0	0	1	0	1	1	1	0	1	0	0	0	1	1	0	0	0	1	0	0	0	1
seedBP5.seedMinPu0.2	14	0	0	0	0	0	0	0	0	1	0	1	0	0	0	0	1	0	0	0	0	1	1	1	0	1	0	0	1	0	1	1	1	0	1	0	0	0	1	1	0	0	0	0	0	0	0	0
seedBP5.seedMinPu0.2.seedNoGU	16	0	0	0	0	0	0	1	0	1	0	1	0	0	0	0	1	0	0	0	0	0	1	1	0	1	1	1	1	0	1	1	1	0	1	0	0	0	1	1	0	0	0	0	0	0	0	0
seedBP5.seedMinPu0.5	7	0	0	0	0	0	0	1	0	0	0	0	1	0	0	0	0	0	0	0	0	1	0	0	0	0	0	0	0	0	0	0	1	0	1	0	0	0	1	1	0	0	0	0	0	0	0	0

Salmonella typhimurium *NC_003197*

	sum	ArcZ-STM1682	ArcZ-STM2970	ArcZ-STM3216	ChiX-STM0687	ChiX-STM1313	CyaR-STM0833	GcvB-STM0002	GcvB-STM0245	GcvB-STM0399	GcvB-STM0602	GcvB-STM0665	GcvB-STM0959	GcvB-STM1299	GcvB-STM1452	GcvB-STM1746	GcvB-STM2355	GcvB-STM2526	GcvB-STM3062	GcvB-STM3064	GcvB-STM3225	GcvB-STM3564	GcvB-STM3567	GcvB-STM3630	GcvB-STM3903	GcvB-STM3909	GcvB-STM3930	GcvB-STM4351	GcvB-STM4398	MicA-STM4231	MicC-STM1572	MicF-STM0366	MicF-STM0959	MicF-STM1328	RybB-STM0413	RybB-STM0687	RybB-STM0999	RybB-STM1070	RybB-STM1473	RybB-STM1530	RybB-STM1572	RybB-STM1732	RybB-STM1995	RybB-STM2267	RybB-STM2391	SgrS-STM2945	SgrS-STM3962	Spot42-STM2190
Target found among top-100 predictions for the sRNA for any tested parameterization	40	1	0	0	1	1	1	1	1	1	1	1	1	1	0	0	1	1	1	1	1	1	1	1	1	1	1	1	1	1	1	1	1	1	1	1	1	1	1	1	1	0	0	1	1	0	1	1
seedBP5.seedMinPu0.5.seedNoGU	6	0	0	0	0	0	0	1	0	0	0	0	1	0	0	0	0	0	0	0	0	0	0	0	0	0	0	0	0	0	0	0	1	0	1	0	0	0	1	1	0	0	0	0	0	0	0	0
seedBP5.seedMinPu0.seedNoGU	18	0	0	0	1	1	1	0	0	1	0	1	0	0	0	0	0	0	0	1	0	1	1	1	0	1	0	0	1	0	1	0	1	0	1	0	0	0	1	1	0	0	0	1	0	0	0	1
seedBP5.seedNoGU	18	0	0	0	1	1	1	0	0	1	0	1	0	0	0	0	0	0	0	1	0	1	1	1	0	1	0	0	1	0	1	0	1	0	1	0	0	0	1	1	0	0	0	1	0	0	0	1
seedBP6	15	0	0	0	1	1	1	0	0	1	0	0	0	0	0	0	0	0	0	1	0	0	0	1	0	1	0	0	1	0	1	0	1	0	1	0	0	0	1	1	0	0	0	1	0	0	0	1
seedBP6.seedMinPu0	15	0	0	0	1	1	1	0	0	1	0	0	0	0	0	0	0	0	0	1	0	0	0	1	0	1	0	0	1	0	1	0	1	0	1	0	0	0	1	1	0	0	0	1	0	0	0	1
seedBP6.seedMinPu0.1	16	0	0	0	1	1	0	0	0	1	0	1	0	0	0	0	1	0	0	1	0	1	1	1	0	1	0	0	1	0	1	1	1	0	0	0	0	0	1	1	0	0	0	0	0	0	0	0
seedBP6.seedMinPu0.0001	15	0	0	0	1	1	1	0	0	1	0	0	0	0	0	0	0	0	0	1	0	0	0	1	0	1	0	0	1	0	1	0	1	0	1	0	0	0	1	1	0	0	0	1	0	0	0	1
seedBP6.seedMinPu0.001	15	0	0	0	1	1	1	0	0	1	0	0	0	0	0	0	0	0	0	1	0	0	0	1	0	1	0	0	1	0	1	0	1	0	1	0	0	0	1	1	0	0	0	1	0	0	0	1
seedBP6.seedMinPu0.01	16	0	0	0	1	1	1	0	0	1	0	0	0	0	0	0	0	0	0	1	0	0	1	1	0	1	0	0	1	0	1	0	1	0	1	0	0	0	1	1	0	0	0	1	0	0	0	1
seedBP6.seedMinPu0.0001.seedNoGU	19	0	0	0	1	1	1	0	0	1	0	1	0	0	0	0	1	0	0	1	0	1	1	1	0	1	0	0	1	0	1	1	1	0	1	0	0	0	1	0	0	0	0	1	0	0	0	1
seedBP6.seedMinPu0.1.seedNoGU	18	0	0	0	1	1	0	1	0	1	0	1	0	0	0	0	1	0	0	1	0	0	1	1	0	1	1	1	1	0	1	1	1	0	0	0	0	0	1	1	0	0	0	0	0	0	0	0
seedBP6.seedMinPu0.01.seedNoGU	18	0	0	0	1	1	1	0	0	1	0	1	0	0	0	0	1	0	0	1	0	0	1	1	0	1	0	0	1	0	1	1	1	0	1	0	0	0	1	0	0	0	0	1	0	0	0	1
seedBP6.seedMinPu0.001.seedNoGU	19	0	0	0	1	1	1	0	0	1	0	1	0	0	0	0	1	0	0	1	0	1	1	1	0	1	0	0	1	0	1	1	1	0	1	0	0	0	1	0	0	0	0	1	0	0	0	1
seedBP6.seedMinPu0.2	15	0	0	0	0	0	0	1	0	1	0	1	0	1	0	0	1	0	0	0	0	1	0	1	0	1	1	1	0	0	1	1	1	0	0	0	0	0	1	1	0	0	0	0	0	0	0	0
seedBP6.seedMinPu0.2.seedNoGU	12	0	0	0	0	0	0	1	0	1	1	1	0	0	0	0	1	0	0	1	0	0	0	1	0	1	0	0	0	0	1	0	1	0	0	0	0	0	1	1	0	0	0	0	0	0	0	0
seedBP6.seedMinPu0.5	2	0	0	0	0	0	0	0	0	0	0	0	1	0	0	0	0	0	0	0	0	0	1	0	0	0	0	0	0	0	0	0	0	0	0	0	0	0	0	0	0	0	0	0	0	0	0	0
seedBP6.seedMinPu0.5.seedNoGU	1	0	0	0	0	0	0	0	0	0	0	0	1	0	0	0	0	0	0	0	0	0	0	0	0	0	0	0	0	0	0	0	0	0	0	0	0	0	0	0	0	0	0	0	0	0	0	0
seedBP6.seedMinPu0.seedNoGU	19	0	0	0	1	1	1	0	0	1	0	1	0	0	0	0	1	0	0	1	0	1	1	1	0	1	0	0	1	0	1	1	1	0	1	0	0	0	1	0	0	0	0	1	0	0	0	1
seedBP6.seedNoGU	19	0	0	0	1	1	1	0	0	1	0	1	0	0	0	0	1	0	0	1	0	1	1	1	0	1	0	0	1	0	1	1	1	0	1	0	0	0	1	0	0	0	0	1	0	0	0	1
seedBP7	16	0	0	0	1	1	1	0	0	1	0	0	0	0	0	0	0	0	0	1	0	0	1	1	0	1	0	0	1	0	1	0	1	0	1	0	0	0	1	1	0	0	0	1	0	0	0	1
seedBP7.seedMinPu0	16	0	0	0	1	1	1	0	0	1	0	0	0	0	0	0	0	0	0	1	0	0	1	1	0	1	0	0	1	0	1	0	1	0	1	0	0	0	1	1	0	0	0	1	0	0	0	1
seedBP7.seedMinPu0.001	17	0	0	0	1	1	1	0	0	1	0	0	0	0	0	0	0	0	0	1	0	0	1	1	0	1	0	0	1	0	1	1	1	0	1	0	0	0	1	1	0	0	0	1	0	0	0	1
seedBP7.seedMinPu0.01	19	0	0	0	1	1	1	0	0	1	0	1	0	0	0	0	0	0	0	1	0	1	1	1	0	1	0	0	1	0	1	1	1	0	1	0	0	0	1	1	0	0	0	1	0	0	0	1
seedBP7.seedMinPu0.1	15	0	0	0	1	1	0	0	0	1	0	1	0	0	0	0	1	0	0	1	0	1	0	0	0	1	0	1	1	0	1	1	1	0	0	0	0	0	1	1	0	0	0	0	0	0	0	0
seedBP7.seedMinPu0.0001	16	0	0	0	1	1	1	0	0	1	0	0	0	0	0	0	0	0	0	1	0	0	1	1	0	1	0	0	1	0	1	0	1	0	1	0	0	0	1	1	0	0	0	1	0	0	0	1
seedBP7.seedMinPu0.0001.seedNoGU	17	0	0	0	1	1	1	0	0	1	0	1	0	0	0	0	1	0	0	1	0	1	1	1	0	1	0	0	1	0	1	0	0	0	1	0	0	0	1	0	0	0	0	1	0	0	0	1
seedBP7.seedMinPu0.001.seedNoGU	18	0	0	0	1	1	1	0	0	1	0	1	0	0	0	0	1	0	0	1	0	1	1	1	0	1	0	1	1	0	1	0	0	0	1	0	0	0	1	0	0	0	0	1	0	0	0	1
seedBP7.seedMinPu0.1.seedNoGU	14	0	0	0	1	1	0	1	0	0	1	1	0	0	0	0	1	0	0	1	1	0	1	0	0	1	0	0	1	0	1	0	0	1	0	0	0	0	1	0	0	0	0	0	0	0	0	0
seedBP7.seedMinPu0.01.seedNoGU	17	0	0	0	1	1	1	0	0	1	0	1	0	0	0	0	1	0	0	1	0	0	1	1	0	1	0	1	1	0	1	0	0	0	1	0	0	0	1	0	0	0	0	1	0	0	0	1
seedBP7.seedMinPu0.2	4	0	0	0	0	0	0	0	0	0	0	0	0	0	0	0	0	0	0	0	0	0	0	0	0	0	0	0	0	0	1	0	1	0	0	0	0	0	1	1	0	0	0	0	0	0	0	0
seedBP7.seedMinPu0.2.seedNoGU	2	0	0	0	0	0	0	0	0	0	0	0	0	0	0	0	0	0	0	0	0	0	0	0	0	0	0	0	0	0	1	0	0	0	0	0	0	0	1	0	0	0	0	0	0	0	0	0
seedBP7.seedMinPu0.5	0	0	0	0	0	0	0	0	0	0	0	0	0	0	0	0	0	0	0	0	0	0	0	0	0	0	0	0	0	0	0	0	0	0	0	0	0	0	0	0	0	0	0	0	0	0	0	0
seedBP7.seedMinPu0.5.seedNoGU	0	0	0	0	0	0	0	0	0	0	0	0	0	0	0	0	0	0	0	0	0	0	0	0	0	0	0	0	0	0	0	0	0	0	0	0	0	0	0	0	0	0	0	0	0	0	0	0
seedBP7.seedMinPu0.seedNoGU	17	0	0	0	1	1	1	0	0	1	0	1	0	0	0	0	1	0	0	1	0	1	1	1	0	1	0	0	1	0	1	0	0	0	1	0	0	0	1	0	0	0	0	1	0	0	0	1
seedBP7.seedNoGU	17	0	0	0	1	1	1	0	0	1	0	1	0	0	0	0	1	0	0	1	0	1	1	1	0	1	0	0	1	0	1	0	0	0	1	0	0	0	1	0	0	0	0	1	0	0	0	1
seedBP8	16	0	0	0	1	1	1	0	0	1	0	0	0	0	0	0	0	0	0	1	0	0	1	1	0	1	0	0	1	0	1	1	1	0	0	0	0	0	1	1	0	0	0	1	0	0	0	1
seedBP8.seedMinPu0	16	0	0	0	1	1	1	0	0	1	0	0	0	0	0	0	0	0	0	1	0	0	1	1	0	1	0	0	1	0	1	1	1	0	0	0	0	0	1	1	0	0	0	1	0	0	0	1
seedBP8.seedMinPu0.1	12	0	0	0	0	0	0	1	0	0	1	0	0	0	0	0	0	0	0	1	0	1	0	0	0	1	0	1	1	0	1	0	1	1	0	0	0	0	1	1	0	0	0	0	0	0	0	0
seedBP8.seedMinPu0.0001	16	0	0	0	1	1	1	0	0	1	0	0	0	0	0	0	0	0	0	1	0	0	1	1	0	1	0	0	1	0	1	1	1	0	0	0	0	0	1	1	0	0	0	1	0	0	0	1
seedBP8.seedMinPu0.001	15	0	0	0	1	1	0	0	0	1	0	0	0	0	0	0	0	0	0	1	0	0	1	1	0	1	0	0	1	0	1	1	1	0	0	0	0	0	1	1	0	0	0	1	0	0	0	1
seedBP8.seedMinPu0.01	16	0	0	0	1	1	0	0	0	1	0	0	0	0	0	0	0	0	0	1	0	1	1	1	0	1	0	0	1	0	1	1	1	0	0	0	0	0	1	1	0	0	0	1	0	0	0	1
seedBP8.seedMinPu0.001.seedNoGU	17	0	0	0	1	1	0	1	0	1	0	1	0	0	0	0	1	0	0	1	0	1	1	1	0	1	1	0	1	0	0	0	0	1	0	0	0	0	1	0	0	0	0	1	0	0	0	1
seedBP8.seedMinPu0.0001.seedNoGU	16	0	0	0	1	1	1	0	0	1	0	1	0	0	0	0	1	0	0	1	0	1	1	1	0	1	0	0	1	1	0	0	0	0	0	0	0	0	1	0	0	0	0	1	0	0	0	1
seedBP8.seedMinPu0.1.seedNoGU	8	0	0	0	0	0	0	1	1	0	1	0	0	0	0	0	0	0	0	1	0	0	0	0	0	1	0	0	1	0	0	0	0	1	0	0	0	0	1	0	0	0	0	0	0	0	0	0
seedBP8.seedMinPu0.01.seedNoGU	11	0	0	0	1	1	0	1	0	0	0	0	0	0	0	0	0	0	0	1	0	0	1	0	0	1	0	0	1	0	0	0	0	1	0	0	0	0	1	0	0	0	0	1	0	0	0	1
seedBP8.seedMinPu0.2	2	0	0	0	0	0	0	0	0	0	0	0	0	0	0	0	0	0	0	0	0	0	0	0	0	0	0	0	0	0	1	0	0	0	0	0	0	0	1	0	0	0	0	0	0	0	0	0
seedBP8.seedMinPu0.2.seedNoGU	1	0	0	0	0	0	0	0	0	0	0	0	0	0	0	0	0	0	0	0	0	0	0	0	0	0	0	0	0	0	0	0	0	0	0	0	0	0	1	0	0	0	0	0	0	0	0	0
seedBP8.seedMinPu0.5	0	0	0	0	0	0	0	0	0	0	0	0	0	0	0	0	0	0	0	0	0	0	0	0	0	0	0	0	0	0	0	0	0	0	0	0	0	0	0	0	0	0	0	0	0	0	0	0
seedBP8.seedMinPu0.5.seedNoGU	0	0	0	0	0	0	0	0	0	0	0	0	0	0	0	0	0	0	0	0	0	0	0	0	0	0	0	0	0	0	0	0	0	0	0	0	0	0	0	0	0	0	0	0	0	0	0	0
seedBP8.seedMinPu0.seedNoGU	16	0	0	0	1	1	1	0	0	1	0	1	0	0	0	0	1	0	0	1	0	1	1	1	0	1	0	0	1	1	0	0	0	0	0	0	0	0	1	0	0	0	0	1	0	0	0	1
seedBP8.seedNoGU	16	0	0	0	1	1	1	0	0	1	0	1	0	0	0	0	1	0	0	1	0	1	1	1	0	1	0	0	1	1	0	0	0	0	0	0	0	0	1	0	0	0	0	1	0	0	0	1
seedBP9	17	0	0	0	1	1	1	0	0	1	0	1	0	0	0	0	0	0	0	1	0	1	1	1	0	1	0	0	1	0	1	1	1	0	0	0	0	0	1	1	0	0	0	1	0	0	0	0
seedBP9.seedNoGU	15	0	0	0	1	1	1	1	0	1	0	1	0	0	0	0	1	0	0	1	0	0	0	1	0	1	1	0	1	0	0	0	0	1	0	0	0	0	1	0	0	0	0	1	0	0	0	0
seedBP10	15	0	0	0	1	1	0	0	0	1	0	1	0	0	0	0	1	0	0	0	0	1	1	1	0	1	0	0	1	0	1	0	1	0	0	0	0	0	1	1	0	0	0	1	0	0	0	0
seedBP10.seedNoGU	13	0	0	0	1	1	0	1	0	1	1	1	0	0	0	0	1	0	1	0	0	0	0	0	1	1	1	0	1	0	0	0	0	0	0	0	0	0	0	0	0	0	0	1	0	0	0	0
seedBP11	13	0	0	0	1	1	0	0	0	1	0	1	0	0	0	0	1	0	0	1	0	1	1	1	0	1	0	0	0	0	1	0	0	0	0	0	0	0	1	1	0	0	0	0	0	0	0	0

Salmonella typhimurium
NC_003197

	sum	ArcZ-STM1682	ArcZ-STM2970	ArcZ-STM3216	ChiX-STM0687	ChiX-STM1313	CyaR-STM0833	GcvB-STM0002	GcvB-STM0245	GcvB-STM0399	GcvB-STM0602	GcvB-STM0665	GcvB-STM0959	GcvB-STM1299	GcvB-STM1452	GcvB-STM1746	GcvB-STM2355	GcvB-STM2526	GcvB-STM3062	GcvB-STM3064	GcvB-STM3225	GcvB-STM3564	GcvB-STM3567	GcvB-STM3630	GcvB-STM3903	GcvB-STM3909	GcvB-STM3930	GcvB-STM4351	GcvB-STM4398	MicA-STM4231	MicC-STM1572	MicF-STM0366	MicF-STM0959	MicF-STM1328	RybB-STM0413	RybB-STM0687	RybB-STM0999	RybB-STM1070	RybB-STM1473	RybB-STM1530	RybB-STM1572	RybB-STM1732	RybB-STM1995	RybB-STM2267	RybB-STM2391	SgrS-STM2945	SgrS-STM3962	Spot42-STM2190
Target found among top-100 predictions for the sRNA for any tested parameterization	40	1	0	0	1	1	1	1	1	1	1	1	1	1	0	0	1	1	1	1	1	1	1	1	1	1	1	1	1	1	1	1	1	1	1	1	1	1	1	1	1	0	0	1	1	0	1	1
seedBP11.seedNoGU	6	0	0	0	1	1	0	0	0	0	1	1	0	0	0	0	0	0	1	0	0	0	0	0	0	1	0	0	0	0	0	0	0	0	0	0	0	0	0	0	0	0	0	0	0	0	0	0
seedBP12	10	0	0	0	1	1	0	0	0	0	0	1	0	0	0	0	0	0	0	1	0	1	0	1	0	1	0	0	0	0	1	0	0	0	0	0	0	0	0	1	0	0	0	0	0	0	1	0
seedBP12.seedNoGU	5	0	0	0	1	1	0	0	0	0	1	1	0	0	0	0	0	0	0	0	0	0	0	0	0	1	0	0	0	0	0	0	0	0	0	0	0	0	0	0	0	0	0	0	0	0	0	0
seedBP13	5	0	0	0	0	0	0	0	0	0	0	1	0	0	0	0	0	0	0	0	0	1	0	0	0	1	1	0	0	0	0	0	0	0	0	1	0	0	0	0	0	0	0	0	0	0	0	0
seedBP13.seedNoGU	0	0	0	0	0	0	0	0	0	0	0	0	0	0	0	0	0	0	0	0	0	0	0	0	0	0	0	0	0	0	0	0	0	0	0	0	0	0	0	0	0	0	0	0	0	0	0	0
seedBP14	5	0	0	0	0	0	0	0	0	0	0	0	0	1	0	0	0	0	0	0	0	1	0	0	0	1	1	0	0	0	0	0	0	0	0	0	0	0	0	0	0	0	0	0	1	0	0	0
seedBP14.seedNoGU	0	0	0	0	0	0	0	0	0	0	0	0	0	0	0	0	0	0	0	0	0	0	0	0	0	0	0	0	0	0	0	0	0	0	0	0	0	0	0	0	0	0	0	0	0	0	0	0
seedMaxE0	16	0	0	0	1	1	1	0	0	1	0	0	0	0	0	0	0	0	0	1	0	0	1	1	0	1	0	0	1	0	1	0	1	0	1	0	0	0	1	1	0	0	0	1	0	0	0	1
seedMaxE0.seedMinPu0	16	0	0	0	1	1	1	0	0	1	0	0	0	0	0	0	0	0	0	1	0	0	1	1	0	1	0	0	1	0	1	0	1	0	1	0	0	0	1	1	0	0	0	1	0	0	0	1
seedMaxE0.seedMinPu0.01	19	0	0	0	1	1	1	0	0	1	0	1	0	0	0	0	0	0	0	1	0	1	1	1	0	1	0	0	1	0	1	1	1	0	1	0	0	0	1	1	0	0	0	1	0	0	0	1
seedMaxE0.seedMinPu0.001	17	0	0	0	1	1	1	0	0	1	0	0	0	0	0	0	0	0	0	1	0	0	1	1	0	1	0	0	1	0	1	1	1	0	1	0	0	0	1	1	0	0	0	1	0	0	0	1
seedMaxE0.seedMinPu0.0001	16	0	0	0	1	1	1	0	0	1	0	0	0	0	0	0	0	0	0	1	0	0	1	1	0	1	0	0	1	0	1	0	1	0	1	0	0	0	1	1	0	0	0	1	0	0	0	1
seedMaxE0.seedMinPu0.1	15	0	0	0	1	1	0	0	0	1	0	1	0	0	0	0	1	0	0	1	0	1	0	0	0	1	0	1	1	0	1	1	1	0	0	0	0	0	1	1	0	0	0	0	0	0	0	0
seedMaxE0.seedMinPu0.01.seedNoGU	17	0	0	0	1	1	1	0	0	1	0	1	0	0	0	0	1	0	0	1	0	0	1	1	0	1	0	1	1	0	1	0	0	0	1	0	0	0	1	0	0	0	0	1	0	0	0	1
seedMaxE0.seedMinPu0.001.seedNoGU	18	0	0	0	1	1	1	0	0	1	0	1	0	0	0	0	1	0	0	1	0	1	1	1	0	1	0	1	1	0	1	0	0	0	1	0	0	0	1	0	0	0	0	1	0	0	0	1
seedMaxE0.seedMinPu0.1.seedNoGU	14	0	0	0	1	1	0	1	0	0	1	1	0	0	0	0	1	0	0	1	1	0	1	0	0	1	0	0	1	0	1	0	0	1	0	0	0	0	1	0	0	0	0	0	0	0	0	0
seedMaxE0.seedMinPu0.0001.seedNoGU	17	0	0	0	1	1	1	0	0	1	0	1	0	0	0	0	1	0	0	1	0	1	1	1	0	1	0	0	1	0	1	0	0	0	1	0	0	0	1	0	0	0	0	1	0	0	0	1
seedMaxE0.seedMinPu0.2	4	0	0	0	0	0	0	0	0	0	0	0	0	0	0	0	0	0	0	0	0	0	0	0	0	0	0	0	0	0	1	0	1	0	0	0	0	0	1	1	0	0	0	0	0	0	0	0
seedMaxE0.seedMinPu0.2.seedNoGU	2	0	0	0	0	0	0	0	0	0	0	0	0	0	0	0	0	0	0	0	0	0	0	0	0	0	0	0	0	0	1	0	0	0	0	0	0	0	1	0	0	0	0	0	0	0	0	0
seedMaxE0.seedMinPu0.5	0	0	0	0	0	0	0	0	0	0	0	0	0	0	0	0	0	0	0	0	0	0	0	0	0	0	0	0	0	0	0	0	0	0	0	0	0	0	0	0	0	0	0	0	0	0	0	0
seedMaxE0.seedMinPu0.5.seedNoGU	0	0	0	0	0	0	0	0	0	0	0	0	0	0	0	0	0	0	0	0	0	0	0	0	0	0	0	0	0	0	0	0	0	0	0	0	0	0	0	0	0	0	0	0	0	0	0	0
seedMaxE0.seedMinPu0.seedNoGU	17	0	0	0	1	1	1	0	0	1	0	1	0	0	0	0	1	0	0	1	0	1	1	1	0	1	0	0	1	0	1	0	0	0	1	0	0	0	1	0	0	0	0	1	0	0	0	1
seedMaxE0.seedNoGU	17	0	0	0	1	1	1	0	0	1	0	1	0	0	0	0	1	0	0	1	0	1	1	1	0	1	0	0	1	0	1	0	0	0	1	0	0	0	1	0	0	0	0	1	0	0	0	1
seedMaxE-1	16	0	0	0	1	1	1	0	0	1	0	0	0	0	0	0	0	0	0	1	0	0	1	1	0	1	0	0	1	0	1	0	1	0	1	0	0	0	1	1	0	0	0	1	0	0	0	1
seedMaxE-1.seedMinPu0	16	0	0	0	1	1	1	0	0	1	0	0	0	0	0	0	0	0	0	1	0	0	1	1	0	1	0	0	1	0	1	0	1	0	1	0	0	0	1	1	0	0	0	1	0	0	0	1
seedMaxE-1.seedMinPu0.001	17	0	0	0	1	1	1	0	0	1	0	0	0	0	0	0	0	0	0	1	0	0	1	1	0	1	0	0	1	0	1	1	1	0	1	0	0	0	1	1	0	0	0	1	0	0	0	1
seedMaxE-1.seedMinPu0.0001	16	0	0	0	1	1	1	0	0	1	0	0	0	0	0	0	0	0	0	1	0	0	1	1	0	1	0	0	1	0	1	0	1	0	1	0	0	0	1	1	0	0	0	1	0	0	0	1
seedMaxE-1.seedMinPu0.01	19	0	0	0	1	1	1	0	0	1	0	1	0	0	0	0	0	0	0	1	0	1	1	1	0	1	0	0	1	0	1	1	1	0	1	0	0	0	1	1	0	0	0	1	0	0	0	1
seedMaxE-1.seedMinPu0.1	15	0	0	0	1	1	0	0	0	1	0	1	0	0	0	0	1	0	0	1	0	1	0	0	0	1	0	1	1	0	1	1	1	0	0	0	0	0	1	1	0	0	0	0	0	0	0	0
seedMaxE-1.seedMinPu0.1.seedNoGU	14	0	0	0	1	1	0	1	0	0	1	1	0	0	0	0	1	0	0	1	1	0	1	0	0	1	0	0	1	0	1	0	0	1	0	0	0	0	1	0	0	0	0	0	0	0	0	0
seedMaxE-1.seedMinPu0.0001.seedNoGU	17	0	0	0	1	1	1	0	0	1	0	1	0	0	0	0	1	0	0	1	0	1	1	1	0	1	0	0	1	0	1	0	0	0	1	0	0	0	1	0	0	0	0	1	0	0	0	1
seedMaxE-1.seedMinPu0.001.seedNoGU	18	0	0	0	1	1	1	0	0	1	0	1	0	0	0	0	1	0	0	1	0	1	1	1	0	1	0	1	1	0	1	0	0	0	1	0	0	0	1	0	0	0	0	1	0	0	0	1
seedMaxE-1.seedMinPu0.01.seedNoGU	17	0	0	0	1	1	1	0	0	1	0	1	0	0	0	0	1	0	0	1	0	0	1	1	0	1	0	1	1	0	1	0	0	0	1	0	0	0	1	0	0	0	0	1	0	0	0	1
seedMaxE-1.seedMinPu0.2	4	0	0	0	0	0	0	0	0	0	0	0	0	0	0	0	0	0	0	0	0	0	0	0	0	0	0	0	0	0	1	0	1	0	0	0	0	0	1	1	0	0	0	0	0	0	0	0
seedMaxE-1.seedMinPu0.2.seedNoGU	2	0	0	0	0	0	0	0	0	0	0	0	0	0	0	0	0	0	0	0	0	0	0	0	0	0	0	0	0	0	1	0	0	0	0	0	0	0	1	0	0	0	0	0	0	0	0	0
seedMaxE-1.seedMinPu0.5	0	0	0	0	0	0	0	0	0	0	0	0	0	0	0	0	0	0	0	0	0	0	0	0	0	0	0	0	0	0	0	0	0	0	0	0	0	0	0	0	0	0	0	0	0	0	0	0
seedMaxE-1.seedMinPu0.5.seedNoGU	0	0	0	0	0	0	0	0	0	0	0	0	0	0	0	0	0	0	0	0	0	0	0	0	0	0	0	0	0	0	0	0	0	0	0	0	0	0	0	0	0	0	0	0	0	0	0	0
seedMaxE-1.seedMinPu0.seedNoGU	17	0	0	0	1	1	1	0	0	1	0	1	0	0	0	0	1	0	0	1	0	1	1	1	0	1	0	0	1	0	1	0	0	0	1	0	0	0	1	0	0	0	0	1	0	0	0	1
seedMaxE-1.seedNoGU	17	0	0	0	1	1	1	0	0	1	0	1	0	0	0	0	1	0	0	1	0	1	1	1	0	1	0	0	1	0	1	0	0	0	1	0	0	0	1	0	0	0	0	1	0	0	0	1
seedMaxE-2	17	0	0	0	1	1	1	0	0	1	0	0	0	0	0	0	0	0	0	1	0	0	1	1	0	1	0	0	1	0	1	1	1	0	1	0	0	0	1	1	0	0	0	1	0	0	0	1
seedMaxE-2.seedMinPu0	17	0	0	0	1	1	1	0	0	1	0	0	0	0	0	0	0	0	0	1	0	0	1	1	0	1	0	0	1	0	1	1	1	0	1	0	0	0	1	1	0	0	0	1	0	0	0	1
seedMaxE-2.seedMinPu0.01	19	0	0	0	1	1	1	0	0	1	0	1	0	0	0	0	0	0	0	1	0	1	1	1	0	1	0	0	1	0	1	1	1	0	1	0	0	0	1	1	0	0	0	1	0	0	0	1
seedMaxE-2.seedMinPu0.1	16	0	0	0	1	1	0	0	0	1	0	1	0	1	0	0	1	0	0	1	0	1	0	0	0	1	0	1	1	0	1	1	1	0	0	0	0	0	1	1	0	0	0	0	0	0	0	0
seedMaxE-2.seedMinPu0.0001	17	0	0	0	1	1	1	0	0	1	0	0	0	0	0	0	0	0	0	1	0	0	1	1	0	1	0	0	1	0	1	1	1	0	1	0	0	0	1	1	0	0	0	1	0	0	0	1
seedMaxE-2.seedMinPu0.001	17	0	0	0	1	1	1	0	0	1	0	0	0	0	0	0	0	0	0	1	0	0	1	1	0	1	0	0	1	0	1	1	1	0	1	0	0	0	1	1	0	0	0	1	0	0	0	1
seedMaxE-2.seedMinPu0.0001.seedNoGU	18	0	0	0	1	1	1	0	0	1	0	1	0	0	0	0	1	0	0	1	0	1	1	1	0	1	0	1	1	0	1	0	0	0	1	0	0	0	1	0	0	0	0	1	0	0	0	1
seedMaxE-2.seedMinPu0.1.seedNoGU	14	0	0	0	1	1	0	1	0	0	1	1	0	0	0	0	1	0	0	1	1	0	1	0	0	1	0	0	1	0	1	0	0	1	0	0	0	0	1	0	0	0	0	0	0	0	0	0
seedMaxE-2.seedMinPu0.01.seedNoGU	17	0	0	0	1	1	1	0	0	1	0	1	0	0	0	0	1	0	0	1	0	0	1	1	0	1	0	1	1	0	1	0	0	0	1	0	0	0	1	0	0	0	0	1	0	0	0	1
seedMaxE-2.seedMinPu0.001.seedNoGU	18	0	0	0	1	1	1	0	0	1	0	1	0	0	0	0	1	0	0	1	0	1	1	1	0	1	0	1	1	0	1	0	0	0	1	0	0	0	1	0	0	0	0	1	0	0	0	1
seedMaxE-2.seedMinPu0.2	3	0	0	0	0	0	0	0	0	0	0	0	0	0	0	0	0	0	0	0	0	0	0	0	0	0	0	0	0	0	1	0	0	0	0	0	0	0	1	1	0	0	0	0	0	0	0	0
seedMaxE-2.seedMinPu0.2.seedNoGU	2	0	0	0	0	0	0	0	0	0	0	0	0	0	0	0	0	0	0	0	0	0	0	0	0	0	0	0	0	0	1	0	0	0	0	0	0	0	1	0	0	0	0	0	0	0	0	0
seedMaxE-2.seedMinPu0.5	0	0	0	0	0	0	0	0	0	0	0	0	0	0	0	0	0	0	0	0	0	0	0	0	0	0	0	0	0	0	0	0	0	0	0	0	0	0	0	0	0	0	0	0	0	0	0	0
seedMaxE-2.seedMinPu0.5.seedNoGU	0	0	0	0	0	0	0	0	0	0	0	0	0	0	0	0	0	0	0	0	0	0	0	0	0	0	0	0	0	0	0	0	0	0	0	0	0	0	0	0	0	0	0	0	0	0	0	0
seedMaxE-2.seedMinPu0.seedNoGU	18	0	0	0	1	1	1	0	0	1	0	1	0	0	0	0	1	0	0	1	0	1	1	1	0	1	0	1	1	0	1	0	0	0	1	0	0	0	1	0	0	0	0	1	0	0	0	1
seedMaxE-2.seedNoGU	18	0	0	0	1	1	1	0	0	1	0	1	0	0	0	0	1	0	0	1	0	1	1	1	0	1	0	1	1	0	1	0	0	0	1	0	0	0	1	0	0	0	0	1	0	0	0	1
seedMaxE-3	17	0	0	0	1	1	1	0	0	1	0	0	0	0	0	0	0	0	0	1	0	0	1	1	0	1	0	0	1	0	1	1	1	0	1	0	0	0	1	1	0	0	0	1	0	0	0	1

Salmonella typhimurium *NC_003197*

	sum	ArcZ-STM1682	ArcZ-STM2970	ArcZ-STM3216	ChiX-STM0687	ChiX-STM1313	CyaR-STM0833	GcvB-STM0002	GcvB-STM0245	GcvB-STM0399	GcvB-STM0602	GcvB-STM0665	GcvB-STM0959	GcvB-STM1299	GcvB-STM1452	GcvB-STM1746	GcvB-STM2355	GcvB-STM2526	GcvB-STM3062	GcvB-STM3064	GcvB-STM3225	GcvB-STM3564	GcvB-STM3567	GcvB-STM3630	GcvB-STM3903	GcvB-STM3909	GcvB-STM3930	GcvB-STM4351	GcvB-STM4398	MicA-STM4231	MicC-STM1572	MicF-STM0366	MicF-STM0959	MicF-STM1328	RybB-STM0413	RybB-STM0687	RybB-STM0999	RybB-STM1070	RybB-STM1473	RybB-STM1530	RybB-STM1572	RybB-STM1732	RybB-STM1995	RybB-STM2267	RybB-STM2391	SgrS-STM2945	SgrS-STM3962	Spot42-STM2190
Target found among top-100 predictions for the sRNA for any tested parameterization	40	1	0	0	1	1	1	1	1	1	1	1	1	1	0	0	1	1	1	1	1	1	1	1	1	1	1	1	1	1	1	1	1	1	1	1	1	1	1	1	1	0	0	1	1	0	1	1
seedMaxE-3.seedMinPu0	17	0	0	0	1	1	1	0	0	1	0	0	0	0	0	0	0	0	0	1	0	0	1	1	0	1	0	0	1	0	1	1	1	0	1	0	0	0	1	1	0	0	0	1	0	0	0	1
seedMaxE-3.seedMinPu0.001	19	0	0	0	1	1	1	0	0	1	0	1	0	0	0	0	0	0	0	1	0	1	1	1	0	1	0	0	1	0	1	1	1	0	1	0	0	0	1	1	0	0	0	1	0	0	0	1
seedMaxE-3.seedMinPu0.0001	17	0	0	0	1	1	1	0	0	1	0	0	0	0	0	0	0	0	0	1	0	0	1	1	0	1	0	0	1	0	1	1	1	0	1	0	0	0	1	1	0	0	0	1	0	0	0	1
seedMaxE-3.seedMinPu0.1	17	0	0	0	1	1	0	0	0	1	0	1	0	1	0	0	1	0	0	0	0	1	1	0	0	1	1	1	1	0	1	1	1	0	0	0	0	0	1	1	0	0	0	0	0	0	0	0
seedMaxE-3.seedMinPu0.01	19	0	0	0	1	1	1	0	0	1	0	1	0	0	0	0	0	0	0	1	0	1	1	1	0	1	0	0	1	0	1	1	1	0	1	0	0	0	1	1	0	0	0	1	0	0	0	1
seedMaxE-3.seedMinPu0.001.seedNoGU	18	0	0	0	1	1	1	0	0	1	0	1	0	0	0	0	1	0	0	1	0	1	1	1	0	1	0	1	1	0	1	0	0	0	1	0	0	0	1	0	0	0	0	1	0	0	0	1
seedMaxE-3.seedMinPu0.01.seedNoGU	17	0	0	0	1	1	1	0	0	0	0	1	0	0	0	0	1	0	0	1	0	0	1	1	0	1	0	1	1	0	1	0	0	1	1	0	0	0	1	0	0	0	0	1	0	0	0	1
seedMaxE-3.seedMinPu0.1.seedNoGU	14	0	0	0	1	1	0	1	0	0	1	1	0	0	0	0	1	0	0	1	1	0	1	0	0	1	0	0	1	0	1	0	0	1	0	0	0	0	1	0	0	0	0	0	0	0	0	0
seedMaxE-3.seedMinPu0.0001.seedNoGU	18	0	0	0	1	1	1	0	0	1	0	1	0	0	0	0	1	0	0	1	0	1	1	1	0	1	0	1	1	0	1	0	0	0	1	0	0	0	1	0	0	0	0	1	0	0	0	1
seedMaxE-3.seedMinPu0.2	3	0	0	0	0	0	0	0	0	0	0	0	0	0	0	0	0	0	0	0	0	0	0	0	0	0	0	0	0	0	1	0	0	0	0	0	0	0	1	1	0	0	0	0	0	0	0	0
seedMaxE-3.seedMinPu0.2.seedNoGU	2	0	0	0	0	0	0	0	0	0	0	0	0	0	0	0	0	0	0	0	0	0	0	0	0	0	0	0	0	0	1	0	0	0	0	0	0	0	1	0	0	0	0	0	0	0	0	0
seedMaxE-3.seedMinPu0.5	0	0	0	0	0	0	0	0	0	0	0	0	0	0	0	0	0	0	0	0	0	0	0	0	0	0	0	0	0	0	0	0	0	0	0	0	0	0	0	0	0	0	0	0	0	0	0	0
seedMaxE-3.seedMinPu0.5.seedNoGU	0	0	0	0	0	0	0	0	0	0	0	0	0	0	0	0	0	0	0	0	0	0	0	0	0	0	0	0	0	0	0	0	0	0	0	0	0	0	0	0	0	0	0	0	0	0	0	0
seedMaxE-3.seedMinPu0.seedNoGU	18	0	0	0	1	1	1	0	0	1	0	1	0	0	0	0	1	0	0	1	0	1	1	1	0	1	0	1	1	0	1	0	0	0	1	0	0	0	1	0	0	0	0	1	0	0	0	1
seedMaxE-3.seedNoGU	18	0	0	0	1	1	1	0	0	1	0	1	0	0	0	0	1	0	0	1	0	1	1	1	0	1	0	1	1	0	1	0	0	0	1	0	0	0	1	0	0	0	0	1	0	0	0	1
seedMaxE-4	19	0	0	0	1	1	1	0	0	1	0	1	0	0	0	0	0	0	0	1	0	1	1	1	0	1	0	0	1	0	1	1	1	0	1	0	0	0	1	1	0	0	0	1	0	0	0	1
seedMaxE-4.seedMinPu0	19	0	0	0	1	1	1	0	0	1	0	1	0	0	0	0	0	0	0	1	0	1	1	1	0	1	0	0	1	0	1	1	1	0	1	0	0	0	1	1	0	0	0	1	0	0	0	1
seedMaxE-4.seedMinPu0.01	17	0	0	0	1	1	1	0	0	0	0	0	0	0	0	0	1	0	0	1	0	0	1	1	0	1	0	0	1	0	1	1	1	0	1	0	0	0	1	1	0	0	0	1	0	0	0	1
seedMaxE-4.seedMinPu0.001	19	0	0	0	1	1	1	0	0	1	0	1	0	0	0	0	0	0	0	1	0	1	1	1	0	1	0	0	1	0	1	1	1	0	1	0	0	0	1	1	0	0	0	1	0	0	0	1
seedMaxE-4.seedMinPu0.1	15	0	0	0	1	1	0	1	0	0	0	1	0	1	0	0	0	0	0	0	0	0	1	0	0	1	0	1	1	0	1	1	1	1	0	0	0	0	1	1	0	0	0	0	0	0	0	0
seedMaxE-4.seedMinPu0.0001	19	0	0	0	1	1	1	0	0	1	0	1	0	0	0	0	0	0	0	1	0	1	1	1	0	1	0	0	1	0	1	1	1	0	1	0	0	0	1	1	0	0	0	1	0	0	0	1
seedMaxE-4.seedMinPu0.0001.seedNoGU	18	0	0	0	1	1	1	0	0	1	0	1	0	0	0	0	0	0	0	1	0	1	1	1	0	1	0	1	1	0	1	0	0	1	1	0	0	0	1	0	0	0	0	1	0	0	0	1
seedMaxE-4.seedMinPu0.1.seedNoGU	14	0	0	0	1	1	0	1	0	0	1	1	0	0	0	0	0	0	0	1	1	0	1	0	1	1	0	0	1	0	1	0	0	1	0	0	0	0	1	0	0	0	0	0	0	0	0	0
seedMaxE-4.seedMinPu0.001.seedNoGU	18	0	0	0	1	1	1	0	0	1	0	1	0	0	0	0	0	0	0	1	0	1	1	1	0	1	0	1	1	0	1	0	0	1	1	0	0	0	1	0	0	0	0	1	0	0	0	1
seedMaxE-4.seedMinPu0.01.seedNoGU	14	0	0	0	1	1	1	0	0	0	0	0	0	0	0	0	0	0	0	1	0	0	1	0	0	1	0	1	1	0	1	0	0	1	1	0	0	0	1	0	0	0	0	1	0	0	0	1
seedMaxE-4.seedMinPu0.2	2	0	0	0	0	0	0	0	0	0	0	0	0	0	0	0	0	0	0	0	0	0	0	0	0	0	0	0	0	0	1	0	0	0	0	0	0	0	1	0	0	0	0	0	0	0	0	0
seedMaxE-4.seedMinPu0.2.seedNoGU	2	0	0	0	0	0	0	0	0	0	0	0	0	0	0	0	0	0	0	0	0	0	0	0	0	0	0	0	0	0	1	0	0	0	0	0	0	0	1	0	0	0	0	0	0	0	0	0
seedMaxE-4.seedMinPu0.5	0	0	0	0	0	0	0	0	0	0	0	0	0	0	0	0	0	0	0	0	0	0	0	0	0	0	0	0	0	0	0	0	0	0	0	0	0	0	0	0	0	0	0	0	0	0	0	0
seedMaxE-4.seedMinPu0.5.seedNoGU	0	0	0	0	0	0	0	0	0	0	0	0	0	0	0	0	0	0	0	0	0	0	0	0	0	0	0	0	0	0	0	0	0	0	0	0	0	0	0	0	0	0	0	0	0	0	0	0
seedMaxE-4.seedMinPu0.seedNoGU	18	0	0	0	1	1	1	0	0	1	0	1	0	0	0	0	0	0	0	1	0	1	1	1	0	1	0	1	1	0	1	0	0	1	1	0	0	0	1	0	0	0	0	1	0	0	0	1
seedMaxE-4.seedNoGU	18	0	0	0	1	1	1	0	0	1	0	1	0	0	0	0	0	0	0	1	0	1	1	1	0	1	0	1	1	0	1	0	0	1	1	0	0	0	1	0	0	0	0	1	0	0	0	1
seedMaxE-5	19	0	0	0	1	1	1	0	0	1	0	1	0	0	0	0	0	0	0	1	0	1	1	1	0	1	0	0	1	0	1	1	1	0	1	0	0	0	1	1	0	0	0	1	0	0	0	1
seedMaxE-5.seedMinPu0	19	0	0	0	1	1	1	0	0	1	0	1	0	0	0	0	0	0	0	1	0	1	1	1	0	1	0	0	1	0	1	1	1	0	1	0	0	0	1	1	0	0	0	1	0	0	0	1
seedMaxE-5.seedMinPu0.1	13	0	0	0	0	1	0	1	0	0	1	1	0	0	0	0	0	0	0	1	0	0	1	0	0	1	0	0	1	0	1	0	1	1	0	0	0	0	1	1	0	0	0	0	0	0	0	0
seedMaxE-5.seedMinPu0.0001	19	0	0	0	1	1	1	0	0	1	0	1	0	0	0	0	0	0	0	1	0	1	1	1	0	1	0	0	1	0	1	1	1	0	1	0	0	0	1	1	0	0	0	1	0	0	0	1
seedMaxE-5.seedMinPu0.001	19	0	0	0	1	1	1	0	0	1	0	1	0	0	0	0	0	0	0	1	0	1	1	1	0	1	0	0	1	0	1	1	1	0	1	0	0	0	1	1	0	0	0	1	0	0	0	1
seedMaxE-5.seedMinPu0.01	17	0	0	0	1	1	1	0	0	0	0	1	0	0	0	0	0	0	0	1	0	0	1	0	0	1	0	1	1	0	1	1	1	0	1	0	0	0	1	1	0	0	0	1	0	0	0	1
seedMaxE-5.seedMinPu0.0001.seedNoGU	16	0	0	0	1	1	1	0	0	1	0	1	0	0	0	0	0	0	0	1	0	1	1	1	0	1	0	0	1	0	0	0	0	1	1	0	0	0	1	0	0	0	0	1	0	0	0	1
seedMaxE-5.seedMinPu0.01.seedNoGU	13	0	0	0	1	1	1	1	0	0	0	0	0	0	0	0	0	0	0	1	0	0	1	0	0	1	0	0	1	0	0	0	0	1	1	0	0	0	1	0	0	0	0	1	0	0	0	1
seedMaxE-5.seedMinPu0.1.seedNoGU	12	0	0	0	0	1	0	1	0	0	1	0	0	0	0	0	1	0	1	1	0	0	1	0	1	1	0	0	1	0	0	0	0	1	0	0	0	0	1	0	0	0	0	0	0	0	0	0
seedMaxE-5.seedMinPu0.001.seedNoGU	16	0	0	0	1	1	1	0	0	1	0	1	0	0	0	0	0	0	0	1	0	1	1	1	0	1	0	0	1	0	0	0	0	1	1	0	0	0	1	0	0	0	0	1	0	0	0	1
seedMaxE-5.seedMinPu0.2	2	0	0	0	0	0	0	0	0	0	0	0	0	0	0	0	0	0	0	0	0	0	0	0	0	0	0	0	0	0	1	0	0	0	0	0	0	0	1	0	0	0	0	0	0	0	0	0
seedMaxE-5.seedMinPu0.2.seedNoGU	1	0	0	0	0	0	0	0	0	0	0	0	0	0	0	0	0	0	0	0	0	0	0	0	0	0	0	0	0	0	0	0	0	0	0	0	0	0	1	0	0	0	0	0	0	0	0	0
seedMaxE-5.seedMinPu0.5	0	0	0	0	0	0	0	0	0	0	0	0	0	0	0	0	0	0	0	0	0	0	0	0	0	0	0	0	0	0	0	0	0	0	0	0	0	0	0	0	0	0	0	0	0	0	0	0
seedMaxE-5.seedMinPu0.5.seedNoGU	0	0	0	0	0	0	0	0	0	0	0	0	0	0	0	0	0	0	0	0	0	0	0	0	0	0	0	0	0	0	0	0	0	0	0	0	0	0	0	0	0	0	0	0	0	0	0	0
seedMaxE-5.seedMinPu0.seedNoGU	16	0	0	0	1	1	1	0	0	1	0	1	0	0	0	0	0	0	0	1	0	1	1	1	0	1	0	0	1	0	0	0	0	1	1	0	0	0	1	0	0	0	0	1	0	0	0	1
seedMaxE-5.seedNoGU	16	0	0	0	1	1	1	0	0	1	0	1	0	0	0	0	0	0	0	1	0	1	1	1	0	1	0	0	1	0	0	0	0	1	1	0	0	0	1	0	0	0	0	1	0	0	0	1
seedMaxE-6	14	0	0	0	1	1	1	0	0	0	0	0	0	0	0	0	0	0	0	1	0	0	0	0	0	1	0	0	1	0	0	1	1	1	1	0	0	0	1	1	0	0	0	1	0	0	0	1
seedMaxE-6.seedMinPu0	14	0	0	0	1	1	1	0	0	0	0	0	0	0	0	0	0	0	0	1	0	0	0	0	0	1	0	0	1	0	0	1	1	1	1	0	0	0	1	1	0	0	0	1	0	0	0	1
seedMaxE-6.seedMinPu0.01	14	0	0	0	1	1	1	0	0	0	0	0	0	0	0	0	0	0	0	1	0	0	0	0	0	1	0	0	1	0	0	1	1	1	1	0	0	0	1	1	0	0	0	1	0	0	0	1
seedMaxE-6.seedMinPu0.0001	14	0	0	0	1	1	1	0	0	0	0	0	0	0	0	0	0	0	0	1	0	0	0	0	0	1	0	0	1	0	0	1	1	1	1	0	0	0	1	1	0	0	0	1	0	0	0	1
seedMaxE-6.seedMinPu0.1	12	0	0	0	0	1	0	1	0	0	1	0	1	0	0	0	0	0	0	1	0	0	0	1	0	1	0	0	1	0	0	0	1	1	0	0	0	0	1	1	0	0	0	0	0	0	0	0
seedMaxE-6.seedMinPu0.001	14	0	0	0	1	1	1	0	0	0	0	0	0	0	0	0	0	0	0	1	0	0	0	0	0	1	0	0	1	0	0	1	1	1	1	0	0	0	1	1	0	0	0	1	0	0	0	1
seedMaxE-6.seedMinPu0.001.seedNoGU	11	0	0	0	1	1	1	1	0	0	0	0	0	0	0	0	0	0	0	1	0	0	0	0	0	1	0	0	1	0	0	0	0	1	1	0	0	0	0	0	0	0	0	1	0	0	0	1
seedMaxE-6.seedMinPu0.1.seedNoGU	10	0	0	0	0	1	0	1	0	0	1	0	0	0	0	0	1	0	1	1	0	0	0	0	1	1	0	0	1	0	0	0	0	1	0	0	0	0	0	0	0	0	0	0	0	0	0	0
seedMaxE-6.seedMinPu0.01.seedNoGU	11	0	0	0	1	1	1	1	0	0	0	0	0	0	0	0	0	0	0	1	0	0	0	0	0	1	0	0	1	0	0	0	0	1	1	0	0	0	0	0	0	0	0	1	0	0	0	1

Salmonella typhimurium *NC_003197*

	sum	ArcZ-STM1682	ArcZ-STM2970	ArcZ-STM3216	ChiX-STM0687	ChiX-STM1313	CyaR-STM0833	GcvB-STM0002	GcvB-STM0245	GcvB-STM0399	GcvB-STM0602	GcvB-STM0665	GcvB-STM0959	GcvB-STM1299	GcvB-STM1452	GcvB-STM1746	GcvB-STM2355	GcvB-STM2526	GcvB-STM3062	GcvB-STM3064	GcvB-STM3225	GcvB-STM3564	GcvB-STM3567	GcvB-STM3630	GcvB-STM3903	GcvB-STM3909	GcvB-STM3930	GcvB-STM4351	GcvB-STM4398	MicA-STM4231	MicC-STM1572	MicF-STM0366	MicF-STM0959	MicF-STM1328	RybB-STM0413	RybB-STM0687	RybB-STM0999	RybB-STM1070	RybB-STM1473	RybB-STM1530	RybB-STM1572	RybB-STM1732	RybB-STM1995	RybB-STM2267	RybB-STM2391	SgrS-STM2945	SgrS-STM3962	Spot42-STM2190
Target found among top-100 predictions for the sRNA for any tested parameterization	40	1	0	0	1	1	1	1	1	1	1	1	1	1	0	0	1	1	1	1	1	1	1	1	1	1	1	1	1	1	1	1	1	1	1	1	1	1	1	1	1	0	0	1	1	0	1	1
seedMaxE-6.seedMinPu0.0001.seedNoGU	11	0	0	0	1	1	1	1	0	0	0	0	0	0	0	0	0	0	0	1	0	0	0	0	0	1	0	0	1	0	0	0	0	1	1	0	0	0	0	0	0	0	0	1	0	0	0	1
seedMaxE-6.seedMinPu0.2	1	0	0	0	0	0	0	0	0	0	0	0	0	0	0	0	0	0	0	0	0	0	0	0	0	0	0	0	0	0	0	0	0	0	0	0	0	0	1	0	0	0	0	0	0	0	0	0
seedMaxE-6.seedMinPu0.2.seedNoGU	0	0	0	0	0	0	0	0	0	0	0	0	0	0	0	0	0	0	0	0	0	0	0	0	0	0	0	0	0	0	0	0	0	0	0	0	0	0	0	0	0	0	0	0	0	0	0	0
seedMaxE-6.seedMinPu0.5	0	0	0	0	0	0	0	0	0	0	0	0	0	0	0	0	0	0	0	0	0	0	0	0	0	0	0	0	0	0	0	0	0	0	0	0	0	0	0	0	0	0	0	0	0	0	0	0
seedMaxE-6.seedMinPu0.5.seedNoGU	0	0	0	0	0	0	0	0	0	0	0	0	0	0	0	0	0	0	0	0	0	0	0	0	0	0	0	0	0	0	0	0	0	0	0	0	0	0	0	0	0	0	0	0	0	0	0	0
seedMaxE-6.seedMinPu0.seedNoGU	11	0	0	0	1	1	1	1	0	0	0	0	0	0	0	0	0	0	0	1	0	0	0	0	0	1	0	0	1	0	0	0	0	1	1	0	0	0	0	0	0	0	0	1	0	0	0	1
seedMaxE-6.seedNoGU	11	0	0	0	1	1	1	1	0	0	0	0	0	0	0	0	0	0	0	1	0	0	0	0	0	1	0	0	1	0	0	0	0	1	1	0	0	0	0	0	0	0	0	1	0	0	0	1
seedMaxE-7	9	0	0	0	0	0	0	1	0	0	0	0	0	0	0	0	0	0	0	0	0	0	0	0	0	1	0	0	1	0	0	0	1	1	1	0	0	0	0	1	0	0	0	1	0	0	0	1
seedMaxE-7.seedMinPu0	9	0	0	0	0	0	0	1	0	0	0	0	0	0	0	0	0	0	0	0	0	0	0	0	0	1	0	0	1	0	0	0	1	1	1	0	0	0	0	1	0	0	0	1	0	0	0	1
seedMaxE-7.seedMinPu0.01	8	0	0	0	0	0	0	1	0	0	0	0	0	0	0	0	0	0	0	0	0	0	0	0	0	1	0	0	1	0	0	0	1	1	1	0	0	0	0	1	0	0	0	0	0	0	0	1
seedMaxE-7.seedMinPu0.001	9	0	0	0	0	0	0	1	0	0	0	0	0	0	0	0	0	0	0	0	0	0	0	0	0	1	0	0	1	0	0	0	1	1	1	0	0	0	0	1	0	0	0	1	0	0	0	1
seedMaxE-7.seedMinPu0.0001	9	0	0	0	0	0	0	1	0	0	0	0	0	0	0	0	0	0	0	0	0	0	0	0	0	1	0	0	1	0	0	0	1	1	1	0	0	0	0	1	0	0	0	1	0	0	0	1
seedMaxE-7.seedMinPu0.1	9	0	0	0	0	0	0	1	0	0	1	0	1	0	0	0	0	0	0	0	0	0	0	1	0	1	0	0	1	0	0	0	1	1	0	0	0	0	0	1	0	0	0	0	0	0	0	0
seedMaxE-7.seedMinPu0.1.seedNoGU	7	0	0	0	0	0	0	1	0	0	1	0	0	0	0	0	0	0	1	0	0	0	1	0	0	1	0	0	1	0	0	0	0	1	0	0	0	0	0	0	0	0	0	0	0	0	0	0
seedMaxE-7.seedMinPu0.001.seedNoGU	8	0	0	0	0	0	0	1	0	0	1	0	0	0	0	0	0	0	0	0	0	0	0	0	0	1	0	0	1	0	0	0	0	1	1	0	0	0	0	0	0	0	0	1	0	0	0	1
seedMaxE-7.seedMinPu0.01.seedNoGU	7	0	0	0	0	0	0	1	0	0	1	0	0	0	0	0	0	0	0	0	0	0	0	0	0	1	0	0	1	0	0	0	0	1	1	0	0	0	0	0	0	0	0	0	0	0	0	1
seedMaxE-7.seedMinPu0.0001.seedNoGU	10	0	0	0	0	0	0	1	0	0	1	0	0	0	0	0	0	0	0	1	0	0	0	0	0	1	0	0	1	0	0	0	0	1	1	0	1	0	0	0	0	0	0	1	0	0	0	1
seedMaxE-7.seedMinPu0.2	0	0	0	0	0	0	0	0	0	0	0	0	0	0	0	0	0	0	0	0	0	0	0	0	0	0	0	0	0	0	0	0	0	0	0	0	0	0	0	0	0	0	0	0	0	0	0	0
seedMaxE-7.seedMinPu0.2.seedNoGU	0	0	0	0	0	0	0	0	0	0	0	0	0	0	0	0	0	0	0	0	0	0	0	0	0	0	0	0	0	0	0	0	0	0	0	0	0	0	0	0	0	0	0	0	0	0	0	0
seedMaxE-7.seedMinPu0.5	0	0	0	0	0	0	0	0	0	0	0	0	0	0	0	0	0	0	0	0	0	0	0	0	0	0	0	0	0	0	0	0	0	0	0	0	0	0	0	0	0	0	0	0	0	0	0	0
seedMaxE-7.seedMinPu0.5.seedNoGU	0	0	0	0	0	0	0	0	0	0	0	0	0	0	0	0	0	0	0	0	0	0	0	0	0	0	0	0	0	0	0	0	0	0	0	0	0	0	0	0	0	0	0	0	0	0	0	0
seedMaxE-7.seedMinPu0.seedNoGU	10	0	0	0	0	0	0	1	0	0	1	0	0	0	0	0	0	0	0	1	0	0	0	0	0	1	0	0	1	0	0	0	0	1	1	0	1	0	0	0	0	0	0	1	0	0	0	1
seedMaxE-7.seedNoGU	10	0	0	0	0	0	0	1	0	0	1	0	0	0	0	0	0	0	0	1	0	0	0	0	0	1	0	0	1	0	0	0	0	1	1	0	1	0	0	0	0	0	0	1	0	0	0	1
seedMaxE-8	10	0	0	0	0	0	0	1	0	0	0	0	1	0	0	0	0	0	0	1	0	0	0	0	0	0	0	0	1	0	0	0	1	1	1	0	0	0	0	1	0	0	0	1	0	0	0	1
seedMaxE-8.seedMinPu0	10	0	0	0	0	0	0	1	0	0	0	0	1	0	0	0	0	0	0	1	0	0	0	0	0	0	0	0	1	0	0	0	1	1	1	0	0	0	0	1	0	0	0	1	0	0	0	1
seedMaxE-8.seedMinPu0.01	8	0	0	0	0	0	0	1	0	0	0	0	1	0	0	0	0	0	0	0	0	0	0	0	0	0	0	0	1	0	0	0	1	1	1	0	0	0	0	1	0	0	0	0	0	0	0	1
seedMaxE-8.seedMinPu0.0001	10	0	0	0	0	0	0	1	0	0	0	0	1	0	0	0	0	0	0	1	0	0	0	0	0	0	0	0	1	0	0	0	1	1	1	0	0	0	0	1	0	0	0	1	0	0	0	1
seedMaxE-8.seedMinPu0.001	9	0	0	0	0	0	0	1	0	0	0	0	1	0	0	0	0	0	0	0	0	0	0	0	0	0	0	0	1	0	0	0	1	1	1	0	0	0	0	1	0	0	0	1	0	0	0	1
seedMaxE-8.seedMinPu0.1	7	0	0	0	0	0	0	1	0	0	0	0	1	0	0	0	0	0	1	0	0	0	0	0	0	0	0	0	1	0	0	0	1	1	0	0	0	0	0	1	0	0	0	0	0	0	0	0
seedMaxE-8.seedMinPu0.1.seedNoGU	4	0	0	0	0	0	0	1	0	0	0	0	0	0	0	0	0	0	1	0	0	0	0	0	0	0	0	0	1	0	0	0	0	1	0	0	0	0	0	0	0	0	0	0	0	0	0	0
seedMaxE-8.seedMinPu0.001.seedNoGU	7	0	0	0	0	0	0	1	0	0	0	0	0	0	0	0	0	0	0	0	0	0	0	0	0	0	0	0	1	0	0	0	0	1	1	0	0	0	0	0	0	0	0	1	1	0	0	1
seedMaxE-8.seedMinPu0.0001.seedNoGU	8	0	0	0	0	0	0	1	0	0	0	0	0	0	0	0	0	0	0	1	0	0	0	0	0	0	0	0	1	0	0	0	0	1	1	0	0	0	0	0	0	0	0	1	1	0	0	1
seedMaxE-8.seedMinPu0.01.seedNoGU	5	0	0	0	0	0	0	1	0	0	0	0	0	0	0	0	0	0	0	0	0	0	0	0	0	0	0	0	1	0	0	0	0	1	1	0	0	0	0	0	0	0	0	0	0	0	0	1
seedMaxE-8.seedMinPu0.2	0	0	0	0	0	0	0	0	0	0	0	0	0	0	0	0	0	0	0	0	0	0	0	0	0	0	0	0	0	0	0	0	0	0	0	0	0	0	0	0	0	0	0	0	0	0	0	0
seedMaxE-8.seedMinPu0.2.seedNoGU	0	0	0	0	0	0	0	0	0	0	0	0	0	0	0	0	0	0	0	0	0	0	0	0	0	0	0	0	0	0	0	0	0	0	0	0	0	0	0	0	0	0	0	0	0	0	0	0
seedMaxE-8.seedMinPu0.5	0	0	0	0	0	0	0	0	0	0	0	0	0	0	0	0	0	0	0	0	0	0	0	0	0	0	0	0	0	0	0	0	0	0	0	0	0	0	0	0	0	0	0	0	0	0	0	0
seedMaxE-8.seedMinPu0.5.seedNoGU	0	0	0	0	0	0	0	0	0	0	0	0	0	0	0	0	0	0	0	0	0	0	0	0	0	0	0	0	0	0	0	0	0	0	0	0	0	0	0	0	0	0	0	0	0	0	0	0
seedMaxE-8.seedMinPu0.seedNoGU	8	0	0	0	0	0	0	1	0	0	0	0	0	0	0	0	0	0	0	1	0	0	0	0	0	0	0	0	1	0	0	0	0	1	1	0	0	0	0	0	0	0	0	1	1	0	0	1
seedMaxE-8.seedNoGU	8	0	0	0	0	0	0	1	0	0	0	0	0	0	0	0	0	0	0	1	0	0	0	0	0	0	0	0	1	0	0	0	0	1	1	0	0	0	0	0	0	0	0	1	1	0	0	1
seedMaxE-9	5	0	0	0	0	0	0	1	0	0	0	0	0	0	0	0	0	0	0	0	0	0	0	0	0	0	0	0	0	0	0	0	0	1	1	0	0	0	0	0	0	0	0	1	0	0	0	1
seedMaxE-9.seedMinPu0	5	0	0	0	0	0	0	1	0	0	0	0	0	0	0	0	0	0	0	0	0	0	0	0	0	0	0	0	0	0	0	0	0	1	1	0	0	0	0	0	0	0	0	1	0	0	0	1
seedMaxE-9.seedMinPu0.0001	5	0	0	0	0	0	0	1	0	0	0	0	0	0	0	0	0	0	0	0	0	0	0	0	0	0	0	0	0	0	0	0	0	1	1	0	0	0	0	0	0	0	0	1	0	0	0	1
seedMaxE-9.seedMinPu0.1	3	0	0	0	0	0	0	1	0	0	0	0	0	0	0	0	0	0	1	0	0	0	0	0	0	0	0	0	0	0	0	0	0	1	0	0	0	0	0	0	0	0	0	0	0	0	0	0
seedMaxE-9.seedMinPu0.01	5	0	0	0	0	0	0	1	0	0	0	0	0	0	0	0	0	0	1	0	0	0	0	0	0	0	0	0	0	0	0	0	0	1	1	0	0	0	0	0	0	0	0	0	0	0	0	1
seedMaxE-9.seedMinPu0.001	5	0	0	0	0	0	0	1	0	0	0	0	0	0	0	0	0	0	0	0	0	0	0	0	0	0	0	0	0	0	0	0	0	1	1	0	0	0	0	0	0	0	0	1	0	0	0	1
seedMaxE-9.seedMinPu0.001.seedNoGU	6	0	0	0	0	0	0	1	0	0	0	0	0	0	0	0	0	0	1	0	0	0	0	0	0	0	0	0	0	0	0	0	0	1	1	0	0	0	0	0	0	0	0	1	0	0	0	1
seedMaxE-9.seedMinPu0.1.seedNoGU	3	0	0	0	0	0	0	1	0	0	0	0	0	0	0	0	0	0	1	0	0	0	0	0	0	0	0	0	0	0	0	0	0	1	0	0	0	0	0	0	0	0	0	0	0	0	0	0
seedMaxE-9.seedMinPu0.01.seedNoGU	5	0	0	0	0	0	0	1	0	0	0	0	0	0	0	0	0	0	1	0	0	0	0	0	0	0	0	0	0	0	0	0	0	1	1	0	0	0	0	0	0	0	0	0	0	0	0	1
seedMaxE-9.seedMinPu0.0001.seedNoGU	6	0	0	0	0	0	0	1	0	0	0	0	0	0	0	0	0	0	1	0	0	0	0	0	0	0	0	0	0	0	0	0	0	1	1	0	0	0	0	0	0	0	0	1	0	0	0	1
seedMaxE-9.seedMinPu0.2	0	0	0	0	0	0	0	0	0	0	0	0	0	0	0	0	0	0	0	0	0	0	0	0	0	0	0	0	0	0	0	0	0	0	0	0	0	0	0	0	0	0	0	0	0	0	0	0
seedMaxE-9.seedMinPu0.2.seedNoGU	0	0	0	0	0	0	0	0	0	0	0	0	0	0	0	0	0	0	0	0	0	0	0	0	0	0	0	0	0	0	0	0	0	0	0	0	0	0	0	0	0	0	0	0	0	0	0	0
seedMaxE-9.seedMinPu0.5	0	0	0	0	0	0	0	0	0	0	0	0	0	0	0	0	0	0	0	0	0	0	0	0	0	0	0	0	0	0	0	0	0	0	0	0	0	0	0	0	0	0	0	0	0	0	0	0
seedMaxE-9.seedMinPu0.5.seedNoGU	0	0	0	0	0	0	0	0	0	0	0	0	0	0	0	0	0	0	0	0	0	0	0	0	0	0	0	0	0	0	0	0	0	0	0	0	0	0	0	0	0	0	0	0	0	0	0	0
seedMaxE-9.seedMinPu0.seedNoGU	6	0	0	0	0	0	0	1	0	0	0	0	0	0	0	0	0	0	1	0	0	0	0	0	0	0	0	0	0	0	0	0	0	1	1	0	0	0	0	0	0	0	0	1	0	0	0	1
seedMaxE-9.seedNoGU	6	0	0	0	0	0	0	1	0	0	0	0	0	0	0	0	0	0	1	0	0	0	0	0	0	0	0	0	0	0	0	0	0	1	1	0	0	0	0	0	0	0	0	1	0	0	0	1
seedMaxE-10	3	0	0	0	0	0	0	1	0	0	0	0	0	0	0	0	0	0	0	0	0	0	0	0	0	0	0	0	0	0	0	0	0	1	1	0	0	0	0	0	0	0	0	0	0	0	0	0

Salmonella typhimurium NC_003197

	sum	ArcZ-STM1682	ArcZ-STM2970	ArcZ-STM3216	ChiX-STM0687	ChiX-STM1313	CyaR-STM0833	GcvB-STM0002	GcvB-STM0245	GcvB-STM0399	GcvB-STM0602	GcvB-STM0665	GcvB-STM0959	GcvB-STM1299	GcvB-STM1452	GcvB-STM1746	GcvB-STM2355	GcvB-STM2526	GcvB-STM3062	GcvB-STM3064	GcvB-STM3225	GcvB-STM3564	GcvB-STM3567	GcvB-STM3630	GcvB-STM3903	GcvB-STM3909	GcvB-STM3930	GcvB-STM4351	GcvB-STM4398	MicA-STM4231	MicC-STM1572	MicF-STM0366	MicF-STM0959	MicF-STM1328	RybB-STM0413	RybB-STM0687	RybB-STM0999	RybB-STM1070	RybB-STM1473	RybB-STM1530	RybB-STM1572	RybB-STM1732	RybB-STM1995	RybB-STM2267	RybB-STM2391	SgrS-STM2945	SgrS-STM3962	Spot42-STM2190
Target found among top-100 predictions for the sRNA for any tested parameterization	40	1	0	0	1	1	1	1	1	1	1	1	1	1	0	0	1	1	1	1	1	1	1	1	1	1	1	1	1	1	1	1	1	1	1	1	1	1	1	1	1	0	0	1	1	0	1	1
seedMaxE-10.seedMinPu0	3	0	0	0	0	0	0	1	0	0	0	0	0	0	0	0	0	0	0	0	0	0	0	0	0	0	0	0	0	0	0	0	0	1	1	0	0	0	0	0	0	0	0	0	0	0	0	0
seedMaxE-10.seedMinPu0.1	2	0	0	0	0	0	0	1	0	0	0	0	0	0	0	0	0	0	0	0	0	0	0	0	0	0	0	0	0	0	0	0	0	1	0	0	0	0	0	0	0	0	0	0	0	0	0	0
seedMaxE-10.seedMinPu0.0001	3	0	0	0	0	0	0	1	0	0	0	0	0	0	0	0	0	0	0	0	0	0	0	0	0	0	0	0	0	0	0	0	0	1	1	0	0	0	0	0	0	0	0	0	0	0	0	0
seedMaxE-10.seedMinPu0.001	3	0	0	0	0	0	0	1	0	0	0	0	0	0	0	0	0	0	0	0	0	0	0	0	0	0	0	0	0	0	0	0	0	1	1	0	0	0	0	0	0	0	0	0	0	0	0	0
seedMaxE-10.seedMinPu0.01	3	0	0	0	0	0	0	1	0	0	0	0	0	0	0	0	0	0	0	0	0	0	0	0	0	0	0	0	0	0	0	0	0	1	1	0	0	0	0	0	0	0	0	0	0	0	0	0
seedMaxE-10.seedMinPu0.01.seedNoGU	3	0	0	0	0	0	0	1	0	0	0	0	0	0	0	0	0	0	0	0	0	0	0	0	0	0	0	0	0	0	0	0	0	1	1	0	0	0	0	0	0	0	0	0	0	0	0	0
seedMaxE-10.seedMinPu0.0001.seedNoGU	3	0	0	0	0	0	0	1	0	0	0	0	0	0	0	0	0	0	0	0	0	0	0	0	0	0	0	0	0	0	0	0	0	1	1	0	0	0	0	0	0	0	0	0	0	0	0	0
seedMaxE-10.seedMinPu0.1.seedNoGU	2	0	0	0	0	0	0	1	0	0	0	0	0	0	0	0	0	0	0	0	0	0	0	0	0	0	0	0	0	0	0	0	0	1	0	0	0	0	0	0	0	0	0	0	0	0	0	0
seedMaxE-10.seedMinPu0.001.seedNoGU	3	0	0	0	0	0	0	1	0	0	0	0	0	0	0	0	0	0	0	0	0	0	0	0	0	0	0	0	0	0	0	0	0	1	1	0	0	0	0	0	0	0	0	0	0	0	0	0
seedMaxE-10.seedMinPu0.2	0	0	0	0	0	0	0	0	0	0	0	0	0	0	0	0	0	0	0	0	0	0	0	0	0	0	0	0	0	0	0	0	0	0	0	0	0	0	0	0	0	0	0	0	0	0	0	0
seedMaxE-10.seedMinPu0.2.seedNoGU	0	0	0	0	0	0	0	0	0	0	0	0	0	0	0	0	0	0	0	0	0	0	0	0	0	0	0	0	0	0	0	0	0	0	0	0	0	0	0	0	0	0	0	0	0	0	0	0
seedMaxE-10.seedMinPu0.5	0	0	0	0	0	0	0	0	0	0	0	0	0	0	0	0	0	0	0	0	0	0	0	0	0	0	0	0	0	0	0	0	0	0	0	0	0	0	0	0	0	0	0	0	0	0	0	0
seedMaxE-10.seedMinPu0.5.seedNoGU	0	0	0	0	0	0	0	0	0	0	0	0	0	0	0	0	0	0	0	0	0	0	0	0	0	0	0	0	0	0	0	0	0	0	0	0	0	0	0	0	0	0	0	0	0	0	0	0
seedMaxE-10.seedMinPu0.seedNoGU	3	0	0	0	0	0	0	1	0	0	0	0	0	0	0	0	0	0	0	0	0	0	0	0	0	0	0	0	0	0	0	0	0	1	1	0	0	0	0	0	0	0	0	0	0	0	0	0
seedMaxE-10.seedNoGU	3	0	0	0	0	0	0	1	0	0	0	0	0	0	0	0	0	0	0	0	0	0	0	0	0	0	0	0	0	0	0	0	0	1	1	0	0	0	0	0	0	0	0	0	0	0	0	0
seedMaxEhybrid0	16	0	0	0	1	1	1	0	0	1	0	0	0	0	0	0	0	0	0	1	0	0	1	1	0	1	0	0	1	0	1	0	1	0	1	0	0	0	1	1	0	0	0	1	0	0	0	1
seedMaxEhybrid0.seedMinPu0	16	0	0	0	1	1	1	0	0	1	0	0	0	0	0	0	0	0	0	1	0	0	1	1	0	1	0	0	1	0	1	0	1	0	1	0	0	0	1	1	0	0	0	1	0	0	0	1
seedMaxEhybrid0.seedMinPu0.01	19	0	0	0	1	1	1	0	0	1	0	1	0	0	0	0	0	0	0	1	0	1	1	1	0	1	0	0	1	0	1	1	1	0	1	0	0	0	1	1	0	0	0	1	0	0	0	1
seedMaxEhybrid0.seedMinPu0.1	15	0	0	0	1	1	0	0	0	1	0	1	0	0	0	0	1	0	0	1	0	1	0	0	0	1	0	1	1	0	1	1	1	0	0	0	0	0	1	1	0	0	0	0	0	0	0	0
seedMaxEhybrid0.seedMinPu0.0001	16	0	0	0	1	1	1	0	0	1	0	0	0	0	0	0	0	0	0	1	0	0	1	1	0	1	0	0	1	0	1	0	1	0	1	0	0	0	1	1	0	0	0	1	0	0	0	1
seedMaxEhybrid0.seedMinPu0.001	17	0	0	0	1	1	1	0	0	1	0	0	0	0	0	0	0	0	0	1	0	0	1	1	0	1	0	0	1	0	1	1	1	0	1	0	0	0	1	1	0	0	0	1	0	0	0	1
seedMaxEhybrid0.seedMinPu0.1.seedNoGU	14	0	0	0	1	1	0	1	0	0	1	1	0	0	0	0	1	0	0	1	1	0	1	0	0	1	0	0	1	0	1	0	0	1	0	0	0	0	1	0	0	0	0	0	0	0	0	0
seedMaxEhybrid0.seedMinPu0.01.seedNoGU	17	0	0	0	1	1	1	0	0	1	0	1	0	0	0	0	1	0	0	1	0	0	1	1	0	1	0	1	1	0	1	0	0	0	1	0	0	0	1	0	0	0	0	1	0	0	0	1
seedMaxEhybrid0.seedMinPu0.001.seedNoGU	18	0	0	0	1	1	1	0	0	1	0	1	0	0	0	0	1	0	0	1	0	1	1	1	0	1	0	1	1	0	1	0	0	0	1	0	0	0	1	0	0	0	0	1	0	0	0	1
seedMaxEhybrid0.seedMinPu0.0001.seedNoGU	17	0	0	0	1	1	1	0	0	1	0	1	0	0	0	0	1	0	0	1	0	1	1	1	0	1	0	0	1	0	1	0	0	0	1	0	0	0	1	0	0	0	0	1	0	0	0	1
seedMaxEhybrid0.seedMinPu0.2	4	0	0	0	0	0	0	0	0	0	0	0	0	0	0	0	0	0	0	0	0	0	0	0	0	0	0	0	0	0	1	0	1	0	0	0	0	0	1	1	0	0	0	0	0	0	0	0
seedMaxEhybrid0.seedMinPu0.2.seedNoGU	2	0	0	0	0	0	0	0	0	0	0	0	0	0	0	0	0	0	0	0	0	0	0	0	0	0	0	0	0	0	1	0	0	0	0	0	0	0	1	0	0	0	0	0	0	0	0	0
seedMaxEhybrid0.seedMinPu0.5	0	0	0	0	0	0	0	0	0	0	0	0	0	0	0	0	0	0	0	0	0	0	0	0	0	0	0	0	0	0	0	0	0	0	0	0	0	0	0	0	0	0	0	0	0	0	0	0
seedMaxEhybrid0.seedMinPu0.5.seedNoGU	0	0	0	0	0	0	0	0	0	0	0	0	0	0	0	0	0	0	0	0	0	0	0	0	0	0	0	0	0	0	0	0	0	0	0	0	0	0	0	0	0	0	0	0	0	0	0	0
seedMaxEhybrid0.seedMinPu0.seedNoGU	17	0	0	0	1	1	1	0	0	1	0	1	0	0	0	0	1	0	0	1	0	1	1	1	0	1	0	0	1	0	1	0	0	0	1	0	0	0	1	0	0	0	0	1	0	0	0	1
seedMaxEhybrid0.seedNoGU	17	0	0	0	1	1	1	0	0	1	0	1	0	0	0	0	1	0	0	1	0	1	1	1	0	1	0	0	1	0	1	0	0	0	1	0	0	0	1	0	0	0	0	1	0	0	0	1
seedMaxEhybrid-1	16	0	0	0	1	1	1	0	0	1	0	0	0	0	0	0	0	0	0	1	0	0	1	1	0	1	0	0	1	0	1	0	1	0	1	0	0	0	1	1	0	0	0	1	0	0	0	1
seedMaxEhybrid-1.seedMinPu0	16	0	0	0	1	1	1	0	0	1	0	0	0	0	0	0	0	0	0	1	0	0	1	1	0	1	0	0	1	0	1	0	1	0	1	0	0	0	1	1	0	0	0	1	0	0	0	1
seedMaxEhybrid-1.seedMinPu0.0001	16	0	0	0	1	1	1	0	0	1	0	0	0	0	0	0	0	0	0	1	0	0	1	1	0	1	0	0	1	0	1	0	1	0	1	0	0	0	1	1	0	0	0	1	0	0	0	1
seedMaxEhybrid-1.seedMinPu0.01	19	0	0	0	1	1	1	0	0	1	0	1	0	0	0	0	0	0	0	1	0	1	1	1	0	1	0	0	1	0	1	1	1	0	1	0	0	0	1	1	0	0	0	1	0	0	0	1
seedMaxEhybrid-1.seedMinPu0.1	15	0	0	0	1	1	0	0	0	1	0	1	0	0	0	0	1	0	0	1	0	1	0	0	0	1	0	1	1	0	1	1	1	0	0	0	0	0	1	1	0	0	0	0	0	0	0	0
seedMaxEhybrid-1.seedMinPu0.001	17	0	0	0	1	1	1	0	0	1	0	0	0	0	0	0	0	0	0	1	0	0	1	1	0	1	0	0	1	0	1	1	1	0	1	0	0	0	1	1	0	0	0	1	0	0	0	1
seedMaxEhybrid-1.seedMinPu0.001.seedNoGU	18	0	0	0	1	1	1	0	0	1	0	1	0	0	0	0	1	0	0	1	0	1	1	1	0	1	0	1	1	0	1	0	0	0	1	0	0	0	1	0	0	0	0	1	0	0	0	1
seedMaxEhybrid-1.seedMinPu0.1.seedNoGU	14	0	0	0	1	1	0	1	0	0	1	1	0	0	0	0	1	0	0	1	1	0	1	0	0	1	0	0	1	0	1	0	0	1	0	0	0	0	1	0	0	0	0	0	0	0	0	0
seedMaxEhybrid-1.seedMinPu0.01.seedNoGU	17	0	0	0	1	1	1	0	0	1	0	1	0	0	0	0	1	0	0	1	0	0	1	1	0	1	0	1	1	0	1	0	0	0	1	0	0	0	1	0	0	0	0	1	0	0	0	1
seedMaxEhybrid-1.seedMinPu0.0001.seedNoGU	17	0	0	0	1	1	1	0	0	1	0	1	0	0	0	0	1	0	0	1	0	1	1	1	0	1	0	0	1	0	1	0	0	0	1	0	0	0	1	0	0	0	0	1	0	0	0	1
seedMaxEhybrid-1.seedMinPu0.2	4	0	0	0	0	0	0	0	0	0	0	0	0	0	0	0	0	0	0	0	0	0	0	0	0	0	0	0	0	0	1	0	1	0	0	0	0	0	1	1	0	0	0	0	0	0	0	0
seedMaxEhybrid-1.seedMinPu0.2.seedNoGU	2	0	0	0	0	0	0	0	0	0	0	0	0	0	0	0	0	0	0	0	0	0	0	0	0	0	0	0	0	0	1	0	0	0	0	0	0	0	1	0	0	0	0	0	0	0	0	0
seedMaxEhybrid-1.seedMinPu0.5	0	0	0	0	0	0	0	0	0	0	0	0	0	0	0	0	0	0	0	0	0	0	0	0	0	0	0	0	0	0	0	0	0	0	0	0	0	0	0	0	0	0	0	0	0	0	0	0
seedMaxEhybrid-1.seedMinPu0.5.seedNoGU	0	0	0	0	0	0	0	0	0	0	0	0	0	0	0	0	0	0	0	0	0	0	0	0	0	0	0	0	0	0	0	0	0	0	0	0	0	0	0	0	0	0	0	0	0	0	0	0
seedMaxEhybrid-1.seedMinPu0.seedNoGU	17	0	0	0	1	1	1	0	0	1	0	1	0	0	0	0	1	0	0	1	0	1	1	1	0	1	0	0	1	0	1	0	0	0	1	0	0	0	1	0	0	0	0	1	0	0	0	1
seedMaxEhybrid-1.seedNoGU	17	0	0	0	1	1	1	0	0	1	0	1	0	0	0	0	1	0	0	1	0	1	1	1	0	1	0	0	1	0	1	0	0	0	1	0	0	0	1	0	0	0	0	1	0	0	0	1
seedMaxEhybrid-2	16	0	0	0	1	1	1	0	0	1	0	0	0	0	0	0	0	0	0	1	0	0	1	1	0	1	0	0	1	0	1	0	1	0	1	0	0	0	1	1	0	0	0	1	0	0	0	1
seedMaxEhybrid-2.seedMinPu0	16	0	0	0	1	1	1	0	0	1	0	0	0	0	0	0	0	0	0	1	0	0	1	1	0	1	0	0	1	0	1	0	1	0	1	0	0	0	1	1	0	0	0	1	0	0	0	1
seedMaxEhybrid-2.seedMinPu0.01	19	0	0	0	1	1	1	0	0	1	0	1	0	0	0	0	0	0	0	1	0	1	1	1	0	1	0	0	1	0	1	1	1	0	1	0	0	0	1	1	0	0	0	1	0	0	0	1
seedMaxEhybrid-2.seedMinPu0.001	17	0	0	0	1	1	1	0	0	1	0	0	0	0	0	0	0	0	0	1	0	0	1	1	0	1	0	0	1	0	1	1	1	0	1	0	0	0	1	1	0	0	0	1	0	0	0	1
seedMaxEhybrid-2.seedMinPu0.0001	16	0	0	0	1	1	1	0	0	1	0	0	0	0	0	0	0	0	0	1	0	0	1	1	0	1	0	0	1	0	1	0	1	0	1	0	0	0	1	1	0	0	0	1	0	0	0	1
seedMaxEhybrid-2.seedMinPu0.1	15	0	0	0	1	1	0	0	0	1	0	1	0	0	0	0	1	0	0	1	0	1	0	0	0	1	0	1	1	0	1	1	1	0	0	0	0	0	1	1	0	0	0	0	0	0	0	0
seedMaxEhybrid-2.seedMinPu0.001.seedNoGU	18	0	0	0	1	1	1	0	0	1	0	1	0	0	0	0	1	0	0	1	0	1	1	1	0	1	0	1	1	0	1	0	0	0	1	0	0	0	1	0	0	0	0	1	0	0	0	1
seedMaxEhybrid-2.seedMinPu0.1.seedNoGU	14	0	0	0	1	1	0	1	0	0	1	1	0	0	0	0	1	0	0	1	1	0	1	0	0	1	0	0	1	0	1	0	0	1	0	0	0	0	1	0	0	0	0	0	0	0	0	0
seedMaxEhybrid-2.seedMinPu0.0001.seedNoGU	17	0	0	0	1	1	1	0	0	1	0	1	0	0	0	0	1	0	0	1	0	1	1	1	0	1	0	0	1	0	1	0	0	0	1	0	0	0	1	0	0	0	0	1	0	0	0	1

Salmonella typhimurium NC_003197

	sum	ArcZ-STM1682	ArcZ-STM2970	ArcZ-STM3216	ChiX-STM0687	ChiX-STM1313	CyaR-STM0833	GcvB-STM0002	GcvB-STM0245	GcvB-STM0399	GcvB-STM0602	GcvB-STM0665	GcvB-STM0959	GcvB-STM1299	GcvB-STM1452	GcvB-STM1746	GcvB-STM2355	GcvB-STM2526	GcvB-STM3062	GcvB-STM3064	GcvB-STM3225	GcvB-STM3564	GcvB-STM3567	GcvB-STM3630	GcvB-STM3903	GcvB-STM3909	GcvB-STM3930	GcvB-STM4351	GcvB-STM4398	MicA-STM4231	MicC-STM1572	MicF-STM0366	MicF-STM0959	MicF-STM1328	RybB-STM0413	RybB-STM0687	RybB-STM0999	RybB-STM1070	RybB-STM1473	RybB-STM1530	RybB-STM1572	RybB-STM1732	RybB-STM1995	RybB-STM2267	RybB-STM2391	SgrS-STM2945	SgrS-STM3962	Spot42-STM2190
Target found among top-100 predictions for the sRNA for any tested parameterization	40	1	0	0	1	1	1	1	1	1	1	1	1	1	0	0	1	1	1	1	1	1	1	1	1	1	1	1	1	1	1	1	1	1	1	1	1	1	1	1	1	0	0	1	1	0	1	1
seedMaxEhybrid-2.seedMinPu0.01.seedNoGU	17	0	0	0	1	1	1	0	0	1	0	1	0	0	0	0	1	0	0	1	0	0	1	1	0	1	0	1	1	0	1	0	0	0	1	0	0	0	1	0	0	0	0	1	0	0	0	1
seedMaxEhybrid-2.seedMinPu0.2	4	0	0	0	0	0	0	0	0	0	0	0	0	0	0	0	0	0	0	0	0	0	0	0	0	0	0	0	0	0	1	0	1	0	0	0	0	0	1	1	0	0	0	0	0	0	0	0
seedMaxEhybrid-2.seedMinPu0.2.seedNoGU	2	0	0	0	0	0	0	0	0	0	0	0	0	0	0	0	0	0	0	0	0	0	0	0	0	0	0	0	0	0	1	0	0	0	0	0	0	0	1	0	0	0	0	0	0	0	0	0
seedMaxEhybrid-2.seedMinPu0.5	0	0	0	0	0	0	0	0	0	0	0	0	0	0	0	0	0	0	0	0	0	0	0	0	0	0	0	0	0	0	0	0	0	0	0	0	0	0	0	0	0	0	0	0	0	0	0	0
seedMaxEhybrid-2.seedMinPu0.5.seedNoGU	0	0	0	0	0	0	0	0	0	0	0	0	0	0	0	0	0	0	0	0	0	0	0	0	0	0	0	0	0	0	0	0	0	0	0	0	0	0	0	0	0	0	0	0	0	0	0	0
seedMaxEhybrid-2.seedMinPu0.seedNoGU	17	0	0	0	1	1	1	0	0	1	0	1	0	0	0	0	1	0	0	1	0	1	1	1	0	1	0	0	1	0	1	0	0	0	1	0	0	0	1	0	0	0	0	1	0	0	0	1
seedMaxEhybrid-2.seedNoGU	17	0	0	0	1	1	1	0	0	1	0	1	0	0	0	0	1	0	0	1	0	1	1	1	0	1	0	0	1	0	1	0	0	0	1	0	0	0	1	0	0	0	0	1	0	0	0	1
seedMaxEhybrid-3	16	0	0	0	1	1	1	0	0	1	0	0	0	0	0	0	0	0	0	1	0	0	1	1	0	1	0	0	1	0	1	0	1	0	1	0	0	0	1	1	0	0	0	1	0	0	0	1
seedMaxEhybrid-3.seedMinPu0	16	0	0	0	1	1	1	0	0	1	0	0	0	0	0	0	0	0	0	1	0	0	1	1	0	1	0	0	1	0	1	0	1	0	1	0	0	0	1	1	0	0	0	1	0	0	0	1
seedMaxEhybrid-3.seedMinPu0.01	19	0	0	0	1	1	1	0	0	1	0	1	0	0	0	0	0	0	0	1	0	1	1	1	0	1	0	0	1	0	1	1	1	0	1	0	0	0	1	1	0	0	0	1	0	0	0	1
seedMaxEhybrid-3.seedMinPu0.1	15	0	0	0	1	1	0	0	0	1	0	1	0	0	0	0	1	0	0	1	0	1	0	0	0	1	0	1	1	0	1	1	1	0	0	0	0	0	1	1	0	0	0	0	0	0	0	0
seedMaxEhybrid-3.seedMinPu0.001	17	0	0	0	1	1	1	0	0	1	0	0	0	0	0	0	0	0	0	1	0	0	1	1	0	1	0	0	1	0	1	1	1	0	1	0	0	0	1	1	0	0	0	1	0	0	0	1
seedMaxEhybrid-3.seedMinPu0.0001	16	0	0	0	1	1	1	0	0	1	0	0	0	0	0	0	0	0	0	1	0	0	1	1	0	1	0	0	1	0	1	0	1	0	1	0	0	0	1	1	0	0	0	1	0	0	0	1
seedMaxEhybrid-3.seedMinPu0.001.seedNoGU	18	0	0	0	1	1	1	0	0	1	0	1	0	0	0	0	1	0	0	1	0	1	1	1	0	1	0	1	1	0	1	0	0	0	1	0	0	0	1	0	0	0	0	1	0	0	0	1
seedMaxEhybrid-3.seedMinPu0.01.seedNoGU	17	0	0	0	1	1	1	0	0	1	0	1	0	0	0	0	1	0	0	1	0	0	1	1	0	1	0	1	1	0	1	0	0	0	1	0	0	0	1	0	0	0	0	1	0	0	0	1
seedMaxEhybrid-3.seedMinPu0.1.seedNoGU	14	0	0	0	1	1	0	1	0	0	1	1	0	0	0	0	1	0	0	1	1	0	1	0	0	1	0	0	1	0	1	0	0	1	0	0	0	0	1	0	0	0	0	0	0	0	0	0
seedMaxEhybrid-3.seedMinPu0.0001.seedNoGU	17	0	0	0	1	1	1	0	0	1	0	1	0	0	0	0	1	0	0	1	0	1	1	1	0	1	0	0	1	0	1	0	0	0	1	0	0	0	1	0	0	0	0	1	0	0	0	1
seedMaxEhybrid-3.seedMinPu0.2	3	0	0	0	0	0	0	0	0	0	0	0	0	0	0	0	0	0	0	0	0	0	0	0	0	0	0	0	0	0	1	0	0	0	0	0	0	0	1	1	0	0	0	0	0	0	0	0
seedMaxEhybrid-3.seedMinPu0.2.seedNoGU	2	0	0	0	0	0	0	0	0	0	0	0	0	0	0	0	0	0	0	0	0	0	0	0	0	0	0	0	0	0	1	0	0	0	0	0	0	0	1	0	0	0	0	0	0	0	0	0
seedMaxEhybrid-3.seedMinPu0.5	0	0	0	0	0	0	0	0	0	0	0	0	0	0	0	0	0	0	0	0	0	0	0	0	0	0	0	0	0	0	0	0	0	0	0	0	0	0	0	0	0	0	0	0	0	0	0	0
seedMaxEhybrid-3.seedMinPu0.5.seedNoGU	0	0	0	0	0	0	0	0	0	0	0	0	0	0	0	0	0	0	0	0	0	0	0	0	0	0	0	0	0	0	0	0	0	0	0	0	0	0	0	0	0	0	0	0	0	0	0	0
seedMaxEhybrid-3.seedMinPu0.seedNoGU	17	0	0	0	1	1	1	0	0	1	0	1	0	0	0	0	1	0	0	1	0	1	1	1	0	1	0	0	1	0	1	0	0	0	1	0	0	0	1	0	0	0	0	1	0	0	0	1
seedMaxEhybrid-3.seedNoGU	17	0	0	0	1	1	1	0	0	1	0	1	0	0	0	0	1	0	0	1	0	1	1	1	0	1	0	0	1	0	1	0	0	0	1	0	0	0	1	0	0	0	0	1	0	0	0	1
seedMaxEhybrid-4	16	0	0	0	1	1	1	0	0	1	0	0	0	0	0	0	0	0	0	1	0	0	1	1	0	1	0	0	1	0	1	0	1	0	1	0	0	0	1	1	0	0	0	1	0	0	0	1
seedMaxEhybrid-4.seedMinPu0	16	0	0	0	1	1	1	0	0	1	0	0	0	0	0	0	0	0	0	1	0	0	1	1	0	1	0	0	1	0	1	0	1	0	1	0	0	0	1	1	0	0	0	1	0	0	0	1
seedMaxEhybrid-4.seedMinPu0.01	19	0	0	0	1	1	1	0	0	1	0	1	0	0	0	0	0	0	0	1	0	1	1	1	0	1	0	0	1	0	1	1	1	0	1	0	0	0	1	1	0	0	0	1	0	0	0	1
seedMaxEhybrid-4.seedMinPu0.0001	16	0	0	0	1	1	1	0	0	1	0	0	0	0	0	0	0	0	0	1	0	0	1	1	0	1	0	0	1	0	1	0	1	0	1	0	0	0	1	1	0	0	0	1	0	0	0	1
seedMaxEhybrid-4.seedMinPu0.001	17	0	0	0	1	1	1	0	0	1	0	0	0	0	0	0	0	0	0	1	0	0	1	1	0	1	0	0	1	0	1	1	1	0	1	0	0	0	1	1	0	0	0	1	0	0	0	1
seedMaxEhybrid-4.seedMinPu0.1	17	0	0	0	1	1	0	0	0	1	0	1	0	1	0	0	1	0	0	1	0	1	0	0	0	1	0	1	1	0	1	1	1	1	0	0	0	0	1	1	0	0	0	0	0	0	0	0
seedMaxEhybrid-4.seedMinPu0.01.seedNoGU	16	0	0	0	1	1	1	0	0	1	0	1	0	0	0	0	1	0	0	1	0	0	1	1	0	1	0	1	1	0	0	0	0	0	1	0	0	0	1	0	0	0	0	1	0	0	0	1
seedMaxEhybrid-4.seedMinPu0.001.seedNoGU	17	0	0	0	1	1	1	0	0	1	0	1	0	0	0	0	1	0	0	1	0	1	1	1	0	1	0	1	1	0	0	0	0	0	1	0	0	0	1	0	0	0	0	1	0	0	0	1
seedMaxEhybrid-4.seedMinPu0.1.seedNoGU	13	0	0	0	1	1	0	1	0	0	1	1	0	0	0	0	1	0	0	1	1	0	1	0	0	1	0	0	1	0	0	0	0	1	0	0	0	0	1	0	0	0	0	0	0	0	0	0
seedMaxEhybrid-4.seedMinPu0.0001.seedNoGU	16	0	0	0	1	1	1	0	0	1	0	1	0	0	0	0	1	0	0	1	0	1	1	1	0	1	0	0	1	0	0	0	0	0	1	0	0	0	1	0	0	0	0	1	0	0	0	1
seedMaxEhybrid-4.seedMinPu0.2	3	0	0	0	0	0	0	0	0	0	0	0	0	0	0	0	0	0	0	0	0	0	0	0	0	0	0	0	0	0	1	0	0	0	0	0	0	0	1	1	0	0	0	0	0	0	0	0
seedMaxEhybrid-4.seedMinPu0.2.seedNoGU	1	0	0	0	0	0	0	0	0	0	0	0	0	0	0	0	0	0	0	0	0	0	0	0	0	0	0	0	0	0	0	0	0	0	0	0	0	0	1	0	0	0	0	0	0	0	0	0
seedMaxEhybrid-4.seedMinPu0.5	0	0	0	0	0	0	0	0	0	0	0	0	0	0	0	0	0	0	0	0	0	0	0	0	0	0	0	0	0	0	0	0	0	0	0	0	0	0	0	0	0	0	0	0	0	0	0	0
seedMaxEhybrid-4.seedMinPu0.5.seedNoGU	0	0	0	0	0	0	0	0	0	0	0	0	0	0	0	0	0	0	0	0	0	0	0	0	0	0	0	0	0	0	0	0	0	0	0	0	0	0	0	0	0	0	0	0	0	0	0	0
seedMaxEhybrid-4.seedMinPu0.seedNoGU	16	0	0	0	1	1	1	0	0	1	0	1	0	0	0	0	1	0	0	1	0	1	1	1	0	1	0	0	1	0	0	0	0	0	1	0	0	0	1	0	0	0	0	1	0	0	0	1
seedMaxEhybrid-4.seedNoGU	16	0	0	0	1	1	1	0	0	1	0	1	0	0	0	0	1	0	0	1	0	1	1	1	0	1	0	0	1	0	0	0	0	0	1	0	0	0	1	0	0	0	0	1	0	0	0	1
seedMaxEhybrid-5	17	0	0	0	1	1	1	0	0	1	0	0	0	0	0	0	0	0	0	1	0	0	1	1	0	1	0	0	1	0	1	1	1	0	1	0	0	0	1	1	0	0	0	1	0	0	0	1
seedMaxEhybrid-5.seedMinPu0	17	0	0	0	1	1	1	0	0	1	0	0	0	0	0	0	0	0	0	1	0	0	1	1	0	1	0	0	1	0	1	1	1	0	1	0	0	0	1	1	0	0	0	1	0	0	0	1
seedMaxEhybrid-5.seedMinPu0.0001	17	0	0	0	1	1	1	0	0	1	0	0	0	0	0	0	0	0	0	1	0	0	1	1	0	1	0	0	1	0	1	1	1	0	1	0	0	0	1	1	0	0	0	1	0	0	0	1
seedMaxEhybrid-5.seedMinPu0.01	18	0	0	0	1	1	1	0	0	1	0	1	0	0	0	0	0	0	0	1	0	0	1	1	0	1	0	0	1	0	1	1	1	0	1	0	0	0	1	1	0	0	0	1	0	0	0	1
seedMaxEhybrid-5.seedMinPu0.1	18	0	0	0	1	1	0	1	0	1	0	1	0	1	0	0	1	0	0	0	0	0	1	0	0	1	1	1	1	0	1	1	1	1	0	0	0	0	1	1	0	0	0	0	0	0	0	0
seedMaxEhybrid-5.seedMinPu0.001	17	0	0	0	1	1	1	0	0	1	0	0	0	0	0	0	0	0	0	1	0	0	1	1	0	1	0	0	1	0	1	1	1	0	1	0	0	0	1	1	0	0	0	1	0	0	0	1
seedMaxEhybrid-5.seedMinPu0.01.seedNoGU	17	0	0	0	1	1	1	0	0	1	0	1	0	0	0	0	1	0	0	1	0	0	1	1	0	1	0	1	1	0	0	0	0	1	1	0	0	0	1	0	0	0	0	1	0	0	0	1
seedMaxEhybrid-5.seedMinPu0.0001.seedNoGU	16	0	0	0	1	1	1	0	0	1	0	1	0	0	0	0	1	0	0	1	0	1	1	1	0	1	0	0	1	0	0	0	0	0	1	0	0	0	1	0	0	0	0	1	0	0	0	1
seedMaxEhybrid-5.seedMinPu0.1.seedNoGU	13	0	0	0	1	1	0	1	0	0	1	1	0	0	0	0	1	0	0	1	1	0	1	0	0	1	0	0	1	0	0	0	0	1	0	0	0	0	1	0	0	0	0	0	0	0	0	0
seedMaxEhybrid-5.seedMinPu0.001.seedNoGU	17	0	0	0	1	1	1	0	0	1	0	1	0	0	0	0	1	0	0	1	0	1	1	1	0	1	0	1	1	0	0	0	0	0	1	0	0	0	1	0	0	0	0	1	0	0	0	1
seedMaxEhybrid-5.seedMinPu0.2	2	0	0	0	0	0	0	0	0	0	0	0	0	0	0	0	0	0	0	0	0	0	0	0	0	0	0	0	0	0	1	0	0	0	0	0	0	0	1	0	0	0	0	0	0	0	0	0
seedMaxEhybrid-5.seedMinPu0.2.seedNoGU	1	0	0	0	0	0	0	0	0	0	0	0	0	0	0	0	0	0	0	0	0	0	0	0	0	0	0	0	0	0	0	0	0	0	0	0	0	0	1	0	0	0	0	0	0	0	0	0
seedMaxEhybrid-5.seedMinPu0.5	0	0	0	0	0	0	0	0	0	0	0	0	0	0	0	0	0	0	0	0	0	0	0	0	0	0	0	0	0	0	0	0	0	0	0	0	0	0	0	0	0	0	0	0	0	0	0	0
seedMaxEhybrid-5.seedMinPu0.5.seedNoGU	0	0	0	0	0	0	0	0	0	0	0	0	0	0	0	0	0	0	0	0	0	0	0	0	0	0	0	0	0	0	0	0	0	0	0	0	0	0	0	0	0	0	0	0	0	0	0	0
seedMaxEhybrid-5.seedMinPu0.seedNoGU	16	0	0	0	1	1	1	0	0	1	0	1	0	0	0	0	1	0	0	1	0	1	1	1	0	1	0	0	1	0	0	0	0	0	1	0	0	0	1	0	0	0	0	1	0	0	0	1
seedMaxEhybrid-5.seedNoGU	16	0	0	0	1	1	1	0	0	1	0	1	0	0	0	0	1	0	0	1	0	1	1	1	0	1	0	0	1	0	0	0	0	0	1	0	0	0	1	0	0	0	0	1	0	0	0	1
seedMaxEhybrid-6	16	0	0	0	1	1	1	0	0	1	0	0	0	0	0	0	0	0	0	1	0	0	1	1	0	1	0	0	1	0	1	1	1	0	1	0	0	0	0	1	0	0	0	1	0	0	0	1

Salmonella typhimurium NC_003197

	sum	ArcZ-STM1682	ArcZ-STM2970	ArcZ-STM3216	ChiX-STM0687	ChiX-STM1313	CyaR-STM0833	GcvB-STM0002	GcvB-STM0245	GcvB-STM0399	GcvB-STM0602	GcvB-STM0665	GcvB-STM0959	GcvB-STM1299	GcvB-STM1452	GcvB-STM1746	GcvB-STM2355	GcvB-STM2526	GcvB-STM3062	GcvB-STM3064	GcvB-STM3225	GcvB-STM3564	GcvB-STM3567	GcvB-STM3630	GcvB-STM3903	GcvB-STM3909	GcvB-STM3930	GcvB-STM4351	GcvB-STM4398	MicA-STM4231	MicC-STM1572	MicF-STM0366	MicF-STM0959	MicF-STM1328	RybB-STM0413	RybB-STM0687	RybB-STM0999	RybB-STM1070	RybB-STM1473	RybB-STM1530	RybB-STM1572	RybB-STM1732	RybB-STM1995	RybB-STM2267	RybB-STM2391	SgrS-STM2945	SgrS-STM3962	Spot42-STM2190
Target found among top-100 predictions for the sRNA for any tested parameterization	40	1	0	0	1	1	1	1	1	1	1	1	1	1	0	0	1	1	1	1	1	1	1	1	1	1	1	1	1	1	1	1	1	1	1	1	1	1	1	1	1	0	0	1	1	0	1	1
seedMaxEhybrid-6.seedMinPu0	16	0	0	0	1	1	1	0	0	1	0	0	0	0	0	0	0	0	0	1	0	0	1	1	0	1	0	0	1	0	1	1	1	0	1	0	0	0	0	1	0	0	0	1	0	0	0	1
seedMaxEhybrid-6.seedMinPu0.1	13	0	0	0	1	1	0	1	0	1	0	1	0	0	0	0	1	0	0	0	0	0	1	0	0	1	0	0	1	0	1	0	1	1	0	0	0	0	0	1	0	0	0	0	0	0	0	0
seedMaxEhybrid-6.seedMinPu0.0001	16	0	0	0	1	1	1	0	0	1	0	0	0	0	0	0	0	0	0	1	0	0	1	1	0	1	0	0	1	0	1	1	1	0	1	0	0	0	0	1	0	0	0	1	0	0	0	1
seedMaxEhybrid-6.seedMinPu0.001	18	0	0	0	1	1	1	0	0	1	0	1	0	0	0	0	0	0	0	1	0	1	1	1	0	1	0	0	1	0	1	1	1	0	1	0	0	0	0	1	0	0	0	1	0	0	0	1
seedMaxEhybrid-6.seedMinPu0.01	18	0	0	0	1	1	1	0	0	1	0	1	0	0	0	0	1	0	0	1	0	0	1	1	0	1	0	0	1	0	1	1	1	0	1	0	0	0	0	1	0	0	0	1	0	0	0	1
seedMaxEhybrid-6.seedMinPu0.0001.seedNoGU	15	0	0	0	1	1	1	0	0	1	0	1	0	0	0	0	1	0	0	1	0	1	1	1	0	1	0	0	1	0	0	0	0	0	1	0	0	0	0	0	0	0	0	1	0	0	0	1
seedMaxEhybrid-6.seedMinPu0.01.seedNoGU	16	0	0	0	1	1	1	0	0	1	0	1	0	0	0	0	1	0	0	1	0	0	1	1	0	1	0	1	1	0	0	0	0	1	1	0	0	0	0	0	0	0	0	1	0	0	0	1
seedMaxEhybrid-6.seedMinPu0.1.seedNoGU	12	0	0	0	1	1	0	1	0	0	1	1	0	0	0	0	1	0	0	1	1	0	1	0	0	1	0	0	1	0	0	0	0	1	0	0	0	0	0	0	0	0	0	0	0	0	0	0
seedMaxEhybrid-6.seedMinPu0.001.seedNoGU	17	0	0	0	1	1	1	0	0	1	0	1	0	0	0	0	1	0	0	1	0	1	1	1	0	1	0	1	1	0	0	0	0	1	1	0	0	0	0	0	0	0	0	1	0	0	0	1
seedMaxEhybrid-6.seedMinPu0.2	1	0	0	0	0	0	0	0	0	0	0	0	0	0	0	0	0	0	0	0	0	0	0	0	0	0	0	0	0	0	1	0	0	0	0	0	0	0	0	0	0	0	0	0	0	0	0	0
seedMaxEhybrid-6.seedMinPu0.2.seedNoGU	1	0	0	0	0	0	0	0	0	0	0	0	0	0	0	0	0	0	0	0	0	0	0	0	0	0	0	0	0	0	0	0	0	0	0	0	0	0	0	0	0	0	0	1	0	0	0	0
seedMaxEhybrid-6.seedMinPu0.5	0	0	0	0	0	0	0	0	0	0	0	0	0	0	0	0	0	0	0	0	0	0	0	0	0	0	0	0	0	0	0	0	0	0	0	0	0	0	0	0	0	0	0	0	0	0	0	0
seedMaxEhybrid-6.seedMinPu0.5.seedNoGU	0	0	0	0	0	0	0	0	0	0	0	0	0	0	0	0	0	0	0	0	0	0	0	0	0	0	0	0	0	0	0	0	0	0	0	0	0	0	0	0	0	0	0	0	0	0	0	0
seedMaxEhybrid-6.seedMinPu0.seedNoGU	15	0	0	0	1	1	1	0	0	1	0	1	0	0	0	0	1	0	0	1	0	1	1	1	0	1	0	0	1	0	0	0	0	0	1	0	0	0	0	0	0	0	0	1	0	0	0	1
seedMaxEhybrid-6.seedNoGU	15	0	0	0	1	1	1	0	0	1	0	1	0	0	0	0	1	0	0	1	0	1	1	1	0	1	0	0	1	0	0	0	0	0	1	0	0	0	0	0	0	0	0	1	0	0	0	1
seedMaxEhybrid-7	18	0	0	0	1	1	1	0	0	1	0	1	0	0	0	0	0	0	0	1	0	1	1	1	0	1	0	0	1	0	1	1	1	0	1	0	0	0	0	1	0	0	0	1	0	0	0	1
seedMaxEhybrid-7.seedMinPu0	18	0	0	0	1	1	1	0	0	1	0	1	0	0	0	0	0	0	0	1	0	1	1	1	0	1	0	0	1	0	1	1	1	0	1	0	0	0	0	1	0	0	0	1	0	0	0	1
seedMaxEhybrid-7.seedMinPu0.001	18	0	0	0	1	0	1	0	0	1	0	1	0	0	0	0	1	0	0	1	0	1	1	1	0	1	0	0	1	0	1	1	1	0	1	0	0	0	0	1	0	0	0	1	0	0	0	1
seedMaxEhybrid-7.seedMinPu0.1	13	0	0	0	0	0	0	1	0	0	1	1	1	0	0	0	0	0	0	1	0	0	1	1	0	1	0	0	1	0	1	0	1	1	0	0	0	0	0	1	0	0	0	0	0	0	0	0
seedMaxEhybrid-7.seedMinPu0.01	14	0	0	0	1	0	1	0	0	0	0	0	0	0	0	0	1	0	0	1	0	0	1	0	0	1	0	0	1	0	1	1	1	0	1	0	0	0	0	1	0	0	0	1	0	0	0	1
seedMaxEhybrid-7.seedMinPu0.0001	18	0	0	0	1	1	1	0	0	1	0	1	0	0	0	0	0	0	0	1	0	1	1	1	0	1	0	0	1	0	1	1	1	0	1	0	0	0	0	1	0	0	0	1	0	0	0	1
seedMaxEhybrid-7.seedMinPu0.1.seedNoGU	10	0	0	0	0	0	0	1	0	0	1	0	0	0	0	0	1	0	1	1	0	0	1	0	1	1	0	0	1	0	0	0	0	1	0	0	0	0	0	0	0	0	0	0	0	0	0	0
seedMaxEhybrid-7.seedMinPu0.001.seedNoGU	15	0	0	0	1	0	1	0	0	1	0	1	0	0	0	0	1	0	0	1	0	1	1	1	0	1	0	0	1	0	0	0	0	1	1	0	0	0	0	0	0	0	0	1	0	0	0	1
seedMaxEhybrid-7.seedMinPu0.0001.seedNoGU	15	0	0	0	1	1	1	0	0	1	0	1	0	0	0	0	1	0	0	1	0	1	1	1	0	1	0	0	1	0	0	0	0	0	1	0	0	0	0	0	0	0	0	1	0	0	0	1
seedMaxEhybrid-7.seedMinPu0.01.seedNoGU	10	0	0	0	1	0	1	0	0	0	0	0	0	0	0	0	0	0	0	1	0	0	1	0	0	1	0	0	1	0	0	0	0	1	1	0	0	0	0	0	0	0	0	1	0	0	0	1
seedMaxEhybrid-7.seedMinPu0.2	1	0	0	0	0	0	0	0	0	0	0	0	0	0	0	0	0	0	0	0	0	0	0	0	0	0	0	0	0	0	1	0	0	0	0	0	0	0	0	0	0	0	0	0	0	0	0	0
seedMaxEhybrid-7.seedMinPu0.2.seedNoGU	0	0	0	0	0	0	0	0	0	0	0	0	0	0	0	0	0	0	0	0	0	0	0	0	0	0	0	0	0	0	0	0	0	0	0	0	0	0	0	0	0	0	0	0	0	0	0	0
seedMaxEhybrid-7.seedMinPu0.5	0	0	0	0	0	0	0	0	0	0	0	0	0	0	0	0	0	0	0	0	0	0	0	0	0	0	0	0	0	0	0	0	0	0	0	0	0	0	0	0	0	0	0	0	0	0	0	0
seedMaxEhybrid-7.seedMinPu0.5.seedNoGU	0	0	0	0	0	0	0	0	0	0	0	0	0	0	0	0	0	0	0	0	0	0	0	0	0	0	0	0	0	0	0	0	0	0	0	0	0	0	0	0	0	0	0	0	0	0	0	0
seedMaxEhybrid-7.seedMinPu0.seedNoGU	15	0	0	0	1	1	1	0	0	1	0	1	0	0	0	0	1	0	0	1	0	1	1	1	0	1	0	0	1	0	0	0	0	0	1	0	0	0	0	0	0	0	0	1	0	0	0	1
seedMaxEhybrid-7.seedNoGU	15	0	0	0	1	1	1	0	0	1	0	1	0	0	0	0	1	0	0	1	0	1	1	1	0	1	0	0	1	0	0	0	0	0	1	0	0	0	0	0	0	0	0	1	0	0	0	1
seedMaxEhybrid-8	10	0	0	0	1	1	1	0	0	1	0	0	0	0	0	0	0	0	0	0	0	0	0	0	0	0	0	0	1	0	0	0	0	1	1	0	0	0	0	1	0	0	0	1	0	0	0	1
seedMaxEhybrid-8.seedMinPu0	10	0	0	0	1	1	1	0	0	1	0	0	0	0	0	0	0	0	0	0	0	0	0	0	0	0	0	0	1	0	0	0	0	1	1	0	0	0	0	1	0	0	0	1	0	0	0	1
seedMaxEhybrid-8.seedMinPu0.0001	10	0	0	0	1	1	1	0	0	1	0	0	0	0	0	0	0	0	0	0	0	0	0	0	0	0	0	0	1	0	0	0	0	1	1	0	0	0	0	1	0	0	0	1	0	0	0	1
seedMaxEhybrid-8.seedMinPu0.01	7	0	0	0	1	0	0	0	0	0	0	0	0	0	0	0	0	0	0	0	0	0	0	0	0	0	0	0	1	0	0	0	0	1	1	0	0	0	0	1	0	0	0	1	0	0	0	1
seedMaxEhybrid-8.seedMinPu0.1	7	0	0	0	0	0	0	1	0	0	0	0	1	0	0	0	0	0	0	0	0	1	0	1	0	0	0	0	1	0	0	0	0	1	0	0	0	0	0	1	0	0	0	0	0	0	0	0
seedMaxEhybrid-8.seedMinPu0.001	8	0	0	0	1	0	0	0	0	1	0	0	0	0	0	0	0	0	0	0	0	0	0	0	0	0	0	0	1	0	0	0	0	1	1	0	0	0	0	1	0	0	0	1	0	0	0	1
seedMaxEhybrid-8.seedMinPu0.1.seedNoGU	5	0	0	0	0	0	0	1	0	0	0	0	0	0	0	0	0	0	1	0	0	0	1	0	0	0	0	0	1	0	0	0	0	1	0	0	0	0	0	0	0	0	0	0	0	0	0	0
seedMaxEhybrid-8.seedMinPu0.01.seedNoGU	7	0	0	0	1	0	0	1	0	0	0	0	0	0	0	0	0	0	0	0	0	0	0	0	0	0	0	0	1	0	0	0	0	1	1	0	0	0	0	0	0	0	0	1	0	0	0	1
seedMaxEhybrid-8.seedMinPu0.001.seedNoGU	8	0	0	0	1	0	0	1	0	1	0	0	0	0	0	0	0	0	0	0	0	0	0	0	0	0	0	0	1	0	0	0	0	1	1	0	0	0	0	0	0	0	0	1	0	0	0	1
seedMaxEhybrid-8.seedMinPu0.0001.seedNoGU	9	0	0	0	1	1	1	0	0	1	0	0	0	0	0	0	0	0	0	0	0	0	0	0	0	0	0	0	1	0	0	0	0	1	1	0	0	0	0	0	0	0	0	1	0	0	0	1
seedMaxEhybrid-8.seedMinPu0.2	0	0	0	0	0	0	0	0	0	0	0	0	0	0	0	0	0	0	0	0	0	0	0	0	0	0	0	0	0	0	0	0	0	0	0	0	0	0	0	0	0	0	0	0	0	0	0	0
seedMaxEhybrid-8.seedMinPu0.2.seedNoGU	0	0	0	0	0	0	0	0	0	0	0	0	0	0	0	0	0	0	0	0	0	0	0	0	0	0	0	0	0	0	0	0	0	0	0	0	0	0	0	0	0	0	0	0	0	0	0	0
seedMaxEhybrid-8.seedMinPu0.5	0	0	0	0	0	0	0	0	0	0	0	0	0	0	0	0	0	0	0	0	0	0	0	0	0	0	0	0	0	0	0	0	0	0	0	0	0	0	0	0	0	0	0	0	0	0	0	0
seedMaxEhybrid-8.seedMinPu0.5.seedNoGU	0	0	0	0	0	0	0	0	0	0	0	0	0	0	0	0	0	0	0	0	0	0	0	0	0	0	0	0	0	0	0	0	0	0	0	0	0	0	0	0	0	0	0	0	0	0	0	0
seedMaxEhybrid-8.seedMinPu0.seedNoGU	9	0	0	0	1	1	1	0	0	1	0	0	0	0	0	0	0	0	0	0	0	0	0	0	0	0	0	0	1	0	0	0	0	1	1	0	0	0	0	0	0	0	0	1	0	0	0	1
seedMaxEhybrid-8.seedNoGU	9	0	0	0	1	1	1	0	0	1	0	0	0	0	0	0	0	0	0	0	0	0	0	0	0	0	0	0	1	0	0	0	0	1	1	0	0	0	0	0	0	0	0	1	0	0	0	1
seedMaxEhybrid-9	10	0	0	0	1	1	1	0	0	1	0	0	0	0	0	0	0	0	0	0	0	0	0	0	0	0	0	0	1	0	0	0	0	1	1	0	0	0	0	1	0	0	0	1	0	0	0	1
seedMaxEhybrid-9.seedMinPu0	10	0	0	0	1	1	1	0	0	1	0	0	0	0	0	0	0	0	0	0	0	0	0	0	0	0	0	0	1	0	0	0	0	1	1	0	0	0	0	1	0	0	0	1	0	0	0	1
seedMaxEhybrid-9.seedMinPu0.01	8	0	0	0	1	0	0	1	0	0	0	0	0	0	0	0	0	0	0	0	0	0	0	0	0	0	0	0	1	0	0	0	0	1	1	0	0	0	0	1	0	0	0	1	0	0	0	1
seedMaxEhybrid-9.seedMinPu0.1	5	0	0	0	0	0	0	1	0	0	0	0	1	0	0	0	0	0	1	0	0	0	0	0	0	0	0	0	0	0	0	0	0	1	0	0	0	0	0	1	0	0	0	0	0	0	0	0
seedMaxEhybrid-9.seedMinPu0.0001	10	0	0	0	1	1	1	0	0	1	0	0	0	0	0	0	0	0	0	0	0	0	0	0	0	0	0	0	1	0	0	0	0	1	1	0	0	0	0	1	0	0	0	1	0	0	0	1
seedMaxEhybrid-9.seedMinPu0.001	7	0	0	0	1	0	0	0	0	0	0	0	0	0	0	0	0	0	0	0	0	0	0	0	0	0	0	0	1	0	0	0	0	1	1	0	0	0	0	1	0	0	0	1	0	0	0	1
seedMaxEhybrid-9.seedMinPu0.001.seedNoGU	7	0	0	0	1	0	0	1	0	0	0	0	0	0	0	0	0	0	0	0	0	0	0	0	0	0	0	0	1	0	0	0	0	1	1	0	0	0	0	0	0	0	0	1	0	0	0	1
seedMaxEhybrid-9.seedMinPu0.1.seedNoGU	3	0	0	0	0	0	0	1	0	0	0	0	0	0	0	0	0	0	1	0	0	0	0	0	0	0	0	0	0	0	0	0	0	1	0	0	0	0	0	0	0	0	0	0	0	0	0	0
seedMaxEhybrid-9.seedMinPu0.01.seedNoGU	9	0	0	0	1	0	0	1	0	0	0	0	0	0	0	0	0	0	0	0	0	0	0	0	0	0	0	0	1	0	0	0	0	1	1	0	0	0	0	0	1	0	0	1	0	0	1	1

Salmonella typhimurium NC_003197

	sum	ArcZ-STM1682	ArcZ-STM2970	ArcZ-STM3216	ChiX-STM0687	ChiX-STM1313	CyaR-STM0833	GcvB-STM0002	GcvB-STM0245	GcvB-STM0399	GcvB-STM0602	GcvB-STM0665	GcvB-STM0959	GcvB-STM1299	GcvB-STM1452	GcvB-STM1746	GcvB-STM2355	GcvB-STM2526	GcvB-STM3062	GcvB-STM3064	GcvB-STM3225	GcvB-STM3564	GcvB-STM3567	GcvB-STM3630	GcvB-STM3903	GcvB-STM3909	GcvB-STM3930	GcvB-STM4351	GcvB-STM4398	MicA-STM4231	MicC-STM1572	MicF-STM0366	MicF-STM0959	MicF-STM1328	RybB-STM0413	RybB-STM0687	RybB-STM0999	RybB-STM1070	RybB-STM1473	RybB-STM1530	RybB-STM1572	RybB-STM1732	RybB-STM1995	RybB-STM2267	RybB-STM2391	SgrS-STM2945	SgrS-STM3962	Spot42-STM2190
Target found among top-100 predictions for the sRNA for any tested parameterization	40	1	0	0	1	1	1	1	1	1	1	1	1	1	0	0	1	1	1	1	1	1	1	1	1	1	1	1	1	1	1	1	1	1	1	1	1	1	1	1	1	0	0	1	1	0	1	1
seedMaxEhybrid-9.seedMinPu0.0001.seedNoGU	11	0	0	0	1	1	1	1	0	1	0	0	0	0	0	0	0	0	0	0	0	0	0	0	0	0	0	0	1	0	0	0	0	1	1	0	1	0	0	0	0	0	0	1	0	0	0	1
seedMaxEhybrid-9.seedMinPu0.2	0	0	0	0	0	0	0	0	0	0	0	0	0	0	0	0	0	0	0	0	0	0	0	0	0	0	0	0	0	0	0	0	0	0	0	0	0	0	0	0	0	0	0	0	0	0	0	0
seedMaxEhybrid-9.seedMinPu0.2.seedNoGU	0	0	0	0	0	0	0	0	0	0	0	0	0	0	0	0	0	0	0	0	0	0	0	0	0	0	0	0	0	0	0	0	0	0	0	0	0	0	0	0	0	0	0	0	0	0	0	0
seedMaxEhybrid-9.seedMinPu0.5	0	0	0	0	0	0	0	0	0	0	0	0	0	0	0	0	0	0	0	0	0	0	0	0	0	0	0	0	0	0	0	0	0	0	0	0	0	0	0	0	0	0	0	0	0	0	0	0
seedMaxEhybrid-9.seedMinPu0.5.seedNoGU	0	0	0	0	0	0	0	0	0	0	0	0	0	0	0	0	0	0	0	0	0	0	0	0	0	0	0	0	0	0	0	0	0	0	0	0	0	0	0	0	0	0	0	0	0	0	0	0
seedMaxEhybrid-9.seedMinPu0.seedNoGU	11	0	0	0	1	1	1	1	0	1	0	0	0	0	0	0	0	0	0	0	0	0	0	0	0	0	0	0	1	0	0	0	0	1	1	0	1	0	0	0	0	0	0	1	0	0	0	1
seedMaxEhybrid-9.seedNoGU	11	0	0	0	1	1	1	1	0	1	0	0	0	0	0	0	0	0	0	0	0	0	0	0	0	0	0	0	1	0	0	0	0	1	1	0	1	0	0	0	0	0	0	1	0	0	0	1
seedMaxEhybrid-10	8	0	0	0	0	1	1	0	0	1	0	0	0	0	0	0	0	0	0	0	0	0	0	0	0	0	0	0	1	0	0	0	0	1	1	0	0	0	0	0	0	0	0	1	0	0	0	1
seedMaxEhybrid-10.seedMinPu0	8	0	0	0	0	1	1	0	0	1	0	0	0	0	0	0	0	0	0	0	0	0	0	0	0	0	0	0	1	0	0	0	0	1	1	0	0	0	0	0	0	0	0	1	0	0	0	1
seedMaxEhybrid-10.seedMinPu0.1	2	0	0	0	0	0	0	0	0	0	0	0	0	0	0	0	0	0	1	0	0	0	0	0	0	0	0	0	0	0	0	0	0	1	0	0	0	0	0	0	0	0	0	0	0	0	0	0
seedMaxEhybrid-10.seedMinPu0.01	4	0	0	0	0	0	0	0	0	0	0	0	0	0	0	0	0	0	0	0	0	0	0	0	0	0	0	0	1	0	0	0	0	1	1	0	0	0	0	0	0	0	0	0	0	0	0	1
seedMaxEhybrid-10.seedMinPu0.0001	7	0	0	0	0	0	1	0	0	1	0	0	0	0	0	0	0	0	0	0	0	0	0	0	0	0	0	0	1	0	0	0	0	1	1	0	0	0	0	0	0	0	0	1	0	0	0	1
seedMaxEhybrid-10.seedMinPu0.001	5	0	0	0	0	0	0	0	0	0	0	0	0	0	0	0	0	0	0	0	0	0	0	0	0	0	0	0	1	0	0	0	0	1	1	0	0	0	0	0	0	0	0	1	0	0	0	1
seedMaxEhybrid-10.seedMinPu0.001.seedNoGU	6	0	0	0	0	0	0	0	0	0	0	0	0	0	0	0	0	0	0	0	0	0	0	0	0	0	0	0	1	0	0	0	0	1	1	0	0	0	0	0	0	0	0	1	1	0	0	1
seedMaxEhybrid-10.seedMinPu0.0001.seedNoGU	11	0	0	0	0	0	1	0	0	1	0	0	0	0	0	0	0	0	0	1	0	0	0	0	0	0	0	0	1	0	0	0	0	1	1	0	1	1	0	0	0	0	0	1	1	0	0	1
seedMaxEhybrid-10.seedMinPu0.01.seedNoGU	5	0	0	0	0	0	0	0	0	0	0	0	0	0	0	0	0	0	0	0	0	0	0	0	0	0	0	0	1	0	0	0	0	1	1	0	0	0	0	0	1	0	0	0	0	0	0	1
seedMaxEhybrid-10.seedMinPu0.1.seedNoGU	2	0	0	0	0	0	0	0	0	0	0	0	0	0	0	0	0	0	1	0	0	0	0	0	0	0	0	0	0	0	0	0	0	1	0	0	0	0	0	0	0	0	0	0	0	0	0	0
seedMaxEhybrid-10.seedMinPu0.2	0	0	0	0	0	0	0	0	0	0	0	0	0	0	0	0	0	0	0	0	0	0	0	0	0	0	0	0	0	0	0	0	0	0	0	0	0	0	0	0	0	0	0	0	0	0	0	0
seedMaxEhybrid-10.seedMinPu0.2.seedNoGU	0	0	0	0	0	0	0	0	0	0	0	0	0	0	0	0	0	0	0	0	0	0	0	0	0	0	0	0	0	0	0	0	0	0	0	0	0	0	0	0	0	0	0	0	0	0	0	0
seedMaxEhybrid-10.seedMinPu0.5	0	0	0	0	0	0	0	0	0	0	0	0	0	0	0	0	0	0	0	0	0	0	0	0	0	0	0	0	0	0	0	0	0	0	0	0	0	0	0	0	0	0	0	0	0	0	0	0
seedMaxEhybrid-10.seedMinPu0.5.seedNoGU	0	0	0	0	0	0	0	0	0	0	0	0	0	0	0	0	0	0	0	0	0	0	0	0	0	0	0	0	0	0	0	0	0	0	0	0	0	0	0	0	0	0	0	0	0	0	0	0
seedMaxEhybrid-10.seedMinPu0.seedNoGU	10	0	0	0	0	1	1	0	0	1	0	0	0	0	0	0	0	0	0	0	0	0	0	0	0	0	0	0	1	0	0	0	0	1	1	0	1	0	0	0	0	0	0	1	1	0	0	1
seedMaxEhybrid-10.seedNoGU	10	0	0	0	0	1	1	0	0	1	0	0	0	0	0	0	0	0	0	0	0	0	0	0	0	0	0	0	1	0	0	0	0	1	1	0	1	0	0	0	0	0	0	1	1	0	0	1

id	sum	ArcZ-b1892	ArcZ-b2741	ArcZ-b3546	ChiX-b0619	ChiX-b0681	ChiX-b1737	CyaR-b0723	CyaR-b0814	CyaR-b1740	CyaR-b1824	CyaR-b2416	CyaR-b2666	CyaR-b2687	DsrA-b1237	DsrA-b2741	DsrA-b3251	FnrS-b0723	FnrS-b0755	FnrS-b0887	FnrS-b1107	FnrS-b1479	FnrS-b1531	FnrS-b1656	FnrS-b1841	FnrS-b2153	FnrS-b2303	FnrS-b2531	FnrS-b3829	FnrS-b3908	GcvB-b1040	GcvB-b1130	GcvB-b3089	GcvB-b4208	MicA-b0411	MicA-b0814	MicA-b0957	MicA-b1130	MicC-b2215	MicF-b0241	MicF-b0889	MicF-b0929
seedBP4.seedNoGU_IntaRNA	51	0	0	0	1	1	1	0	1	0	1	1	0	0	1	1	0	1	0	0	0	0	1	0	1	0	0	1	1	0	0	0	0	0	0	0	1	1	1	0	1	1
seedBP5.seedNoGU_IntaRNA	55	0	0	0	1	1	1	0	1	0	1	1	0	0	1	1	0	1	0	0	0	0	1	1	1	0	0	1	1	0	0	0	0	0	0	0	1	1	1	0	1	1
seedBP6.seedNoGU_IntaRNA	57	0	0	0	1	1	1	0	1	0	1	1	0	0	1	1	0	0	0	0	0	0	1	1	1	0	0	1	1	0	0	0	0	0	0	0	1	1	1	0	1	1
seedBP7.seedNoGU_IntaRNA	64	0	0	0	1	1	1	0	1	0	1	1	0	0	1	1	0	0	0	0	0	0	1	0	1	0	0	1	1	0	0	0	0	1	0	1	1	1	1	1	0	1
seedBP8.seedNoGU_IntaRNA	65	0	0	0	1	1	1	0	1	0	0	1	0	0	1	1	0	0	0	1	0	1	1	0	1	0	0	1	1	0	0	1	1	1	0	0	1	1	1	1	0	1
seedBP9.seedNoGU_IntaRNA	48	0	1	0	1	1	1	0	1	0	0	0	0	0	1	1	0	0	0	0	0	1	0	0	1	0	0	1	0	0	0	0	1	1	0	0	1	1	1	1	0	1
seedBP10.seedNoGU_IntaRNA	40	0	1	0	1	1	1	0	0	0	0	0	0	0	1	1	0	0	0	0	0	1	0	0	0	0	0	1	0	0	0	0	0	1	0	0	0	1	1	1	0	1
seedBP11.seedNoGU_IntaRNA	22	0	0	0	1	1	1	0	0	0	0	0	0	0	1	1	0	0	0	0	0	1	0	0	0	0	0	1	0	0	0	0	0	0	0	0	0	0	1	1	0	1
seedBP12.seedNoGU_IntaRNA	17	0	0	0	1	1	1	0	0	0	0	0	0	0	1	1	0	0	0	0	0	0	0	0	0	0	0	1	0	0	0	0	0	0	0	0	0	0	1	1	0	1
seedBP13.seedNoGU_IntaRNA	3	0	0	0	0	0	0	0	0	0	0	0	0	0	1	0	0	0	0	0	0	0	0	0	0	0	0	0	0	0	0	0	0	0	0	0	0	0	1	0	0	0
seedBP14.seedNoGU_IntaRNA	1	0	0	0	0	0	0	0	0	0	0	0	0	0	0	0	0	0	0	0	0	0	0	0	0	0	0	0	0	0	0	0	0	0	0	0	0	0	1	0	0	0
seedBP4_IntaRNA	51	0	0	0	1	1	1	0	1	0	1	1	0	0	1	1	0	1	0	0	0	0	1	0	1	0	0	1	1	0	0	0	0	0	0	0	1	1	1	0	1	1
seedBP5_IntaRNA	50	0	0	0	1	1	1	0	1	0	1	1	0	0	1	1	0	1	0	0	0	0	1	0	1	0	0	1	1	0	0	0	0	0	0	0	1	1	1	0	1	1
seedBP6_IntaRNA	51	0	0	0	1	1	1	0	1	0	1	1	0	0	1	1	0	1	0	0	0	0	1	0	1	0	0	1	1	0	0	0	0	0	0	0	1	1	1	0	1	1
seedBP7_IntaRNA	52	0	0	0	1	1	1	0	1	0	1	1	0	0	1	1	0	1	0	0	0	0	1	0	1	0	0	1	1	0	0	0	0	0	0	0	1	1	1	0	1	1
seedBP8_IntaRNA	54	0	0	0	1	1	1	0	1	0	1	1	0	0	1	1	0	1	0	0	0	0	1	1	1	0	0	1	1	0	0	0	0	0	0	0	1	1	1	0	1	1
seedBP9_IntaRNA	50	0	0	0	1	1	1	0	1	0	1	1	0	0	1	1	0	0	0	0	0	0	0	0	1	0	0	1	0	0	0	0	0	1	0	0	1	1	1	0	1	1
seedBP10_IntaRNA	45	0	0	0	1	1	1	0	0	0	0	0	0	0	1	1	0	0	0	0	0	0	0	0	1	0	0	1	0	0	0	0	0	1	0	0	1	1	1	1	1	1
seedBP11_IntaRNA	41	0	0	0	1	1	1	0	0	0	0	0	0	0	1	1	0	0	0	0	0	1	0	0	1	0	0	1	0	0	0	0	0	0	1	0	1	0	1	1	0	1
seedBP12_IntaRNA	34	0	0	0	1	1	1	0	0	0	0	0	0	0	1	1	0	0	0	0	0	0	0	0	0	0	0	1	0	0	0	0	0	0	0	0	1	0	1	1	0	1
seedBP13_IntaRNA	19	0	0	0	1	0	0	0	0	0	0	0	0	0	1	1	0	0	0	0	0	0	0	0	0	0	0	1	0	0	0	0	1	0	0	0	0	0	1	1	0	0
seedBP14_IntaRNA	11	0	0	0	0	0	0	0	0	0	0	0	0	0	0	0	0	1	0	0	0	0	0	0	0	0	0	1	0	0	0	0	1	0	0	0	0	0	1	0	0	0
seedBP4.intLenMax20_IntaRNA	53	0	0	0	1	1	1	0	0	0	0	1	0	0	1	0	0	0	0	1	0	0	0	0	1	0	0	1	0	0	0	0	0	0	0	0	1	1	1	0	1	1
seedBP5.intLenMax20_IntaRNA	51	0	0	0	1	1	1	0	0	0	0	1	0	0	1	0	0	0	0	1	0	0	0	0	1	0	0	1	0	0	0	0	0	0	0	0	1	1	1	0	1	1
seedBP6.intLenMax20_IntaRNA	51	0	0	0	1	1	1	0	0	0	0	1	0	0	1	0	0	0	0	1	0	0	0	0	1	0	0	1	0	0	0	0	0	0	0	0	1	1	1	0	1	1
seedBP7.intLenMax20_IntaRNA	51	0	0	0	1	1	1	0	0	0	0	1	0	0	1	1	0	0	0	1	0	0	0	0	1	0	0	1	0	0	0	0	0	0	0	0	1	1	1	0	1	1
seedBP8.intLenMax20_IntaRNA	51	0	0	0	1	1	1	0	0	0	0	1	0	0	1	1	0	0	0	1	0	0	0	0	1	0	0	1	0	0	0	0	0	0	0	0	1	1	1	0	1	1
seedBP9.intLenMax20_IntaRNA	45	0	0	0	1	1	1	0	0	0	0	0	0	0	1	0	0	0	0	0	0	0	0	0	1	0	0	1	0	0	0	0	0	0	0	0	1	1	1	1	1	1
seedBP10.intLenMax20_IntaRNA	40	0	0	0	1	1	1	0	0	0	0	0	0	0	1	0	0	0	0	0	0	0	0	0	1	0	0	1	0	0	0	0	0	0	0	0	1	1	1	1	0	0
seedBP11.intLenMax20_IntaRNA	37	0	0	0	1	1	1	0	0	0	0	0	0	0	1	1	0	0	0	0	0	0	0	0	1	0	0	1	0	0	0	0	0	0	1	0	1	0	1	1	0	0
seedBP12.intLenMax20_IntaRNA	30	0	0	0	1	1	0	0	0	0	0	0	0	0	1	0	0	0	0	0	0	0	0	0	0	0	0	0	0	0	0	0	0	0	0	0	1	0	1	1	0	0
seedBP13.intLenMax20_IntaRNA	16	0	0	0	1	0	0	0	0	0	0	0	0	0	1	0	0	0	0	0	0	0	0	0	0	0	0	0	0	0	0	0	0	0	0	0	0	0	1	1	0	0
seedBP14.intLenMax20_IntaRNA	8	0	0	0	0	0	0	0	0	0	0	0	0	0	0	0	0	1	0	0	0	0	0	0	0	0	0	0	0	0	1	0	0	0	0	0	0	0	1	0	0	0
seedBP7_TargetRNA2	26	0	0	0	1	1	1	0	0	1	0	0	0	1	1	1	0	0	0	0	0	0	0	0	0	0	0	0	0	0	0	0	0	0	0	0	1	0	1	1	0	1
seedBP8_TargetRNA2	28	0	0	0	1	1	1	0	0	0	0	0	0	0	0	0	0	0	0	0	0	0	0	0	0	0	0	0	0	0	0	0	0	0	0	0	1	0	1	1	0	1
seedBP9_TargetRNA2	39	0	1	0	1	1	1	0	0	1	0	0	0	0	1	1	0	0	0	0	0	0	0	0	0	0	0	0	0	0	0	0	0	1	0	0	1	0	1	1	0	1
seedBP10_TargetRNA2	40	0	1	0	1	1	1	0	0	0	0	0	0	0	1	1	0	0	0	0	0	0	0	0	0	0	0	0	0	0	0	0	1	1	0	0	0	1	1	1	0	1
seedBP11_TargetRNA2	22	0	0	0	1	1	1	0	0	0	0	0	0	0	1	1	0	0	0	0	0	1	0	0	0	0	0	1	0	0	0	0	0	0	0	0	0	0	1	1	0	1
seedBP12_TargetRNA2	17	0	0	0	1	1	1	0	0	0	0	0	0	0	1	1	0	0	0	0	0	0	0	0	0	0	0	1	0	0	0	0	0	0	0	0	0	0	1	1	0	1
seedBP4.seedNoGU_RIsearch2	6	0	0	0	0	0	0	0	0	0	0	0	0	0	0	1	0	0	0	0	0	0	0	0	0	0	0	1	0	0	0	0	0	0	0	0	0	0	0	0	0	0
seedBP5.seedNoGU_RIsearch2	6	0	0	0	0	0	0	0	0	0	0	0	0	0	0	1	0	0	0	0	0	0	0	0	0	0	0	1	0	0	0	0	0	0	0	0	0	0	0	0	0	0
seedBP6.seedNoGU_RIsearch2	9	1	0	0	0	0	1	0	0	0	0	0	0	0	0	1	0	0	0	0	0	0	0	0	0	0	0	1	0	0	0	0	0	0	0	0	0	0	0	0	0	0
seedBP7.seedNoGU_RIsearch2	14	1	0	0	0	0	1	0	0	0	0	0	0	0	1	1	0	0	0	0	0	0	0	0	0	0	0	1	0	0	0	0	0	1	0	0	0	0	0	0	0	0
seedBP8.seedNoGU_RIsearch2	16	1	0	0	0	0	1	0	0	0	0	0	0	0	1	1	0	0	0	0	0	0	0	0	0	0	0	1	0	0	0	0	0	1	0	0	0	1	0	0	0	0
seedBP9.seedNoGU_RIsearch2	25	1	0	0	1	1	1	0	0	0	0	0	0	0	1	1	0	0	0	0	0	0	0	0	1	0	0	1	0	0	0	0	0	1	0	0	1	1	0	0	0	1
seedBP10.seedNoGU_RIsearch2	31	0	0	0	1	1	1	0	0	0	0	0	0	0	1	1	0	0	0	0	0	1	0	0	0	0	0	1	0	0	0	0	0	1	0	0	0	1	1	0	0	1
seedBP11.seedNoGU_RIsearch2	20	0	0	0	1	1	1	0	0	0	0	0	0	0	1	1	0	0	0	0	0	1	0	0	0	0	0	1	0	0	0	0	0	0	0	0	0	0	1	0	0	1
seedBP12.seedNoGU_RIsearch2	16	0	0	0	1	1	1	0	0	0	0	0	0	0	1	1	0	0	0	0	0	0	0	0	0	0	0	1	0	0	0	0	0	0	0	0	0	0	1	0	0	1
seedBP13.seedNoGU_RIsearch2	3	0	0	0	0	0	0	0	0	0	0	0	0	0	1	0	0	0	0	0	0	0	0	0	0	0	0	0	0	0	0	0	0	0	0	0	0	0	1	0	0	0
seedBP14.seedNoGU_RIsearch2	1	0	0	0	0	0	0	0	0	0	0	0	0	0	0	0	0	0	0	0	0	0	0	0	0	0	0	0	0	0	0	0	0	0	0	0	0	0	1	0	0	0
seedBP4_RIsearch2	7	0	0	0	0	0	0	0	0	0	0	0	0	0	0	1	0	0	0	0	0	0	0	0	0	0	0	1	0	0	0	0	0	0	0	0	0	0	0	0	0	0
seedBP5_RIsearch2	9	0	0	0	0	0	0	0	0	0	0	0	0	0	0	1	0	0	0	0	0	0	0	0	0	0	0	1	0	0	0	0	0	0	0	0	0	0	0	0	0	0
seedBP6_RIsearch2	7	0	0	0	0	0	0	0	0	0	0	0	0	0	0	1	0	0	0	0	0	0	0	0	0	0	0	1	0	0	0	0	0	0	0	0	0	0	0	0	0	0
seedBP7_RIsearch2	7	0	0	0	0	0	0	0	0	0	0	0	0	0	0	1	0	0	0	0	0	0	0	0	0	0	0	1	0	0	0	0	0	0	0	0	0	0	0	0	0	0
seedBP8_RIsearch2	8	0	0	0	0	0	0	0	0	0	0	0	0	0	0	1	0	0	0	0	0	0	0	0	0	0	0	1	0	0	0	0	0	0	0	0	0	0	0	0	0	0
seedBP9_RIsearch2	10	1	0	0	0	0	1	0	0	0	0	0	0	0	0	1	0	0	0	0	0	0	0	0	0	0	0	1	0	0	0	0	0	0	0	0	0	0	1	0	0	0
seedBP10_RIsearch2	9	0	0	0	0	0	1	0	0	0	0	0	0	0	1	1	0	0	0	0	0	0	0	0	0	0	0	1	0	0	0	0	0	0	0	0	0	0	1	0	0	0
seedBP11_RIsearch2	9	0	0	0	0	0	1	0	0	0	0	0	0	0	1	1	0	0	0	0	0	0	0	0	1	0	0	1	0	0	0	0	0	0	0	0	0	0	0	0	0	0
seedBP12_RIsearch2	12	0	0	0	0	0	1	0	0	0	0	0	0	0	1	1	0	0	0	0	0	0	0	0	0	0	0	1	0	0	0	0	0	0	0	0	1	0	0	0	0	1
seedBP13_RIsearch2	13	0	0	0	1	0	0	0	0	0	0	0	0	0	1	1	0	0	0	0	0	0	0	0	0	0	0	1	0	0	0	0	0	0	0	0	0	0	0	0	0	0
seedBP14_RIsearch2	9	0	0	0	0	0	0	0	0	0	0	0	0	0	0	0	0	1	0	0	0	0	0	0	0	0	0	1	0	0	0	0	0	0	0	0	0	0	1	0	0	0
outMaxE0_RIsearch2	7	0	0	0	0	0	0	0	0	0	0	0	0	0	0	1	0	0	0	0	0	0	0	0	0	0	0	1	0	0	0	0	0	0	0	0	0	0	0	0	0	0
outMaxE20_RIsearch2	7	0	0	0	0	0	0	0	0	0	0	0	0	0	0	1	0	0	0	0	0	0	0	0	0	0	0	1	0	0	0	0	0	0	0	0	0	0	0	0	0	0

id	MicF-b3912	OxyS-b1892	OxyS-b2731	RprA-b1040	RprA-b1341	RprA-b2741	RybB-b0081	RybB-b0721	RybB-b0805	RybB-b1256	RybB-b2215	RybB-b2594	RyhB-b0118	RyhB-b0156	RyhB-b0288	RyhB-b0592	RyhB-b0683	RyhB-b0721	RyhB-b0723	RyhB-b0894	RyhB-b1107	RyhB-b1200	RyhB-b1452	RyhB-b1531	RyhB-b1588	RyhB-b1612	RyhB-b1656	RyhB-b1778	RyhB-b1981	RyhB-b2069	RyhB-b2155	RyhB-b2206	RyhB-b2530	RyhB-b3365	RyhB-b3607	RyhB-b3942	RyhB-b4070	RyhB-b4122
seedBP4.seedNoGU_IntaRNA	0	0	0	1	0	1	1	0	0	0	0	0	0	0	0	0	0	0	0	1	1	0	0	0	0	0	0	0	0	0	0	0	0	1	1	0	0	0
seedBP5.seedNoGU_IntaRNA	0	0	0	1	0	1	1	0	0	0	1	0	0	0	0	0	0	0	0	1	1	0	0	0	0	0	0	0	0	0	0	0	0	1	1	0	0	0
seedBP6.seedNoGU_IntaRNA	0	0	0	1	0	1	0	0	0	0	1	0	0	0	0	0	0	0	0	1	1	0	0	0	0	0	0	0	0	0	0	0	0	1	1	0	0	0
seedBP7.seedNoGU_IntaRNA	0	0	0	1	0	1	0	0	1	0	1	0	0	1	0	1	0	1	0	1	0	0	0	0	1	0	0	0	0	0	1	1	0	1	1	0	0	0
seedBP8.seedNoGU_IntaRNA	0	0	0	1	0	1	0	0	0	0	1	0	0	1	0	1	0	1	0	1	0	0	0	0	0	0	0	1	0	0	1	1	1	0	1	0	0	1
seedBP9.seedNoGU_IntaRNA	0	0	0	1	0	0	0	0	0	0	0	0	0	1	0	1	0	1	0	0	0	0	0	0	0	0	0	1	1	0	0	0	0	0	1	0	0	0
seedBP10.seedNoGU_IntaRNA	0	0	0	1	0	0	0	0	0	0	0	0	0	0	0	1	0	0	0	0	0	0	0	0	0	0	0	1	1	0	0	0	0	0	1	0	1	0
seedBP11.seedNoGU_IntaRNA	0	0	0	1	0	0	0	0	0	0	0	0	0	0	0	0	0	0	0	0	0	0	0	0	0	0	0	0	0	0	0	0	0	0	1	0	0	0
seedBP12.seedNoGU_IntaRNA	0	0	0	1	0	0	0	0	0	0	0	0	0	0	0	0	0	0	0	0	0	0	0	0	0	0	0	0	0	0	0	0	0	0	1	0	0	0
seedBP13.seedNoGU_IntaRNA	0	0	0	1	0	0	0	0	0	0	0	0	0	0	0	0	0	0	0	0	0	0	0	0	0	0	0	0	0	0	0	0	0	0	0	0	0	0
seedBP14.seedNoGU_IntaRNA	0	0	0	0	0	0	0	0	0	0	0	0	0	0	0	0	0	0	0	0	0	0	0	0	0	0	0	0	0	0	0	0	0	0	0	0	0	0
seedBP4_IntaRNA	0	0	0	1	0	1	1	0	0	0	0	0	0	0	0	0	0	0	0	1	1	0	0	0	0	0	0	0	0	0	0	0	0	1	1	0	0	0
seedBP5_IntaRNA	0	0	0	1	0	1	1	0	0	0	0	0	0	0	0	0	0	0	0	1	1	0	0	0	0	0	0	0	0	0	0	0	0	1	1	0	0	0
seedBP6_IntaRNA	0	0	0	1	0	1	1	0	0	0	0	0	0	0	0	0	0	0	0	1	1	0	0	0	0	0	0	0	0	0	0	0	0	1	1	0	0	0
seedBP7_IntaRNA	0	0	0	1	0	1	1	0	0	0	0	0	0	0	0	0	0	0	0	1	1	0	0	0	0	0	0	0	0	0	0	0	0	1	1	0	0	0
seedBP8_IntaRNA	0	0	0	1	0	1	1	0	0	0	1	0	0	0	0	0	0	0	0	1	1	0	0	0	0	0	0	0	0	0	0	0	0	0	1	0	0	0
seedBP9_IntaRNA	0	0	0	1	0	0	1	0	0	0	0	0	0	1	0	0	0	1	0	0	1	0	0	0	0	0	0	0	0	0	1	0	0	0	1	0	0	0
seedBP10_IntaRNA	0	0	0	1	0	0	1	0	0	0	0	0	0	0	0	0	0	1	0	0	1	0	0	0	1	0	0	0	0	0	1	0	0	0	1	0	0	0
seedBP11_IntaRNA	1	0	0	1	1	0	0	0	0	0	0	0	0	0	0	0	0	1	0	0	1	0	0	0	0	0	0	1	0	0	1	0	0	0	1	0	0	0
seedBP12_IntaRNA	1	0	0	1	0	0	0	0	0	0	0	0	0	0	0	0	0	1	0	0	1	0	0	0	0	0	0	1	1	0	1	0	0	0	1	0	0	0
seedBP13_IntaRNA	0	0	0	1	0	0	0	0	0	0	0	0	0	0	0	0	0	1	0	0	1	0	0	0	0	0	0	0	0	0	0	0	0	0	1	0	0	0
seedBP14_IntaRNA	0	0	0	1	0	0	0	0	0	0	0	0	0	0	0	0	0	0	0	0	0	0	0	0	0	0	0	0	0	0	0	0	0	0	0	0	0	0
seedBP4.intLenMax20_IntaRNA	0	0	0	1	0	0	0	0	0	1	0	0	0	0	0	0	0	1	0	1	1	0	1	0	1	0	0	1	0	0	1	1	0	1	1	0	0	1
seedBP5.intLenMax20_IntaRNA	0	0	0	1	0	0	0	0	0	1	0	0	0	0	0	0	0	1	0	1	1	0	1	0	1	0	0	1	0	0	1	0	0	1	1	0	0	1
seedBP6.intLenMax20_IntaRNA	0	0	0	1	0	0	0	0	0	1	0	0	0	0	0	0	0	1	0	1	1	0	1	0	1	0	0	1	0	0	1	0	0	1	1	0	0	1
seedBP7.intLenMax20_IntaRNA	0	0	0	1	0	0	0	0	0	1	0	0	0	0	0	0	0	1	0	1	1	0	0	0	1	0	0	1	0	0	1	0	0	1	1	0	0	1
seedBP8.intLenMax20_IntaRNA	1	0	0	1	0	0	0	0	0	0	0	0	0	0	0	0	0	1	0	1	1	0	0	0	1	0	0	1	0	0	1	0	0	0	1	0	0	1
seedBP9.intLenMax20_IntaRNA	1	0	0	1	0	0	0	0	0	0	0	0	0	0	0	0	0	1	0	0	1	0	0	0	1	0	0	1	0	0	1	0	0	0	1	0	0	1
seedBP10.intLenMax20_IntaRNA	1	0	0	1	0	0	0	0	0	0	0	0	0	0	0	0	0	1	0	0	0	0	0	0	1	0	0	1	1	0	1	0	0	0	1	0	0	0
seedBP11.intLenMax20_IntaRNA	1	0	0	1	1	0	0	0	0	0	0	0	0	0	0	0	0	1	0	0	0	0	0	0	0	0	0	1	1	0	1	0	0	0	1	0	0	0
seedBP12.intLenMax20_IntaRNA	1	0	0	1	0	0	0	0	0	0	0	0	0	0	0	0	0	1	0	0	0	0	0	0	0	0	0	1	1	0	1	0	0	0	1	0	0	0
seedBP13.intLenMax20_IntaRNA	0	0	0	1	0	0	0	0	0	0	0	0	0	0	0	0	0	1	0	0	0	0	0	0	0	0	0	0	0	0	0	0	0	0	1	0	0	0
seedBP14.intLenMax20_IntaRNA	0	0	0	1	0	0	0	0	0	0	0	0	0	0	0	0	0	0	0	0	0	0	0	0	0	0	0	0	0	0	0	0	0	0	0	0	0	0
seedBP7_TargetRNA2	0	0	0	1	0	0	0	0	0	0	0	0	0	0	0	0	0	1	0	0	0	0	0	0	0	0	0	0	0	0	0	0	1	0	0	0	0	0
seedBP8_TargetRNA2	0	0	0	1	0	0	0	0	0	0	0	0	0	0	0	0	0	1	0	0	0	0	0	0	0	0	0	1	1	0	1	0	0	0	1	0	0	0
seedBP9_TargetRNA2	0	0	0	1	0	0	0	0	0	0	0	0	0	1	0	1	0	1	0	0	0	0	0	0	0	0	1	1	1	0	0	0	0	0	1	0	0	0
seedBP10_TargetRNA2	0	0	0	1	0	0	0	0	0	0	0	0	0	0	0	1	0	0	0	0	0	0	0	0	0	0	0	1	1	0	0	0	0	0	1	0	1	0
seedBP11_TargetRNA2	0	0	0	1	0	0	0	0	0	0	0	0	0	0	0	0	0	0	0	0	0	0	0	0	0	0	0	0	0	0	0	0	0	0	1	0	0	0
seedBP12_TargetRNA2	0	0	0	1	0	0	0	0	0	0	0	0	0	0	0	0	0	0	0	0	0	0	0	0	0	0	0	0	0	0	0	0	0	0	1	0	0	0
seedBP4.seedNoGU_RIsearch2	0	0	0	1	0	0	0	0	0	0	0	0	0	0	0	0	0	0	0	0	0	0	0	0	0	0	0	0	0	0	0	0	0	0	0	0	0	0
seedBP5.seedNoGU_RIsearch2	0	0	0	1	0	0	0	0	0	0	0	0	0	0	0	0	0	0	0	0	0	0	0	0	0	0	0	0	0	0	0	0	0	0	0	0	0	0
seedBP6.seedNoGU_RIsearch2	0	0	0	1	0	0	0	0	0	0	0	0	0	0	0	0	0	0	0	0	0	0	0	0	0	0	0	0	0	0	0	0	0	0	0	0	0	0
seedBP7.seedNoGU_RIsearch2	0	0	0	1	0	0	0	0	0	0	0	0	0	0	0	0	0	0	0	0	0	0	0	0	0	0	0	0	0	0	0	0	0	0	0	0	0	0
seedBP8.seedNoGU_RIsearch2	0	0	0	1	0	0	0	0	0	0	0	0	0	0	0	0	0	0	0	1	0	0	0	0	0	0	0	0	0	0	0	1	0	0	0	0	0	0
seedBP9.seedNoGU_RIsearch2	0	0	0	1	0	0	0	0	0	0	0	0	0	0	0	0	0	0	0	0	0	0	0	0	0	0	0	0	0	0	0	0	0	0	0	0	0	0
seedBP10.seedNoGU_RIsearch2	0	0	0	1	0	0	0	0	0	0	0	0	0	0	0	1	0	0	0	0	0	0	0	0	0	0	0	0	0	0	0	0	0	0	1	0	1	0
seedBP11.seedNoGU_RIsearch2	0	0	0	1	0	0	0	0	0	0	0	0	0	0	0	0	0	0	0	0	0	0	0	0	0	0	0	0	0	0	0	0	0	0	1	0	0	0
seedBP12.seedNoGU_RIsearch2	0	0	0	1	0	0	0	0	0	0	0	0	0	0	0	0	0	0	0	0	0	0	0	0	0	0	0	0	0	0	0	0	0	0	1	0	0	0
seedBP13.seedNoGU_RIsearch2	0	0	0	1	0	0	0	0	0	0	0	0	0	0	0	0	0	0	0	0	0	0	0	0	0	0	0	0	0	0	0	0	0	0	0	0	0	0
seedBP14.seedNoGU_RIsearch2	0	0	0	0	0	0	0	0	0	0	0	0	0	0	0	0	0	0	0	0	0	0	0	0	0	0	0	0	0	0	0	0	0	0	0	0	0	0
seedBP4_RIsearch2	0	0	0	1	0	0	0	0	0	0	0	0	0	0	0	0	0	0	0	0	0	0	0	0	0	0	0	0	0	0	0	0	0	0	0	0	0	1
seedBP5_RIsearch2	0	0	0	1	0	0	0	0	0	0	0	0	0	0	0	0	0	0	0	0	0	0	0	0	0	0	0	0	0	0	0	1	0	0	0	0	0	1
seedBP6_RIsearch2	0	0	0	1	0	0	0	0	0	0	0	0	0	0	0	0	0	0	0	0	0	0	0	0	0	0	0	0	0	0	0	0	0	0	0	0	0	0
seedBP7_RIsearch2	0	0	0	1	0	0	1	0	0	0	0	0	0	0	0	0	0	0	0	0	0	0	0	0	0	0	0	0	0	0	0	0	0	0	0	0	0	0
seedBP8_RIsearch2	0	0	0	1	0	0	1	0	0	0	0	0	0	0	0	0	0	0	0	0	0	0	0	0	0	0	0	0	0	0	0	0	0	0	0	0	0	0
seedBP9_RIsearch2	0	0	0	1	0	0	1	0	0	0	0	0	0	0	0	0	0	0	0	0	0	0	0	0	0	0	0	0	0	0	0	0	0	0	0	0	0	0
seedBP10_RIsearch2	0	0	0	1	0	0	1	0	0	0	0	0	0	0	0	0	0	0	0	0	0	0	0	0	0	0	0	0	0	0	0	0	0	0	0	0	0	0
seedBP11_RIsearch2	0	0	0	1	0	0	0	0	0	0	0	0	0	0	0	0	0	0	0	0	0	0	0	0	0	0	0	0	0	0	0	0	0	0	0	0	0	0
seedBP12_RIsearch2	0	0	0	1	0	0	0	0	0	0	0	0	0	0	0	0	0	0	0	0	0	0	0	0	0	0	0	0	0	0	0	0	0	0	0	0	0	0
seedBP13_RIsearch2	0	0	0	1	0	0	0	1	0	0	0	0	0	0	0	0	0	1	0	0	1	0	0	0	0	0	0	0	0	0	0	0	0	0	1	0	0	0
seedBP14_RIsearch2	0	0	0	1	0	0	0	1	0	0	0	0	0	0	0	0	0	0	0	0	0	0	0	0	0	0	0	0	0	0	0	0	0	0	0	0	0	0
outMaxE0_RIsearch2	0	0	0	1	0	0	0	0	0	0	0	0	0	0	0	0	0	0	0	0	0	0	0	0	0	0	0	0	0	0	0	0	0	0	0	0	0	0
outMaxE20_RIsearch2	0	0	0	1	0	0	0	0	0	0	0	0	0	0	0	0	0	0	0	0	0	0	0	0	0	0	0	0	0	0	0	0	0	0	0	0	0	0

id	SgrS-b1101	SgrS-b1817	SgrS-b2416	Spot42-b0039	Spot42-b0720	Spot42-b0721	Spot42-b0728	Spot42-b0757	Spot42-b1136	Spot42-b1302	Spot42-b1398	Spot42-b1761	Spot42-b1901	Spot42-b2221	Spot42-b2702	Spot42-b2715	Spot42-b2801	Spot42-b2802	Spot42-b3224	Spot42-b3566	Spot42-b3927	Spot42-b3962	Spot42-b4311
seedBP4.seedNoGU_IntaRNA	1	0	1	0	0	0	0	0	1	1	1	1	0	0	0	0	1	0	1	0	1	0	1
seedBP5.seedNoGU_IntaRNA	1	0	1	0	0	0	0	0	1	1	1	1	0	0	0	0	1	0	1	0	1	0	1
seedBP6.seedNoGU_IntaRNA	1	0	1	0	0	1	0	1	1	1	1	1	0	1	0	0	1	0	1	0	1	0	1
seedBP7.seedNoGU_IntaRNA	1	0	1	0	0	1	1	1	1	1	1	1	0	1	0	1	1	0	1	1	1	0	0
seedBP8.seedNoGU_IntaRNA	1	0	1	0	1	1	0	1	1	1	1	1	1	1	0	1	1	0	1	0	1	0	0
seedBP9.seedNoGU_IntaRNA	0	0	1	0	1	0	0	1	1	0	1	1	0	0	0	0	1	0	1	0	1	0	0
seedBP10.seedNoGU_IntaRNA	0	0	0	0	1	0	0	1	1	0	0	1	0	0	0	1	1	0	1	0	0	0	1
seedBP11.seedNoGU_IntaRNA	0	0	0	0	0	0	0	0	1	0	0	0	0	0	0	1	1	0	1	0	0	0	0
seedBP12.seedNoGU_IntaRNA	0	0	0	0	0	0	0	0	0	0	0	0	0	0	0	0	0	0	1	0	0	0	0
seedBP13.seedNoGU_IntaRNA	0	0	0	0	0	0	0	0	0	0	0	0	0	0	0	0	0	0	0	0	0	0	0
seedBP14.seedNoGU_IntaRNA	0	0	0	0	0	0	0	0	0	0	0	0	0	0	0	0	0	0	0	0	0	0	0
seedBP4_IntaRNA	1	0	1	0	0	1	0	0	1	1	1	1	0	0	0	0	1	0	1	0	1	0	1
seedBP5_IntaRNA	1	0	0	0	0	1	0	0	1	1	1	1	0	0	0	0	1	0	1	0	1	0	1
seedBP6_IntaRNA	1	0	0	0	0	1	0	1	1	1	1	1	0	0	0	0	1	0	1	0	1	0	1
seedBP7_IntaRNA	1	0	0	0	0	1	0	1	1	1	1	1	0	0	0	0	1	0	1	0	1	0	1
seedBP8_IntaRNA	1	0	1	0	0	1	0	1	1	1	1	1	0	0	0	0	1	0	1	0	1	0	1
seedBP9_IntaRNA	0	0	1	0	0	1	0	1	1	0	1	1	0	0	0	0	1	0	1	0	1	0	1
seedBP10_IntaRNA	0	0	0	0	0	1	1	1	1	0	0	1	0	0	0	0	1	0	1	0	1	0	1
seedBP11_IntaRNA	0	0	0	0	0	1	1	0	1	0	0	0	0	0	0	0	1	0	1	0	1	0	1
seedBP12_IntaRNA	0	0	0	0	0	1	1	0	1	0	0	0	0	0	0	0	0	0	1	0	1	0	1
seedBP13_IntaRNA	0	0	0	0	0	0	1	0	0	0	0	0	0	0	0	0	0	0	0	0	1	0	1
seedBP14_IntaRNA	0	0	0	0	0	0	0	0	0	0	0	0	0	0	0	0	0	0	0	0	0	0	1
seedBP4.intLenMax20_IntaRNA	1	0	0	0	0	1	0	0	1	1	1	1	1	1	0	1	1	0	1	0	1	0	1
seedBP5.intLenMax20_IntaRNA	1	0	0	0	0	1	0	0	1	1	1	1	1	1	0	0	1	0	1	0	1	0	1
seedBP6.intLenMax20_IntaRNA	1	0	0	0	0	1	0	0	1	1	1	1	1	1	0	0	1	0	1	0	1	0	1
seedBP7.intLenMax20_IntaRNA	1	0	0	0	0	1	0	0	1	1	1	1	1	1	0	0	1	0	1	0	1	0	1
seedBP8.intLenMax20_IntaRNA	1	0	0	0	0	1	0	0	1	1	1	1	1	1	0	1	1	0	1	0	1	0	1
seedBP9.intLenMax20_IntaRNA	0	0	0	0	0	1	0	1	1	0	1	1	0	0	0	0	1	0	1	0	1	0	1
seedBP10.intLenMax20_IntaRNA	0	0	0	0	1	1	1	1	1	0	0	1	0	0	0	0	1	0	1	0	1	0	1
seedBP11.intLenMax20_IntaRNA	0	0	0	0	0	1	1	0	1	0	0	0	0	0	0	0	1	0	1	0	1	0	1
seedBP12.intLenMax20_IntaRNA	0	0	0	1	0	1	1	0	1	0	0	0	0	0	0	0	0	0	1	0	1	0	1
seedBP13.intLenMax20_IntaRNA	0	0	0	0	0	0	1	0	0	0	0	0	0	0	0	0	0	0	0	0	1	0	1
seedBP14.intLenMax20_IntaRNA	0	0	0	0	0	0	0	0	0	0	0	0	0	0	0	0	0	0	0	0	0	0	1
seedBP7_TargetRNA2	1	0	0	1	1	1	0	0	0	0	0	0	0	1	0	0	0	0	1	0	0	0	0
seedBP8_TargetRNA2	1	0	0	0	0	0	0	0	0	0	1	0	0	1	0	0	1	0	1	0	1	0	0
seedBP9_TargetRNA2	1	0	0	0	0	0	0	1	1	0	1	0	0	0	0	0	1	0	1	0	1	0	0
seedBP10_TargetRNA2	0	0	0	0	1	0	0	1	1	0	0	1	0	0	0	0	1	0	1	0	0	0	1
seedBP11_TargetRNA2	0	0	0	0	0	0	0	0	1	0	0	0	0	0	0	1	1	0	1	0	0	0	0
seedBP12_TargetRNA2	0	0	0	0	0	0	0	0	0	0	0	0	0	0	0	0	0	0	1	0	0	0	0
seedBP4.seedNoGU_RIsearch2	0	0	0	0	0	0	0	0	0	0	0	0	0	0	0	0	0	0	0	0	0	0	0
seedBP5.seedNoGU_RIsearch2	0	0	0	0	0	0	0	0	0	0	0	0	0	0	0	0	0	0	0	0	0	0	0
seedBP6.seedNoGU_RIsearch2	0	0	0	0	0	0	0	0	0	0	0	0	0	0	0	0	0	0	0	0	0	0	0
seedBP7.seedNoGU_RIsearch2	0	0	0	0	0	0	0	0	0	1	0	0	0	0	0	1	0	0	0	0	0	0	0
seedBP8.seedNoGU_RIsearch2	0	0	0	0	0	0	0	1	0	0	0	0	0	0	0	1	0	0	0	0	0	0	0
seedBP9.seedNoGU_RIsearch2	0	0	0	0	0	0	0	1	0	0	1	0	0	0	0	1	0	0	1	0	0	0	0
seedBP10.seedNoGU_RIsearch2	0	0	0	0	0	0	0	1	0	0	0	1	0	0	0	1	1	0	1	0	0	0	0
seedBP11.seedNoGU_RIsearch2	0	0	0	0	0	0	0	0	0	0	0	0	0	0	0	1	1	0	1	0	0	0	0
seedBP12.seedNoGU_RIsearch2	0	0	0	0	0	0	0	0	0	0	0	0	0	0	0	0	0	0	1	0	0	0	0
seedBP13.seedNoGU_RIsearch2	0	0	0	0	0	0	0	0	0	0	0	0	0	0	0	0	0	0	0	0	0	0	0
seedBP14.seedNoGU_RIsearch2	0	0	0	0	0	0	0	0	0	0	0	0	0	0	0	0	0	0	0	0	0	0	0
seedBP4_RIsearch2	0	0	0	0	0	0	0	0	0	0	0	0	0	0	0	0	0	0	0	0	0	0	0
seedBP5_RIsearch2	0	0	0	0	0	0	0	0	0	0	0	0	0	0	0	0	0	0	0	0	0	0	0
seedBP6_RIsearch2	0	0	0	0	0	0	0	0	0	0	0	0	0	0	0	0	0	0	0	0	0	0	0
seedBP7_RIsearch2	0	0	0	0	0	0	0	0	0	0	0	0	0	0	0	0	0	0	0	0	0	0	0
seedBP8_RIsearch2	0	0	0	0	0	0	0	0	0	0	0	0	0	0	0	0	0	0	0	0	0	0	0
seedBP9_RIsearch2	0	0	0	0	0	0	0	0	0	0	0	0	0	0	0	0	0	0	0	0	0	0	0
seedBP10_RIsearch2	0	0	0	0	0	0	0	0	0	0	0	0	0	0	0	0	0	0	0	0	0	0	0
seedBP11_RIsearch2	0	0	0	0	0	0	0	0	0	0	0	0	0	0	0	0	0	0	0	0	0	0	0
seedBP12_RIsearch2	0	0	0	0	0	1	0	0	0	0	0	0	0	0	0	0	0	0	1	0	0	0	0
seedBP13_RIsearch2	0	0	0	0	0	0	0	0	0	0	0	0	0	0	0	0	0	0	0	0	0	0	0
seedBP14_RIsearch2	0	0	0	0	0	0	0	0	0	0	0	0	0	0	0	0	0	0	0	0	0	0	0
outMaxE0_RIsearch2	0	0	0	0	0	0	0	0	0	0	0	0	0	0	0	0	0	0	0	0	0	0	0
outMaxE20_RIsearch2	0	0	0	0	0	0	0	0	0	0	0	0	0	0	0	0	0	0	0	0	0	0	0

id	ArcZ-STM1682	ArcZ-STM2970	ArcZ-STM3216	ChiX-STM0687	ChiX-STM1313	CyaR-STM0833	GcvB-STM0002	GcvB-STM0245	GcvB-STM0399	GcvB-STM0602	GcvB-STM0665	GcvB-STM0959	GcvB-STM1299	GcvB-STM1452	GcvB-STM1746	GcvB-STM2355	GcvB-STM2526	GcvB-STM3062	GcvB-STM3064	GcvB-STM3225	GcvB-STM3564	GcvB-STM3567	GcvB-STM3630	GcvB-STM3903	GcvB-STM3909	GcvB-STM3930	GcvB-STM4351	GcvB-STM4398	MicA-STM4231	MicC-STM1572	MicF-STM0366	MicF-STM0959	MicF-STM1328	RybB-STM0413	RybB-STM0687	RybB-STM0999	RybB-STM1070	RybB-STM1473	RybB-STM1530	RybB-STM1572	RybB-STM1732	RybB-STM1995	RybB-STM2267	RybB-STM2391	SgrS-STM2945	SgrS-STM3962	Spot42-STM2190
seedBP4.seedNoGU_IntaRNA	0	0	0	1	1	1	0	0	1	0	0	0	0	0	0	0	0	0	1	0	0	1	1	0	1	0	0	1	0	1	0	1	0	1	0	0	0	1	1	0	0	0	1	0	0	0	1
seedBP5.seedNoGU_IntaRNA	0	0	0	1	1	1	0	0	1	0	1	0	0	0	0	0	0	0	1	0	1	1	1	0	1	0	0	1	0	1	0	1	0	1	0	0	0	1	1	0	0	0	1	0	0	0	1
seedBP6.seedNoGU_IntaRNA	0	0	0	1	1	1	0	0	1	0	1	0	0	0	0	1	0	0	1	0	1	1	1	0	1	0	0	1	0	1	1	1	0	1	0	0	0	1	0	0	0	0	1	0	0	0	1
seedBP7.seedNoGU_IntaRNA	0	0	0	1	1	1	0	0	1	0	1	0	0	0	0	1	0	0	1	0	1	1	1	0	1	0	0	1	0	1	0	0	0	1	0	0	0	1	0	0	0	0	1	0	0	0	1
seedBP8.seedNoGU_IntaRNA	0	0	0	1	1	1	0	0	1	0	1	0	0	0	0	1	0	0	1	0	1	1	1	0	1	0	0	1	1	0	0	0	0	0	0	0	0	1	0	0	0	0	1	0	0	0	1
seedBP9.seedNoGU_IntaRNA	0	0	0	1	1	1	1	0	1	0	1	0	0	0	0	1	0	0	1	0	0	0	1	0	1	1	0	1	0	0	0	0	1	0	0	0	0	1	0	0	0	0	1	0	0	0	0
seedBP10.seedNoGU_IntaRNA	0	0	0	1	1	0	1	0	1	1	1	0	0	0	0	1	0	1	0	0	0	0	0	1	1	1	0	1	0	0	0	0	0	0	0	0	0	0	0	0	0	0	1	0	0	0	0
seedBP11.seedNoGU_IntaRNA	0	0	0	1	1	0	0	0	0	1	1	0	0	0	0	0	0	1	0	0	0	0	0	0	1	0	0	0	0	0	0	0	0	0	0	0	0	0	0	0	0	0	0	0	0	0	0
seedBP12.seedNoGU_IntaRNA	0	0	0	1	1	0	0	0	0	1	1	0	0	0	0	0	0	0	0	0	0	0	0	0	1	0	0	0	0	0	0	0	0	0	0	0	0	0	0	0	0	0	0	0	0	0	0
seedBP13.seedNoGU_IntaRNA	0	0	0	0	0	0	0	0	0	0	0	0	0	0	0	0	0	0	0	0	0	0	0	0	0	0	0	0	0	0	0	0	0	0	0	0	0	0	0	0	0	0	0	0	0	0	0
seedBP14.seedNoGU_IntaRNA	0	0	0	0	0	0	0	0	0	0	0	0	0	0	0	0	0	0	0	0	0	0	0	0	0	0	0	0	0	0	0	0	0	0	0	0	0	0	0	0	0	0	0	0	0	0	0
seedBP4_IntaRNA	0	0	0	1	1	1	0	0	1	0	0	0	0	0	0	0	0	0	1	0	0	0	1	0	1	0	0	1	0	1	0	1	0	1	0	0	0	1	1	0	0	0	1	0	0	0	1
seedBP5_IntaRNA	0	0	0	1	1	1	0	0	1	0	0	0	0	0	0	0	0	0	1	0	0	0	1	0	1	0	0	1	0	1	0	1	0	1	0	0	0	1	1	0	0	0	1	0	0	0	1
seedBP6_IntaRNA	0	0	0	1	1	1	0	0	1	0	0	0	0	0	0	0	0	0	1	0	0	0	1	0	1	0	0	1	0	1	0	1	0	1	0	0	0	1	1	0	0	0	1	0	0	0	1
seedBP7_IntaRNA	0	0	0	1	1	1	0	0	1	0	0	0	0	0	0	0	0	0	1	0	0	1	1	0	1	0	0	1	0	1	0	1	0	1	0	0	0	1	1	0	0	0	1	0	0	0	1
seedBP8_IntaRNA	0	0	0	1	1	1	0	0	1	0	0	0	0	0	0	0	0	0	1	0	0	1	1	0	1	0	0	1	0	1	1	1	0	0	0	0	0	1	1	0	0	0	1	0	0	0	1
seedBP9_IntaRNA	0	0	0	1	1	1	0	0	1	0	1	0	0	0	0	0	0	0	1	0	1	1	1	0	1	0	0	1	0	1	1	1	0	0	0	0	0	1	1	0	0	0	1	0	0	0	0
seedBP10_IntaRNA	0	0	0	1	1	0	0	0	1	0	1	0	0	0	0	1	0	0	0	0	1	1	1	0	1	0	0	1	0	1	0	1	0	0	0	0	0	1	1	0	0	0	1	0	0	0	0
seedBP11_IntaRNA	0	0	0	1	1	0	0	0	1	0	1	0	0	0	0	1	0	0	1	0	1	1	1	0	1	0	0	0	0	1	0	0	0	0	0	0	0	1	1	0	0	0	0	0	0	0	0
seedBP12_IntaRNA	0	0	0	1	1	0	0	0	0	0	1	0	0	0	0	0	0	0	1	0	1	0	1	0	1	0	0	0	0	1	0	0	0	0	0	0	0	0	1	0	0	0	0	0	0	1	0
seedBP13_IntaRNA	0	0	0	0	0	0	0	0	0	0	1	0	0	0	0	0	0	0	0	0	1	0	0	0	1	1	0	0	0	0	0	0	0	0	1	0	0	0	0	0	0	0	0	0	0	0	0
seedBP14_IntaRNA	0	0	0	0	0	0	0	0	0	0	0	0	1	0	0	0	0	0	0	0	1	0	0	0	1	1	0	0	0	0	0	0	0	0	0	0	0	0	0	0	0	0	0	1	0	0	0
seedBP4.intLenMax20_IntaRNA	0	0	0	1	1	0	0	0	0	0	1	0	0	0	0	1	1	0	0	0	0	0	0	0	1	0	0	0	0	1	1	1	1	0	0	0	0	1	1	0	0	0	1	0	0	0	1
seedBP5.intLenMax20_IntaRNA	0	0	0	1	1	0	0	0	0	0	1	0	0	0	0	1	1	0	0	0	0	0	0	0	1	0	0	0	0	1	1	1	1	0	0	0	0	1	1	0	0	0	1	0	0	0	1
seedBP6.intLenMax20_IntaRNA	0	0	0	1	1	0	0	0	0	0	1	0	0	0	0	1	1	0	0	0	0	0	0	0	1	0	0	0	0	1	1	1	1	0	0	0	0	1	1	0	0	0	1	0	0	0	1
seedBP7.intLenMax20_IntaRNA	0	0	0	1	1	0	0	0	0	0	1	0	0	0	0	1	1	0	0	0	0	0	0	0	1	0	0	0	0	1	1	1	1	0	0	0	0	1	1	0	0	0	1	0	0	0	1
seedBP8.intLenMax20_IntaRNA	0	0	0	1	1	0	0	0	0	0	1	0	0	0	0	1	1	0	0	0	0	0	0	0	1	0	0	0	0	1	1	1	1	0	0	0	0	1	1	0	0	0	1	0	0	0	1
seedBP9.intLenMax20_IntaRNA	0	0	0	1	1	0	0	0	0	0	1	0	1	0	0	1	1	0	0	0	0	0	0	0	1	0	0	0	0	1	1	1	1	0	0	0	0	1	1	0	0	0	1	0	0	1	0
seedBP10.intLenMax20_IntaRNA	0	0	0	1	1	0	0	0	0	0	1	0	1	0	0	1	1	0	0	0	0	0	0	0	1	0	0	0	0	1	0	0	0	0	0	0	0	1	1	0	0	0	1	0	0	1	0
seedBP11.intLenMax20_IntaRNA	0	0	0	1	1	0	0	0	0	0	1	0	1	0	0	0	1	0	0	0	0	0	0	0	1	0	0	0	0	1	0	0	0	0	1	0	0	1	1	0	0	0	0	0	0	1	0
seedBP12.intLenMax20_IntaRNA	0	0	0	1	0	0	0	0	0	0	1	0	0	0	0	0	1	0	0	1	1	0	0	0	1	0	0	0	0	1	0	0	0	0	1	0	0	0	1	0	0	0	0	0	0	1	0
seedBP13.intLenMax20_IntaRNA	0	0	0	0	0	0	0	0	0	0	1	0	0	0	0	0	0	0	0	1	1	0	0	0	1	0	0	0	0	0	0	0	0	0	1	0	0	0	0	0	0	0	0	1	0	0	0
seedBP14.intLenMax20_IntaRNA	0	0	0	0	0	0	0	0	0	0	0	0	0	0	0	0	0	0	0	0	1	0	0	0	1	0	0	0	0	0	0	0	0	0	0	0	0	0	0	0	0	0	0	1	0	0	0
seedBP7_TargetRNA2	0	0	0	1	1	0	0	0	0	0	0	0	0	0	0	0	1	0	0	0	0	0	0	0	1	1	0	0	0	0	0	0	0	0	0	0	0	0	0	0	0	0	1	0	0	0	0
seedBP8_TargetRNA2	0	0	0	1	1	0	0	0	0	0	1	0	0	0	0	1	0	0	0	0	0	0	1	0	1	1	0	0	0	0	0	0	0	0	0	1	0	0	0	0	0	0	0	0	0	0	1
seedBP9_TargetRNA2	0	0	0	1	1	0	0	0	1	1	1	0	0	0	0	1	0	0	1	0	0	0	1	1	1	0	0	1	0	0	0	0	0	0	0	0	0	1	0	0	0	0	0	0	0	0	0
seedBP10_TargetRNA2	0	0	0	1	1	0	1	1	1	1	1	0	0	0	0	1	0	1	0	0	0	0	0	1	1	1	1	1	0	0	0	0	0	0	0	0	0	0	0	0	0	0	1	0	0	0	0
seedBP11_TargetRNA2	0	0	0	1	1	0	0	0	0	1	1	0	0	0	0	0	0	1	0	0	0	0	0	0	1	0	0	0	0	0	0	0	0	0	0	0	0	0	0	0	0	0	0	0	0	0	0
seedBP12_TargetRNA2	0	0	0	1	1	0	0	0	0	1	1	0	0	0	0	0	0	0	0	0	0	0	0	0	1	0	0	0	0	0	0	0	0	0	0	0	0	0	0	0	0	0	0	0	0	0	0
seedBP4.seedNoGU_RIsearch2	0	0	0	0	0	0	0	1	0	0	0	0	0	0	0	0	0	0	0	0	0	0	1	0	1	0	0	0	0	0	0	0	0	0	0	0	0	0	0	0	0	0	0	0	0	0	0
seedBP5.seedNoGU_RIsearch2	0	0	0	0	0	0	0	0	0	0	1	0	0	0	0	0	0	0	0	0	0	0	1	0	1	0	0	0	0	0	0	0	0	0	0	0	0	0	0	0	0	0	0	0	0	0	0
seedBP6.seedNoGU_RIsearch2	0	0	0	0	0	0	0	0	0	0	1	0	0	0	0	0	0	0	0	0	0	0	1	0	1	0	0	0	0	0	0	0	0	0	0	0	0	0	0	0	0	0	1	0	0	0	0
seedBP7.seedNoGU_RIsearch2	0	0	0	0	1	0	0	0	0	0	1	0	0	0	0	0	0	0	0	0	0	0	1	0	1	0	0	0	0	0	0	0	0	0	0	0	0	0	0	0	0	0	1	0	0	0	0
seedBP8.seedNoGU_RIsearch2	0	0	0	0	1	0	0	0	0	0	1	0	0	0	0	0	0	0	0	0	0	0	1	0	1	0	0	0	0	0	0	0	0	0	0	0	0	0	0	0	0	0	0	0	0	0	0
seedBP9.seedNoGU_RIsearch2	0	0	0	1	1	0	0	0	0	0	1	0	0	0	0	0	0	0	0	0	0	0	1	1	1	1	0	1	0	0	0	0	0	0	0	0	0	0	0	0	0	0	0	0	0	0	0
seedBP10.seedNoGU_RIsearch2	0	0	0	1	1	0	0	0	0	1	1	0	0	0	0	1	0	1	0	0	0	0	0	1	1	1	0	1	0	0	0	0	0	0	0	0	0	0	0	0	0	0	1	0	0	0	0
seedBP11.seedNoGU_RIsearch2	0	0	0	1	1	0	0	0	0	1	1	0	0	0	0	0	0	1	0	0	0	0	0	0	1	0	0	0	0	0	0	0	0	0	0	0	0	0	0	0	0	0	0	0	0	0	0
seedBP12.seedNoGU_RIsearch2	0	0	0	1	1	0	0	0	0	1	1	0	0	0	0	0	0	0	0	0	0	0	0	0	1	0	0	0	0	0	0	0	0	0	0	0	0	0	0	0	0	0	0	0	0	0	0
seedBP13.seedNoGU_RIsearch2	0	0	0	0	0	0	0	0	0	0	0	0	0	0	0	0	0	0	0	0	0	0	0	0	0	0	0	0	0	0	0	0	0	0	0	0	0	0	0	0	0	0	0	0	0	0	0
seedBP14.seedNoGU_RIsearch2	0	0	0	0	0	0	0	0	0	0	0	0	0	0	0	0	0	0	0	0	0	0	0	0	0	0	0	0	0	0	0	0	0	0	0	0	0	0	0	0	0	0	0	0	0	0	0
seedBP4_RIsearch2	0	0	0	1	0	0	0	1	0	0	0	0	0	0	0	0	0	0	0	0	0	0	0	0	1	0	0	0	0	0	0	0	0	0	0	0	0	0	0	0	0	0	0	0	0	0	0
seedBP5_RIsearch2	0	0	0	1	0	0	0	1	0	0	0	0	0	0	0	0	0	0	0	0	0	0	1	0	1	0	0	0	0	0	0	0	0	0	0	0	0	0	0	0	0	0	0	0	0	0	0
seedBP6_RIsearch2	0	0	0	1	0	0	0	1	0	0	0	0	0	0	0	0	0	0	0	0	0	0	1	0	1	0	0	0	0	0	0	0	0	0	0	0	0	0	0	0	0	0	0	0	0	0	0
seedBP7_RIsearch2	0	0	0	1	0	0	0	0	0	0	0	0	0	0	0	0	0	0	0	0	0	0	1	0	1	0	0	0	0	0	0	0	0	0	0	0	0	0	0	0	0	0	0	0	0	0	0
seedBP8_RIsearch2	0	0	0	1	0	0	0	0	0	0	0	0	0	0	0	0	0	0	0	0	0	0	1	0	1	0	0	0	0	0	0	1	0	0	0	0	0	0	0	0	0	0	0	0	0	0	0
seedBP9_RIsearch2	0	0	0	0	0	0	0	0	0	0	0	0	0	0	0	0	0	0	0	0	0	0	1	0	1	0	0	0	0	0	0	1	0	0	0	0	0	0	0	0	0	0	0	0	0	0	0
seedBP10_RIsearch2	0	0	0	0	0	0	0	0	0	0	1	0	0	0	0	0	0	0	0	0	0	0	0	0	1	0	0	0	0	0	0	0	0	0	0	0	0	0	0	0	0	0	0	0	0	0	0
seedBP11_RIsearch2	0	0	0	0	1	0	0	0	0	0	1	0	0	0	0	0	0	0	0	0	0	0	0	0	1	0	0	0	0	0	0	0	0	0	0	0	0	0	0	0	0	0	0	0	0	0	0
seedBP12_RIsearch2	0	0	0	0	1	0	0	0	0	0	1	0	0	0	0	0	0	0	0	0	0	0	0	0	1	0	0	0	0	0	0	0	0	0	0	0	0	0	0	0	0	0	0	0	0	0	0
seedBP13_RIsearch2	0	0	0	0	0	0	0	0	0	0	1	0	0	0	0	0	0	0	0	0	0	0	0	0	1	1	0	0	0	0	0	0	0	0	0	0	0	0	0	0	0	0	0	1	0	0	0
seedBP14_RIsearch2	0	0	0	0	0	0	0	0	0	0	0	0	0	0	0	0	0	0	0	0	1	0	0	0	1	1	0	0	0	0	0	0	0	0	0	0	0	0	0	0	0	0	0	1	0	0	0
outMaxE0_RIsearch2	0	0	0	1	0	0	0	1	0	0	0	0	0	0	0	0	0	0	0	0	0	0	1	0	1	0	0	0	0	0	0	0	0	0	0	0	0	0	0	0	0	0	0	0	0	0	0
outMaxE20_RIsearch2	0	0	0	1	0	0	0	1	0	0	0	0	0	0	0	0	0	0	0	0	0	0	1	0	1	0	0	0	0	0	0	0	0	0	0	0	0	0	0	0	0	0	0	0	0	0	0
